# Boolean model of growth signaling, cell cycle and apoptosis predicts the molecular mechanism of aberrant cell cycle progression driven by hyperactive *PI3K*

**DOI:** 10.1371/journal.pcbi.1006402

**Published:** 2019-03-15

**Authors:** Herbert Sizek, Andrew Hamel, Dávid Deritei, Sarah Campbell, Erzsébet Ravasz Regan

**Affiliations:** 1 Biochemistry and Molecular Biology, The College of Wooster, Wooster, OH, United States of America; 2 Department of Physics, Pennsylvania State University, State College, PA, United States of America; 3 Department of Network and Data Science, Central European University, Budapest, Hungary; University of Virginia, UNITED STATES

## Abstract

The *PI3K*/*AKT* signaling pathway plays a role in most cellular functions linked to cancer progression, including cell growth, proliferation, cell survival, tissue invasion and angiogenesis. It is generally recognized that hyperactive *PI3K*/*AKT1* are oncogenic due to their boost to cell survival, cell cycle entry and growth-promoting metabolism. That said, the dynamics of *PI3K* and *AKT1* during cell cycle progression are highly nonlinear. In addition to negative feedback that curtails their activity, protein expression of *PI3K* subunits has been shown to oscillate in dividing cells. The low-*PI3K*/low-*AKT1* phase of these oscillations is required for cytokinesis, indicating that oncogenic *PI3K* may directly contribute to genome duplication. To explore this, we construct a Boolean model of growth factor signaling that can reproduce *PI3K* oscillations and link them to cell cycle progression and apoptosis. The resulting modular model reproduces hyperactive *PI3K*-driven cytokinesis failure and genome duplication and predicts the molecular drivers responsible for these failures by linking hyperactive *PI3K* to mis-regulation of Polo-like kinase 1 (*Plk1*) expression late in G2. To do this, our model captures the role of *Plk1* in cell cycle progression and accurately reproduces multiple effects of its loss: G2 arrest, mitotic catastrophe, chromosome mis-segregation / aneuploidy due to premature anaphase, and cytokinesis failure leading to genome duplication, depending on the timing of *Plk1* inhibition along the cell cycle. Finally, we offer testable predictions on the molecular drivers of *PI3K* oscillations, the timing of these oscillations with respect to division, and the role of altered *Plk1* and *FoxO* activity in genome-level defects caused by hyperactive *PI3K*. Our model is an important starting point for the predictive modeling of cell fate decisions that include *AKT1*-driven senescence, as well as the non-intuitive effects of drugs that interfere with mitosis.

## Author summary

Complex diseases such as cancer often alter more than one facet of a cell’s function. In addition to breakdown in individual functions, cancer progression leads to unhealthy combinations of cellular behaviors. For example, cancer cells rely on non-physiological combinations of cell functions drawn from an arsenal that includes proliferation, resistance to apoptosis, migration, and blood vessel recruitment. These functions are all critical to health or development, often in a different tissue than that of the tumor. Building predictive models that reproduce this coordination of functions could greatly boost our ability to combat complex disease. Here, we develop a large network model of the processes that control a mammalian cell’s life and death. Our model reproduces a non-intuitive oscillation in a key cell division pathway (*PI3K*/*AKT1*), along with the cell-cycle altering effect of its oncogenic activation. To do this, we incorporate the role of *Polo-like kinase 1* (mitotic driver, chemotherapy target) and model mitotic failure when *Plk1* is blocked. Finally, we offer testable predictions on the unexplored drivers of *PI3K* oscillations, their timing with respect to division, and the mechanism by which hyperactive *PI3K* leads to genome-level defects. Thus, our work can aid development of powerful models that cover most processes that go awry when cells transition into malignancy.

## Introduction

Mammalian cells require extracellular growth signals to divide and specific survival signals to avoid programmed cell death (apoptosis) [1]. The pathways leading to proliferation, quiescent survival or apoptosis are not fully independent; rather, they have a large degree of crosstalk. For example, most pathways activated by mitogenic signals such as *PI3K* → *AKT1* and *MAPK* signaling also promote survival [2,3]. Moreover, regulatory proteins required for normal cell cycle progression such as *E2F1*, *Myc* and cyclin-dependent kinases (CDKs) can promote apoptosis as well [4,5]. Conversely, cell cycle inhibitors such as *p16*^*INK4a*^ can enhance survival [6]. As several of our most intractable diseases—cancer, cardiovascular problems and cellular aging-related complications—all involve dysregulation of these processes [7,8], creating predictive models to characterize them has been an ongoing focus for computational and systems biology. Approaches that couple computational modeling with experimental validation have made impressive strides in deciphering the networks in charge of cell cycle progression [9–11] and apoptosis [12–15], as well as the mechanisms of cell cycle arrest in response to stressors such as DNA damage [16–20]. Building on these efforts, our collective focus is increasingly shifting from models that describe individual functions towards ones that successfully integrate several aspects of cellular behavior [21–28]. These integrated models aim to predict the context-dependent outcomes of the crosstalk between different subsystem of large signaling networks, along with the knock-on effects of perturbing one subsystem on others. Furthering this effort, here we offer a comprehensive model of the nonlinear dynamics of *PI3K* → *AKT1* ⊣ *FoxO* signaling coupled to the cell cycle. Our model can reproduce non-intuitive phenotypic effects of oncogenic *PI3K* [29], and offer testable predictions about the molecular mechanism responsible for them.

**Fig 1:**
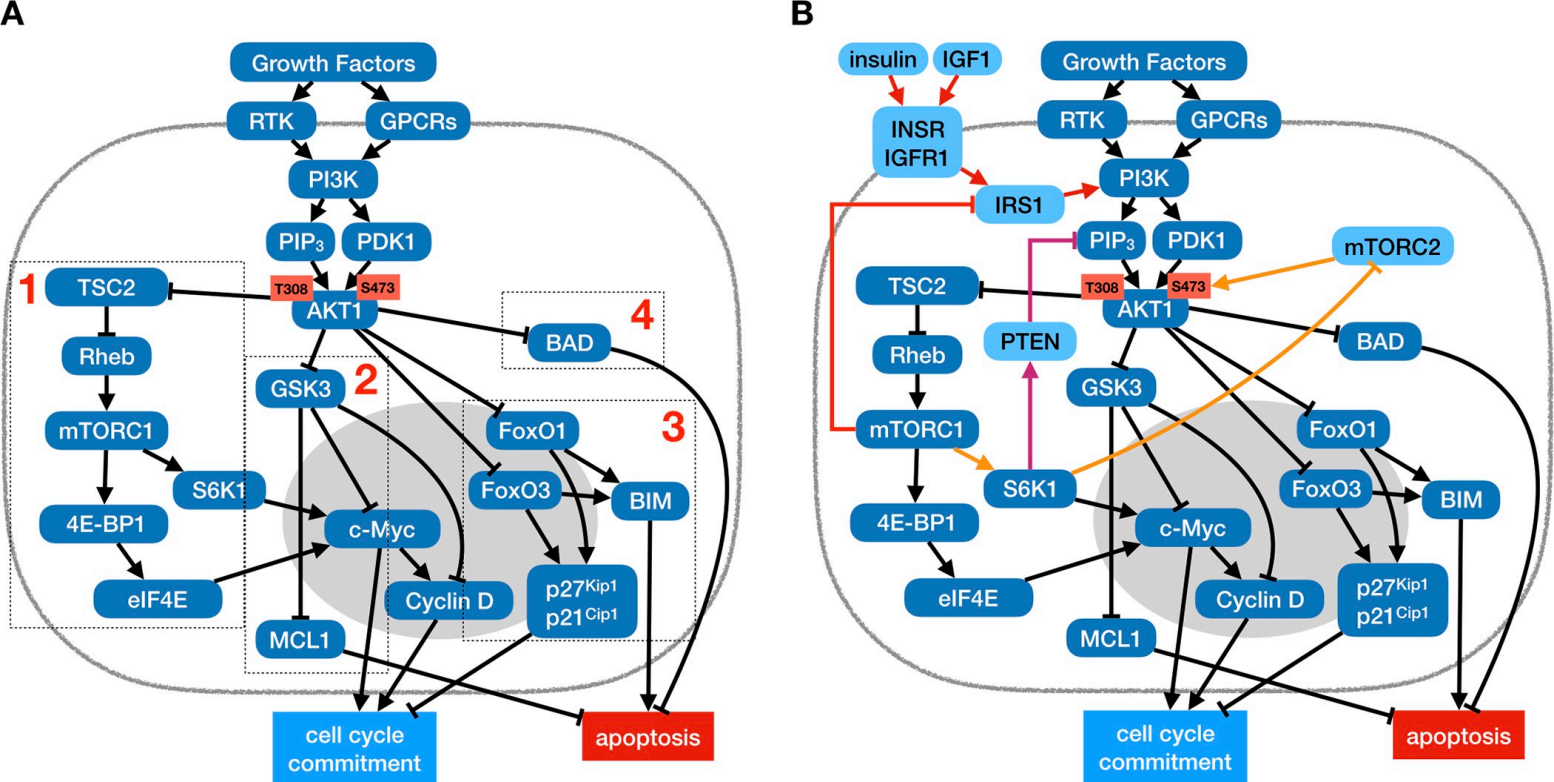
*PI3K* → *AKT1* signaling is regulated by multiple layers of negative feedback that fine-tune its control of growth, cell cycle commitment and survival. (A) Feed-forward network of interactions from growth receptors to *PI3K* and *AKT1* (detailed description of molecular mechanisms in *Methods & Model*). *Box 1*: *AKT1* activates the *mTORC1* pathway, driving volume growth; *box 2*: *AKT1* blocks the *GSK3β* pathway responsible for dampening cell cycle entry and survival signaling; *box 3*: *AKT1* blocks the *FoxO* transcription factors that drive expression of anti-proliferative and pro-apoptotic genes; *box 4*: *AKT1* promotes cell survival by keeping the pro-apoptotic protein *BAD* in check. (B) The *mTORC1* pathway feeds back to dampen *PI3K* → *AKT1* signaling by mediating the degradation of insulin receptor substrates (*red arrows*), aiding the cytoplasmic translocation of *PTEN* (*purple arrows*) and dampening *mTORC2* activation (*orange arrows*).

The canonical *PI3K* → *AKT1* pathway is a major relay for growth and survival signals (**Fig 1A**) [30], as phosphorylated *AKT1* has more than a hundred known direct targets [31,32]. *First*, *AKT1* promotes the cell growth required for division and tissue growth, primarily by activating the *mTORC1* signaling complex. Provided that the cell is not experiencing amino acid or energy deprivation, *mTORC1* aids cell cycle commitment (**Fig 1A**, box 1), and orchestrates changes in cellular growth metabolism by increasing protein synthesis, lipid and nucleotide metabolism, and mitochondrial biogenesis [33,34]. *Second*, *AKT1* inhibits *GSK3β*, counteracting its destabilizing effects on cell cycle-promoting and anti-apoptotic genes (**Fig 1A**, box 2) [35]. *Third*, *AKT1* aids cell cycle entry and survival by translocating the *FoxO* family of transcription factors out of the nucleus, thus decreasing cell cycle inhibitor and pro-apoptotic gene expression (**Fig 1A**, box 3) [36]. *Fourth*, *AKT1* phosphorylates the pro-apoptotic *BAD*, blocking its mitochondrial localization (**Fig 1A**, box 4) [37].

The array of signaling events described above all point to a coherent role of *AKT1* in promoting survival and proliferation. There is mounting evidence, however, that *PI3K* → *AKT1* activity during cell cycle progression is more complex [31,32]. Overactive *AKT1* in cancer cells has been associated with driving cells into senescence (an aging cell state characterized by permanent cell cycle arrest) [38,39]. More intriguing are studies showing that active *FoxO3* and/or *FoxO1* not only block cell cycle entry but are paradoxically required for its subsequent completion. A study by Alvarez *et al* has shown that the attenuation of the *PI3K* → *AKT1* pathway after restriction point passage was required for *FoxO3* activity in G2, which in turn aided the completion of cytokinesis [29]. To explain their observations, the authors showed that *FoxO3* upregulates the expression of the mitotic *cyclin B* and polo-like kinase 1 (*Plk1*), thus promoting the G2/M transition and progression out of telophase. Furthermore, work by Yuan *et al* also implicated *FoxO1* in G2-phase *Plk1* regulation [40]. This effect, however, may be short lived, as both *FoxO* factors are inhibited by *Plk1* phosphorylation [41,42]. To capture these subtleties in a model, we first need to understand the mechanisms that generate a short-lived *AKT1* pulse.

There are several known feedback mechanisms that can explain the pulse-like spike and subsequent attenuation of *AKT1* following growth factor stimulation (**Fig 1B**). Most of these involve *mTORC1*, and several are specific to insulin and *IGF1* signaling [32]. For example, *mTORC1* is known to mediate the degradation of insulin receptor substrates *IRS1/2* required for insulin and *IGF1* signaling (**Fig 1B**, red arrows) [43]. In addition, inhibition of *FoxO* transcription downstream of *AKT1* leads to attenuated transcription of insulin and *IGF1* receptors [44]. To further complicate the picture, activation of the *mTORC1* target *S6K* sets in motion two growth receptor-independent negative feedback loops. First, *S6K* can attenuate *mTORC2* activity required for full *AKT1* activation (**Fig 1B**, orange arrows) [42,43]. Second, *S6K* can promote nuclear export of the *PI3K* inhibition *PTEN* (**Fig 1B**, purple arrows) [45]. Together, these mechanisms are thought to dampen *AKT1* activation following an initial peak and regulate the homeostatic maintenance of *AKT1* under ongoing growth stimulation.

In addition to feedback downstream of *AKT1*, a study by Yuan *et al* has demonstrated that even *before AKT1* signaling downstream of growth receptors has a chance to engage, only a relatively small subpopulation of cells (~30%) are responsive to these signals to begin with [46]. The remaining cells do not suffer from lack of receptor activation or lack of *AKT1* protein; rather, they have very low levels of the *PI3K* subunit *p110* at the time of stimulation. More surprising was their finding that as *AKT1* peaked in the responding population of cells, the initially high *p110* underwent rapid degradation. This effect essentially co-occurred with *AKT1* activation; a process too rapid for the feedback mechanisms downstream of AKT1 to mediate. Moreover, the feedback detailed above acts on *AKT1* phosphorylation and/or *PI3K* activity, not on their protein expression. Yuan *et al* showed that the cycle of rapid *p110* degradation and subsequent re-synthesis was mandatory for sustaining proliferation, as clonal populations with degradation-resistant *p110* and sustained peak *AKT1* activity entered senescence at a high rate. Importantly, their findings were not restricted to a single growth receptor, pointing to a general, yet unrecognized set of feedback loops driving the expression cycle of *p110*. Finally, their work indicated that *p110* heterogeneity in quiescent cells is strongly influenced by local cell density. Another study confirms that the catalytic *p110* subunit of *PI3K* is indeed rapidly degraded upon growth stimulation in two additional cell lines, and that it re-accumulates slowly (~2 hours) [47]. This work points to the *NEDDL4* ubiquitin ligase as the driver of *p110* degradation [47]. In addition, *AKT1* phosphorylation has been shown to exhibit at least two clear peaks before the end of S-phase in cells entering the cell cycle from quiescence [29], an effect observable despite rapid de-synchronization of the cell culture. These studies, however, do not address the molecular mechanisms that trigger *p110* degradation specifically in response to growth factor signaling, its subsequent re-synthesis, or the way it’s oscillations interface with cell cycle control.

Current computational models of the regulation of mammalian cell life and death do not account for dynamic *p110* expression [16]. Models that incorporate feedback on *AKT1* activity typically focus on the intricacies of the *mTORC1* / *mTORC2* crosstalk [48] or the effects of negative feedback on the strength of *AKT1* signaling [49,50], but do not encompass the full cell cycle. Here, we put forth a large Boolean model of the regulatory interactions driving dynamic growth factor signaling, cell cycle progression and apoptosis. We built the model by bringing together several separately published, disconnected pieces of evidence regarding *p110* protein and mRNA regulation [47,51,52]. We then linked the resulting growth signaling layer to an updated Boolean cell cycle model [11], as well as the molecular network responsible for survival vs. apoptosis. The resulting Boolean model reproduces the cell-cycle dependent role of *PI3K*, *AKT1* and *FoxO* proteins [29,46] by linking them to *Plk1* regulation in G2. As expected, it generates straightforward behaviors such as lack of cell cycle commitment in the absence of high *p110* expression [46], or G1 shortening in the presence of hyperactive *PI3K* / *AKT1*. The novelty and value of our model, however, stems from its ability to reproduce more intricate, non-intuitive phenotypic outcomes. *First*, our model reproduces the path to apoptosis in the event of a mitotic catastrophe [53]. *Second*, our model generates four distinct cell fates in response to *Plk1* inhibition, depending on the timing of *Plk1* loss [54]: i) G2 arrest [55], ii) mitotic catastrophe [54,56–58], iii) premature anaphase and chromosome mis-segregation leading to aneuploidy [59], and iv) failure to complete cytokinesis following telophase [60–62], which can lead to genome duplication [59]. *Third*, our model can replicate failure of cytokinesis and accumulation of binucleate telophase cells driven by hyperactive *PI3K* / *AKT1* or by *FoxO3* inhibition [29].

Our model’s ability to accurately reproduce a range of cell fates triggered by altered *PI3K*, *AKT1*, *FoxO3* or *Plk1* activity leads to several experimentally testable predictions. Namely, we predict 1) the molecular mechanisms of *p110* degradation in response to high *PI3K* activation, and the transcriptional driver of its re-synthesis; 2) that the degradation / re-synthesis cycle of *p110* occurs at least twice per division cycle (along with the molecular mechanism for their phase-locking); 3) that cell cycle defects in response to *PI3K* / *AKT1* over-activation or *FoxO3* knockdown are generally due to a loss of *Plk1* in telophase; 4) loss of strong growth signaling in *p110*-overexpressing cells allows for normal cell cycle completion; and finally, 5) that cells in which *p110* is inhibited after the start of DNA synthesis can still pre-commit to another cell cycle in the presence of saturating growth stimulation.

## Results

In order to build a mechanistic model of the dynamics of growths signaling and its influence on cell cycle progression and apoptosis, we turned to Boolean modeling [63]. Using a modular approach proposed in [11], we first collected key growth signaling pathways driving cell cycle commitment in a *Growth Signaling module* responsible for the dynamics of *PI3K*, *AKT1*, *MAPK* and *mTORC*. Next, we identified key regulatory subsystems that control cell cycle progression, such as the *Restriction Switch* driving the initial commitment to DNA synthesis [11], the *Phase Switch* driving cell cycle progression from G2 to M and back to G1 [11] (expanded from [11] to account for the mitotic role of *Plk1* [54]), and a regulatory switch that tracks the licensing and firing of replication origins. Finally, we synthesized several published models of the survival vs. apoptosis decision into an *Apoptotic Switch*. These modules are tied together into an 87-node network by direct regulatory crosstalk, as well as a few nodes that represent cellular processes we do not track in molecular detail (e.g., DNA Replication, mitotic spindle assembly or cytokinesis). Following a detailed description of our model, we show that it faithfully reproduced quiescent, apoptotic and dividing cell phenotypes, and that its behavior is robust under synchronous or asynchronous update. To understand the role of dynamic *PI3K* signaling in healthy cell cycle progression, we then explore the consequences of *Plk1* inhibition at different points along the cell cycle and show that the non-intuitive consequences of *PI3K* hyperactivation are explained by mild *Plk1* inhibition in G2/M.

### Modeling the dynamic regulation of *p110* expression during growth factor signaling

**Fig 2:**
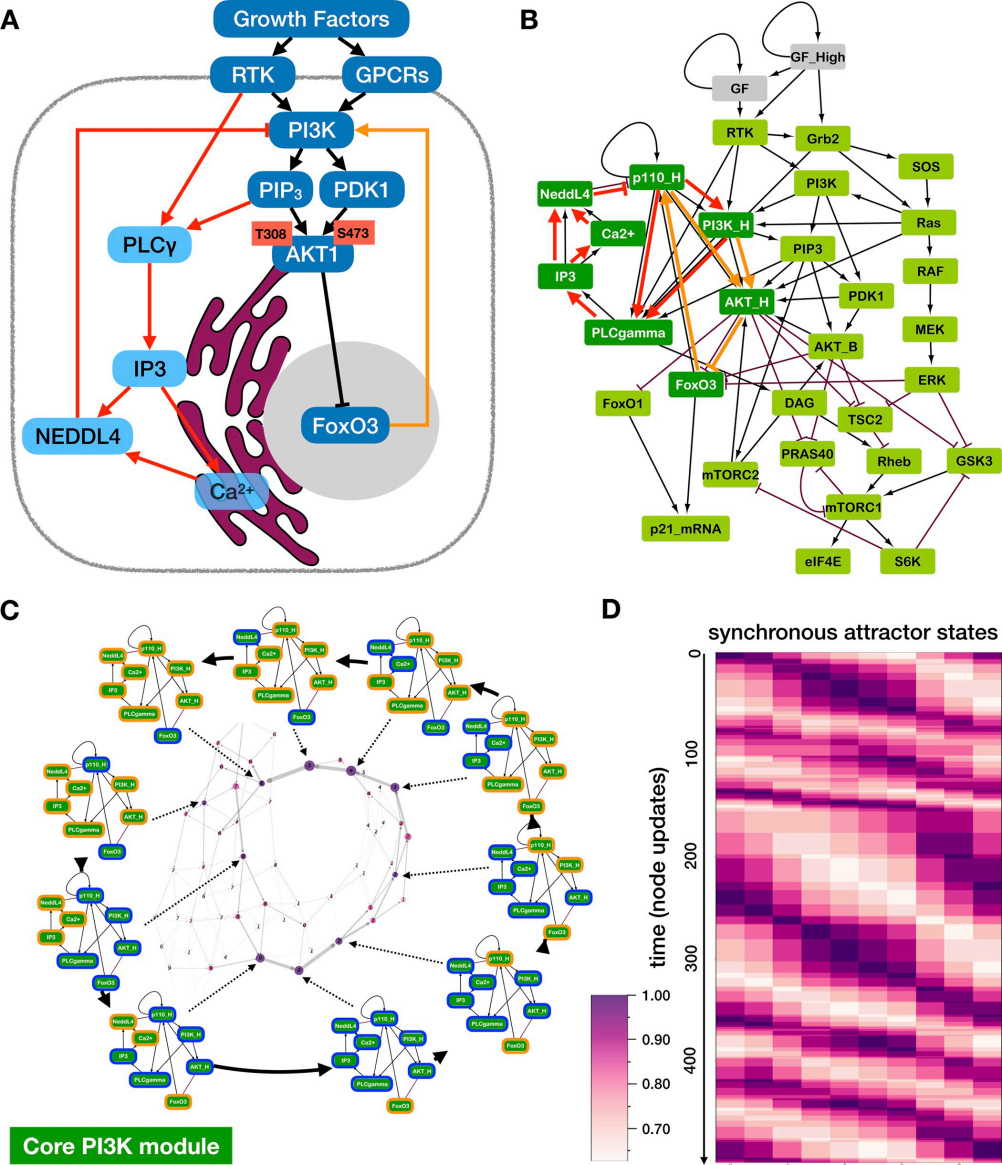
Two hypothesized negative feedback loops control degradation and re-synthesis of *PI3K* and *AKT1* signaling. (A) Degradation of the *PI3K* subunit *p110* may be driven by the *PLCγ*-dependent activation of the *NEDDL4* ubiquitin ligase (*red links*); re-synthesis of *p110* may be driven by *FoxO3*, which re-enters the nucleus following *p110* degradation, as *AKT1* activity falls (*orange link*). (B) *Growth Signaling Module* of our Boolean model, including the degradation/re-synthesis circuit in control of *p110* expression (*left*, *dark green*), basal *PI3K/AKT1* signaling (*middle*), downstream effectors of *AKT1* (*mTORC1* signaling, *GSK3* & *FoxO1*, *bottom*), and the *MAPK* cascade (*right*). *Black* →: activation; red ⊣: inhibition; *thick red links*: *p110* degradation; *thick orange loop*: *p110* re-synthesis. (C) *Periphery*: sequence of network states along the synchronous limit cycle of the core *PI3K* circuit. *Orange/blue borders*: ON (expressed and/or active) / OFF (not expressed and/or inactive) node. *Middle*: state transition graph of the general asynchronous model (one random node updated per timestep; sampled for 10,000 steps), yielding a complex limit cycle that follows the synchronous cycle. *Node size*: visitation frequency; *label*: most similar synchronous cycle state; *node color*: overlap of similar synchronous cycle state (one minus normalized Hamming distance); *layout*: Kamada-Kawai algorithm (NetworkX [67], Python). (D) Overlap of states along a general asynchronous update trajectory (*y* axis) with each attractor state along the synchronous limit cycle (*x* axis). *Time-step*: update of a single random node.

In order to build a *Growth Signaling* module that incorporates the molecular drivers of *p110* dynamics, we turned to the literature in search of mechanisms that can drive rapid *p110* degradation and gradual re-synthesis (**Fig 2A**). Both the free and *p85*-bound versions of the *PIK3CA* (*p110α*) subunit of *PI3K* have been shown to undergo proteasome-dependent degradation triggered by the E3 ubiquitin ligase *NEDDL4* [47]. The activity of *NEDDL4*, in turn, requires Ca^2+^ and inositol trisphosphate (IP3) [51]. This led us to hypothesize that the ability of *NEDDL4* to ubiquitinate *p110* spikes in response to sudden growth factor stimulation. Namely, growth receptors activate phospholipase C γ (*PLCγ*), an enzyme that generates IP3 from membrane-bound PIP2. IP3 diffuses to the endoplasmic reticulum, where it triggers Ca^2+^ release into the cytosol [64]. Thus, IP3 and Ca^2+^ are available to activate *NEDDL4* within minutes of receptor activation, leading to rapid *p110* degradation. As membrane tethering of *PLCγ* requires *PIP3*—a product of active *PI3K* [65,66], the cascade leading to the polyubiquitination of *p110* can only occur in cells that express high levels of *p110* when growth signals arrive (as observed by Yuan *et al* [46]). To summarize, we posit that strong *PI3K* activation initiates a negative feedback loop leading to its own degradation, independently of its effect on *AKT1* (**Fig 2A,** red links).

Next, we turned to the mechanism of *p110* re-synthesis. Studies of the *p110α* promoter indicate that this gene is positively regulated by *FoxO3* [52]. We hypothesized that reactivation of *FoxO3* in the G2 phase of the cell cycle, after the initial *AKT1* activation subsides, is the driving force behind *p110* re-synthesis (**Fig 2A,** orange link). To integrate these negative feedback loops with the canonical *PI3K* / *AKT1* signaling cascade activated by growth receptors, we introduced separate Boolean nodes to track basal vs. peak *PI3K* and *AKT1* activity (**Fig 2B;** Boolean gates: **S1A Table**). Our model can thus distinguish between survival signaling in a low growth factor environment (where basal *PI3K* and *AKT1* are ON) and peak *PI3K/AKT1* activation following the arrival of a strong mitogenic stimulus. Complemented by a linear *MAPK* cascade and *mTORC*1/2 signaling, this non-linear *PI3K/AKT1* axis dominates the behavior of the resulting Boolean *Growth Signaling* module (**Fig 2B**).

Modeling the two feedback loops controlling *p110* expression in isolation shows that they generate a sustained, robust oscillation (**Fig 2C**), even though our model does not account for the fact that *p110* degradation is significantly faster than its re-synthesis. This oscillation is the only attractor state of the small module regardless of Boolean update. As **Fig 2C** indicates, the synchronous attractor cycle clearly maps onto the cyclic succession of complex attractor states of the general asynchronous model (**Fig 2C**, weighted, directed network in the middle). In addition to never leaving the complex attractor shown on **Fig 2C**, asynchronous time series repeatedly walk through cycles of states that resemble the synchronous limit cycle (**Fig 2D**). Within the context of the larger *Growth Signaling* module, this oscillation only occurs under ongoing high growth factor stimulation.

### Modeling cell cycle commitment, the licensing of replication origins and the survival/apoptosis switch

In order to investigate the downstream consequences of an oscillating *Growth Signaling* module, we next updated our previously published cell cycle model [11] and extended it with an apoptotic switch (described in detail in *Methods & Models*).

#### Core cell cycle switches

**Fig 3:**
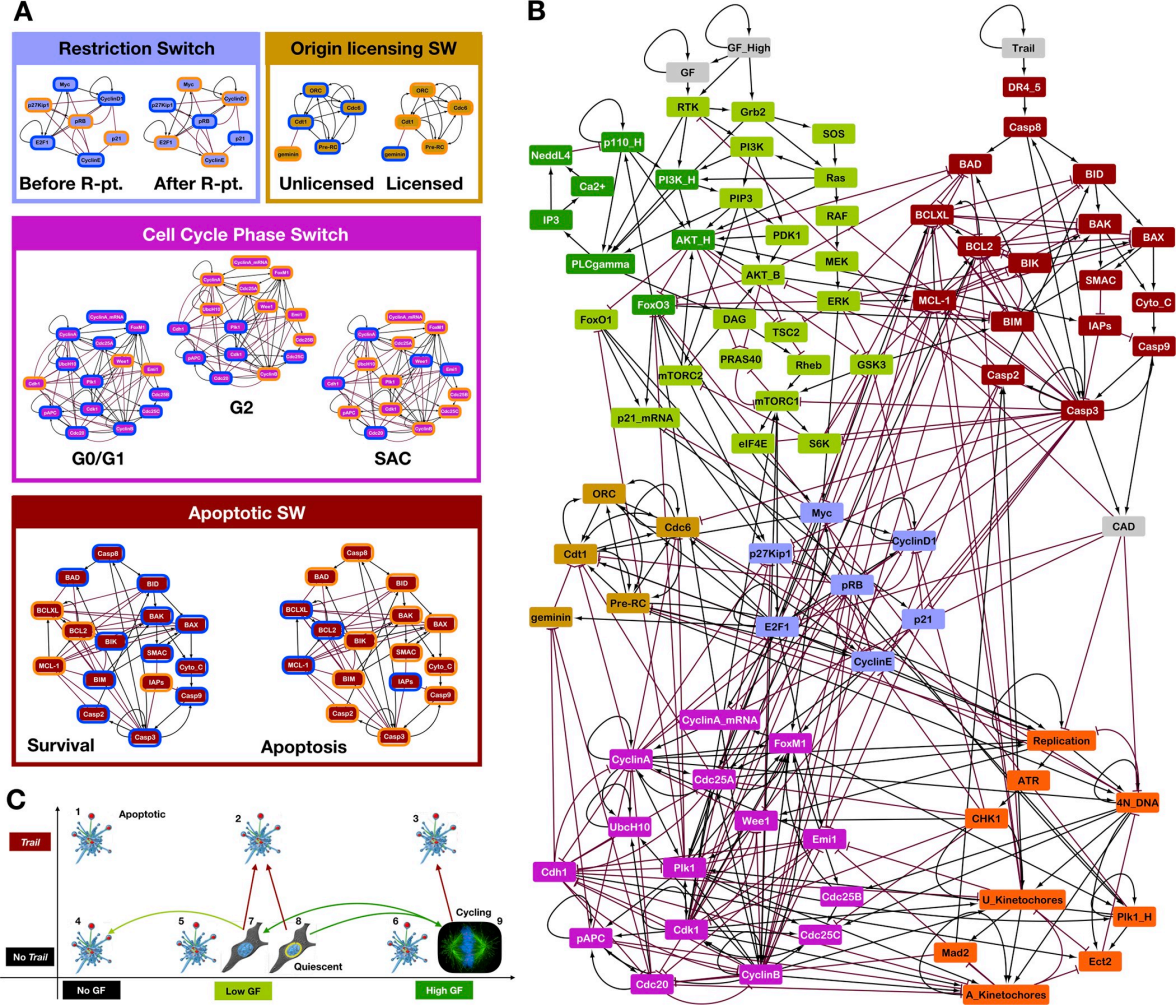
Modular Boolean model reproduces the expected quiescent, apoptotic, and cell cycle phenotypes in various extracellular environments. (A) Stable attractor states of isolated regulatory switches. *Blue / light brown / purple / dark red boxes*: stable states of the *Restriction / Origin of Replication Licensing / Phase / Apoptotic Switch*. *Orange / blue node border*: ON / OFF state. (B) Network representation of the Boolean model partitioned into regulatory switches and processes. *Gray*: inputs representing environmental factors; *green*: Growth Signaling; *dark red*: Apoptotic Switch; *light brown*: Origin of Replication Licensing Switch; *blue*: Restriction Switch; *purple*: Phase Switch; *orange*: cell cycle processes and molecules that bridge between the multi-stable modules. *Black* →: activation; *red* ⊣: inhibition. (C) Cell phenotypes predicted for every combination of no/low/high growth-factor (*x* axis) and *Trail* exposure (*y* axis). The network-wide ON/OFF states of each attractor and the molecular signatures that define their phenotypes are detailed in **S2 Table**. *Blue fragmented cell*: apoptotic states (#1–6); *gray elongated cell*: quiescent/non-dividing states (#7–8); *cell with mitotic spindle*: cell undergoing repeated cycles (#9). *Yellow circle around nucleus*: 4N DNA content; *double-/single-headed arrows between cells*: reversible/ irreversible phenotypic transitions in response to changing environments; *green arrow*: change in growth factor levels; *red*: change in *Trail* exposure. *Image credits*: apoptotic cell [78]; quiescent cell: *https://en.wikipedia.org/wiki/Cell_culture#/media/File:HeLa_cells_stained_with_Hoechst_33258.jpg*; mitotic spindle: *https://en.wikipedia.org/wiki/Cell_division#/media/File:Kinetochore.jpg*.

First, we modeled the switch-like restriction point control guarding cell cycle entry by reusing the *p21*-positive version of our published *Restriction Switch* (**Fig 3**, blue subgraph & box; Boolean gates: **S1B Table**) [11]. In isolation this module has two stable states corresponding to cell states before and after restriction point passage. Second, we expanded our published tri-stable *Phase Switch* driving mitotic entry and exit to account for the regulation and key functions of Polo kinase 1 (*Plk1*) (**Fig 3A**, purple subgraph & box; Boolean gates: **S1C Table**) [54]. *Plk1* is activated in early G2 by the *FoxM1* transcription factor [68]. In addition, decreased *Plk1* expression in the absence of *FoxO3* [29] and/or *FoxO1* [40] during G2 connects *Plk1* availability to the dynamics of *PI3K* → *AKT1* ⊣ *FoxO* signaling. The updated *Phase Switch* retains three stable point attractors, matching the activity pattern of this network in G0/G1, at the G2 checkpoint, and at the Spindle Assembly Checkpoint (SAC). Third, we built a small switch that tracks the assembly, licensing and firing of replication origins (**Fig 3A**, brown subgraph & box; Boolean gates: **S1D Table**). This two-state switch reproduces the stability of assembled Pre-Origin of Replication Complexes; its stable states correspond to unlicensed and licensed origins. Fourth, we accounted for the progression and completion of cell cycle processes not modeled in molecular detail (**Fig 3B**, orange nodes; Boolean gates: **S1E Table**). The *Replication* and *4N_DNA* nodes track DNA synthesys [11]; the unattached kinetochore node (*U_Kinetochore*) denotes incomplete mitotic spindle assembly and ongoing metaphase, while the attached kinetochore node (*A_Kinetochore*) marks completion of the mitotic spindle. Finally, key regulators of the coupling between regulatory switches and cell cycle processes, such as S-phase checkpoint signaling (*Chk1*), the unattached kinetochore sensor *Mad2*, and a marker of contractile ring assembly and cytokinesis (*Ect2*) further link the modules.

#### The apoptotic switch

To account for the apoptotic effects of growth factor withdrawal and death due to mitotic failure, we synthesized published models of apoptotic commitment to create a detailed Boolean regulatory switch (**Fig 3B**, dark red subgraph & box; Boolean gates: **S1F Table**) [12–15,69–71]. This switch has two stable states corresponding to *survival* and *apoptosis*, and it is flipped when extrinsic signals from death receptors, or intrinsic signals due to mitotic failure trigger Mitochondrial Outer Membrane Permeabilization (MOMP), leading to the activation of executioner *Caspase 3* [12]. While the positive feedback loops that stabilize apoptosis are common to most published models, the signals that trigger mitotic catastrophe have not yet been modeled. To do this we incorporated *Caspase 2* activation during prolonged or perturbed metaphase [53,72]. Literature indicates that normal mitotic progression is a balancing act on the part of *Cyclin B*/*Cdk1* and *Plk1*. On one hand, both kinases phosphorylate and inhibit the anti-apoptotic *BCL2/BCL-X*_*L*_ proteins, priming cells for apoptosis [73–75]. On the other hand, *Cyclin B*/*Cdk1* inhibits *Caspase 2*, keeping cells alive as long as mitosis is not stalled [76]. In addition to the loss of *Cdk1* activity, metaphase cells also undergo *Caspase 2* mediated apoptosis in the absence of *Plk1* [77]. Our model captures this balance of pro- and anti-apoptotic signals, such that loss of *Cdk1* or *Plk1* activity before cells clear the spindle assembly checkpoint can push them into mitotic catastrophe.

### The network of linked regulatory models reproduces environment-dependent proliferation, quiescence, and/or apoptosis

Linked together, the modules generate a dense 87-node Boolean model with 375 links (**Fig 3B**). The synchronous dynamics of the full model is heavily constrained by the switch-like behavior of its modules, as evidenced by the small number of tightly coordinated behaviors (phenotypes) it generates. Indeed, when the state space of the network is sampled extensively using noisy synchronous update (*Methods & Model–Mapping the attractor landscape of large Boolean networks using synchronous update*), every attractor corresponds to a distinct cellular phenotype. These attractors are characterized in detail in **S2 Table**, along with key molecular signatures that allow us to match them to specific phenotypes. **Fig 3C** summarizes them according to the extracellular environment each phenotype occurs in; namely, the absence / low abundance / high abundance of growth factors (*x* axis on **Fig 3C**) combined with the presence / absence of the apoptotic signal *Trail* (*y* axis). **Table 1** matches cell phenotypes generated by our model to experimentally documented cell behaviors in multiple cell types. As expected, irreversible apoptosis is stable in every environment. Moreover, the ongoing presence of saturating *Trail* (i.e., *Trail* input is ON 100% of the time) destabilizes every other cell state, leaving apoptosis as the only stable option [79–82]. Similarly, the complete absence of growth / survival signals also leads to apoptosis [83–85]. In contrast, low levels of growth signaling support quiescent cell states, and our model identifies two distinct forms. First is a healthy cell state with 2N DNA content (**Fig 3C**, elongated cell with blue nucleus on). Second, our model also produces a G0-like state representing cells that have failed to complete mitosis or cytokinesis in the past, now stuck with a 4N DNA content (**Fig 3C**, elongated cell with yellow circle around nucleus). Finally, exposure to high levels of growth factor results in a cyclic attractor representing continuously cycling cells (**Fig 3C**, mitotic cell).

**Table 1:** Model attractors reproduce experimentally observed cell phenotypes.

Model behavior		Experimentally observed cell behavior	Cell type	Reference
***Trail-mediated apoptosis***				
	• Deterministic stimulation with *Trail* = ON kills quiescent cells in 3 time-steps; cycling cells in 2 to 21 steps (**S3 Fig** & **S1 File**). • Non-saturating *Trail* stimulation leads to fractional killing in cycling (**Fig 5B**) and quiescent cells. • Cells in metaphase are more sensitive to *Trail*-mediated apoptosis than at any other point during the cell cycle (**S3A Fig**).	Saturating concentration of *Trail* can kill ~100% of cells.	JURKAT (immortalized human T lymphocytes) *~100% cell death at* ≃ *25 ng/mL @ 24h*	Fig 2 in [79].
			7 human pancreatic cancer cell lines *~100% cell death at* ≃ *100 ng/m @ 24h*	Fig 1 in [80].
			MCF10A (immortalized, non-transformed human mammary epithelial cells) *~100% cell death at* ≃ *250 ng/mL @ 6h*	Fig 1D in [81].
		*Trail* is strongly synergistic with microtubule-targeting chemotherapy agents that trap cells in metaphase and delay SAC passage; even a low dose of *Trail* can kill them.	T98G (human glioblastoma cell line) HCT116 (human colon cancer cell line)	Fig 1 in [82].
***Growth factor withdrawal-mediated apoptosis***				
	• Deterministic stimulation with *GF* = OFF (*GF*_*High*_ = OFF) kills quiescent cells in 4 time-steps (**S1 File**). • Incomplete *GF* removal results in fractional killing of quiescent cells (**Fig 5B**, bottom right).	Murine hematopoietic stem cells fully deprived of extracellular IL3 (a survival signal they depend on), exhibit 70–90% death in 36 hours.	FL5.12 (murine Hematopoietic stem cell line) 32Dcl3 (murine myeloid cell line)	Fig 1B-C in [83].
		HC11 cells in serum-free medium undergo apoptosis (~45% of cells die within four days), while *EGF* or Fetal bovine serum protects them.	HC11 cells (mammary epithelial cells)	Fig 1B in [84].
		Cortical mouse astrocytes lacking the *EGF* receptor *EGFR* undergo apoptosis, and the resulting decrease of neurotrophic growth factors they secrete leads to neuronal loss.	cortical mouse astrocytes and neurons	Fig 1D, 2G, 4 in [85].
***Quiescence in low growth factor environments***				
	• Deterministic stimulation with *GF* = ON (*GF*_*High*_ = OFF) leads to quiescent cells (**Fig 4**, left). • Increasing amounts of mitogenic stimulation (*GF*_*High*_), above the levels required for survival, lead to stochastic cell cycle entry of a fraction of cells (**Fig 5B**, bottom left).	An increasing fraction of rat embryonic fibroblasts entered the cell cycle at increasing serum concentrations from 0.02% to 3%.	rat embryonic fibroblasts	Fig 2–4 in [86].
		An increasing fraction of MCF10A cells enter the cell cycle at increasing *EGF*/serum concentrations from 0.05% to 1.25%, reaching ~100% at 20ng/mL EGF / 5% horse serum.	MCF10A (immortalized, non-transformed human mammary epithelial cells)	Fig 1–2 in [87].
		Mouse fetal fibroblasts display wide heterogeneity in the timing of the G1 → S regardless of the level or duration of *IGF-I*, *EGF*, *PDGF-AA*, or *PDGF-BB* treatment.	C3H10T1/2 mouse fetal fibroblasts	Fig 6 in [88].
***Cell cycle progression under strong mitogenic stimulation***				
	• Deterministic stimulation with *GF*_*High*_ = ON leads to continually cycling cells (**Fig 4**).	At 5% bovine growth serum stimulation, ~100% of rat embryonic fibroblasts enter the cell cycle.	rat embryonic fibroblasts	Fig 2–4 in [86].
		At 20ng/mL EGF in 5% horse serum stimulation, ~100% of MCF10As enter the cell cycle.	MCF10A (immortalized, non-transformed human mammary epithelial cells)	Fig 1–2 in [87].

**Fig 4:**
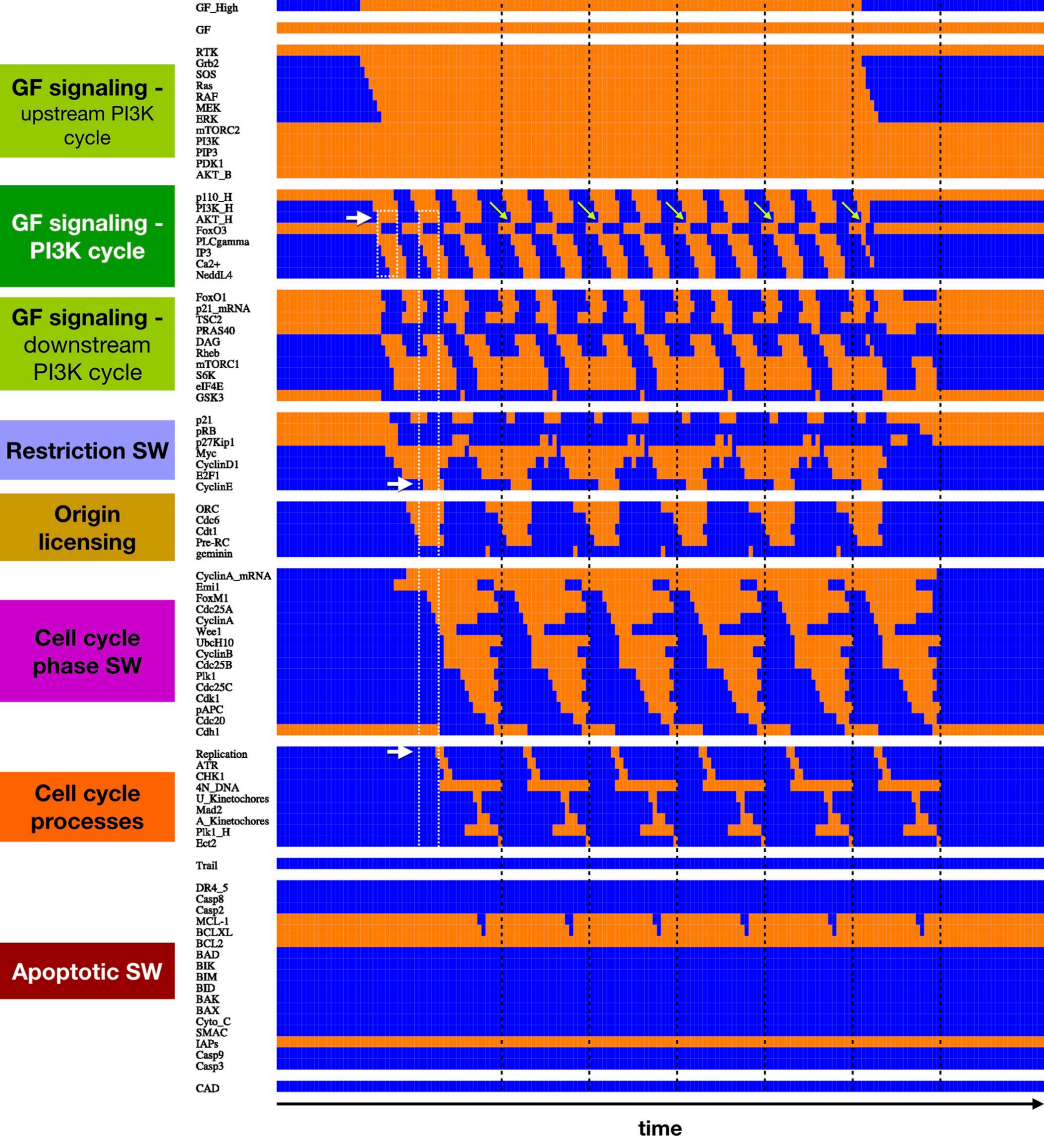
Module-level switches toggle each other to generate the cell cycle, locking *PI3K* oscillations to the rhythm of division. Dynamics of regulatory molecule expression / activity during cell cycle entry from G0, showcasing the phase-locking of *PI3K* oscillations to the cell cycle. *X*-*axis*: time-steps; *y*-axis: nodes of the model organized in modules; *orange/blue*: ON/OFF; *white boxes & arrows*: first two peaks of *AKT1* activation with respect to DNA replication; *black dashed lines*: cytokinesis; *lime arrows*: first *AKT1*-high pulse in each division cycle.

Our modular approach allows us to attribute discrete transitions cells undergo to the dynamics of isolated regulatory switches, apparent in the activation patter of the interlinked modules under synchronous update. For example, the sequence of molecular changes that occur within our modules transitioning from a quiescent state into the cell cycle reveals the higher-order logic by which regulatory switches toggle each other (**Fig 4**). First, cell cycle entry involves the activation of the *Growth Signaling Module*. While the basally active parts of this module remain on, we see a cascade leading to MAPK signaling (**Fig 4**, upstream *PI3K* cycle). This part of the module remains stably ON in a high (saturating) growth factor environment. In contrast, the part of the module responsible for cyclic *PI3K* / *AKT1* activation enters an oscillating pattern, as expected from the limit cycle on **Fig 2C.** Thus, our integrated model of growth signaling and cell cycle progression can reproduce the experimentally documented but unexplained oscillations in *PI3K* expression and *AKT1* activity [46]. Next, cyclic *AKT1* activity triggers downstream oscillations in *mTORC1* signaling and *GSK3β*. As these *AKT1* targets are subject to feedback from the rest of the network, they do not directly mimic the dynamics of *PI3K* and *AKT1* (see *Methods & Model*). Full activation of the *Growth Signaling* module then toggles the *Restriction Switch* into a state representing restriction point passage (later we observe this switch partially, but not fully reset between each cycle). Around the same time, we observe licensing of replication origins (*Origin Licensing Switch*), subsequently reset by the firing of replication origins in S-phase. Now committed, the cell toggles through replication, G2, mitosis and cytokinesis under the control of the *Phase Switch* (see *Cell cycle processes*). In contrast, the *Apoptotic Switch* only experiences minor perturbations, without being flipped.

### Our model’s dynamics is robust to fluctuations in signal propagation and reproduces the cell cycle with synchronous and asynchronous update

To test whether the orderly progression through the cell cycle is robust to random fluctuations in signal arrival time as they propagate through the network, we tested the model’s behavior under random order asynchronous update (*Methods & Model–Boolean Modeling Framework*) [86]. As fixed-point attractors of a Boolean model remain the same regardless of update [87], we focused on the cell cycle. As **S1 Fig** shows, a fully random update order does not abolish the model’s capacity to execute a correct cell cycle sequence, but it does introduce several non-biological behaviors. First, the signals that couple successful DNA replication to the establishment of a G2 state are lost under a subset of update orders, leading to G2 → G1 reset followed by a new cell cycle (endoreduplication). Second, the signals that drive cytokinesis can also be disrupted by certain update orders. Third, the balance of pro- and anti-apoptotic signals during metaphase can tip in favor of apoptosis, as if the cell experienced mitotic catastrophe. Interestingly, all three cell cycle errors are observed *in vitro* in cells experiencing knockdown or overexpression of a variety of cell cycle regulators [16,54,88]. Thus, we conclude that the asynchronous model with random update order mimics the occasional short-term loss of regulators, rather than the robust cycling of healthy cells.

**Fig 5:**
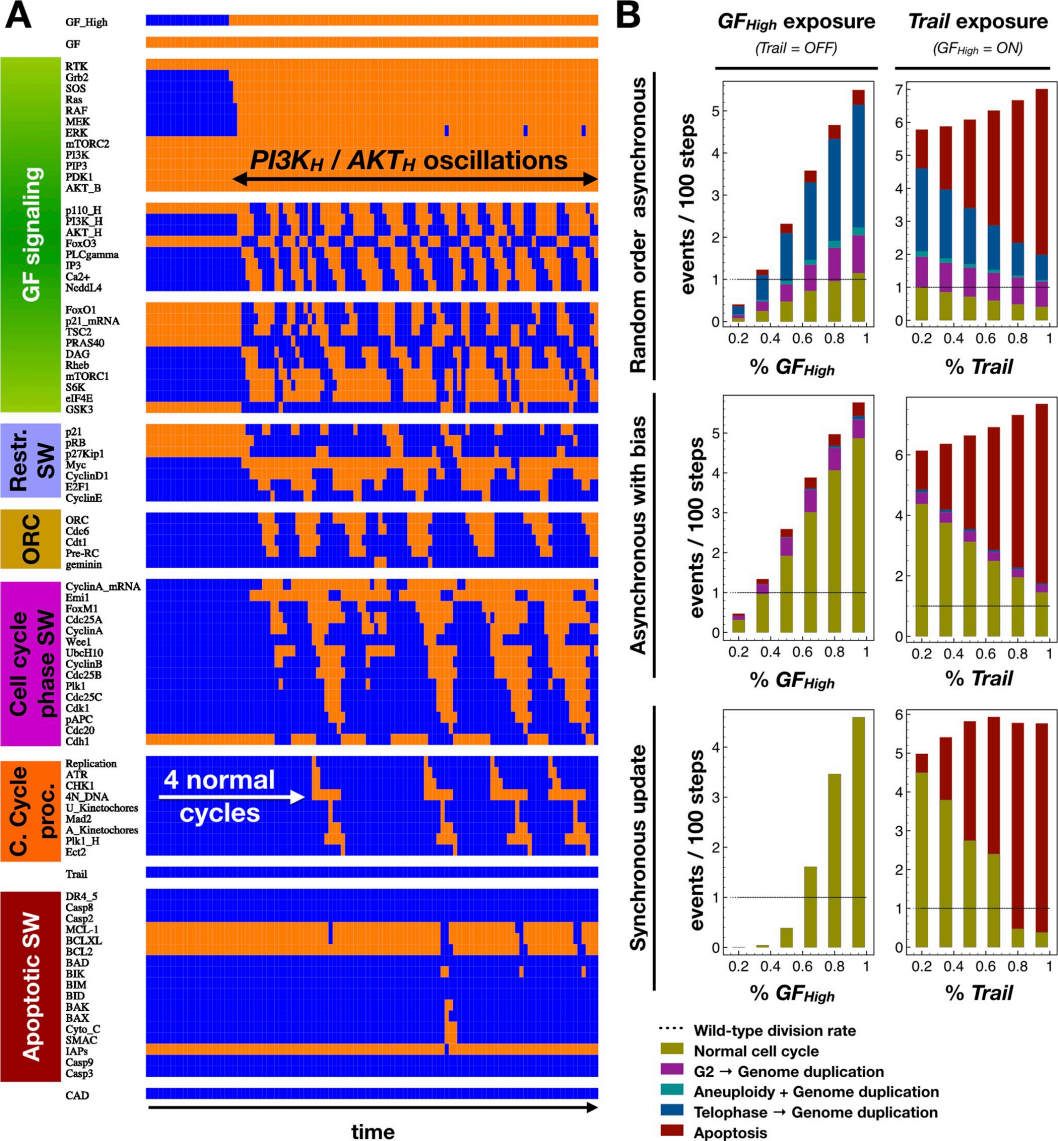
The cycling regulatory cascades of cell division are robust under biased asynchronous update. (A) Dynamics of regulatory molecule expression / activity during cell cycle entry from G0 using biased asynchronous update. *X*-*axis*: time-steps; *y*-axis: nodes organized in modules; *orange/blue*: ON/OFF. (B) Occurrence rate of normal cell cycle completion (*mustard*), G2 → G1 reset followed by genome duplication (*purple*), aberrant mitosis followed by genome duplication (*turquoise*), failed cytokinesis followed by genome duplication (*blue*) and apoptosis (*dark red*) per 100 timesteps, shown as stacked bar charts for increasing growth factor / *Trail* exposure (*left*/*right*) with random order asynchronous / biased asynchronous / synchronous update (*top*/*middle*/*bottom*).

In order to create a restricted random order that forbids asynchronous state transitions resulting from these non-physiological breaks in signal transduction, we identified genes and processes that deviated from their expected activity every time a particular error occurred and created an asynchronous version of the model with biased random update (*Methods & Model–Boolean Modeling Framework)*. To do this, we placed a small subset of nodes at the start or end of each update order, depending on their activation status (11 nodes; list and rationale in **S3 Table**). Using this biased update our model repeatedly and correctly executes the cell cycle, in spite of the asynchronous update (**Fig 5**). Our update bias did not completely eliminate endoreduplication from G2 and apoptosis (**S2 Fig**), but the incidence of these errors decreased drastically. As these errors do occasionally occur in wild-type cells [16,82], we choose not to further restrict our update order to eliminate them. Rather, we measured the rate at which the two update schemes produce normal cell cycle events vs. different errors via a series of simulations at varying levels of growth factor and *Trail* stimulation. We did this by setting *GF*_*H*_ or *Trail* ON with probability *p* in each time-step, OFF otherwise. As **Fig 5B** indicates, the asynchronous model with biased update shows a similar response to growth factors and *Trail* as the synchronous model. Moreover, the incidence of apoptosis or endoreduplication after G2 is significantly lower than under random update, and lack of cytokinesis all but disappears.

The apoptotic fixed-point is reachable from cell cycle under both random-order and biased asynchronous update, indicating that the cell cycle is not, strictly speaking, a complex attractor [89]. Nevertheless, starting an asynchronous time series from *any* state along the synchronous cell cycle attractor results in long time-courses featuring repeated (if occasionally incorrect) cycles (**S4 Fig**). This indicates that the system’s state space has a metastable region that traps its dynamics in a way that resembles a complex attractor. To test whether this metastable collection of states is also a *cycle*, we sampled the state transition graph of the asynchronous model with both update schemes by starting 10 independent time courses of 1000 steps from each state along the synchronous cell cycle. In order to sample the metastable basin rather than the path to the apoptotic attractor, we prematurely interrupted each run if it reached a fixed point. We then overlayed all observed states and transitions, visualizing the largest strongly connected component (**S5 Fig**, left). To test whether these state transition graphs are consistent with robust execution of the cell cycle, we classified each state as representing G1, S, G2, metaphase, anaphase, telophase and cytokinesis depending on the ON/OFF state of key processes (**S6 Fig**). Instead of a cycle, however, the resulting network revealed distinct regions of state-space representing G1, S and G2, then a few highly restricted and often-visited paths through anaphase and cytokinesis. Thus, asynchronous update indicates that there may be widespread molecular heterogeneity in G1, S and G2, but most of the network we model locks into a few unique states during anaphase.

It is worth noting that our model features *two* internal oscillators, the core cell cycle and the *PI3K* degradation / re-synthesis cycle. As **Fig 5** and **S1 Fig** indicate, these two cycles are not completely phase-locked under asynchronous update. As the cell cycle proceeds, the small *PI3K* oscillator and the downstream *mTORC1* pathway can be found in nearly any state. The sole exception is anaphase, where the two cycles appear to sync up. To show that the heterogeneity is chiefly within the growth pathway, we projected the state transition graph of each asynchronous model onto a subspace where each network state represents a unique ON/OFF state within the *core* cell cycle modules (*Restriction SW*, *Origin of Replicaton SW*, *Phase SW* and *Cell cycle processes*), regardless of the state of all other nodes. This process collapsed the complex state transition graph of the biased model onto a clear cyclic flow of transitions, representing normal cell cycle progression (**S5 Fig**, bottom right). In contrast, the random asynchronous model’s dynamics has a loop corresponding to the cell cycle, but it is dominated by prominent “backward” transitions representing endoreduplication from G2 (**S5 Fig**, top right).

To further test our model against published experimental data, we compared its least intuitive dynamical behaviors to experimental observations (**Table 2**) and described them in detail in **S1A–S1D Text**. To summarize, both our synchronous and biased asynchronous model reproduces the cyclic degradation and re-synthesis of *p110*, leading to oscillating *AKT1* signaling (**Figs 4** and **5**). In cells entering the cell cycle from quiescence, this oscillatory behavior generates two distinct phospho-*AKT1* peaks before cells complete DNA synthesis (**Fig 4, S7 Fig**). Furthermore, cells that lack high *p110* protein expression fail to enter the cell cycle in response to growth factors (**S8 Fig**). Our models also reproduce the bifurcation of fates in cells cycling in non-saturating growth environments. Namely, a large fraction of cells were shown to pass the restriction point *before* cytokinesis (in late G2/M of the previous cycle), while the remainder reset into a G0-like state and commits to the next cycle again after a highly variable time-window (**S9** and **S10 Figs**) [90]. Finally, a comparison of 34 model knockout and 11 overexpression phenotypes to experimentally manipulated cell behaviors indicate that our model can faithfully reproduce in vitro cell behavior under a wide range of genetic manipulations (**S4 Table**).

**Table 2:** Model reproduces experimentally documented dynamical behaviors.

Model behavior			Experimentally observed cell behavior	Cell type	Ref.
Synchronous update		Biased asynchronous update			
**Dynamics of the *PI3K* / *AKT1* signaling pathway **					
	• Continuously cycling cells (*GF* = ON & *GF*_*High*_ = ON) show an oscillating pattern of *PI3K*_*H*_ and *AKT*_*H*_ activation driven by *p110*_*H*_ degradation and re-synthesis (**Fig 4**).	• In spite of irregular G1 gaps between consecutive rounds of division in continuously cycling cells (*GF* = ON & *GF*_*High*_ = ON), the *p110*_*H*_ / *PI3K*_*H*_ / *AKT1*_*H*_ network displays a robust oscillatory pattern (**Figs 5, S1** and **S2**).	The catalytic submit of *PI3K*, *p110*, was shown to undergo rapid degradation in response to growth factors, followed by slow re-synthesis.	MCF10A (human mammary epithelial cells)	Figs 2–3 in [46].
				BEAS-2B (human bronchial epithelium) H157 (Human oral squamous cell carcinoma cell line)	Fig 6 in [47].
	• Cells entering the cell cycle from quiescence display two *AKT*_*H*_ activation peaks in G1 and at the start of S phase (**Fig 4**, white boxes).	• In spite of variable G1 length in cells entering the cell cycle from quiescence, *PI3K*_*H*_ and *AKT*_*H*_ activity averaged over 1000 cells (runs) show two distinguishable peaks as a function of time, one on G1 and one that roughly coincides with S-phase (**S7 Fig**).	Western blot for *pAKT1* activity cells synchronously entering the cell cycle shows two distinct peaks of activity. The second peak occurs when about 50% of cells have entered S-phase.	NIH3T3 (primary mouse embryonic fibroblast cells, spontaneously immortalized)	Fig 1D in [29].
**High *p110* expression requirement for the initiation of proliferation**					
	• Cells cannot enter the cell cycle in the absence of high *p110* protein expression (**S8A Fig**). • Increasing levels of *p110*_*H*_ knockdown slow proliferation by lengthening the time cells spend in G1 (**S8B Fig**).	• Increasing levels of *p110*_*H*_ knockdown slow proliferation (**S8C Fig**).	High *p110* protein expression is required for colony formation.	MCF10A (human mammary epithelial cells).	Fig 5 in [46].
**Restriction point passage in growth-stimulated quiescent vs. continuously cycling cells**					
	• Cells entering the cell cycle from quiescence pass the restriction point in late G1, after which they commit to a full division cycle. This event is marked by full activation of *E2F1*, leading to *Cyclin E* expression and S-phase entry (**S9A Fig**).		Quiescent cells entering the cell cycle pass a point of no return called the restriction point marked by the full (and bimodal) activation of *E2F1*-driven transcriptional activity that locks in a bistable switch. Cells that pass this point commit to a full division cycle even in the absence of continued growth stimulation.	rat embryonic fibroblasts	Fig 2–4 in [86].
	• Under saturating mitogenic stimulation, cycling cells can commit to another division cycle before finishing their current one, and thus can execute a full cell cycle in the absence of mitogenic stimulation (**S9B Fig**).	• Under strong mitogenic stimulation the asynchronous simulation produces a wide variety of G1 intervals. In the shortest of these, the *Restriction Switch* is locked on by the end of metaphase in the previous cycle (**Fig 5A**, last G1 gap).	Rapidly dividing mammalian cells can pre-commit to a division cycle before finishing their current one. In this case, they execute a full cell cycle after mitogen withdrawal, such that their last exposure to mitogens occurs sometime during the previous G2 phase.	MCF10A (human mammary epithelial cells) HS68 (human foreskin cells) Swiss3T3 (primary human fibroblasts)	Fig 3 in [91].
	• Under non-saturating mitogenic stimulation, cycling cells stochastically toggle between pre-commitment and a G0-like pause of various lengths (**S1 File;** also see [11] for this behavior in the core cell cycle model).	• The state transition graph of cycling cells shows two distinct groups of G1 states; one along the continuous cell cycle (the path of pre-commitment), and one that clusters apart (the G0-like pause). Return from this G0-like cluster of states generally goes through the late-G1 state along the cycle (**S5B Fig**).	Not all cells in the population pre-commit to another cycle.		

### Attenuated *Plk1* expression in anaphase phenocopies the cell cycle defects of *PI3K* and *AKT1* overexpression

Experimental data indicates that hyperactive *PI3K* and/or *AKT1* in G2 leads to an enrichment of binucleated cells stuck in telophase [29,40]. Studies that document these errors point to the loss of *FoxO3* and/or *FoxO1* activity in G2 (a consequence of hyperactive *AKT1*), two transcription factors that positively regulate the expression of mitotic *cyclin B*, as well as polo-like kinase 1 (*Plk1*). *Cyclin B* accumulation is only required for metaphase entry (a process that appears normal in cells with hyperactive *PI3K/AKT1*); its activity is not required for cytokinesis. *Plk1*, in contrast, plays distinct roles at every phase of mitosis and cytokinesis [54]. Thus, we hypothesized that telophase enrichment in cells with hyperactive *PI3K/AKT1* may be due to compromised *Plk1* expression in G2 or early mitosis [29,40], and that partial knockdown of *Plk1* in our model phenocopies this error.

To test this, our previously published *Phase Switch* [11] required a revision to incorporate the complex regulatory role of *Plk1* (**Fig 3A**). Experimental evidence indicates that *Plk1* is upregulated in G2 by the *FoxM1* transcription factor (also newly added). While the combinatorial regulation of *Plk1* by *FoxM1*, *FoxO3* and *FoxO1* has not been investigated, experiments clearly show that *Plk1* remains active until late telophase [60,61]. That said, *Plk1* protein level drop dramatically in anaphase due to proteasomal degradation by *APC/C^Cdh1^* [60,61]. It is the availability of the remaining *Plk1* pool, responsible for the assembly of a contractile ring, that appears compromised in the absence of *FoxO* activity in G2 [29]. To capture this within a Boolean framework, we accounted for the role of *FoxO* factors in creating an *increased* pool of *Plk1* by introducing two Boolean nodes to track *Plk1* activity (**S11 Fig, S1E Text**). Thus, the *Plk1* node represents the active enzyme required for mitotic entry, normal mitotic progression and anaphase completion. In contrast, *Plk1*_H_ = ON represents the short-lived accumulation of a large enough *Plk1* pool to survive *APC/C^Cdh1^* mediated degradation past anaphase, and aid the assembly of a contractile ring by recruiting the *RhoA* GEF protein *Ect2* [60].

**Fig 6:**
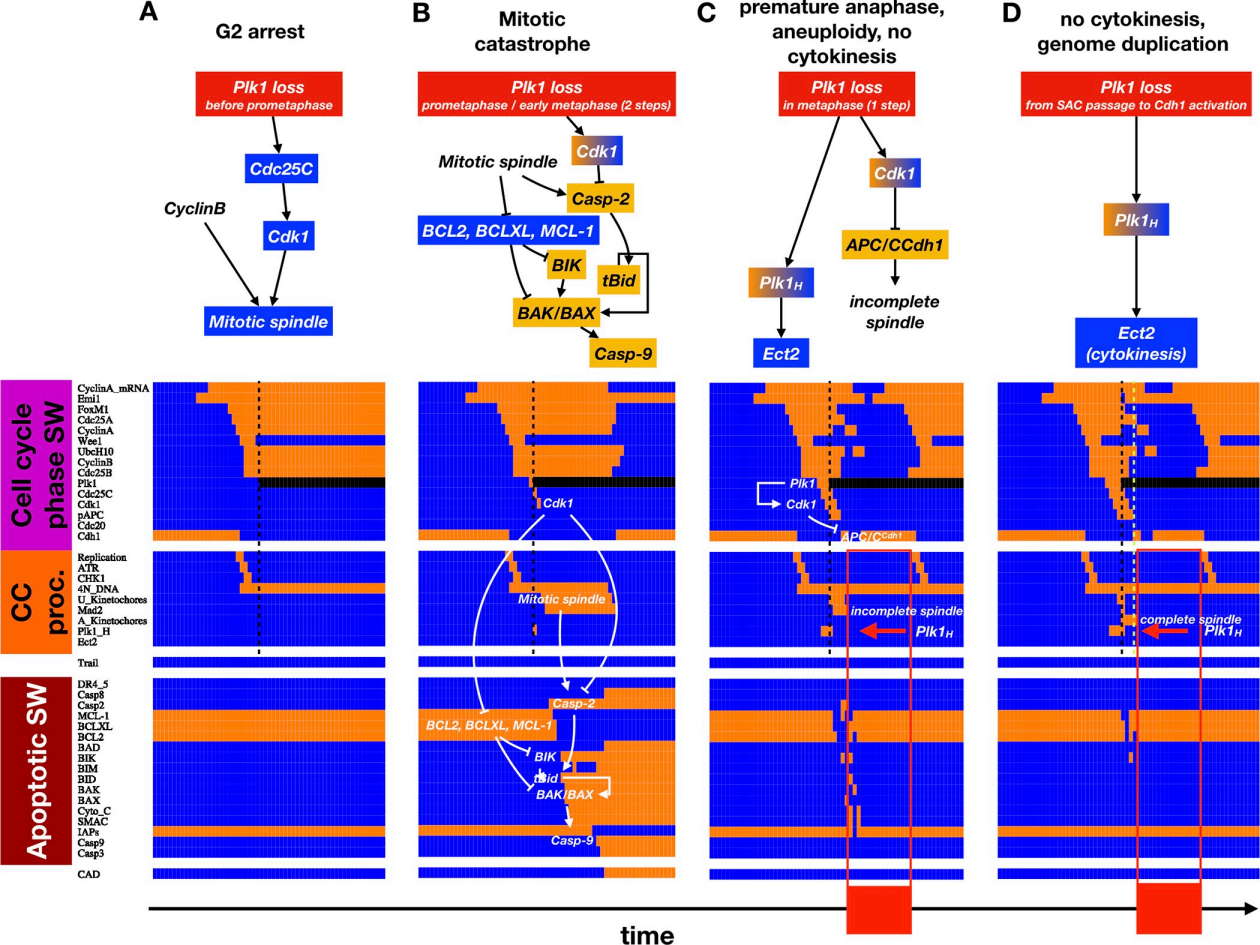
*Plk1* inhibition at different points along the cell cycle can cause G2 arrest, mitotic catastrophe, aberrant mitosis, or failure of cytokinesis. (A-D) *Top*: (A) Molecular mechanism leading to G2 arrest via *Plk1* knockdown before the start of prometaphase due to a lack of *Cdk1* activation; (B) mitotic catastrophe and apoptosis via *Plk1* knockdown in prometaphase or early metaphase due to concurrent *Casp2* activation and deactivation of the antiapoptotic *BCL2* family; (C) aberrant (premature) anaphase and no cytokinesis via *Plk1* knockdown later in metaphase due to premature *APC/C*^*Cdh1*^ activation, and (D) normal anaphase but no cytokinesis via *Plk1* knockdown post SAC passage due to loss of *Plk1*_*H*_ in telophase. *Orange/blue background*: higher/lower than normal activity; *gradient background*: premature node transition*; no background*: other relevant node / process; →: activation; ⊣: inhibition. *Bottom*: Dynamics of expression / activity of *Phase Switch*, *Cell cycle processes* and *Apoptotic Switch* nodes in cells exposed to *Plk1* inhibition at different stages of the cell cycle. *X*-*axis*: time-steps; *y*-axis: nodes of the model organized in modules; *orange*: ON (expressed and/or active); *blue*: OFF (not expressed and/or inactive); *black*: OFF, forcibly inhibited. *Black dashed line*: timing of *Plk1* inhibition; *white pathways*: processes that initiate apoptosis (B), premature anaphase (C), or failed cytokinesis (D); *red box & bar*: telophase/G1 in the absence of cytokinesis, followed the next round of DNA synthesis; *lime green line*: point of normal *APC/C*^*Cdh1*^ activation, marking the end of the Plk1 inhibition window that can compromise cytokinesis (D); only relevant module activity is shown (full dynamics available in **S1 File**).

Next, we tested whether our model can accurately account for all known roles of *Plk1* during cell cycle progression. To this end, we first modeled the inhibition of *Plk1* at different points along the cell cycle using synchronous update [54]. As **Fig 6** shows, *Plk1* inhibition in our model reproduces four distinct, experimentally documented phenotypic outcomes, depending on the precise timing of *Plk1* inhibition during the cell cycle (**Table 3**). First, loss of *Plk1* before prometaphase (i.e., before robust *Cdc25C* & *Cdk1* activation) results in G2 arrest (**Fig 6A**). Second, complete of loss *Plk1* at the prometaphase /metaphase transition or early metaphase leads to prolonged arrest and mitotic catastrophe (**Fig 6B**). Third, our model predicts that *Plk1* loss in *late* metaphase can trigger permute anaphase rather than mitotic catastrophe, leading to chromosome mis-segregation and aneuploidy (**Fig 6C**). This occurs when *Plk1* and *CyclinB* / *Cdk1* are both available to phosphorylate the *APC/C* subunit of the Anaphase Promoting Complex [92], leading to *Cyclin A* degradation [93]. The loss of this key *APC/C*^*Cdh1*^ inhibitor, together with the subsequent loss of *Cdk1* activity in the absence of *Plk1*, results in premature *APC/C*^*Cdh1*^ activation. *APC/C*^*Cdh1*^ disassembles the incomplete mitotic spindle, allowing a narrow escape from apoptosis and instead leading to chromosome mis-segregation and premature telophase. Lack of *Plk1* past this point guarantees that cytokinesis does not follow. Making matters worse, our model shows that continued growth factor signaling can lead to a new round DNA synthesis (**Fig 6C**). Fourth, *Plk1* inhibition a time-step later leads to normal anaphase mediated by *APC/C*^*Cdc20*^ (**Fig 6D**). As long as *Plk1* inhibition starts before *APC/C*^*Cdh1*^ activation, however, cytokinesis still fails (**Fig 6D**, lime green line).

**Table 3:** Model reproduces the experimentally documented effects of *Plk1* knockdown and *p110/PI3K/AKT1* over-expression / hyperactivity.

	Model behavior			Experimental evidence
		Synchronous update	Asynchronous update	
**Timing and/or strength of *Plk1* inhibition determines the resulting cell cycle error**				
	***Complete absence of Plk1 leads to G2 arrest*.**	Loss of *Plk1* before prometaphase results in G2 arrest (**Fig 6A**).	Strong *Plk1* knockdown reduces the overall rate of cell cycle entry (**Fig 7**).	*Plk1* activation by *Cyclin A/Cdk2* at the G2/M boundary is required for mitotic entry [55].
		Very strong *Plk1* inhibition reduces the overall rate of cell cycle entry (**Fig 7**).		
	***Strong Plk1 knockdown leads to mitotic arrest*, *mitotic catastrophe and apoptosis*.**	Loss *Plk1* at the prometaphase /metaphase transition or early metaphase leads to prolonged arrest and mitotic catastrophe (**Fig 6B**).		*Plk1* is required for the formation and maintenance of microtubule-kinetochore attachments [98,99].
		Strong *Plk1* knockdown increases the likelihood of apoptosis following mitotic catastrophe (**Fig 7**).		
	***Moderate Plk1 knockdown can cause premature*, *aberrant anaphase*, *aneuploidy and genome duplication*.**	*Plk1* loss in *late* metaphase triggers permute anaphase rather than mitotic catastrophe, leading to chromosome mis-segregation, aneuploidy and genome duplication (**Fig 6C**).		Aneuploidy and genome duplication have been documented in *Plk1*-inhibited cells [59].
		Moderate *Plk1* knockdown increases the likelihood of premature anaphase (leading to aneuploidy), followed by genome duplication (**Fig 7**).		
	***Weak Plk1 knockdown enriches for cells that fail to undergo cytokinesis*, *leading to genome duplication*.**	*Plk1* loss in anaphase / telophase leads to failure of cytokinesis and genome duplication (**Fig 6D**).		Due to its role in driving contractile ring assembly (by recruiting the RhoGEF *Ect2* to the central spindle), *Plk1* knockdown in telophase blocks cytokinesis [62].
		Weak *Plk1* knockdown increases the likelihood of failed cytokinesis and genome duplication, despite normal mitotic progression (**Fig 7**).		
**Forced expression of *p110***_***H***_**, hyperactive *PI3K***_***H***_ **or *AKT1*, and loss of *FoxO3*/*FoxO1* result in failure of cytokinesis**				
	***Forced expression of p110***_***H***_ ***result in failure of cytokinesis***	Forced expression of *p110*_*H*_ result in failure of cytokinesis in cells exposed to high levels of mitogenic stimulation (**Fig 8A**)		Expression of the constitutively active *p110* mutant *p110CAAX* lead to the enrichment of binucleated telophase cells (Fig 1C in [29]).
		Strong forced expression of *p110*_*H*_ increases the likelihood of cells that fail to undergo cytokinesis (**Fig 8B** and **8C**)		
	***Forced expression of PI3K***_***H***_ ***result in failure of cytokinesis***	Strong forced expression of *PI3K*_*H*_ increases the likelihood of cells that fail to undergo cytokinesis (**Fig 8B** and **8C**)		Genome doubling has been documented in a transgenic mouse model harboring overactive *PI3K* [95].
	***Forced expression of PI3K***_***H***_ ***and AKT***_***H***_ ***increases the rate of proliferation in low growth factor environments (Fig 8B and 8C).***			Both *PI3K* and *AKT* are powerful proto-oncogenes; their hyperactivity is linked to excess proliferation and tumor formation [32].
	***Partial FoxO3 knockdown enriches for cells that fail to undergo cytokinesis*.**	Knockdown of *FoxO3* increases the fraction of cells that do not complete cytokinesis (**S13 Fig**).		Cells expressing transcriptionally inactive *FoxO3* accumulate in telophase and do not move to G1 at the same rate as control cells (Fig 3 in [29]).
	***Strong FoxO3 knockdown leads to a significant decrease in PI3K / AKT1 signaling and slows proliferation (S13 Fig).***			*FoxO3* and *FoxO1* inhibition leads to down-regulation of *AKT* signaling, lengthens the cell cycle, and weakens colony forming potential in cancer cells [96].

**Fig 7:**
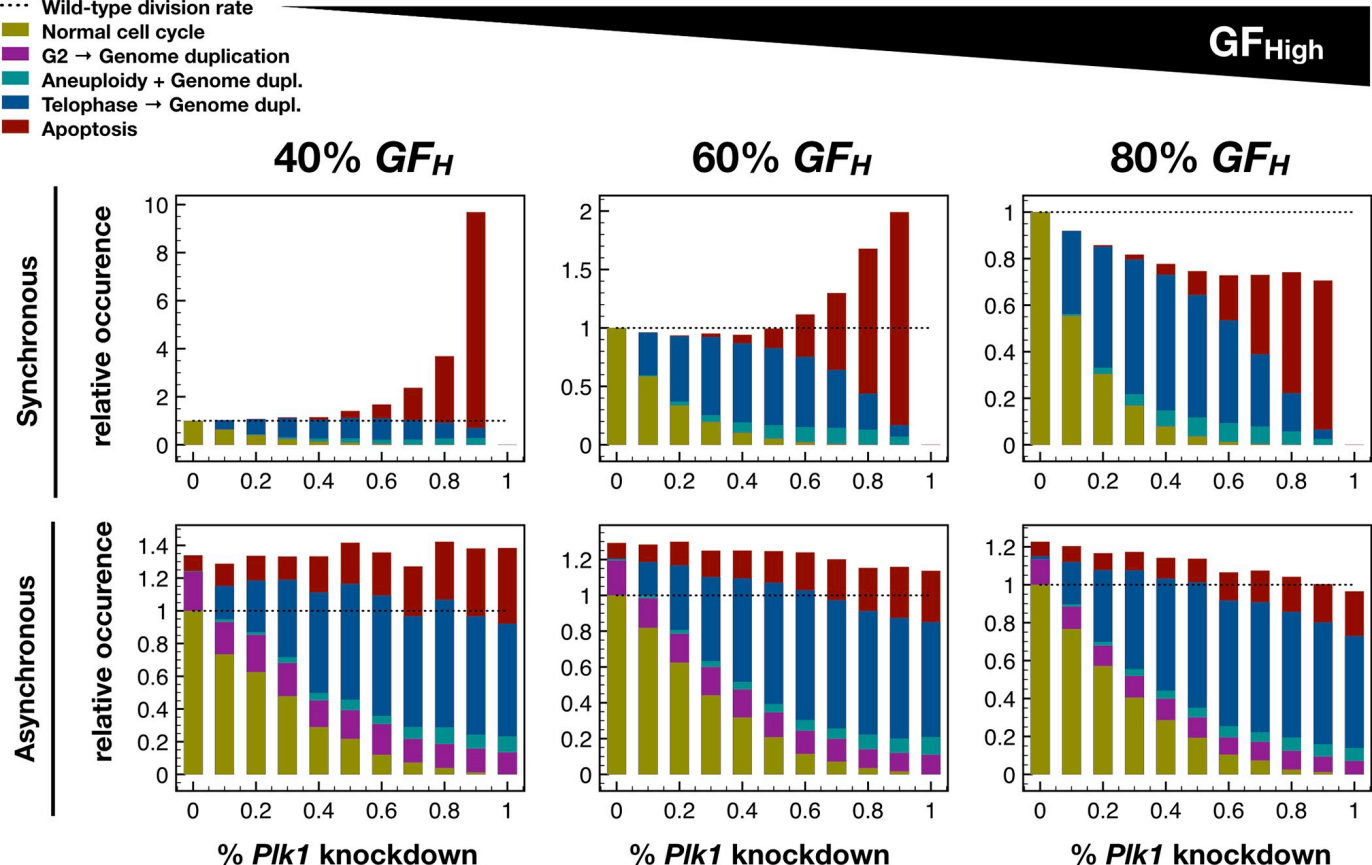
The strength of *Plk1* inhibition sets the relative prominence of cell cycle failure modes. Stacked bar charts showing the relative occurrence of normal cell cycle completion (*mustard*), G2 → G1 reset followed by genome duplication (*purple*), aberrant mitosis followed by genome duplication (*turquoise*), failed cytokinesis followed by genome duplication (*blue*) and apoptosis (*dark red*) relative to the rate of cell cycle in wild-type cells (*black dashed line*) at growth factor exposure of 40%, 60% and 80% in *Plk1*-deficient cells using synchronous (*top*) and biased asynchronous update (*bottom*).

Given that our model adeptly captures four distinct effects of *Plk1* inhibition, next we asked whether partial loss of *Plk1* can phenocopy the effects of hyperactive *PI3K/ AKT1*. We modeled partial knockdown of *Plk1* by running stochastic simulations in different growth conditions, where we forced the OFF state of *Plk1* in every time-step with a fixed probability and allowed the node to obey its normal regulation when not forced (**Fig 7**; *Methods & Model–Modeling non-saturating growth factor stimulation and partial knockdown / overexpression within a Boolean framework*). Our simulations indicate that the dominant failure mode in a population of cells depends on the strength of *Plk1* inhibition. When *Plk1* inhibition is very strong (but not complete), cells often start mitosis but do not complete it. This leads to increased mitotic length, often followed by apoptosis (**Figs 7** and **S12A**). In contrast, aberrant mitosis leading to aneuploidy is more common at moderate *Plk1* inhibition (peak at 60%), though apoptosis is still more likely (**Figs 7** and **S12A**). The most common cell fate at this point, however, is normal mitosis followed by prolonged telophase (**S12B Fig**) and failure to undergo cytokinesis. This remains the prominent failure mode at moderate-to-weak *Plk1* inhibition levels (peak at 30%; **Figs 7** and **S12A**). Performing the same series of *in silico* experiment using biased asynchronous update lead to qualitatively similar results (**Fig 7**), with the caveat that the asynchronous model occasionally skip mitosis altogether–an effect that does not change with *Plk1* inhibition. In summary, *weak Plk1* inhibition in our model phenocopies the experimentally documented effects of hyperactive *PI3K* and/or *AKT1*–in line with the hypothesis that the cause of weakened *Plk1* expression is lack of some (i.e., *FoxO3* and *FoxO1*) but not all transcriptional *Plk1* activators in G2 (*FoxM1* remains active).

### Cyclic degradation of *PI3K* is required for normal cell cycle progression

**Fig 8:**
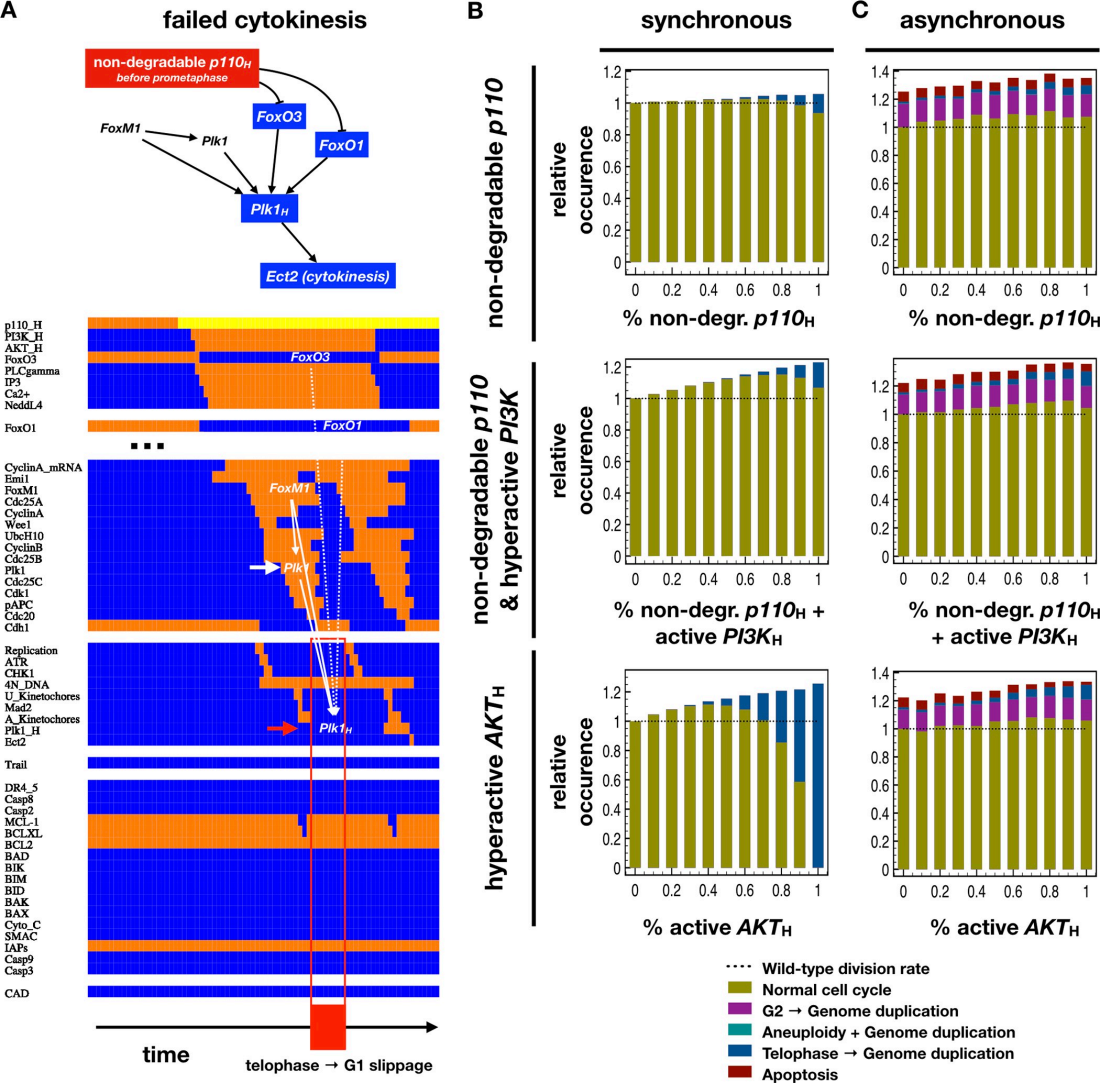
*p110* degradation in G2 is required for cytokinesis. (A) *Top*: Molecular mechanism leading to the failure of cytokinesis in the presence of non-degradable *p110*_*H*_. *Blue background*: lower than normal activity; *no background*: other relevant node / process; →: activation; ⊣: inhibition. *Bottom*: Dynamics of regulatory molecule activity during the transition from cell cycle to telophase, then genome duplication upon expression of non-degradable *p110* (*yellow*). *X*-*axis*: time-steps; *y*-axis: nodes of the model organized in modules; *orange/blue*: ON/OFF; *yellow*: ON, forcibly expressed; *white arrows & nodes*: factors driving *Plk1* expression and lack of *Plk1*_*H*_ accumulation; *red arrows & box*: failure of cytokinesis followed by G1 in bi-nucleated cells; only relevant module activity is shown (full dynamics available in **S1 File**). (B-C) Relative occurrence of normal cell cycle completion (*mustard*) vs. genome duplication following failed cytokinesis (*blue*) relative to the rate of cell cycle in wild-type cells (stacked bar charts) at 80% growth factor exposure in cells with non-degradable *p110*_*H*_ (*top*), non-degradable *p110*_*H*_ + active *PI3K*_*H*_ (*middle*) and hyperactive *AKT*_*H*_ (*bottom*). Modeled using synchronous (B) and biased asynchronous update (C).

To test whether our model accurately links non-degradable *p110* to altered *Plk1* expression leading to failure cytokinesis, we ran *in silico* experiments in which we kept the *p110*_H_ node forcibly ON, starting at different points along the cell cycle (**Fig 8**). As expected, expression of a non-degradable *p110* leads to high sustained *PI3K* and *AKT1* activity. Loss of *FoxO3*/*FoxO1* during G2 and M prevents *Plk1* levels from accumulating enough to outlive *APC/C*^*Cdh1*^-mediated depletion (*Plk1*_*H*_ does not turn on; **Fig 8A**). The result is failure to undergo cytokinesis (**Fig 8A**, red box), matching the experimentally documented enrichment of telophase cells in the presence of overactive *PI3K*, *AKT1*, or inactive *FoxO3* [29]. In addition to telophase enrichment, our model shows genome reduplication in the resulting bi-nucleated cells, also supported by experimental evidence (**Table 3**). Intriguingly, our model predicts that the loss of high growth factors during G2 or M allows these cells to compete cytokinesis (**Fig 8A,** second cycle). This occurs because high *p110* protein expression alone is not sufficient for high *AKT1* activation; it also requires ongoing growth signaling and active *Ras* [91]. Thus, loss of strong growth stimulation allows the re-entry of *FoxO3* into the nucleus, leading to *Plk1* expression and cell cycle completion.

Synchronous update allows us to track the molecular consequences of locking *p110* into a high-expression state, but it has several drawbacks. Most importantly, it assumes the presence of a saturating growth factor environment and 100% *FoxO* inhibition downstream of *PI3K*/*AKT1*. To test whether our results hold in the presence of intrinsic or extrinsic fluctuations such as moderate growth factor availability and incomplete hyperactivation of *p110*_H_, we tracked the fate of cells in a variety of non-saturating growth factor environments. In each environment, we tested the effect of *incomplete p110*_H_, *p110*_H_ + *PI3K*_H_, or *AKT1*_*H*_ over-expression by stochastically forcing the ON state of these nodes with a fixed probability (**Figs 8B** and **S13**). These results also point to a high prevalence of cells that cannot exit telophase in near-saturating growth environments (blue bars on **Fig 8B**). As hyperactive *AKT1*_*H*_ in the model is forced ON regardless of growth signaling, it drives both an increase in proliferation and the failure to exit telophase even in low growth factor environments (**S13B Fig**).

As the mediators of cell cycle progression errors in hyperactive *PI3K*/*AKT1* are thought to be *FoxO* factors, we next showed that partial inhibition of *FoxO3* phenocopies hyperactive *PI3K* and/or *AKT1* in our synchronous model (**S13E Fig, Table 3**). That said, strong *FoxO3* inhibition also slows / stops re-synthesis of *p110*, leading to a lengthened cell cycle (documented in cancer cells and tumors *in vivo* [94]; **Table 3**). The subsequent weakening of *AKT1* signaling counterbalances the loss of *FoxO3*, weakening its effects. In line with this, our biased asynchronous update results do not show an increase in cytokinesis failure with *FoxO3* knockdown (**Figs 8C** and **S13E**).

In addition to reproducing the effects of hyperactive *PI3K/AKT1*, our model offers several experimentally testable predictions: *1)* We predict that the observed cycle of *p110* degradation and re-synthesis is driven by the network in **Fig 2A**. As a result, knockdown of *PLCγ*, *NEDDL4*, or the chelation of intracellular Ca^2+^ is expected to lead to sustained high *p110* protein expression *in vitro*. *2)* In addition to an increase in cell cycle length, we predict *PLCγ* knockdown to enrich for telophase cells that fail to complete cytokinesis. *3)* Continuously cycling cells execute at least two rounds of *PI3K* activation and destruction for each round of division (**Figs 4** and **5**). *4)* Loss of saturating growth signals in G2 allows *p110*-overexpressing to complete a normal cell cycle (**S10 Fig**). *5)* Once committed to a cell cycle, saturating growth stimulation allows cells to keep cycling even if *p110* levels drop later in the cycle, and pre-commitment in *p110*-inhibited cells is driven by mitotic *mTORC1* aiding the re-activation of *Myc* (**S10 Fig**).

## Discussion

In this study we developed a detailed modular Boolean model of the regulatory pathways driving growth factor signaling, cell cycle progression and apoptosis (**Fig 3B**). While there are several published models with a similar coverage of cellular behaviors [15,21,23,25,26], the focus of our study was to capture the dynamical behavior of the *PI3K* → *AKT1* signaling axis driving cell growth. To this end, we proposed a mechanism capable of driving the experimentally documented oscillations of *PI3K* protein expression [46,47], explored the importance of high and low *PI3K* activity during different phases of the cell cycle, and showed that our model can offer mechanistic insight into the cellular effects of hyperactive *PI3K* (failure of cytokinesis). To do this, we identified two negative feedback loops potentially responsible for driving *PI3K* dynamics. The first loop is triggered by high growth factor signaling and high *PI3K* activity, and it involves *PLCγ*-mediated activation of the *NEDDL4* ubiquitin ligase [51], known to target the *p110* subunit of *PI3K* for degradation [47]. The second loop involves the loss of *AKT1*-mediated *FoxO3* inhibition as *PI3K* activity drops, allowing *FoxO3* to drive the re-expression of *p110* [52]. As these two pathways were key to our model’s ability to reproduce the effects of hyperactive *PI3K* and *AKT1* on cell cycle progression, they represent its two most significant predictions.

**Fig 9:**
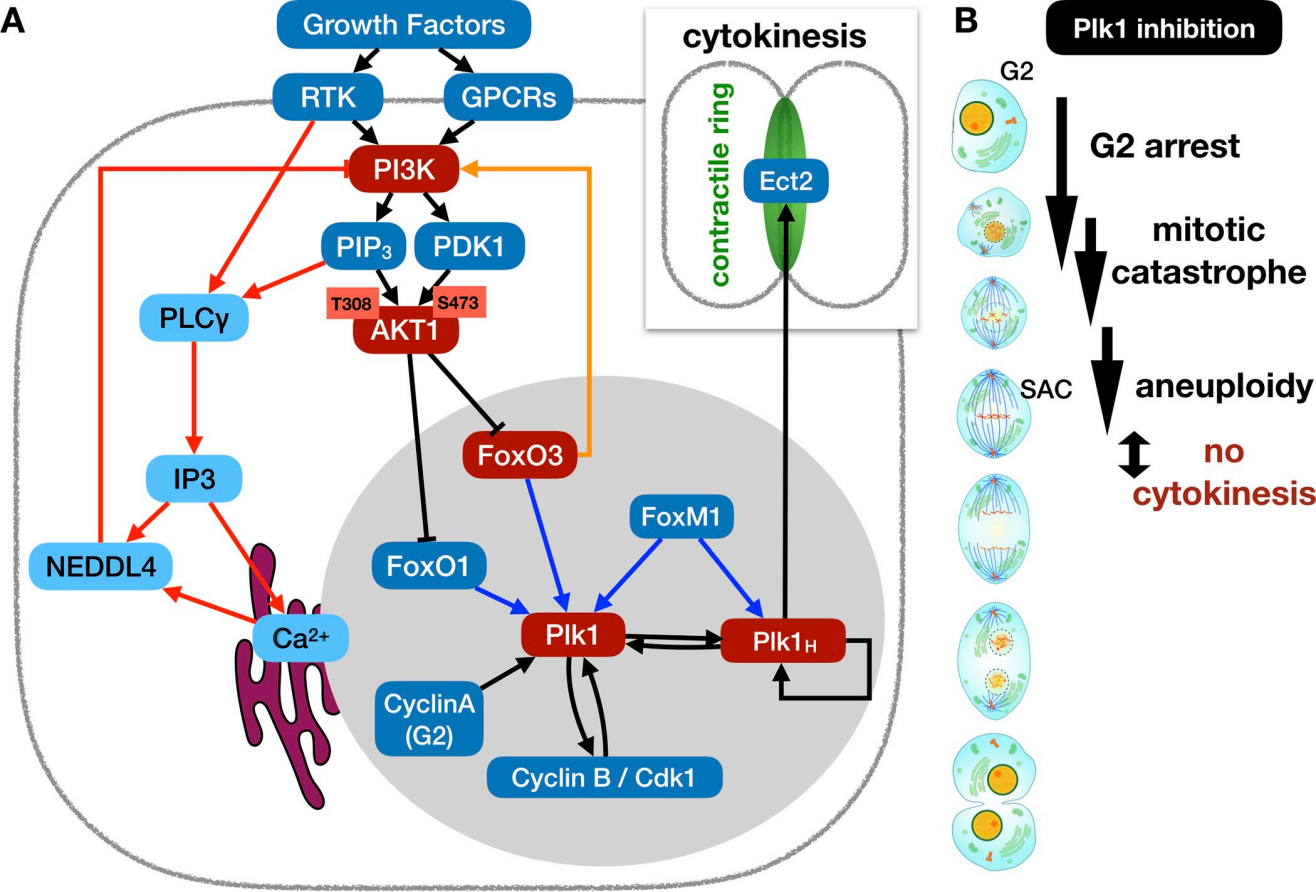
Summary of the molecular mechanisms that link *PI3K* hyperactivity to attenuated *Plk1* expression and failure of cytokinesis. (A) Degradation and re-synthesis of the *PI3K* subunit *p110* is driven by *PLCγ*-dependent activation of the *NEDDL4* (*red links*) and *FoxO3 (orange link)*, respectively. During G2, loss of strong *PI3K / AKT1* signaling is required for nuclear translocation of *FoxO3* and/or *FoxO1*, which aids *Plk1* accumulation to levels that can outlast its degradation in anaphase (modeled via the *Plk1*_*H*_ node). During telophase this remaining pool of *Plk1* localizes to the central spindle and promotes the assembly of a contractile ring by recruiting the *RhoA* GEF *Ect2*. *Red nodes*: key pathway linking *PI3K* and *AKT1* dynamics to *Plk1* and cytokinesis. (B) *Plk1* inhibition at different points along the cell cycle leads to four distinct failure modes. *Image credits*: *https*:*//commons*.*wikimedia*.*org/wiki/File*:*Mitosis_cells_* *sequence*.*svg*.

Linking *PI3K* oscillations to the rhythm of cell division required an update of our previously published *Phase Switch* [11] to include the multifunctional *Plk1* protein required for all phases of mitosis and cytokinesis [54]. According to our model, during normal cell cycle progression the low-*PI3K* / low-*AKT1* phase of the *PI3K* oscillations lead to nuclear re-entry of *FoxO3* and *FoxO1*, which aid the accumulation of *Plk1* and are required for cytokinesis. In addition, we predict that the inhibitory influence of *Plk1* on *FoxO3* [41] helps lock *PI3K* oscillations to the cell cycle (**Fig 4**). That said, a strict phase-locking is only enforced at the metaphase / anaphase transition, as evidenced by the behavior of the asynchronous version of our model (**Figs 5** and **S5**). In addition to offering testable predictions of the mechanisms behind *PI3K* oscillation summarized in **Fig 9**, our model is the first to account for the multifaceted role of *Plk1* in cell cycle progression (**Fig 6**). Namely, we were able to reproduce G2 arrest in the complete absence of *Plk1* [55], mitotic catastrophe in response to *Plk1* removal in metaphase [54,56–58], the potential for premature *APC/C*^*Cdh1*^ activation and chromosome mis-segregation [59], as well as failure to carry out cytokinesis in the absence of a *Plk1* pool that survives *APC/C*^*Cdh1*^-mediated destruction [60–62].

A limitation of our current model stems from uncertainties in the experimental literature on the connection between *Plk1* and *FoxO* factors. As we detailed in *Results*, the combinatorial regulation of *Plk1* by *FoxM1*, *FoxO3* and *FoxO1* is not characterized. It is not clear whether these factors cooperate or independently augment *Plk1* expression. Moreover, our assumption that either *FoxO* factor alone can boost *Plk1* sufficiently to survive until telophase has not been tested *in vitro*. Thus, the logic gates connecting *Plk1* and the *FoxO* factors may need a revision in light of additional data. That said, aspects of *Plk1* regulation that guarantee its loss in telophase but not earlier in cells with hyperactive *PI3K*/*AKT1* requires key elements of our regulatory logic to remain intact [29].

Throughout this work we used synchronous and asynchronous Boolean modeling in parallel, allowing us to leverage the advantages and mitigate the drawbacks of each update scheme. A key advantage of synchronous update is that the dynamics it generates is entirely deterministic [63]. This allowed us to probe the effects of inhibiting nodes at specific times along a dynamical trajectory such as the cell cycle, and predict distinct phenotypic outcomes depending on the precise timing of inhibition. For example, using synchronous update to model *Plk1* inhibition along the cell cycle points to a time-sensitive sequence of failure modes: G2 arrest, mitotic catastrophe and aberrant anaphase, followed by normal anaphase but failed cytokinesis (**Fig 6**). The power of these simulations is that they reveal distinct ways in which the molecular balance of *Plk1*, *Cdk1*/*Cyclin B*, premature activation of *APC/C*^*Cdh1*^, and pro-apoptotic factors accumulated by mitotic delay can be tipped (**Fig 9**). In the presence of molecular noise *in vitro*, however, we expect *Plk1* knockdown to generate a mix of these cell cycle errors. Indeed, experiments indicate that mitotic death and aberrant anaphase co-occur in *Plk1*-inhibited cells [59]. To reproduce this, we simulated the partial stochastic inhibition of *Plk1*, resulting in a changing mix of errors with both synchronous and asynchronous update (**Fig 7**). Our success with the latter is especially helpful for showing that the four failure modes are not artifacts of non-biological synergies in signal arrival, a pitfall of synchronous update.

Comparing cell cycle progression with the two update schemes revealed that asynchronous update introduces a stochasticity in cell cycle entry, observed in several mammalian cell lines [90,97,98]. This is similar to the behavior of the synchronous model in non-saturating environments, and it is largely due to the fact that the *PI3K/AKT1* cycle does not stay in sync with cell cycle progression for most of the cycle. As a result, the ability of *AKT1* to relay growth signals to the *Restriction Switch* in late G2 / early metaphase, and thus pre-commit cells to another division, remains stochastic under asynchronous update even in saturating growth environments. Cycling cells *in vitro* are likely somewhere in between; less random in their ability to re-commit during G2/M than the asynchronous model, but not deterministic either. In addition to cell cycle commitment, overly noisy signal propagation is likely responsible for the asynchronous model’s results in cells with hyperactive *AKT*_*H*_ and low *FoxO3* (**Figs 8C** and **S13E***)*. In contrast to simulations with synchronous update, the fraction of cells that failed to complete cytokinesis under asynchronous update was small. Here we think that synchronous update overestimates, while asynchronous update underestimates the rate of this cell cycle error. In summary, our parallel use of the synchronous and asynchronous Boolean frameworks helped us uncover subtle inter-dependencies in the dynamics of our coupled regulatory modules, but also guaranteed that our results are robust with respect to noise in signal propagation and do not depend on non-biological synchrony of parallel signals with a variety of speeds.

An intriguing model prediction related to the coupling of the cell cycle and the *PI3K* cycle leverages our model’s ability to reproduce pre-commitment to another cycle at the G2/M transition (**Fig 5**) [90], a feature inherited from our previous cell cycle model [11]. Even though high *p110* expression is required for cell cycle entry from quiescence (**S8 Fig**) [46], we predict that under saturating growth factor conditions high *p110 / PI3K* is *not* required for pre-commitment to another cycle (**S10 Fig**). This is surprising, as pre-commitment at the G2/M boundary normally coincides with the *AKT1*-high portion of the second *PI3K* cycle. Our model suggests that the stabilization of *Myc* in *p110*-low cells occurs in pro-metaphase in spite of low *AKT1* and high *GSK3β*, owing to increased activity of *mTORC1* driven by *Cdk1/Cyclin B*, specifically in the presence of *GSK3β* [96]. Experimental validation of these predictions is an important step toward understanding the complex interplay of factors that control *Myc* expression and pre-commitment to another cycle at the G2/M boundary. The same experimental setup that showed the existence of pre-committed cells could accomplish this [90] by probing the effect of simultaneous *MEK* and *PI3K* inhibition on pre-commitment. According to our prediction, this dual inhibition would reduce but not eliminate the fraction of cells that finish their current cycle following *MEK* and *PI3K* inhibition, then complete another.

Our modeling results on partial *Plk1* inhibition offer a cautionary note on targeting *Plk1* as a tumor-suppressive strategy. On one hand, *Plk1* inhibition does significantly limit proliferation due to G2 arrest (**Fig 6A**) and promotes apoptosis in cells that escape from G2 via mitotic failure (**Fig 6B**). On the other hand, our model predicts that an ill-timed, short-lived pulse of *Plk1* inhibition can lead to faulty anaphase, chromosome mis-segregation resulting in aneuploidy, and subsequent genome duplication (**Fig 6C**). According to our model, weak *Plk1* inhibition in individual cells is especially dangerous in this regard (**Fig 7**). Thus, it is possible that cancer therapy based on *Plk1* inhibition [99] could *increase* genomic instability in cells that survive it. A propensity for aneuploidy and genome duplication was indeed observed in *Plk1*-inhibited cells [59], but also in a mouse model harboring the oncogenic mutation in the alpha subunit of *PI3*-*Kinase* [100]. The molecular mechanisms behind the latter were never explained. Our model not only reproduces these outcomes (**Table 3**), but also points to the ill-timed loss of *Plk1* as the likely culprit (**Fig 6**).

Looking ahead, our current model lays the groundwork for modeling the mechanisms of *AKT1*-induced senescence [38,39,100]. An extended version of our model with DNA damage-induced G2 arrest in cells with hyperactive *AKT1* would express most key drivers of senescence (i.e., *mTORC1*, *RB*, *p53* and *p21*). Thus, an important next goal is to complement our model with a DNA damage signaling module [16–18,101,102], then build the regulatory switch that locks in and maintains senescence [20,24,103–105]. The predictive power of our model could be further expanded by revising our *Growth Signaling* and *Apoptosis* modules to leverage more detailed computational models of *MAPK* signaling [25], as well as the apoptosis/necrosis decision [15]. Finally, building a contact inhibition module to capture the connection between cell-cell contacts and *p110* expression could pave the way towards modeling interacting epithelial cell communities [106]. We thus see our current model as a seed for more powerful models of the processes that go awry when healthy cells transition into malignancy.

## Methods and model

### Boolean modeling framework

To capture the complex combinatorial logic by which the 87 molecular species and cellular processes in our model interact, we used a Boolean network modeling formalism [63]. Boolean models approximate the activity of regulatory molecular species as ON (expressed and active) or OFF (not expressed or inactive) [107], and focus on the combinatorial logic by which multiple regulatory inputs work together. This requires specifying the ON/OFF response of each node for every combination of the states of its inputs. The resulting Boolean functions (gates) can be represented as truth tables (input-output tables that specify every response of a node explicitly), or via the Boolean logic operators AND, OR, and NOT (**S1 Table**). Once the Boolean gate of each node is specified, the time-dependent dynamics of the whole network can be simulated from an arbitrary initial condition [63]. Since the expression / activity of the molecules is discrete, time also proceeds in discrete steps in which nodes can change their ON/OFF state.

#### Synchronous update

During the construction of our model we used synchronous update [108], a scheme in which every node of the network updates synchronously in every time-step. The advantage of this scheme is that it renders the dynamics of the system completely deterministic. This allowed us to account for the precise role of each molecular species at every causal step along a biological process and compare it to experimental data. By building a synchronous Boolean model first, we could follow the molecular causes of behaviors that deviated from known cell dynamics, and to fix the model to better match experimental evidence.

#### Asynchronous update

Asynchronous update changes the state of one node at a time and uses this new state as it updates its targets. In *general asynchronous update*, nodes are chosen randomly in each time-step regardless of the last time they were updated (**Fig 3C**). In contrast, *random order asynchronous models* update every node in every time-step, but they do so sequentially in a random order re-shuffled before every step (**S1 Fig**). Asynchronous update schemes are favored in biological modeling, as they simulate the unfolding of the same regulatory process along a large number of slightly different paths, each with different likelihood [109], mimicking a type of stochasticity present *in vitro* [110]. Moreover, asynchronous update eliminates potential artifacts of synchronous modeling; behaviors that rely on perfect and deterministic coordination of parallel signals–a condition that cells rarely satisfy. That said, they can also generate biologically non-realistic sequences of molecular events by failing to follow up on the effects of short-lived signals that live cells reliably respond to (**Figs 5** and **S1**). To mitigate this, we used a hybrid framework where the update order of some, but not all nodes is not random (termed the *biased asynchronous model*, **Fig 5**). As most nodes in our model are controlled by slow as well as fast processes, setting their update frequency was not a viable strategy. Instead we choose to update 11 of the 87 nodes either first or last, depending on their correct state, as detailed in **S3 Table**.

### The state space of a Boolean regulatory network

In a Boolean representation, a regulator network can have 2^*N*^ possible expression / activity profiles, where *N* represents the number of molecules in the model. Starting a time-series from most of these 2^*N*^ states reveals that they are not stable, in that several regulatory nodes immediately change their ON/OFF state as dictated by the ON/OFF state of their inputs. Allowing the network’s dynamics to proceed from an unstable state will lead to a sequence of expression/activity changes that can cascade through the network. Eventually, every such cascade must end in two ways, regardless of update: 1) a stable state in which all Boolean rules are satisfied (called a ***point attractor***), or 2) a more complex set of states that a) repeat in an exact cycle termed a ***limit cycle attractor*** under synchronous update, or b) repeat in a more stochastic sequence of states called a ***complex attractor*** that traps the dynamics under asynchronous update. The latter can also represent a rhythmic, repeating series of state-changes, but this is not guaranteed. The collection of sequential state-changes running from each of the 2^*N*^ model states to the model’s attractor states or cycles can be represented as a directed network of states, termed the *state transition graph*.

Under ***synchronous update*** the model’s dynamics leads to a single attractor from each unstable state. The collection of all the paths leading to the same final state creates a subgraph of the state transition graph, and represents the *attractor basin* of the final attractor state [111]. Conceptually, this attractor basin can be thought of as a valley in the pseudo-energy landscape of the model [112]. As most network states are unstable and lead, in time, to an attractor, biologically relevant robust phenotypes of the model are expected to correspond to its attractor states [113]. Moreover, rhythmic biological behavior such as that of a continuously cycling cell is expected to map onto a limit cycle attractor.

Under ***asynchronous update***, some unstable states can lead to different attractors with different probabilities depending on update order, while others can only lead to a single attractor—making the definition of attractor basins less straightforward. In addition, regions of the state transition graph can act as metastable “valleys” (**S5 Fig**). These represent state collections that trap the dynamics of the system for long periods of time, but it is not strictly speaking impossible for the system to escape to a proper attractor. Indeed, the cell cycle in our asynchronous models is such a metastable “pseudo-attractor”.

### Reproducing our modeling results

To simulate the dynamics of our Boolean model and work through key methods, see “*SI_notebook*.*ipynb”* in **S2 File**, a Jupyter Notebook in Python (*https*:*//jupyter*.*org*). The code in this notebook uses BooleanNet [86] and NetworkX [67]. **S3 File** contains BooleanNet model files, including the full model (“*PI3K_cell_cycle_apoptosis”*). To convert these files to commonly used formats used by other packages, see (*http*:*//colomoto*.*org/biolqm/doc/formats*.*html*).

For synchronous update, see **S2 File -- 1.a** for the *PI3K* oscillator and **S2 File -- 3** for the full model. To run the *PI3K* oscillator module using general asynchronous update see **S2 File -- 1.b**; for the full model with random order asynchronous and biased asynchronous update see **S2 File -- 4.a-c**. To sample the full state space of the individual network modules, see **S2 File -- 2**; to sample and visualize the general asynchronous state transition graph of the *PI3K* oscillator, see **S2 File -- 1.c**; to map the cell cycle pseudo-attractor of the full model, see **S2 File -- 4**. Both files are available as a package at *https*:*//github*.*com/deriteidavid/cell_cycle_apoptosis_Sizek_etal_PloSCompBio_2019*.

### Mapping the attractor landscape of large Boolean networks using synchronous update

In order to generate a comprehensive picture of all the attractor basins of the model, we use a stochastic state space sampling procedure adapted from [114], as described in [11]. To this end, we first implemented a noisy version of synchronous Boolean dynamics, in which each regulatory node is affected by a small amount of noise in every time-step. The noise is implemented as a small probability *p*_*n*_ = 0.02 that each node generates the incorrect output, rather than the one dictated by its inputs [112]. This noisy dynamics sets up a Markov process, guaranteeing that the system can spontaneously visits any state (not just the attractors) with non-zero long-term probability [112,115]. We used the noisy dynamics to aid our sampling procedure by starting the network from a random initial condition and simulating a time-course of *N*_series_ = 20 noisy time-steps. As the model generates this noisy dynamical trajectory, the algorithm pauses at each state it visits to perform two checks. First, it finds the attractor basin this state would fall into if the dynamics were to continue in a deterministic fashion. Second, it scans the immediate neighborhood of this state by enumerating every state the system could reach from the current one via a *single* node-state flip and identifying *their* attractor membership (via deterministic dynamics). This allows the algorithm to access parts of the state space the noisy dynamics might never go near, and to find even small basins relatively fast. As a result, the algorithm is quite slow on random Boolean networks with large numbers of small basins. Our model’s robust phenotype-representing attractor basins, by contrast, are typically large and thus rapidly found. The full algorithm descried in [11] tracks the visitation probability of each state, basin and transition (not used here). The only update to the algorithm since [11] involves partitioning the full state space of the model into sub-spaces corresponding to each unique environmental node state-combination and sampling each subspace from *N*_rnd_ = 500 random initial conditions.

### Automated isolation of a subnetwork from a larger (multi-switch) Boolean network

In order to automatically model the dynamical behavior of any isolated subgraph (**Fig 3A**), we have previously developed an algorithm that defines the Boolean gates of nodes when they lose some of their incoming connections [11]. The main goal of this algorithm was to optimally preserve the regulation of a node by its remaining inputs. Briefly, whenever a subset of inputs is removed from a Boolean gate, the algorithm assumes that they are frozen into either an ON or an OFF state. To best preserve the dynamical influence of the remaining nodes, it finds *one* of the 2^*k*^ possible combinations of frozen inputs such that: a) all remaining input nodes are functional (i.e., they are able to impact the output in some way), and b) the entropy of the remaining Boolean gate fragment, *H*_G_ = - *p* · log(*p*)*-* (1 *- p*) · log(1 *- p*), is as large as possible (*p* is the fraction of OFF-outputs). For easy reproducibility of our module networks, **S3 File** includes a *BooleanNet* model file for each module.

### Boolean network modules representing distinct cellular regulatory functions

#### Growth factor signaling

To build a dynamic *PI3K* → *AKT1* signaling module (**S1A Table**), we first incorporated the canonical *PI3K* → *AKT1* pathways shown in **Fig 1A** (reviewed in [31,32]). Namely, we modeled growth factor stimulation leading to receptor tyrosine kinase (RTK) activation, which in turn activates the *PI3K* enzyme at the plasma membrane [30]. *PI3K* produces PIP_3_, which then recruits both the *PDK1* kinase and its phosphorylation target, *AKT1*. *PDK1* then phosphorylates *AKT1* at one of its two key sites, T308. To account for basal growth factor signaling that promotes cell survival versus the strong but short-lived activation of *AKT1* in response to high growth factor stimulation, we tracked the activity of both *PI3K* and *AKT1* via two Boolean nodes *(PI3K* and *AKT*_*B*_ for basal activity*; PI3K*_H_ and *AKT*_H_ for peak activation). Next, we added key downstream targets of *AKT1*: 1) *AKT1* activates *mTORC1* signaling by inhibitory phosphorylation of *TSC2*, responsible for the inactivation of the Rheb GTPase [33,34]. *mTORC1* targets *S6K* and the translation initiation factor *eIF4E* (held in check by *4E-BP1* in the absence of active *mTORC1)*, both of which aid cell cycle commitment [116] (**Fig 1A**, box 1). 2) *AKT1*-mediated inhibition of *GSK3β* counteracts the destabilizing effects of this kinase on several cell cycle-promoting genes such as *Myc* and *cyclin D1*, as well as the anti-apoptotic *BCL-2* family member *MCL-1* (**Fig 1A**, box 2) [35]. 3) *AKT1* aids cell cycle entry and cell survival by translocating *FoxO* factors out of the nucleus. *FoxO* targets include apoptotic proteins such as *BIM*, and cell cycle inhibitors such as *p27*^*Kip1*^ and *p21*^*Cip1*^ (**Fig 1A**, box 3) [36]. 4) *AKT1* phosphorylates the pro-apoptotic *BCL-2* family member *BAD*, leading to its translocation and sequestration from the mitochondrial membrane (**Fig 1A**, box 4) [37].

In addition, our model incorporates receptor-independent feedback mechanisms known to temper *PI3K*/*AKT1* signaling; namely the effects of *S6K* on *mTORC2* inhibition and *PTEN* translocation to the cytosol [45,117]. The two feedback loops that control high *p110* expression (**Fig 2**) are detailed in *Results* (module available as the *“PI3K”* BooleanNet file in **S3 File**). Finally, the *Growth Signaling Module* includes a linear MAPK cascade that is i) required for maximal *PI3K* activity [91], ii) aids the activation of *mTORC1* and the inhibitor of *FoxO3* as well as *GSK3β* [118–120], iii) drives the expression of *Myc* during cell cycle entry [121], and iv) contributes to survival signaling [122].

#### The restriction switch and the mitotic phase switch

To model the switch-like restriction point control guarding cell cycle entry, we used the *p21*-positive version of our previously published *Restriction Switch* (**Fig 3A** and **3B**; **S1B Table**; module available as the *“Restriction_Switch”* BooleanNet file in **S3 File**) [11]. This switch has two stable states in isolation, representing cells before and after they pass the restriction point. Next, we expanded our previously published *Phase Switch* [11] to account for the critical role of the *FoxO* target *Plk1* (**Fig 3A** and **3B**; **S1C Table**; module available as the *“Phase_Switch”* BooleanNet file in **S3 File**). *Plk1* is required for normal cell cycle progression, as animal cells do not assemble a bipolar spindle in its absence [54]. It is activated in early G2 by the *FoxM1* transcription factor [123,124]. *FoxM1* transitional activity is also essential of the completion of the mitotic program, above and beyond its effect on *Plk1* [125,126]. *FoxM1* levels are kept low by auto-repression in quiescent and G1 cells. During the S/G2 transition this inhibition is relieved by *Cdk2* phosphorylation (in complex with *cyclin E* or *A*) [127], and later by active *Cdk1* [128]. Once active, *FoxM1* regulates several of proteins involved in cell cycle progression, of which our model includes *Cdc25A*, *Cdc25B*, *Plk1* and *cyclin B* (we do not explicitly include the activity of the *Skp1/cullin/F-boxp* protein complex active at the G1/S transition; thus its *FoxM1* target components are also missing from our model) [125]. In addition to *FoxM1*, accumulation of sufficient *Plk1* to drive cytokinesis (modeled by the *Plk1*_H_ node) also requires at least one FoxO factor [29,40]. Finally, *Plk1* degradation is driven by *APC/C*^*Cdh1*^ [61].

To model the downstream effects of *Plk1*, we first incorporated positive feedback on its own transcriptional activator, *FoxM1*. *Cdk1*-phosphorylated *FoxM1* was shown to bind *Plk1*, and further phosphorylation by *Plk1* is required for full transcriptional activity during the G2/M transition [128]. To model this, *FoxM1* requires either *Cyclin E* or *Cyclin A*-bound *Cdk2* activity, or the simultaneous presence of *Cyclin B*/ *Cdk1* and *Plk1* (**S1C Table**). Second, we added a *Plk1* requirement to the activation of *Cdc25C*, as the absence of *Plk1* prevents nuclear localization of *Cdc25C* during prophase [129], and *Plk1*-mediated *Cdc25C* phosphorylation is required for its activity [55]. Third, *Plk1* cooperates with active *Cdk1*/*Cyclin B* complexes to target the *APC/C* inhibitor *Emi1* for destruction [130]. This ensures that *Emi1* is no longer present to interfere with *APC/C*^*Cdc20*^ activation when cells clear the spindle assembly checkpoint [131].

A few of *Plk1*’s effects extend outside the new *Phase Switch*. First, localization of *Plk1* to unattached kinetochores during metaphase is required for the formation of stable microtubule attachments [54]. To model this we require active *Plk1* for the completion of the mitotic spindle, represented by the Attached Kinetochores (*A_Kinetochores)* node. Second, *Plk1* is targeted to the spindle midzone during and after anaphase, where it helps recruit *Ect2* to the central spindle [62]. *Ect2* is a RhoGEF that aids the accumulation of GTP-bound *RhoA* [62], and thus the formation of the contractile ring [132]. To account for the role of *Plk1* at the central spindle, past the point where a large fraction of *Plk1* is degraded, we included a *Plk1*_*H*_ dependent *Ect2* node required for the cytokinesis step of our model (marked by de-activation of the 4N_DNA node). Third, *Plk1* feeds back to tag *FoxO3* and *FoxO1* for export from the nucleus [41,42]. The addition of *Plk1*, *FoxM1* and *Emi1* to our previously published *Phase Switch* resulted in the module on **Fig 3A**. Its three stable states represent the robust expression patterns seen in cells in G0/G1, during G2 (before passage of the DNA damage checkpoint), and at the spindle assembly checkpoint (SAC).

#### The origin licensing switch

To model the dynamics of origin of replication licensing, we built a small bistable regulatory switch that tracks the assembly and firing of replication origins (**Fig 3A and 3B**; **S1D Table**; module available as the *“Origin_Licensing_Switch”* BooleanNet file in **S3 File**) [133,134]. First, the *assembly* of a functional replication complex requires DNA binding of Origin of Replication (*ORC*) proteins, marking the origins of replication along mammalian chromosomes [135]. *ORC* proteins then recruit *Cdc6* (transcribed in late G1 by *E2F1*) [136], and the resulting *ORC/Cdc6* complexes further recruit *Cdt1* (also an *E2F1* target) [137]. Next, the heterohexameric complex of *MCM2*–*7* proteins binds, completing the pre-replication complex (*Pre-ORC*) [135]. Once assembled, the complex remains stable and protected from disassembly until *Cdk2*-dependent phosphorylation of *Cdc6* triggers the firing of replication origins, and dissociation of *Cdc6* [138]. At this point, a replication bubble is formed by the helicase action of the *MCM* complex, and the *Pre-ORC* falls apart [139]. In addition to the degradation of phosphorylated *Cdc6* [138], mammalian *Cdt1* and *ORC* proteins are also degraded at this point, likely due to *Cdk*-dependent phosphorylation and ubiquitination [140].

To model the stability of the assembled *Pre-ORC*, we included a series of positive feedback links from the *Pre-ORC* node (representing the assembled complex waiting to fire) to its components, *ORC*, *Cdc6* and *Cdt1*. As a result, the isolated module has two stable states; ON and OFF (**Fig 3A**). As the cell cycle progresses, this switch is toggled ON when *E2F1* activates its components (as long as *geminin* does not block *Cdt1*, and *Plk1* does not sequester *Cdc6* to the spindle pole or the central spindle) [141,142]. Conversely, it is toggled OFF by *Cdk2*-mediated destruction of *Cdc6*, and the start of DNA synthesis at each origin. The firing of all origins in a mammalian cell, however, does not occur in one instant [133]. The handoff of origin firing from early to late-replicating genes is accompanied by a handoff of *CyclinE/Cdk2* to *CyclinA/Cdk2* complexes [141]. This ongoing process is not trivial to represent in the context of a Boolean model, where the cell-wide availability of licensed *ORCs* is tracked by a single Boolean node (namely *Pre-ORC*). In order to make sure that the turning OFF of this node in our model represents the firing of *all* origins required for successful replication, we placed *Cdc6* under the inhibitory control of *Cyclin A*. In addition, *Pre-ORC* is turned off by the completion of DNA synthesis, marked by the appearance of *4N_DNA* in the context of a *Replication* node that is still ON.

#### Cellular processes during cell cycle progression

The above three switches control cell cycle passage by triggering the processes of DNA replication, spindle assembly and cytokinesis (**Fig 3B**, orange nodes; **S1E Table**). To model this, we included the *Replication* and *4N_DNA* nodes from our published cell cycle model [11], an unattached kinetochore node (*U_Kinetochore*) to denote incomplete mitotic spindle assembly, and attached kinetochore (*A_Kinetochore*) to mark completion of the mitotic spindle. These process-nodes are accompanied by key regulators of the coupling between the regulatory switches and the processes themselves. Namely, *ATR* and *CHK1* are activated during replication to monitor the completion of DNA synthesis by blocking the G2/M transition [142], *Mad2* is a SAC protein that blocks anaphase entry before the mitotic spindle is complete [143], active *Ect2* marks ongoing cytokinesis [143], while *CAD* (Caspase Activated DNAase) fragments DNA in apoptotic cells [144]. Finally, *Plk1*_*H*_ represents a sufficiently large *Plk1* pool to briefly outlive *APC/C*^*Cdh1*^-mediated destruction, and aid cytokinesis. These cell cycle processes, in turn, feedback to influence the control switches. For example, completion of the mitotic spindle (marked by *A_Kinetochore*) blocks *Mad2*, thus relieving the inhibition of *APC/C*^*Cdc20*^ and flipping the *Phase Switch* from SAC to G0/G1.

#### The apoptotic switch

To accurately capture events that can kill cells in the absence of DNA damage—i.e., complete growth factor withdrawal, extrinsic apoptotic signals or mitotic catastrophe, we built on previously published models of apoptotic commitment to create a detailed Boolean version of this regulatory switch (**Fig 3A** and **3B**; **S1E Table**; module available as the *“Apoptotic_Switch”* BooleanNet file in **S3 File**) [12–15,69–71]. Briefly, the switch is flipped when extrinsic signals from death receptors (**S3B Fig**), intrinsic signals such as loss of survival signaling (**S3C Fig**), or mitotic delays (**S12B Fig**) trigger Mitochondrial Outer Membrane Permeabilization (MOMP) [12]. MOMP occurs when the oligomerization of the mitochondrial membrane pore forming *BAK/BAX* proteins is triggered, releasing *cytochrome C* and *SMAC* from mitochondria to the cytosol [145]. These proteins form the *Apoptosome* [146], a platform that aids *Caspase 9* activation followed by *Caspase 3* cleavage [147]. In addition, *SMAC* deactivates the final check on executioner *Caspase 3* activity, the Inhibitor of Apoptosis (*IAP*) proteins [148]. Once active, *Caspase 3* initiates the destruction of a wide range of proteins [149], activates DNA-fragmentation by releasing the Caspase Activated DNAase (*CAD*) [144], and contributes to the switch-like functioning of the apoptotic machinery via *Caspase 6*-mediated positive feedback that leads to further cleavage of initiator caspases [70]. The resulting module has two stable states, corresponding to survival and apoptosis (**Fig 3A**).

Our model accounts for three distinct ways in which apoptosis is triggered. *First*, the extrinsic, receptor-mediated route (**S3B Fig**) is initiated by *Caspase 8* activation at death receptors [150], leading to the cleavage of *tBID* [151]. This triggers *BAK*/*BAX* oligomerization, leading to MOMP [151]. *Caspase 8* also contributes to the direct activation of the executioner *Caspase 3* [152]. Experimental evidence suggests, however, that MOMP is not only involved, but marks the moment of irreversible commitment to apoptosis [70]. The *second* mechanism we modeled is the loss of survival signals (**S3C Fig**). This triggers MOMP primarily via the loss of *BAD* phosphorylation by *AKT1*, *ERK* or *S6K* [37,153,154]. Hypo-phosphorylated *BAD* blocks the antiapoptotic *BCL-2* family proteins (*BCL2*, *BCL-X*_*L*_, *MCL-1*), which normally keep *BIM* and *BIK* (inducers of mitochondrial membrane pore formation), in check [155]. Once pore-forming *BAX* and/or *BAK* oligomerize, apoptosis proceeds as described above. *Finally*, a third path to apoptosis in our model is triggered by mitotic catastrophe marked by prolonged SAC arrest (**Fig 9B**) [53]. Loss of *CyclinB* / *Cdk1* or *Plk1* function before the completion of the mitotic spindle triggers *Caspase 2* activation [57,76], with a similar effect to that of *Caspase 8*, namely *BIK and BID* activation leading to MOMP.

### Modeling non-saturating growth factor stimulation and partial knockdown / overexpression within a Boolean framework

To generate model predictions in non-saturating growth factor conditions, we ran time courses of *T* = 50,000 or 500,000 time-steps in which the *GF*_*H*_ input node was randomly toggled ON/OFF in each time-step with a tunable ON-probability *p*_High_GF_ (**Fig 5B**) [11]. The ongoing simulations tracked the number of cell cycles completed without error (black cycle on **S6 Fig**), the number of genome duplication even from G2 (orange transition on **S6 Fig**), the number of premature metaphase-anaphase transitions that did not involve completion of the mitotic spindle followed by genome duplication (green transition on **S6 Fig**), the number of genome duplication events in the absence of a cytokinesis step between telophase and the next S-phase (red transition on **S6 Fig**), and the number of apoptotic events (purple transition on **S6 Fig** & other apoptotic events). Time courses that resulted in apoptosis before time *T* were restarted until a minimum of *T* steps of live-cell dynamics were sampled. In addition, the simulation tracked the average length of G1, S, G2, metaphase and telophase (the time cells spent with 2 nuclei, even if the cell cycle control network reset to G0/G1).

To generate model predictions with incomplete knockdown or overexpression of a target molecule, we combined the non-saturating stochastic growth factor inputs described above with a similar stochastic locking of the target molecule OFF or ON with a tunable probability *p*_KD_ (knockdown) or *p*_OE_ (over-expression), respectively. In time-steps where the molecule was not locked ON or OFF, it followed the internal Boolean regulatory influences of the rest of the network as if it was unperturbed. To run sample time courses, see **S3 File -- 1.a**; to sample cell cycle errors see **S3 File -- 1.b**.

## Supporting information

S1 Fig Random order asynchronous update often generates cell cycle progression errors. Dynamics of regulatory molecule activity during cell cycle entry from G0 using random order asynchronous update (example time-course chosen to illustrate errors). *X*-*axis*: time-steps; *y*-axis: nodes organized in modules; *orange/blue*: ON/OFF. *Black arrows*: robust *PI3K* oscillations; *white box*: normal cell cycle*; white circles*: common cell cycle progression errors (labeled).
(PDF)

S2 Fig Biased order asynchronous update occasionally generates cell cycle progression errors. Dynamics of regulatory molecule activity during cell cycle entry from G0 using random order asynchronous update (example time-courses chosen to illustrate errors). *X*-*axis*: time-steps; *y*-axis: nodes organized in modules; *orange/blue*: ON/OFF; *white circles*: cell cycle progression errors.
(PDF)

S3 Fig *Trail* exposure and growth factor withdrawal induce apoptosis. (A-C) *Top*: Molecular mechanism leading to apoptosis in response to *Trail* (A-B) and growth factor withdrawal (C). *Red background*: extracellular signal; *orange/blue background*: higher/lower than normal activity; *gradient background*: premature node transition*; no background*: other relevant node / process; →: activation; ⊣: inhibition. *Bottom*: Dynamics of regulatory molecule activity in response to *Trail* exposure in cycling (A) / quiescent (B) cells (synchronous update), or in response to complete growth factor withdrawal (biased asynchronous update, average of 1000 runs) (C). *X*-*axis*: time-steps; *y*-axis: nodes of the model organized in modules; *orange/blue color saturation*: percentage of cells in which a node is ON/OFF in each time-step; only relevant module activity is shown (full dynamics available in **S1 File**).
(PDF)

S4 Fig Random order update with bias generates state series that resemble the state sequence within the synchronous cell cycle. Overlap of states along a random order vs. biased random order asynchronous update trajectory (*y* axis) with attractor states of the synchronous cell cycle (*x* axis). *Time-step*: one randomized update round.
(PDF)

S5 Fig Heterogeneity of microstates in G1, S and G2 is due to a lack of phase-locking between the core cell cycle oscillator and the *PI3K* cycle. (A) State transition graph of the random order (*top*) vs. biased random order (*bottom*) asynchronous models, sampled for 10 independent runs of 1000 time-steps starting from each of the 21 synchronous cell cycle attractor states (cut short if the model reached apoptosis). The largest strongly connected component of each resulting state transition graph representing the cell cycle pseudo-attractor was visualized using the Kamada-Kawai algorithm (NetworkX [67], Python). (B) Projection of each state transition graph onto the sub-space defined by the expression of core cell cycle modules (*bottom*). *Nodes*: collection of all states that have identical core cell cycle node activity but differ in the activity of nodes in other modules such as *Growth Signaling*, illustrated by *linked black circles* from (A) to (B); *Node color*: cell cycle phase best approximated by each sampled state; *node size*: state visitation count; *node label*: most similar synchronous cell cycle state; *black loop (top) & black cycle (bottom)*: areas of the projected state transition graph with a cyclic pattern of transitions that match the cell cycle; *orange arrow (top)*: direct G2 → S transition (endo-reduplication); *orange box (bottom)*: G0-like pause in the G1 phase of the cell cycle, forming a distinct module apart from the G1 states of cells that pre-commit in their previous cycle.
(PDF)

S6 Fig Graphical illustration of the algorithm that tracks correct vs. erroneous cell cycle progression. *White boxes along the cycle*: activity of nodes monitored to determine the model’s cell cycle phase; *black arrows*: state transitions along a normal cycle; *colored arrows & labels*: transitions that represent errors in cell cycle progression.
(PDF)

S7 Fig Two distinct peaks of *AKT1* activity are detectable before the first G2 in quiescent cell populations entering the cell cycle. Biased asynchronous dynamics of regulatory molecule activity in response to high growth factor stimulation in a population of 1000 cells. *Orange/blue color saturation*: percentage of cells in which a node is ON/OFF in each time-step; *white boxes*: first two peaks of high *AKT*_*H*_ activity, observable before the cells loose synchrony of cell cycle progression; *white arrows*: *AKT*_*H*_ (two peaks) and 4N_*DNA* (fraction of cells that finished DNA synthesis).
(PDF)

S8 Fig High *p110* expression in G0 is required for cell cycle entry. (A) *Top*: Synchronous dynamics of regulatory molecule activity during the transition from G0 to early G1, with *p110* inhibition (*black*) before vs. after *Cyclin D* and *E2F1* activation. *X*-*axis*: time-steps; *y*-axis: nodes of the model organized in modules; *orange/blue*: ON/OFF; *black*: OFF, inhibited; only relevant module activity is shown (full dynamics available in **S1 File**). *Bottom*: Molecular mechanism leading to cell cycle commitment in response to *GF*_*H*_, before and after restriction point passage. *Black background*: *p110*_*H*_ inhibition; *orange/blue background*: high/low activity; *gradient background*: nodes in transition; →: activation; ⊣: inhibition. (B) Number of normal divisions competed in 100 time-steps (*top*) and average G1 length (*bottom*) as a function of *p110*_H_ inhibition at varying growth environments (synchronous update). *p*_High_GF_ ∈ [20%, 40%, …, 100%]; *sampling*: 500,000 time-steps. (C) Stacked bar charts showing the relative occurrence of normal cell cycle completion (*mustard*), G2 → G1 reset followed by genome duplication (*purple*), aberrant mitosis followed by genome duplication (*turquoise*), failed cytokinesis followed by genome duplication (*blue*) and apoptosis (*dark red*) as a function of *p110*_H_ inhibition, relative to the cell cycle rate in wild-type cells (*black dashed line*) at *p*_High_GF_ = 95% (biased asynchronous update).
(PDF)

S9 Fig Restriction point passage for cells entering the cell cycle from quiescence is in late G1, while cycling cells can pre-commit in late G2 of the previous cycle. (A-B) *Top*: Molecular mechanism leading to cell cycle commitment in response to *GF*_*H*_, before and after restriction point passage in quiescent (A) and cycling (B) cells, showing the failure (*left*) or success (*right*) of locking in the *Myc* ⇆ *E2F1* and *Myc* ⇆ *mTORC1* feedback loops in (A), or the *Myc* ⇆ *E2F1* loop in the presence/absence of *GSK3-β* and *Cyclin A* in (B). *Orange/blue background*: high/low activity; *gradient background*: nodes in transition; →: activation; ⊣: inhibition; *solid/dashed red arrows*: key interactions impacting / not yet impacting the outcome. *Bottom*: (A) Synchronous dynamics of regulatory molecule activity in response to 6 (*left*) or 7 (*right*) time-steps of high growth factor stimulation in quiescent cells. *White arrows & nodes*: factors driving cell cycle commitment in late G1; *dashed / solid lime green arrow*: lack of / presence of feedback from *E2F1* to *mTORC1*. (B) Synchronous dynamics of regulatory molecule activity in response to high growth factor withdrawal cycling cells during G2, before (*left*) and after (*right*) pre-commitment to another division. *X*-*axis*: time-steps; *y*-axis: nodes of the model organized in modules (showing relevant modules); *orange / blue*: ON / OFF; *white arrows & nodes*: factors driving cell cycle commitment in late G2; *dashed / solid lime green arrow*: *E2F1* inhibition (left) / lack of inhibition (right) by *Cyclin A*; only relevant module activity is shown shown (full dynamics available in **S1 File**).
(PDF)

S10 Fig High *p110* expression is not required for pre-commitment to another cell cycle in saturating growth environments. (A) Synchronous dynamics of regulatory molecule activity in response to *p110*_*H*_ knockdown past the point of commitment from G0 to the first cycle. *Lime green nodes & arrows*: pre-commitment is not driven by *E2F1* reactivation following *Cyclin A* degradation; rather, *Cdk1/Cyclin B*-mediated activation of *mTORC1* → *eIF4E* is required to stabilize *Myc* in spite of the presence of *GSK3β*. *Dark red nodes & arrows*: in the absence of high *AKT1*, *ERK* is required for two additional time-steps compared to wild-type cells, in order to stabilize the *E2F1* ⇄ *Myc* feedback loop; only relevant module activity is shown shown (full dynamics available in **S1 File**). (B) Molecular mechanism responsible for pre-commitment, before and after restriction point passage in prophase, showing the failure (*top*) or success (*bottom*) of locking in the *Myc* ⇆ *E2F1* feedback loop in the absence/presence (*top/bottom*) of *CyclinB/Cdk1*-activated *mTORC1* signaling. Black background: *p110*_*H*_ inhibition; *Orange/blue background*: high/low activity; *gradient background*: nodes in transition; →: activation; ⊣: inhibition; *solid/dashed arrows*: key interactions impacting / not yet impacting the outcome.
(PDF)

S11 Fig Modeling *Plk1* activity and persistence. Regulatory network surrounding *Plk1* expression, enzyme activity and the accumulation of a *Plk1*_H_ pool driven by *FoxO3* or *FoxO1*. *Red nodes*: two Boolean nodes representing *Plk1* activity and accumulation; *Blue nodes*: inputs of the two *Plk1* nodes. *Black arrows*: regulation and maintenance of *Plk1* expression, activity and persistence; *green arrows*: feedback on FoxO factors from *Plk1*, and its downstream target *Cyclin B/Cdk1*.
(PDF)

S12 Fig The strength of *Plk1* inhibition sets the relative prominence of cell cycle failure modes. (A) Number of normal divisions (*first panel*), mitotic catastrophe (*second panel*), aberrant mitosis with genome doubling (*third panel*) and failed cytokinesis with genome doubling (*fourth panel*) per 100 time-steps as a function of *Plk1* inhibition in varying growth environments (synchronous update). (B) Average time spent in G1 (*first panel*), G2 (*second panel*), metaphase (*third panel*) and telophase (binucleated cells in G1) (*fourth panel*) as a function of *Plk1* inhibition in varying growth environments. *p*_High_GF_ ∈ [20%, 40%, …, 100%]; *sampling*: 500,000 time-steps (synchronous update).
(PDF)

S13 Fig Partial *FoxO3* inhibition phenocopies the effects of non-degradable *p110*_*H*_, leading to a mild enrichment of telophase cells. (A-D) Number of normal divisions competed in 100 time-steps (A), average G1 length (B), number of divisions with failed cytokinesis in 100 time-steps (C), and average telophase length (D) as a function of the rate of forced *p110*_*H*_ (*first panel*), *p110*_*H*_ *+ PI3K*_*H*_ (*second panel*), *AKT*_*H*_ activation (*third panel*) and *FoxO3* inhibition (*fourth panel*) in varying growth environments (synchronous update). (E) Stacked bar charts showing relative occurrence of normal cell cycle completion (*mustard*), G2 → G1 reset followed by genome duplication (*purple*), aberrant mitosis followed by genome duplication (*turquoise*), failed cytokinesis followed by genome duplication (*blue*) and apoptosis (*dark red*) as a function of *FoxO3* inhibition, relative to the cell cycle rate in wild-type cells (*black dashed line*) at *p*_High_GF_ = 80% modeled with synchronous (*left*) and biased order asynchronous update (*right*). *Sampling*: 50,000 time-steps.
(PDF)

S1 Text Supplementary text detailing modeling results and validation that do not fall within the main focus of the study, but lend further credibility to the accuracy of the model. (A) Dynamics of *AKT1* during the cell cycle; (B) High *p110* expression in G0 is required for cell cycle entry; (C) Context-dependent timing of R-point passage; (D) Pre-commitment in *p110*-deficient cells; (E) Assumptions for constructing the regulatory logic of *Plk1* and *Plk1*_*H*_.
(PDF)

S1 Table Description and experimental support for the model’s Boolean regulatory logic. Explanation and literature support for each individual link and regulatory logic gate in the model. (A) Growth signaling; (B) Restriction switch; (C) Phase switch; (D) Origin of replication licensing; (E) Cell cycle processes; (F) Apoptotic switch.
(PDF)

S2 Table Attractors of the synchronous Boolean model. (A) Expression profile of synchronous model attractor states, numbered to match **Fig 3**; *orange/blue*: ON/OFF. (B) explanation of the molecular signatures allowing us to match them to cellular phenotypes.
(PDF)

S3 Table Biased update order and rationale. Details and logic of the biased update order required to accurately reproduce cell cycle progression, including figures (*last column*) that summarize relevant regulatory feed-forward and feedback loops susceptible to non-biological signal propagation under fully asynchronous update, mitigated by the early/late update bias on the nodes listed in the table. *Black/red/green arrows*: feed-forward / negative feedback / positive feedback; *node color*: module membership according to **Fig 3**; *translucent nodes*: updated in random order. Time traces under each network show the order of biased update among these nodes during normal cell cycle progression; *dashed horizontal line*: time-step (update-round) boundary; *orange/blue*: ON/OFF; *black up/down arrows*: timestep in which nodes turn ON/OFF.
(PDF)

S4 Table Knockout and overexertion predictions compared to experimental data. *Rows*: independent *in silico* knockout / over-expression experiment (*downward/upward arrows*), performed in stochastic non-saturating environments indicated in column 3. *Figure panels* (columns 5–6): changes to normal cell cycle and/or apoptosis as a function of inhibition / overexpression strength (*x*-axis). Each stacked bar graph shows the relative occurrence of normal cell cycle completion (*mustard*), G2 → G1 reset followed by genome duplication (*purple*), aberrant mitosis followed by genome duplication (*turquoise*), failed cytokinesis followed by genome duplication (*blue*) and apoptosis (*dark red*) as a function of node inhibition/overexpression, relative to the cell cycle rate in wild-type cells (*black dashed line*) with synchronous (*left*) and biased order asynchronous update (*right*). *Sampling*: 50,000 time-steps.
(PDF)

S1 File Additional simulation data. Full dynamics of the model for simulations shown in a truncated form on **Figs 6, 8, S3, S8, S9** and **S10**; additional simulations mentioned in **Tables 1** and **2** but not included on the figures.
(ZIP)

S2 File Jupyter Notebook in Python. Jupyter Notebook in Python that uses the BooleanNet software package to simulate synchronous and asynchronous versions of our model and reproduce our key results (“SI_notebook.ipynb”; available at *https*:*//github*.*com/deriteidavid/cell_cycle_apoptosis_Sizek_etal_PloSCompBio_2019*).
(ZIP)

S3 File BooleanNet model files. Package containing our full Boolean model as well as the 5 modules in BooleanNet format, as used by the software in **S2 File**.
(ZIP)
