# Supplementary figures and images for: Boolean model of growth signaling, cell cycle and apoptosis predicts the molecular mechanism of aberrant cell cycle progression driven by hyperactive PI3K
Source: PLoS Comput Biol. 2019 Mar 15;15(3):e1006402. doi: 10.1371/journal.pcbi.1006402 (PMC6436762; doi:10.1371/journal.pcbi.1006402)

Supplementary Figure 1

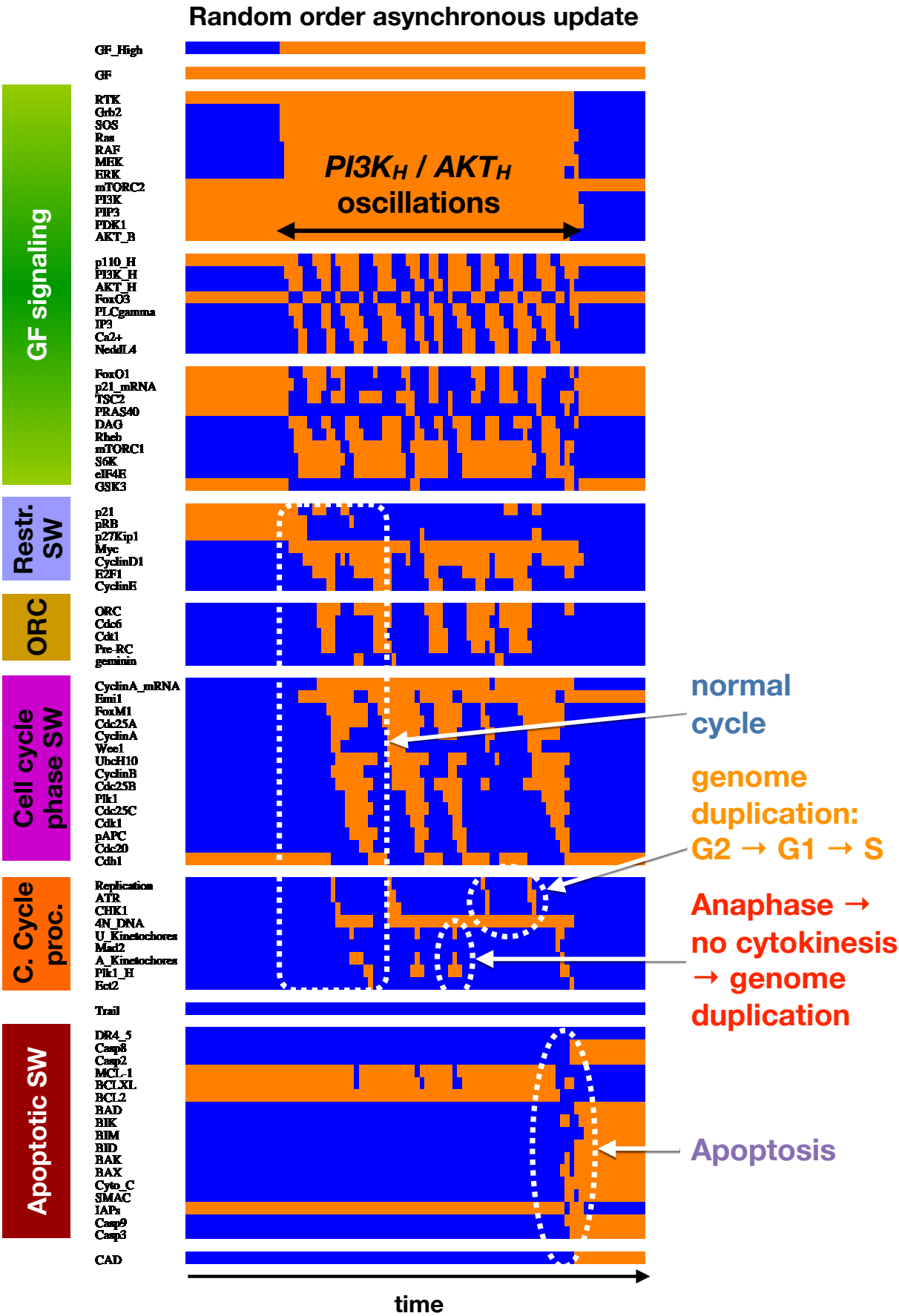

Supplement: S1 Fig — Dynamics of regulatory molecule activity during cell cycle entry from G0 using random order asynchronous update (example time-course chosen to illustrate errors). X-axis: time-steps; y-axis: nodes organized in modules; orange/blue: ON/OFF. Black arrows: robust PI3K oscillations; white box: normal cell cycle; white circles: common cell cycle progression errors (labeled). (PDF) [file pcbi.1006402.s001.pdf]

Supplementary Figure 2

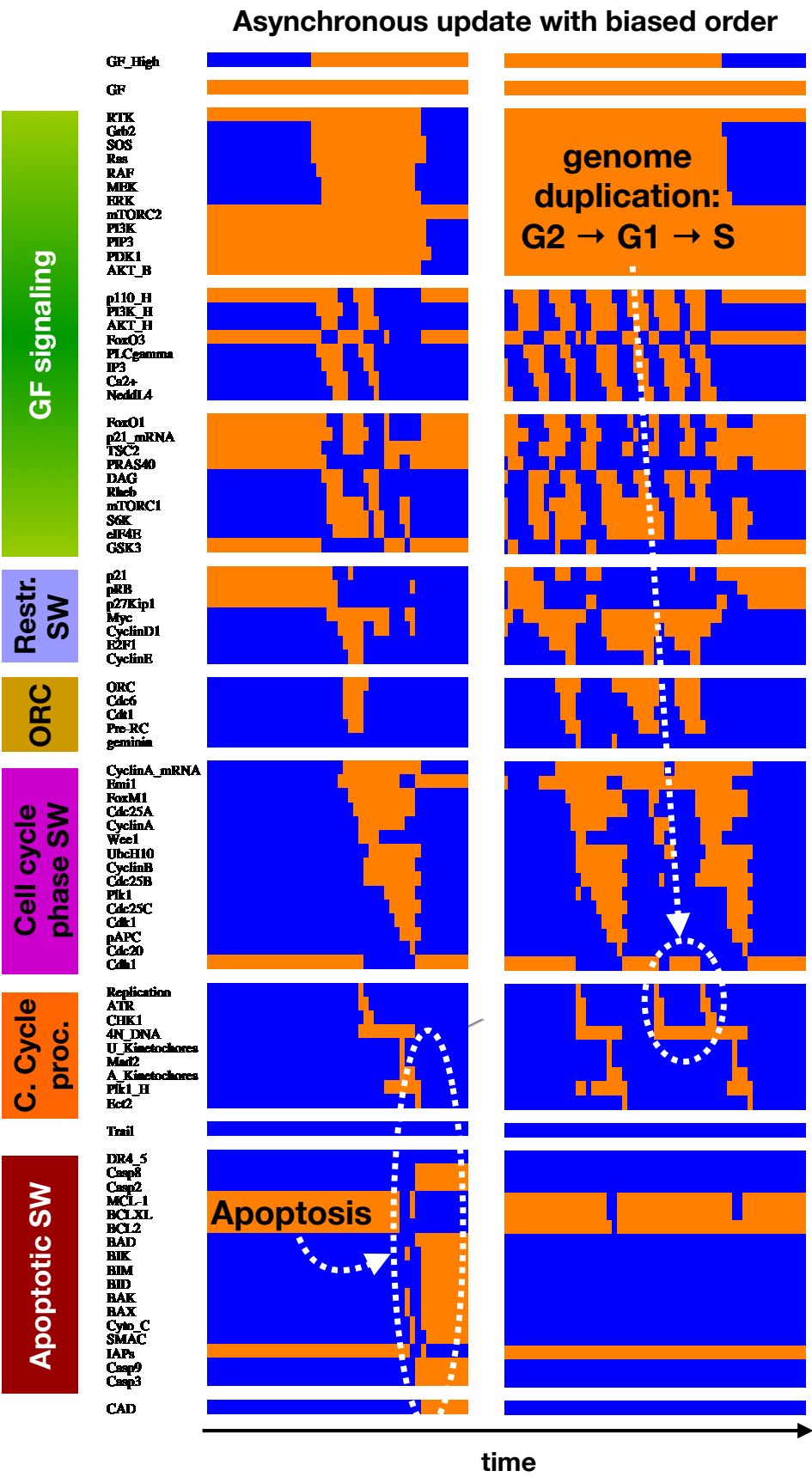

Supplement: S2 Fig — Dynamics of regulatory molecule activity during cell cycle entry from G0 using random order asynchronous update (example time-courses chosen to illustrate errors). X-axis: time-steps; y-axis: nodes organized in modules; orange/blue: ON/OFF; white circles: cell cycle progression errors. (PDF) [file pcbi.1006402.s002.pdf]

Supplementary Figure 3

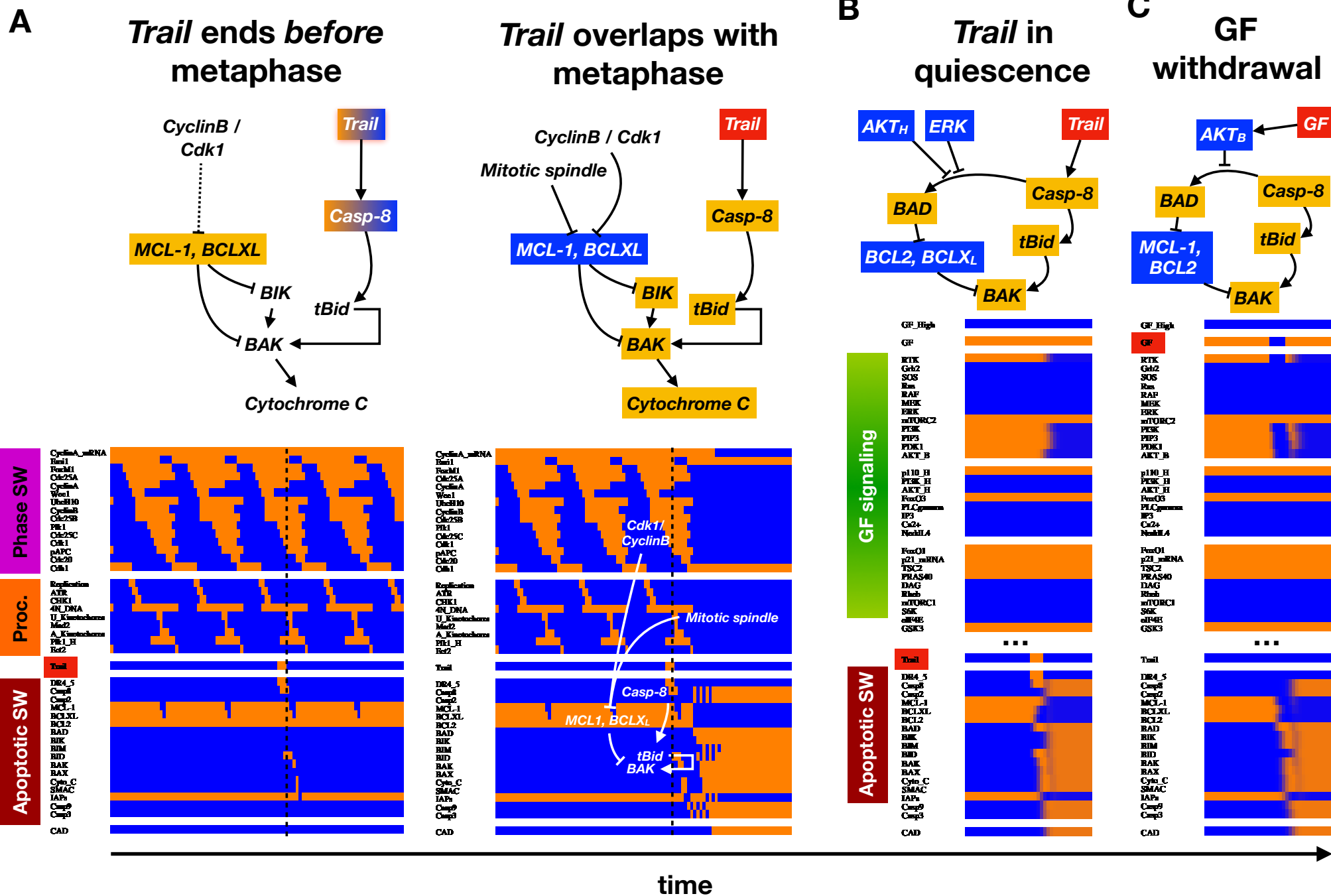

Supplement: S3 Fig — (A-C) Top: Molecular mechanism leading to apoptosis in response to Trail (A-B) and growth factor withdrawal (C). Red background: extracellular signal; orange/blue background: higher/lower than normal activity; gradient background: premature node transition; no background: other relevant node / process; →: activation; ⊣: inhibition. Bottom: Dynamics of regulatory molecule activity in response to Trail exposure in cycling (A) / quiescent (B) cells (synchronous update), or in response to complete growth factor withdrawal (biased asynchronous update, average of 1000 runs) (C). X-axis: time-steps; y-axis: nodes of the model organized in modules; orange/blue color saturation: percentage of cells in which a node is ON/OFF in each time-step; only relevant module activity is shown (full dynamics available in S1 File). (PDF) [file pcbi.1006402.s003.pdf]

Supplementary Figure 4

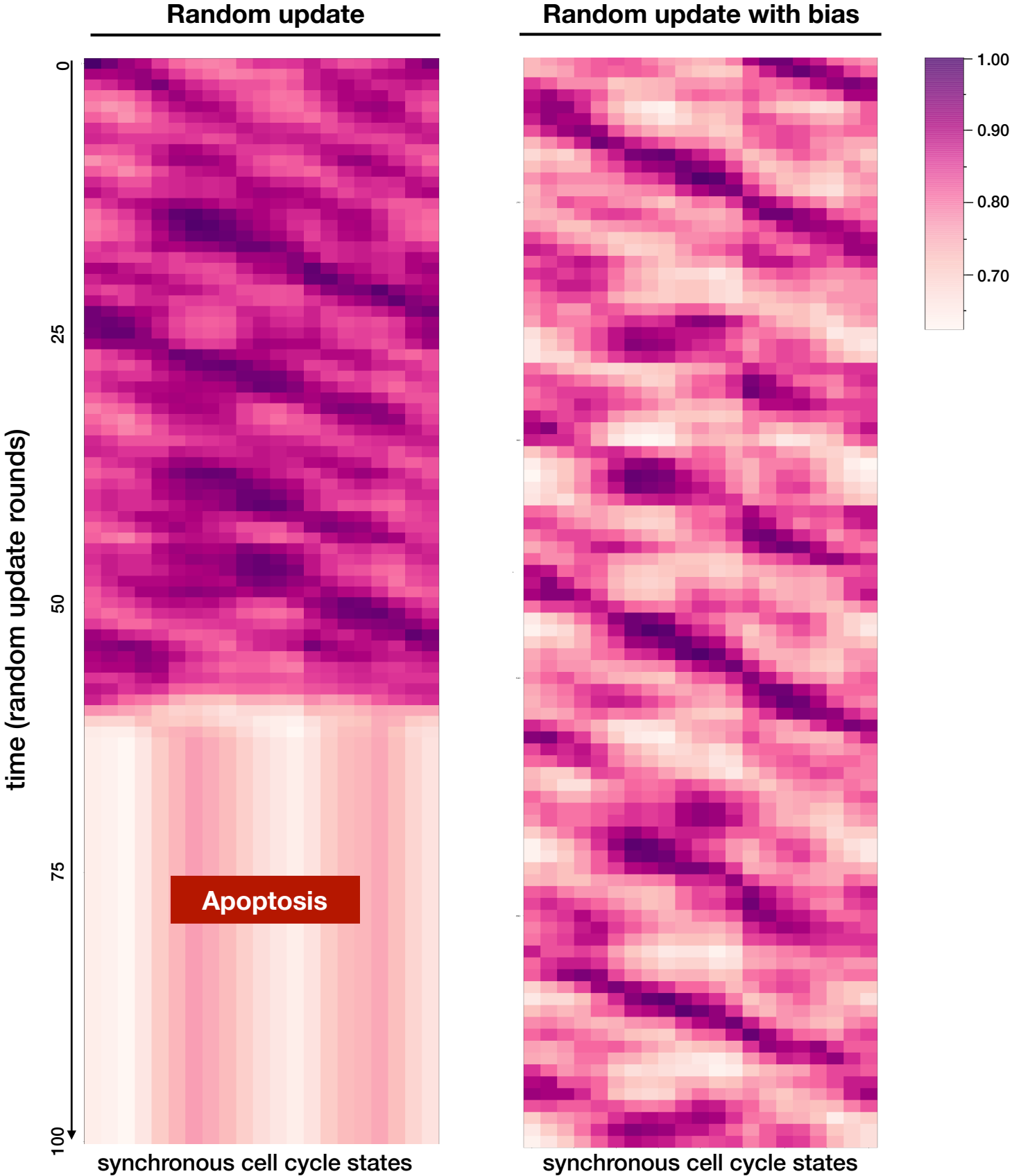

Supplement: S4 Fig — Overlap of states along a random order vs. biased random order asynchronous update trajectory (y axis) with attractor states of the synchronous cell cycle (x axis). Time-step: one randomized update round. (PDF) [file pcbi.1006402.s004.pdf]

Supplementary Figure 6

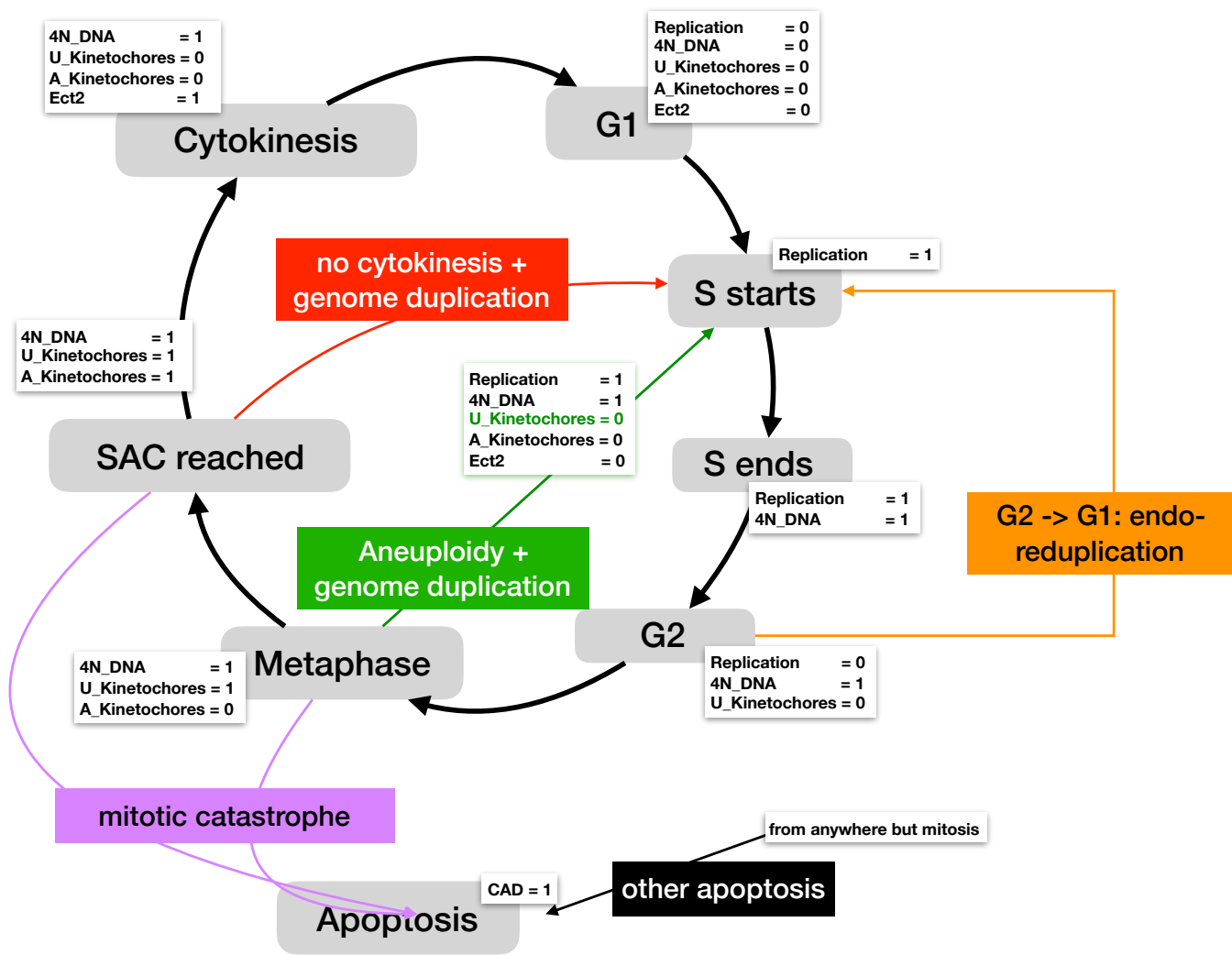

Supplement: S6 Fig — White boxes along the cycle: activity of nodes monitored to determine the model’s cell cycle phase; black arrows: state transitions along a normal cycle; colored arrows & labels: transitions that represent errors in cell cycle progression. (PDF) [file pcbi.1006402.s006.pdf]

Supplementary Figure 7

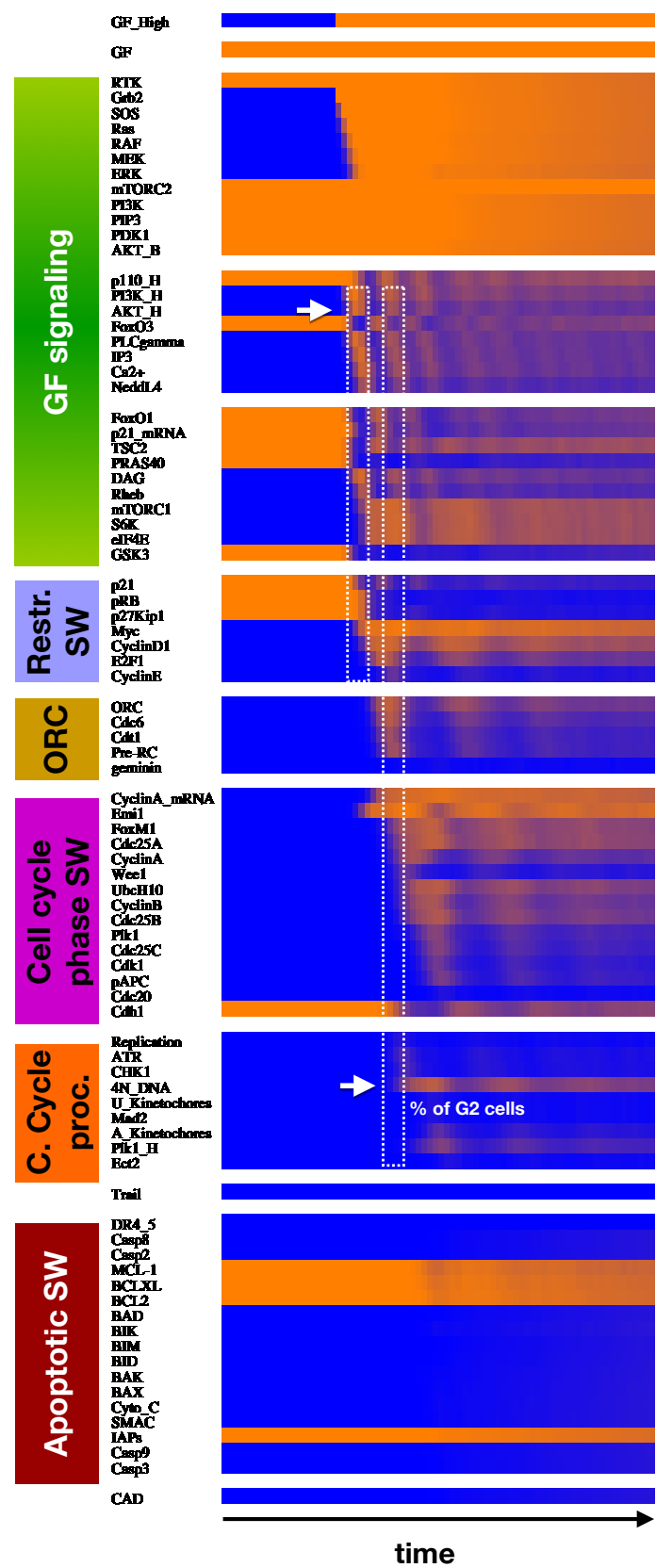

Supplement: S7 Fig — Biased asynchronous dynamics of regulatory molecule activity in response to high growth factor stimulation in a population of 1000 cells. Orange/blue color saturation: percentage of cells in which a node is ON/OFF in each time-step; white boxes: first two peaks of high AKTH activity, observable before the cells loose synchrony of cell cycle progression; white arrows: AKTH (two peaks) and 4N_DNA (fraction of cells that finished DNA synthesis). (PDF) [file pcbi.1006402.s007.pdf]

Supplementary Figure 8

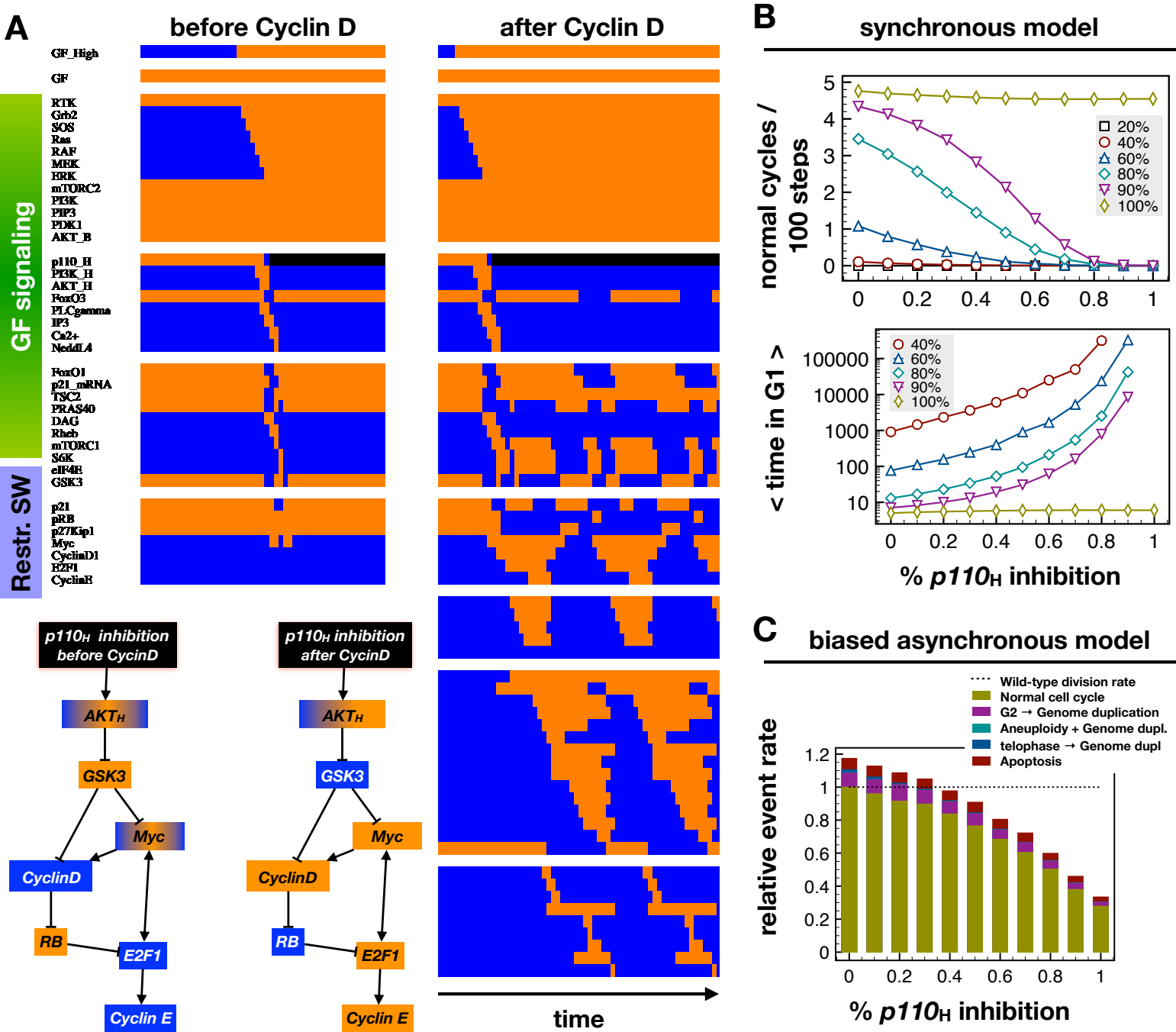

Supplement: S8 Fig — (A) Top: Synchronous dynamics of regulatory molecule activity during the transition from G0 to early G1, with p110 inhibition (black) before vs. after Cyclin D and E2F1 activation. X-axis: time-steps; y-axis: nodes of the model organized in modules; orange/blue: ON/OFF; black: OFF, inhibited; only relevant module activity is shown (full dynamics available in S1 File). Bottom: Molecular mechanism leading to cell cycle commitment in response to GFH, before and after restriction point passage. Black background: p110H inhibition; orange/blue background: high/low activity; gradient background: nodes in transition; →: activation; ⊣: inhibition. (B) Number of normal divisions competed in 100 time-steps (top) and average G1 length (bottom) as a function of p110H inhibition at varying growth environments (synchronous update). pHigh_GF ∈ [20%, 40%, …, 100%]; sampling: 500,000 time-steps. (C) Stacked bar charts showing the relative occurrence of normal cell cycle completion (mustard), G2 → G1 reset followed by genome duplication (purple), aberrant mitosis followed by genome duplication (turquoise), failed cytokinesis followed by genome duplication (blue) and apoptosis (dark red) as a function of p110H inhibition, relative to the cell cycle rate in wild-type cells (black dashed line) at pHigh_GF = 95% (biased asynchronous update). (PDF) [file pcbi.1006402.s008.pdf]

Supplementary Figure 9

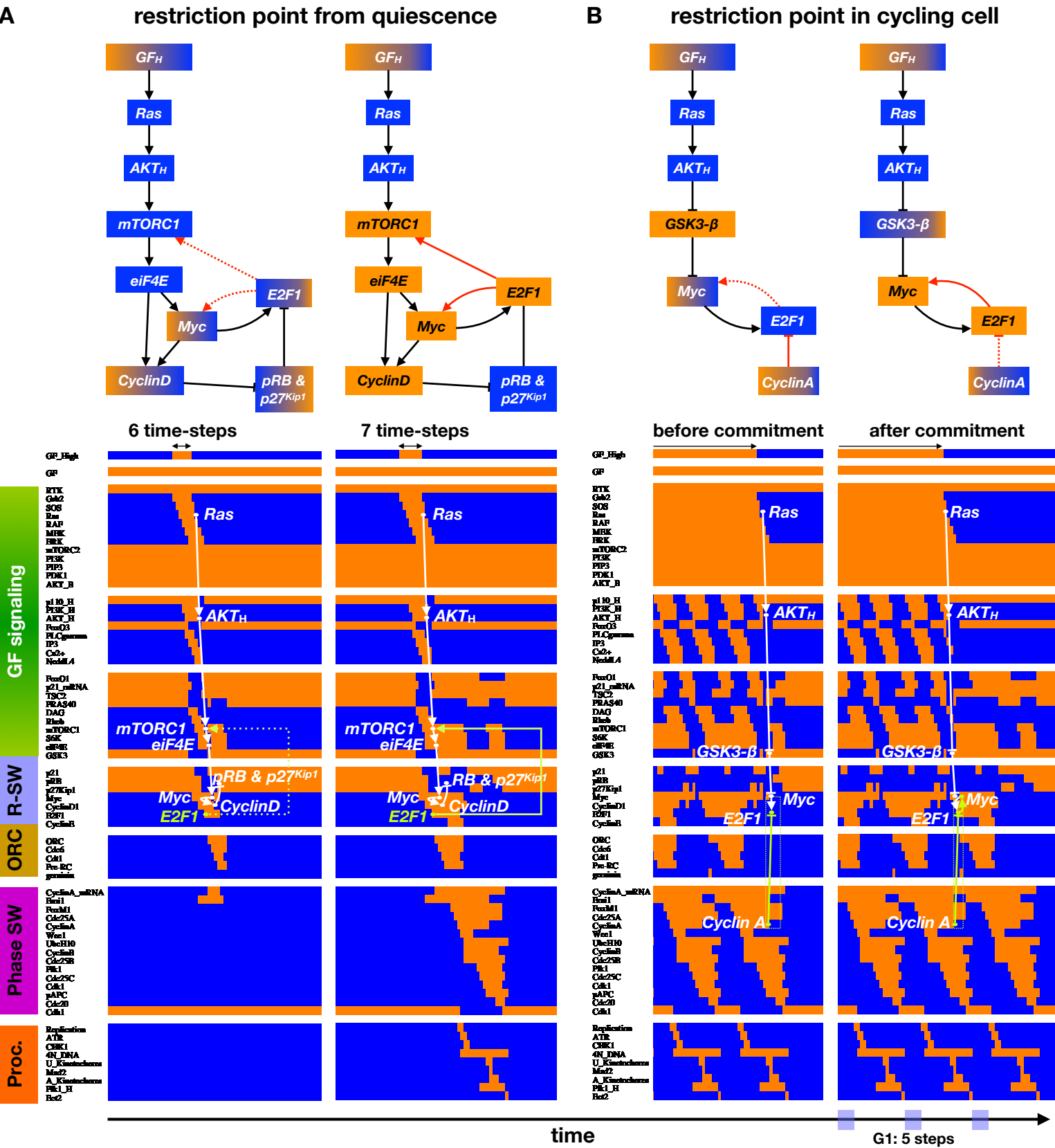

Supplement: S9 Fig — (A-B) Top: Molecular mechanism leading to cell cycle commitment in response to GFH, before and after restriction point passage in quiescent (A) and cycling (B) cells, showing the failure (left) or success (right) of locking in the Myc ⇆ E2F1 and Myc ⇆ mTORC1 feedback loops in (A), or the Myc ⇆ E2F1 loop in the presence/absence of GSK3-β and Cyclin A in (B). Orange/blue background: high/low activity; gradient background: nodes in transition; →: activation; ⊣: inhibition; solid/dashed red arrows: key interactions impacting / not yet impacting the outcome. Bottom: (A) Synchronous dynamics of regulatory molecule activity in response to 6 (left) or 7 (right) time-steps of high growth factor stimulation in quiescent cells. White arrows & nodes: factors driving cell cycle commitment in late G1; dashed / solid lime green arrow: lack of / presence of feedback from E2F1 to mTORC1. (B) Synchronous dynamics of regulatory molecule activity in response to high growth factor withdrawal cycling cells during G2, before (left) and after (right) pre-commitment to another division. X-axis: time-steps; y-axis: nodes of the model organized in modules (showing relevant modules); orange / blue: ON / OFF; white arrows & nodes: factors driving cell cycle commitment in late G2; dashed / solid lime green arrow: E2F1 inhibition (left) / lack of inhibition (right) by Cyclin A; only relevant module activity is shown shown (full dynamics available in S1 File). (PDF) [file pcbi.1006402.s009.pdf]

Supplementary Figure 10

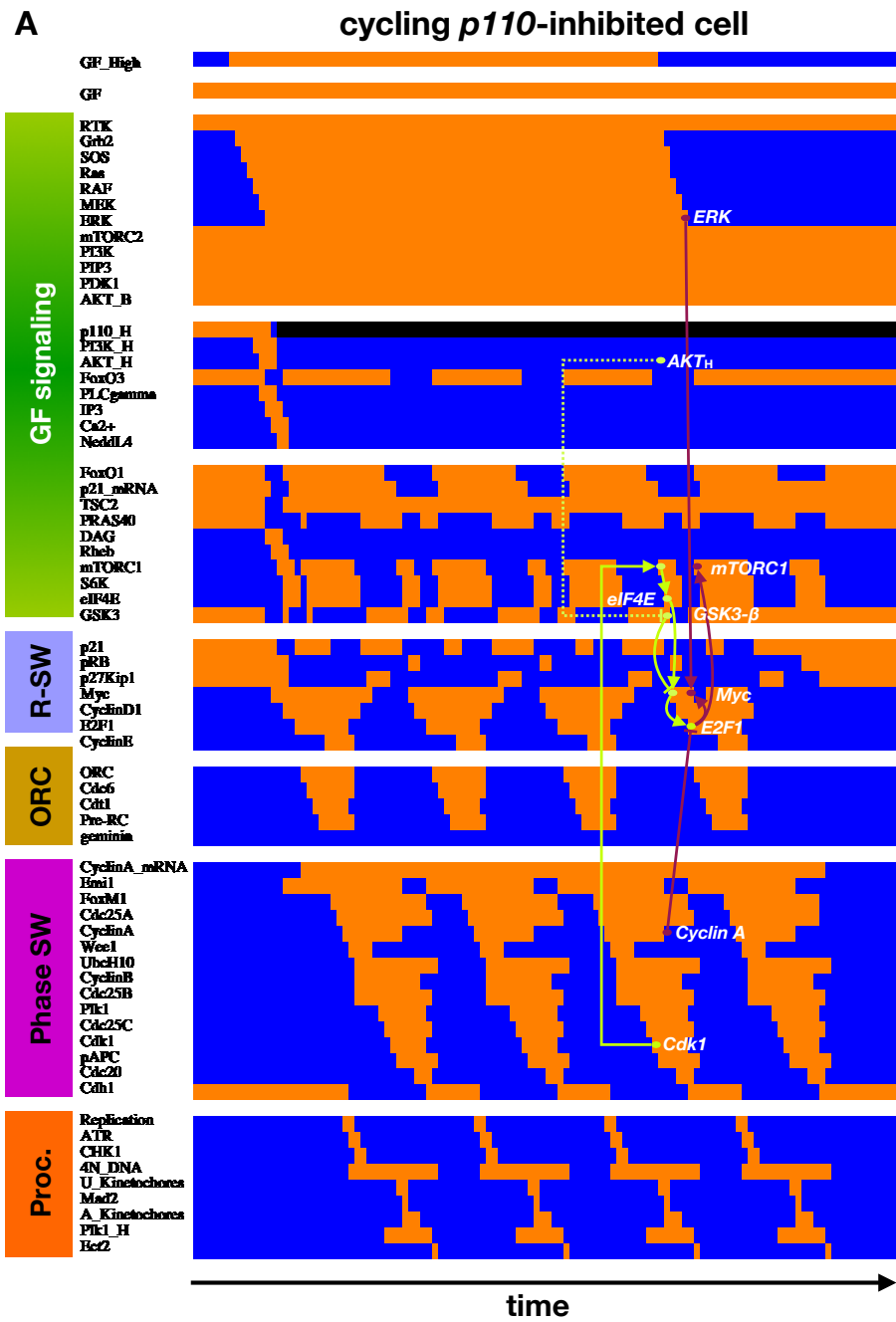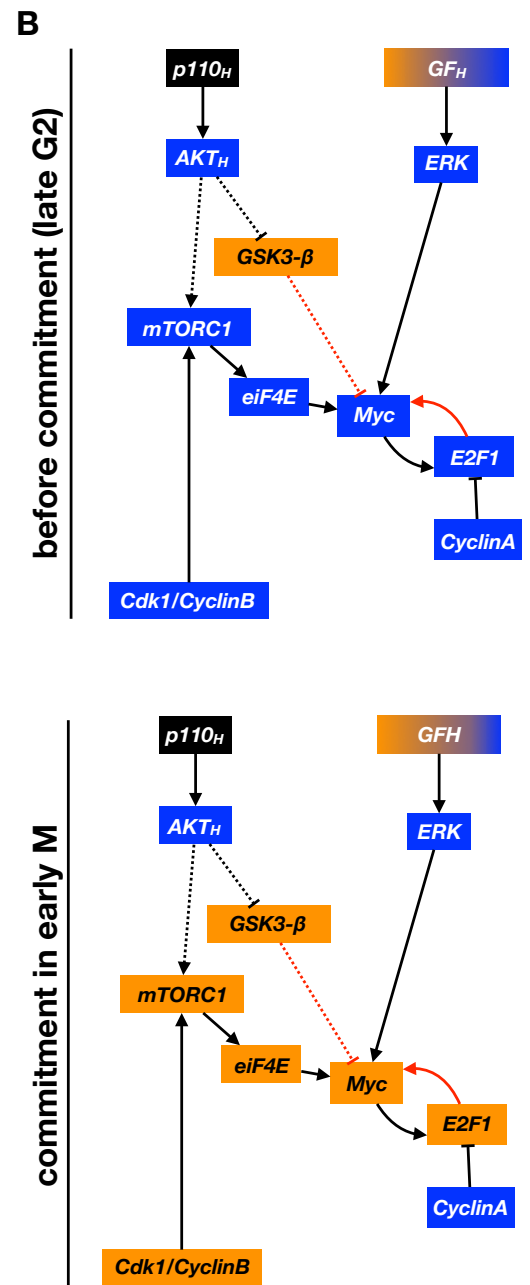

Supplement: S10 Fig — (A) Synchronous dynamics of regulatory molecule activity in response to p110H knockdown past the point of commitment from G0 to the first cycle. Lime green nodes & arrows: pre-commitment is not driven by E2F1 reactivation following Cyclin A degradation; rather, Cdk1/Cyclin B-mediated activation of mTORC1 → eIF4E is required to stabilize Myc in spite of the presence of GSK3β. Dark red nodes & arrows: in the absence of high AKT1, ERK is required for two additional time-steps compared to wild-type cells, in order to stabilize the E2F1 ⇄ Myc feedback loop; only relevant module activity is shown shown (full dynamics available in S1 File). (B) Molecular mechanism responsible for pre-commitment, before and after restriction point passage in prophase, showing the failure (top) or success (bottom) of locking in the Myc ⇆ E2F1 feedback loop in the absence/presence (top/bottom) of CyclinB/Cdk1-activated mTORC1 signaling. Black background: p110H inhibition; Orange/blue background: high/low activity; gradient background: nodes in transition; →: activation; ⊣: inhibition; solid/dashed arrows: key interactions impacting / not yet impacting the outcome. (PDF) [file pcbi.1006402.s010.pdf]

Supplementary Figure 12

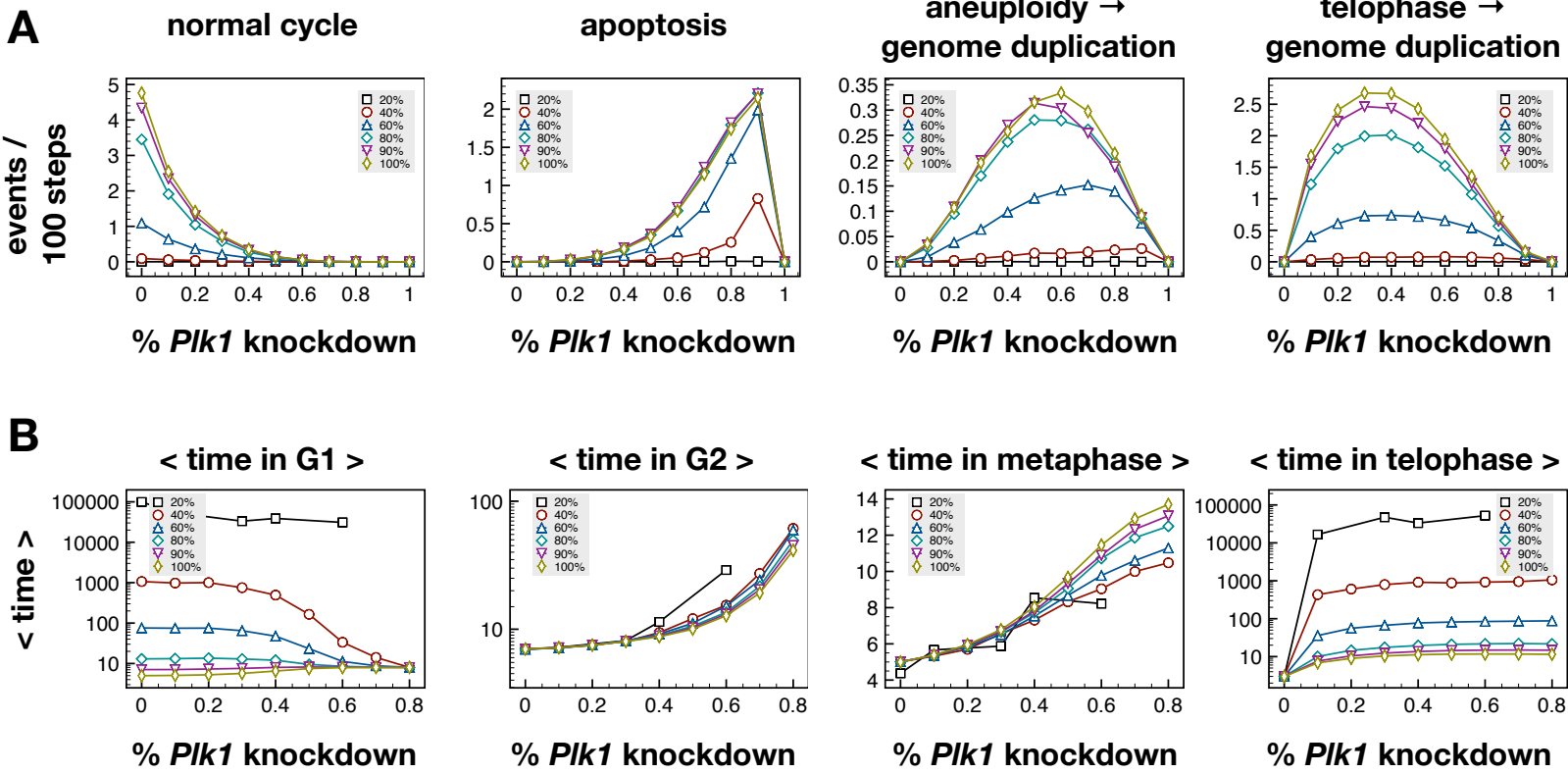

Supplement: S12 Fig — (A) Number of normal divisions (first panel), mitotic catastrophe (second panel), aberrant mitosis with genome doubling (third panel) and failed cytokinesis with genome doubling (fourth panel) per 100 time-steps as a function of Plk1 inhibition in varying growth environments (synchronous update). (B) Average time spent in G1 (first panel), G2 (second panel), metaphase (third panel) and telophase (binucleated cells in G1) (fourth panel) as a function of Plk1 inhibition in varying growth environments. pHigh_GF ∈ [20%, 40%, …, 100%]; sampling: 500,000 time-steps (synchronous update). (PDF) [file pcbi.1006402.s012.pdf]

Supplementary Figure 13

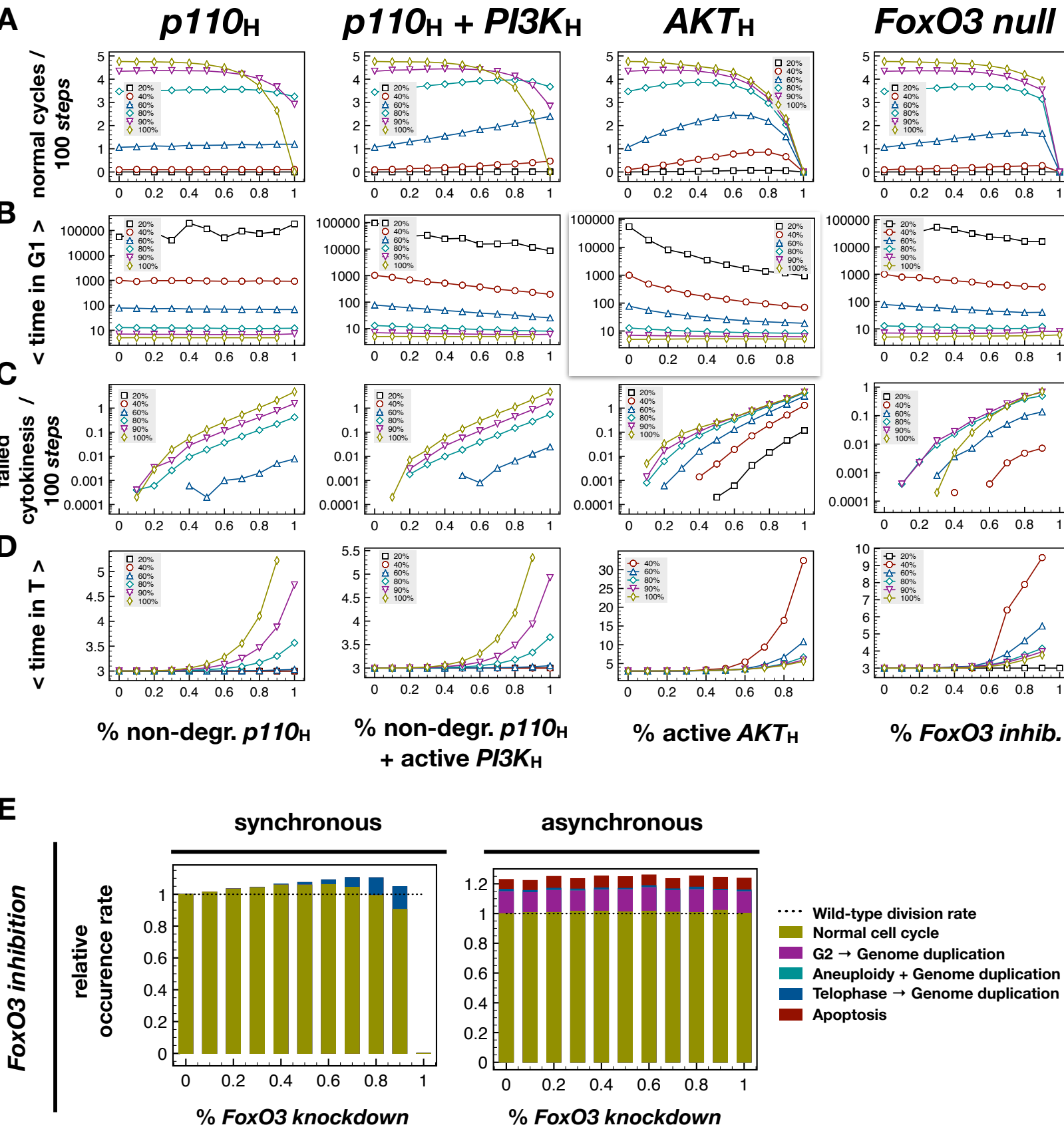

Supplement: S13 Fig — (A-D) Number of normal divisions competed in 100 time-steps (A), average G1 length (B), number of divisions with failed cytokinesis in 100 time-steps (C), and average telophase length (D) as a function of the rate of forced p110H (first panel), p110H + PI3KH (second panel), AKTH activation (third panel) and FoxO3 inhibition (fourth panel) in varying growth environments (synchronous update). (E) Stacked bar charts showing relative occurrence of normal cell cycle completion (mustard), G2 → G1 reset followed by genome duplication (purple), aberrant mitosis followed by genome duplication (turquoise), failed cytokinesis followed by genome duplication (blue) and apoptosis (dark red) as a function of FoxO3 inhibition, relative to the cell cycle rate in wild-type cells (black dashed line) at pHigh_GF = 80% modeled with synchronous (left) and biased order asynchronous update (right). Sampling: 50,000 time-steps. (PDF) [file pcbi.1006402.s013.pdf]

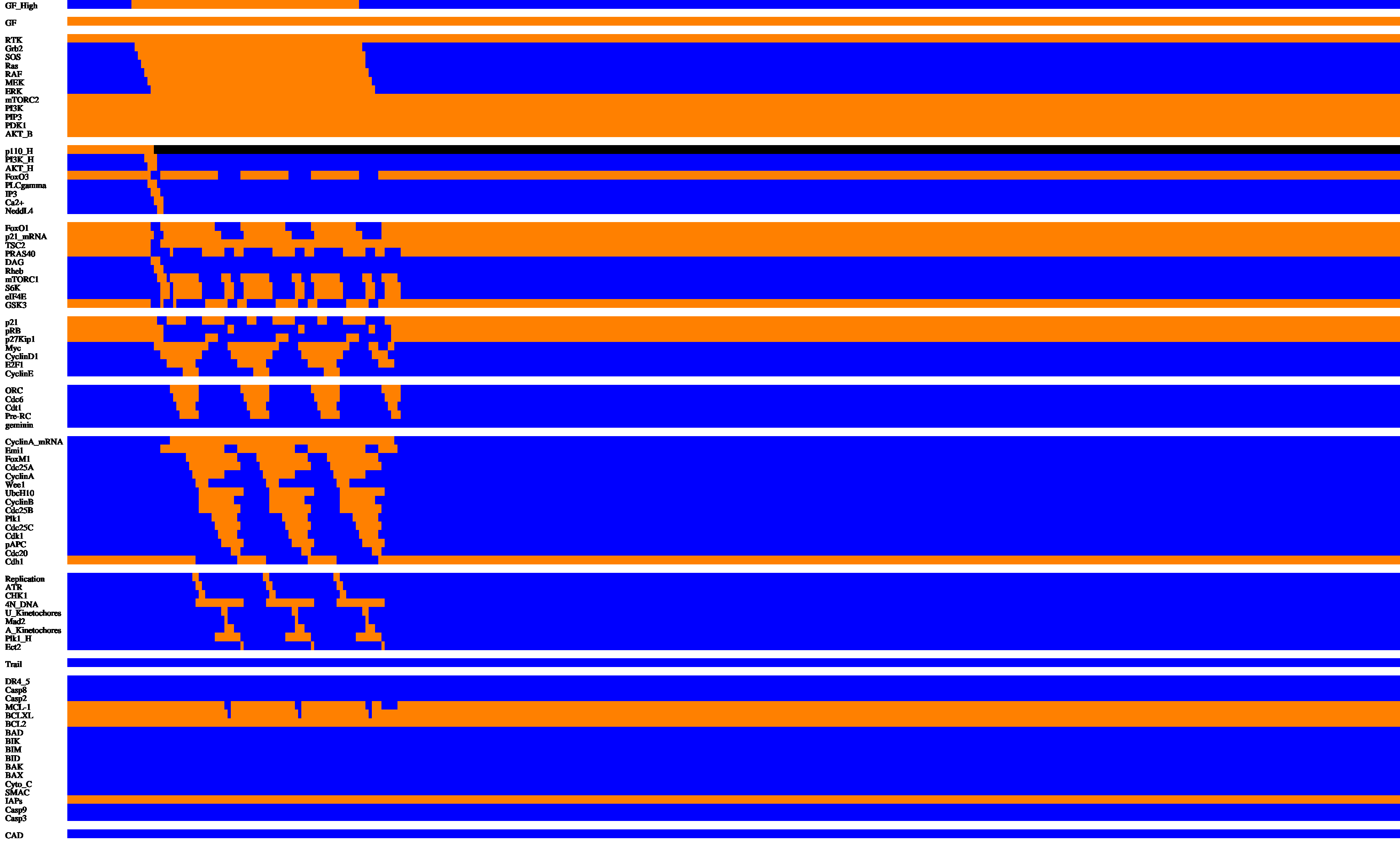

Supplement: S1 File — Full dynamics of the model for simulations shown in a truncated form on Figs 6, 8, S3, S8, S9 and S10; additional simulations mentioned in Tables 1 and 2 but not included on the figures. (ZIP) [file pcbi.1006402.s019.zip › S1_File/SFig_10 - 1_p110_H-KO__GF_High_Pulse_in_CC_ending_before_pre-commitment.pdf]

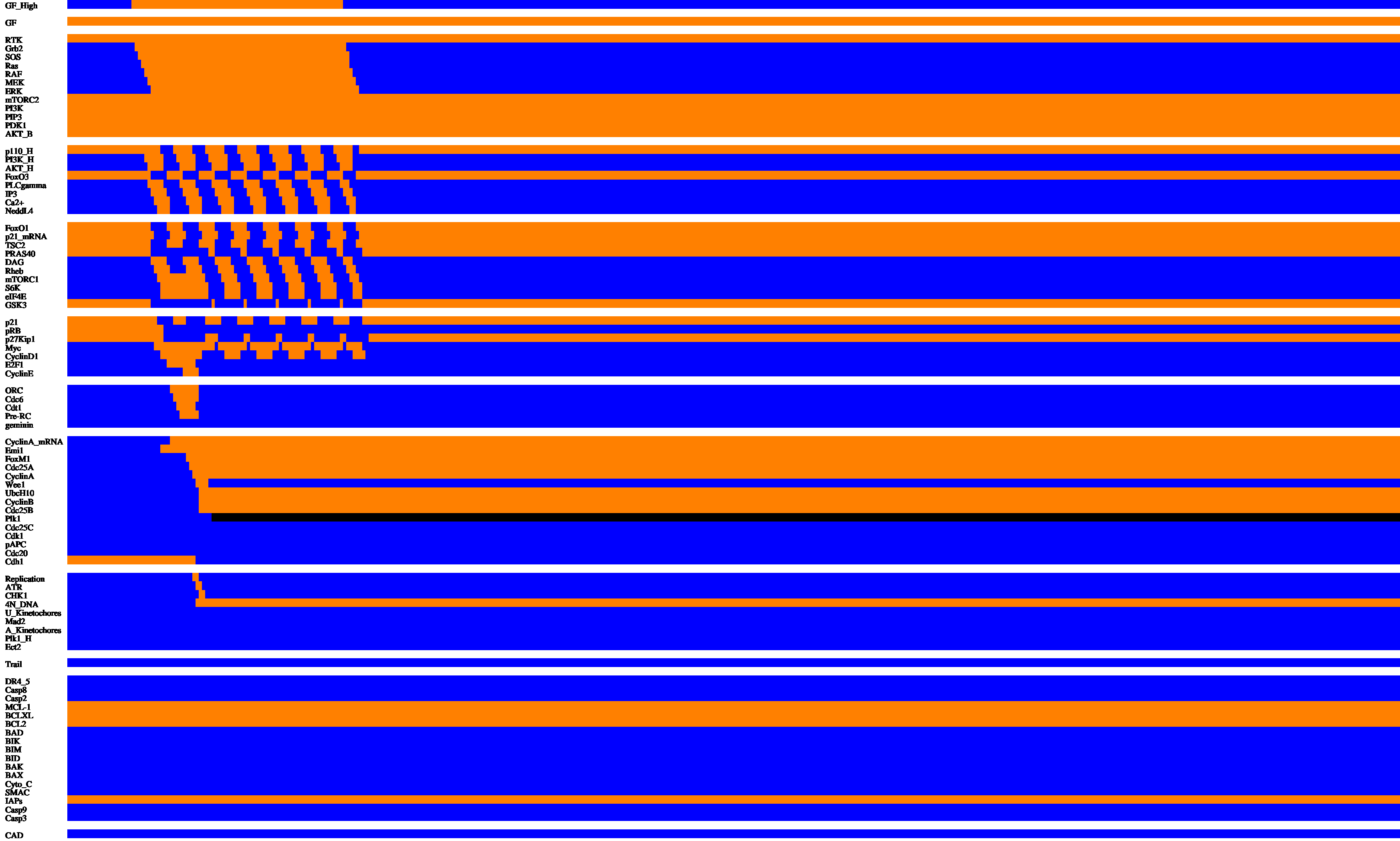

Supplement: S1 File — Full dynamics of the model for simulations shown in a truncated form on Figs 6, 8, S3, S8, S9 and S10; additional simulations mentioned in Tables 1 and 2 but not included on the figures. (ZIP) [file pcbi.1006402.s019.zip › S1_File/Fig_6A - Plk1-KO_in_G2.pdf]

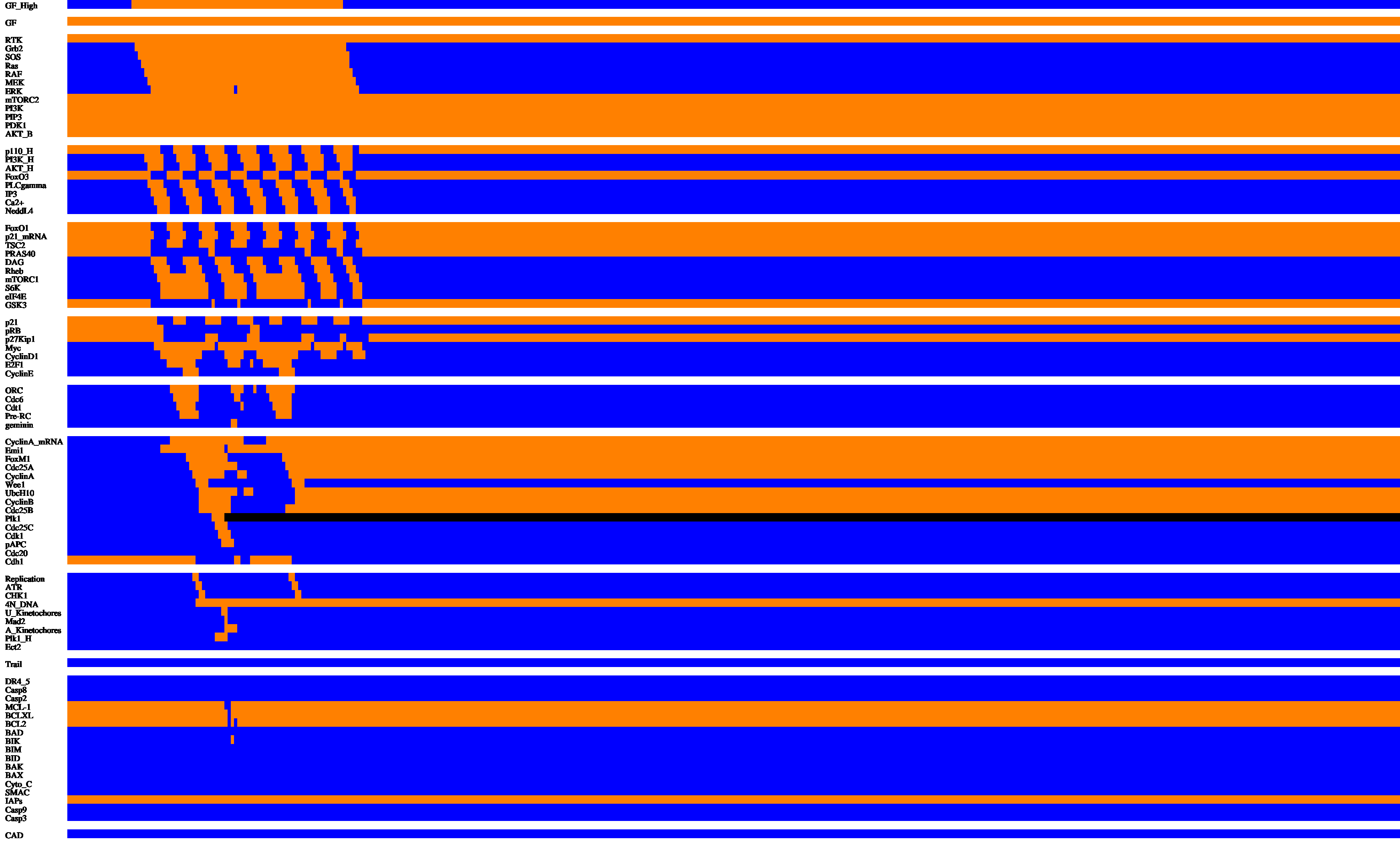

Supplement: S1 File — Full dynamics of the model for simulations shown in a truncated form on Figs 6, 8, S3, S8, S9 and S10; additional simulations mentioned in Tables 1 and 2 but not included on the figures. (ZIP) [file pcbi.1006402.s019.zip › S1_File/Fig_6D - Plk1-KO_at_SAC.pdf]

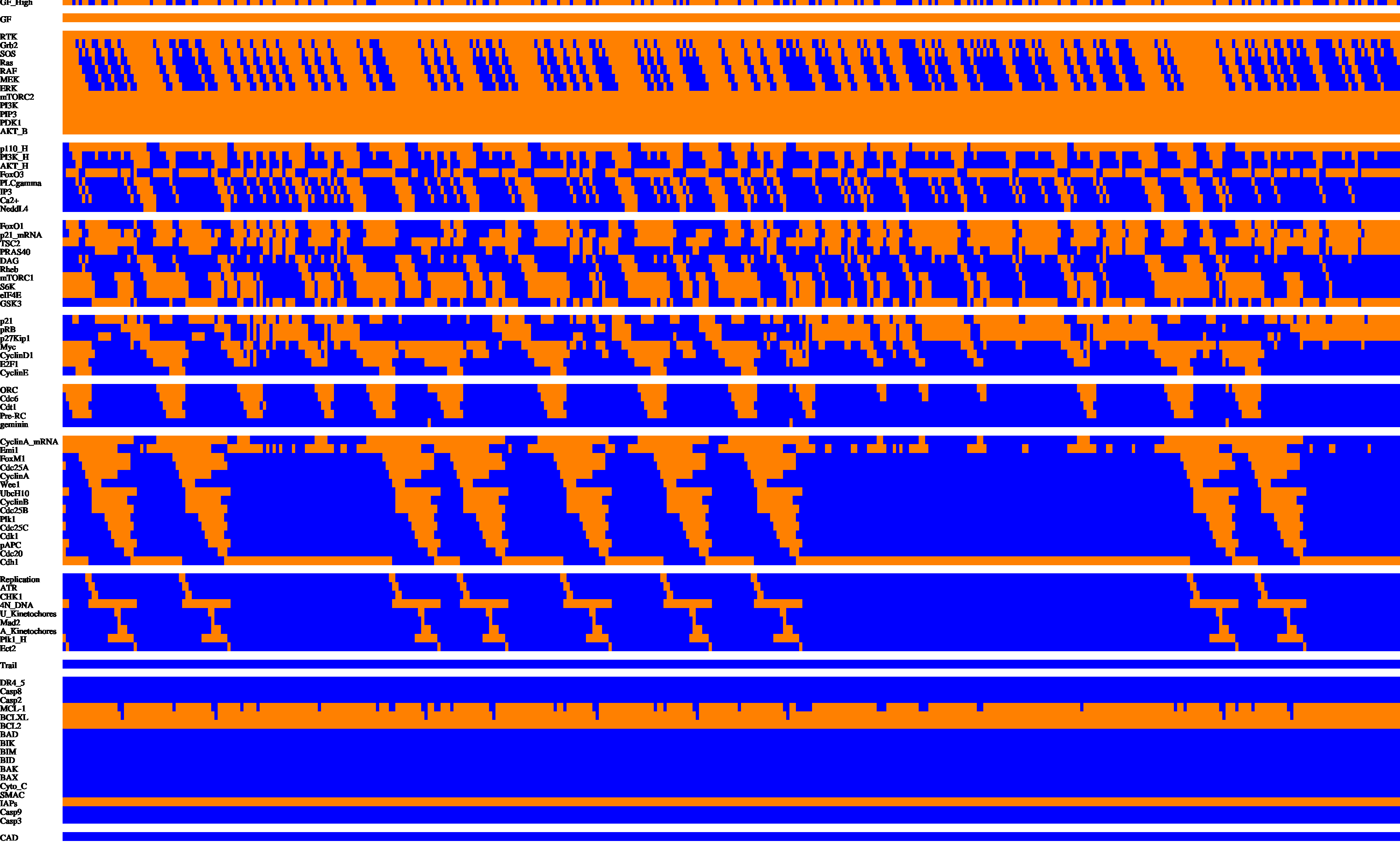

Supplement: S1 File — Full dynamics of the model for simulations shown in a truncated form on Figs 6, 8, S3, S8, S9 and S10; additional simulations mentioned in Tables 1 and 2 but not included on the figures. (ZIP) [file pcbi.1006402.s019.zip › S1_File/3 - Stochastic_GF_High-0.70__Intermittent_Cycles.pdf]

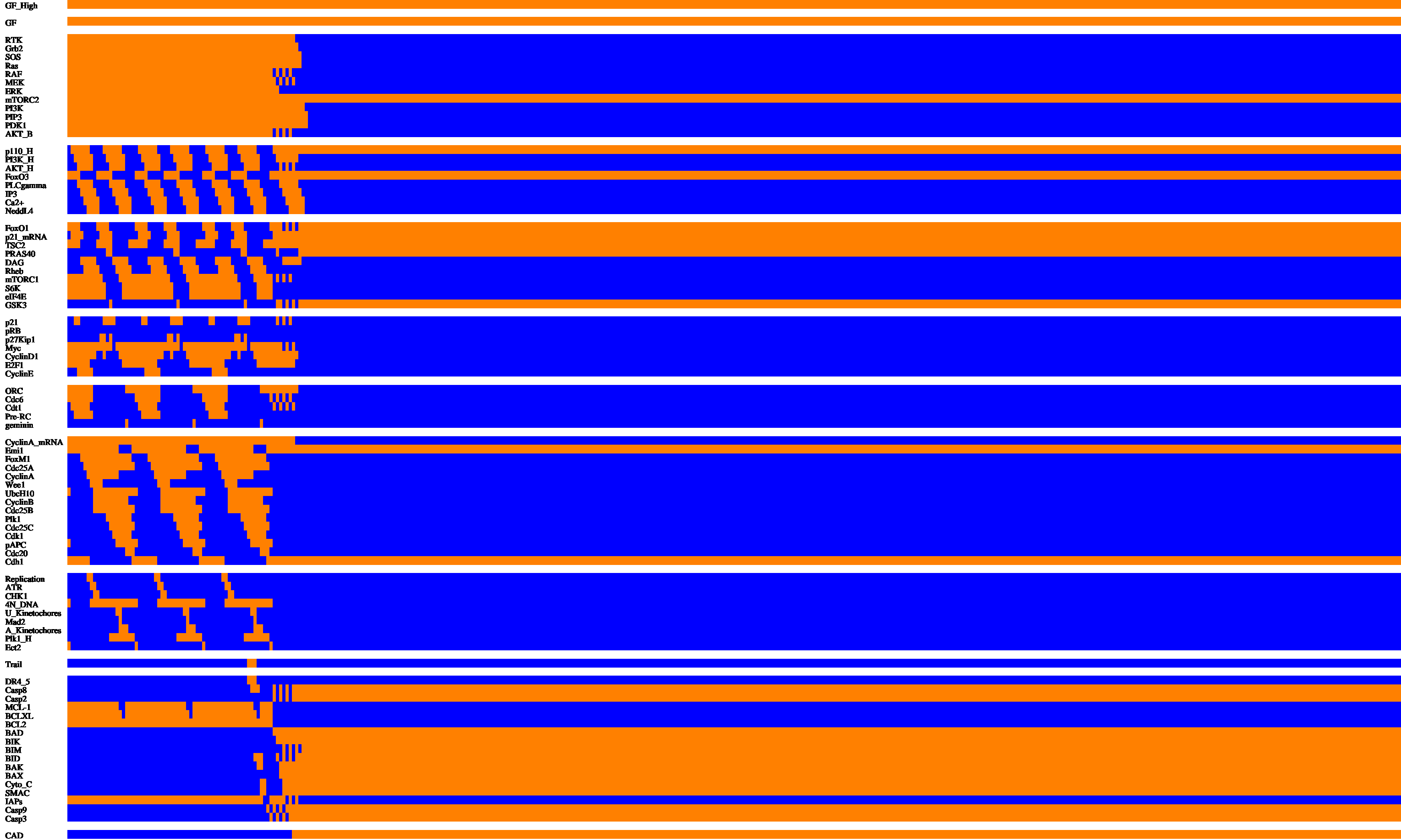

Supplement: S1 File — Full dynamics of the model for simulations shown in a truncated form on Figs 6, 8, S3, S8, S9 and S10; additional simulations mentioned in Tables 1 and 2 but not included on the figures. (ZIP) [file pcbi.1006402.s019.zip › S1_File/SFig_3A - 2_Trail__Apoptosis_Pulse-3_timesteps__Metaphase.pdf]

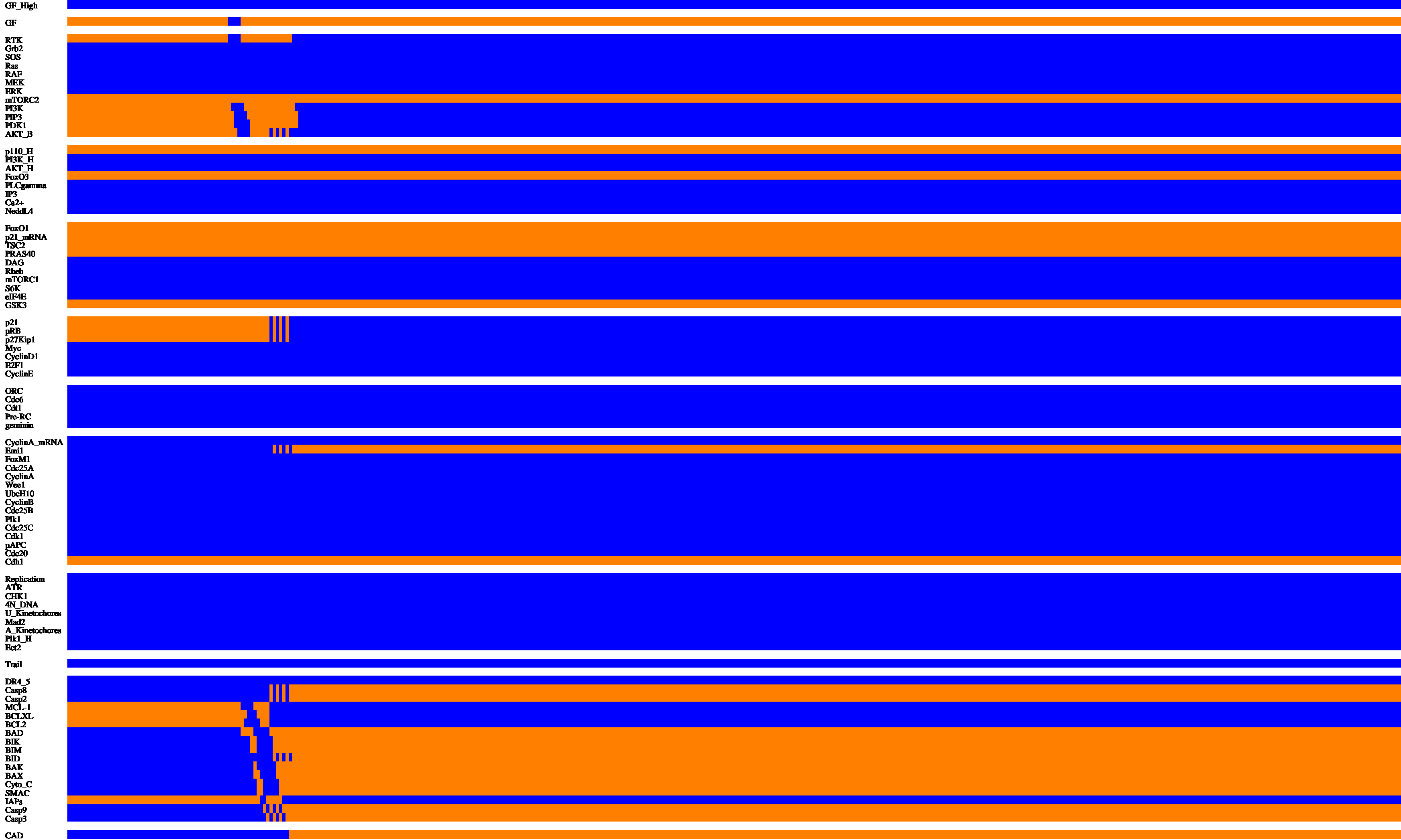

Supplement: S1 File — Full dynamics of the model for simulations shown in a truncated form on Figs 6, 8, S3, S8, S9 and S10; additional simulations mentioned in Tables 1 and 2 but not included on the figures. (ZIP) [file pcbi.1006402.s019.zip › S1_File/2 - GF-loss__Apoptosis_Pulse-4_timesteps__quiescent_cell.pdf]

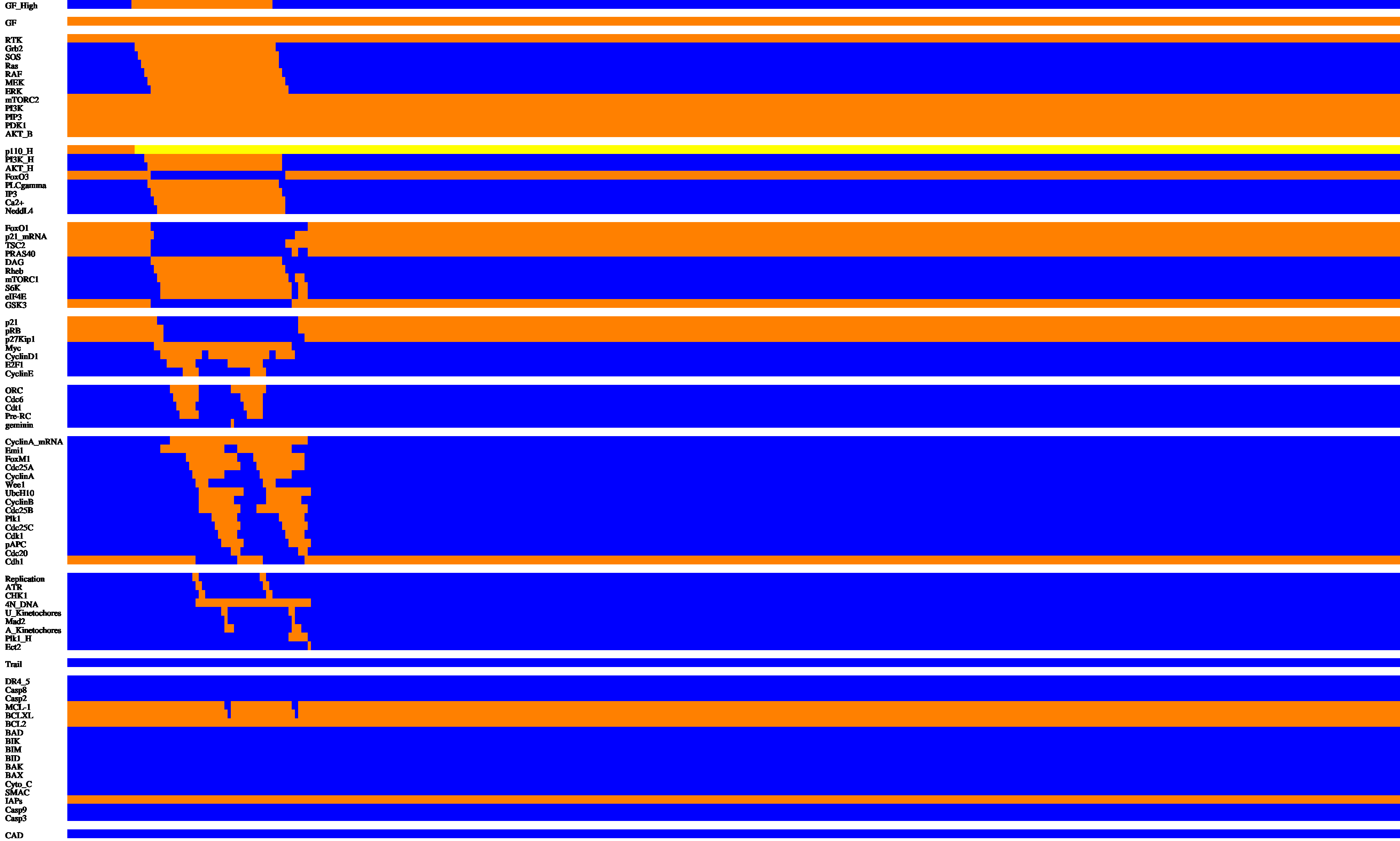

Supplement: S1 File — Full dynamics of the model for simulations shown in a truncated form on Figs 6, 8, S3, S8, S9 and S10; additional simulations mentioned in Tables 1 and 2 but not included on the figures. (ZIP) [file pcbi.1006402.s019.zip › S1_File/Fig_8A - p110_H-OE_failed_cytokinesis.pdf]

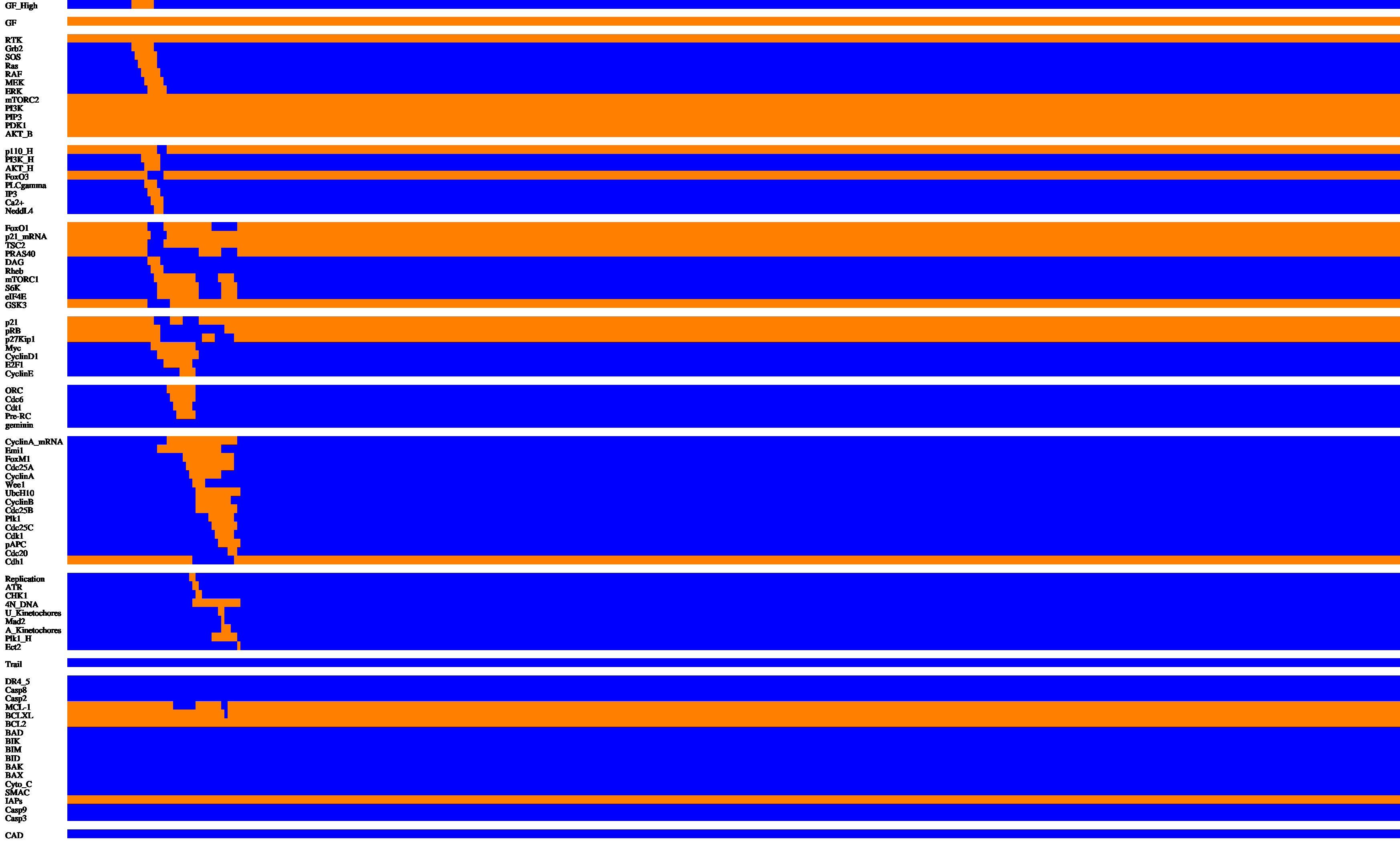

Supplement: S1 File — Full dynamics of the model for simulations shown in a truncated form on Figs 6, 8, S3, S8, S9 and S10; additional simulations mentioned in Tables 1 and 2 but not included on the figures. (ZIP) [file pcbi.1006402.s019.zip › S1_File/SFig_9A - 2_GF_High_Pulse_7_steps_in_G0_after_R-point.pdf]

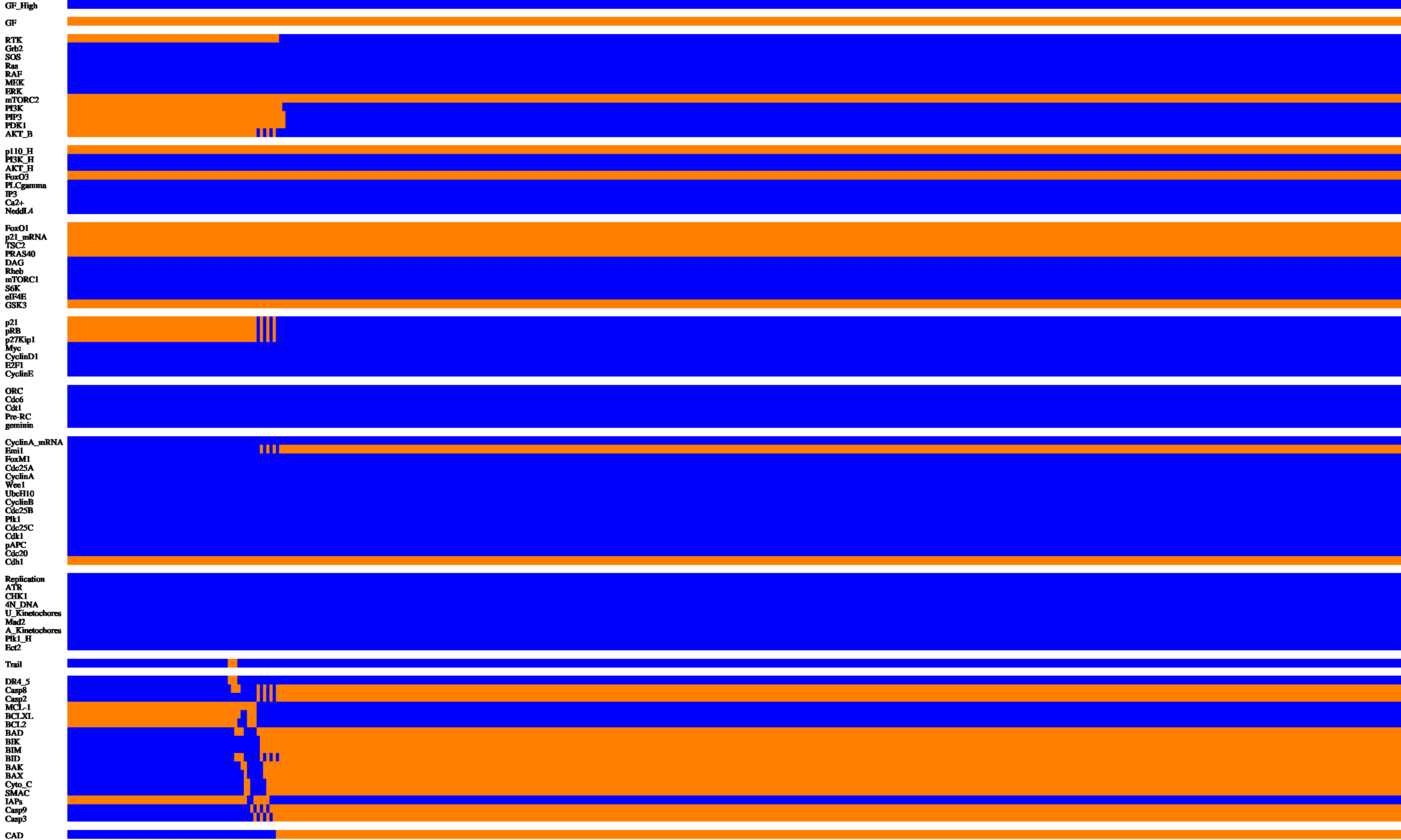

Supplement: S1 File — Full dynamics of the model for simulations shown in a truncated form on Figs 6, 8, S3, S8, S9 and S10; additional simulations mentioned in Tables 1 and 2 but not included on the figures. (ZIP) [file pcbi.1006402.s019.zip › S1_File/1 - Trail__Apoptosis_Pulse-3_timesteps__quiescent_cell.pdf.pdf]

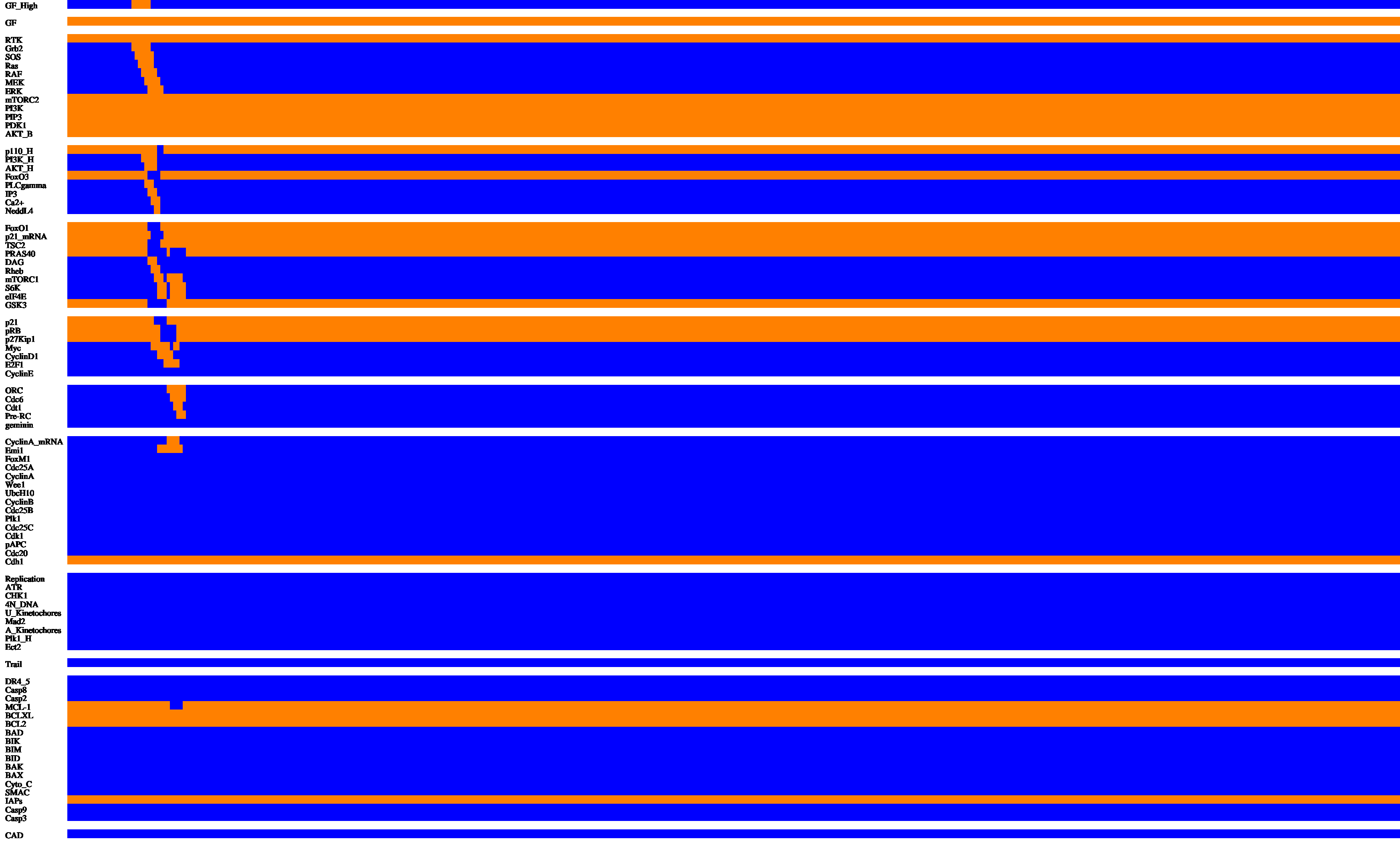

Supplement: S1 File — Full dynamics of the model for simulations shown in a truncated form on Figs 6, 8, S3, S8, S9 and S10; additional simulations mentioned in Tables 1 and 2 but not included on the figures. (ZIP) [file pcbi.1006402.s019.zip › S1_File/SFig_9A - 1_GF_High_Pulse_6_steps_in_G0_before_R-point.pdf]

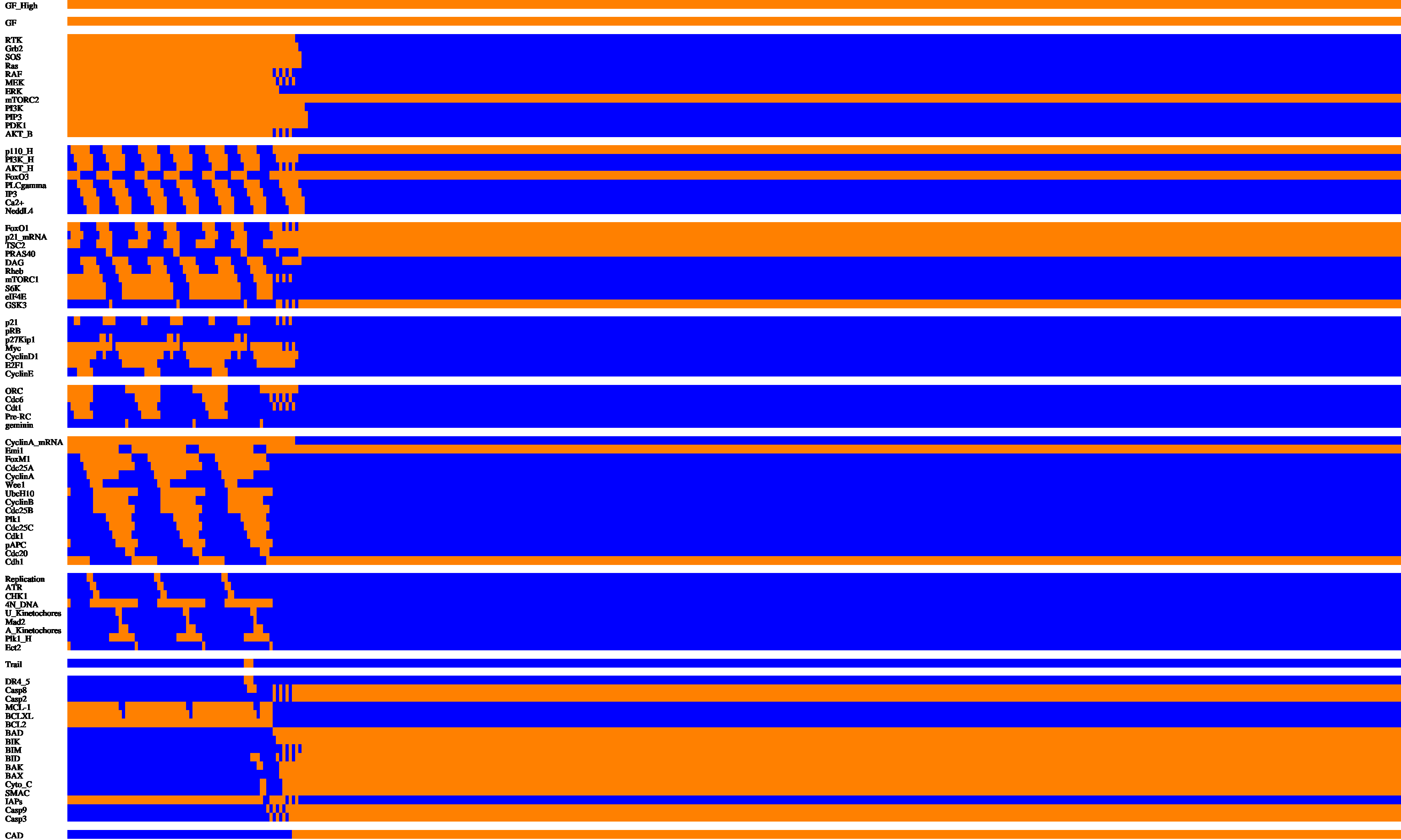

Supplement: S1 File — Full dynamics of the model for simulations shown in a truncated form on Figs 6, 8, S3, S8, S9 and S10; additional simulations mentioned in Tables 1 and 2 but not included on the figures. (ZIP) [file pcbi.1006402.s019.zip › S1_File/SFig_3A - 1_Trail__Apoptosis_Pulse-3_timesteps__before_Metaphase.pdf]

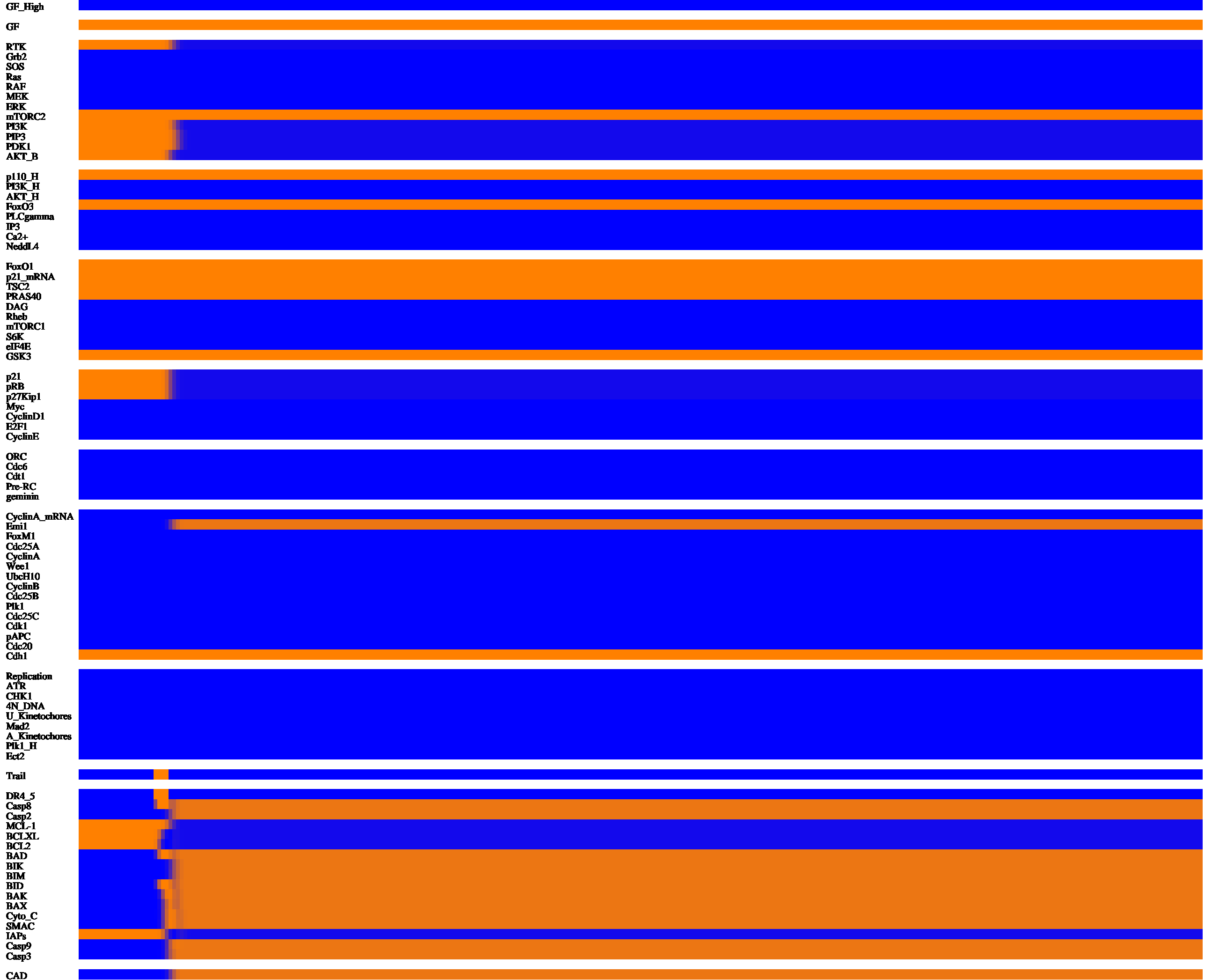

Supplement: S1 File — Full dynamics of the model for simulations shown in a truncated form on Figs 6, 8, S3, S8, S9 and S10; additional simulations mentioned in Tables 1 and 2 but not included on the figures. (ZIP) [file pcbi.1006402.s019.zip › S1_File/SFig_3B - Trail__Apoptosis_Pulse-4_timesteps__Asynchronous_quiescent_cell.pdf]

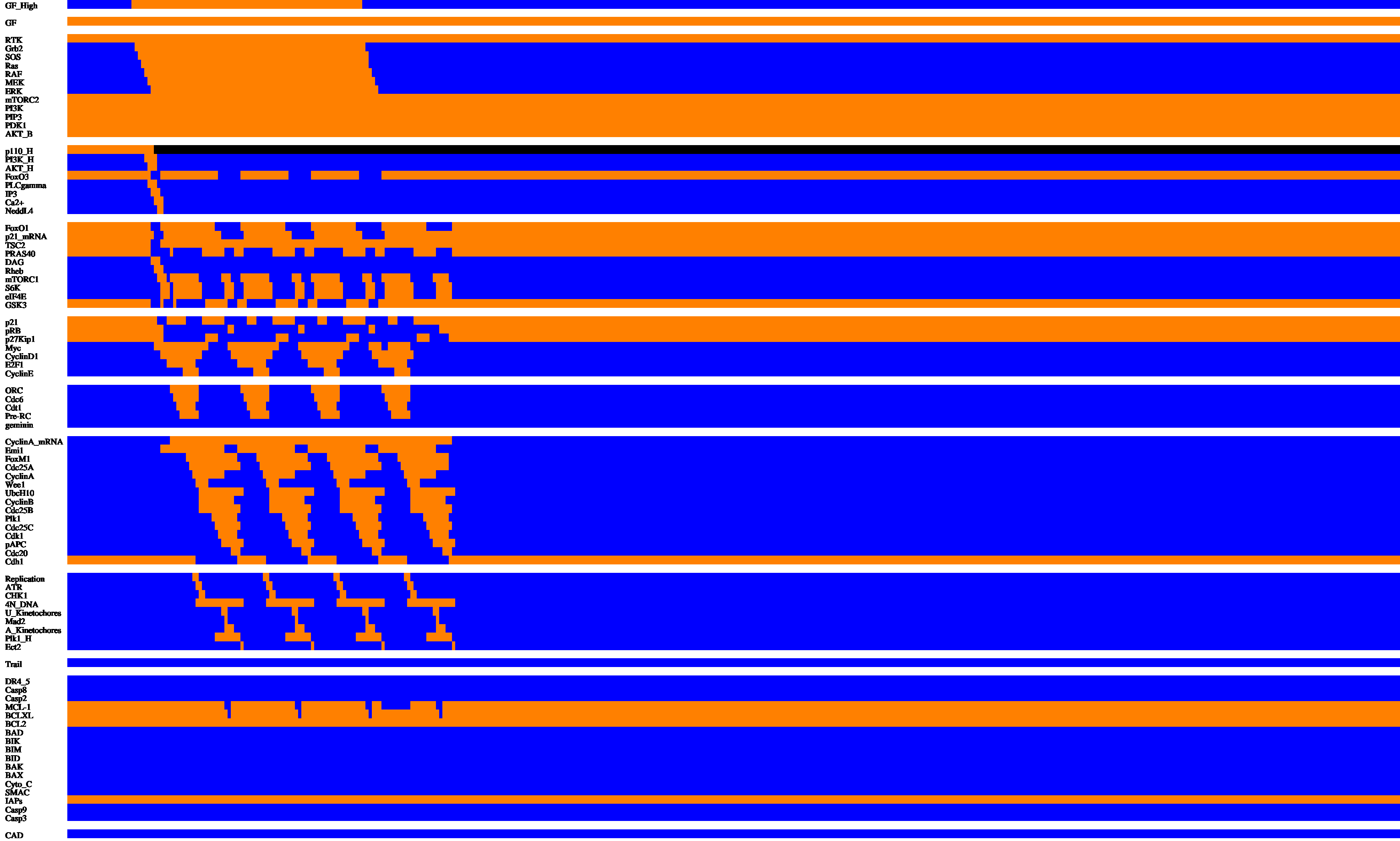

Supplement: S1 File — Full dynamics of the model for simulations shown in a truncated form on Figs 6, 8, S3, S8, S9 and S10; additional simulations mentioned in Tables 1 and 2 but not included on the figures. (ZIP) [file pcbi.1006402.s019.zip › S1_File/SFig_10 - 2_p110_H-KO__GF_High_Pulse_in_CC_ending_after_pre-commitment.pdf]

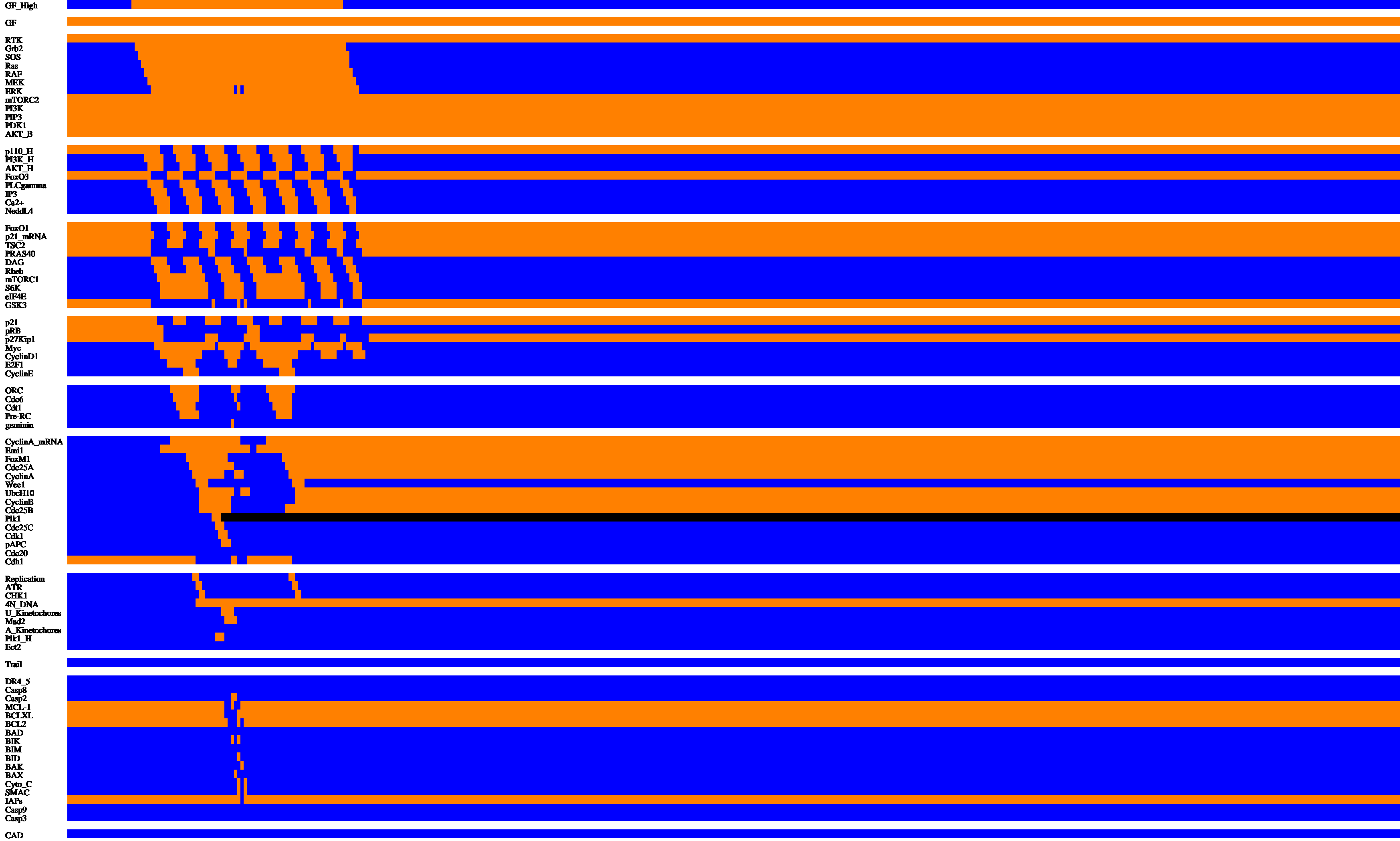

Supplement: S1 File — Full dynamics of the model for simulations shown in a truncated form on Figs 6, 8, S3, S8, S9 and S10; additional simulations mentioned in Tables 1 and 2 but not included on the figures. (ZIP) [file pcbi.1006402.s019.zip › S1_File/Fig_6C - Plk1-KO_in_Metaphase.pdf]

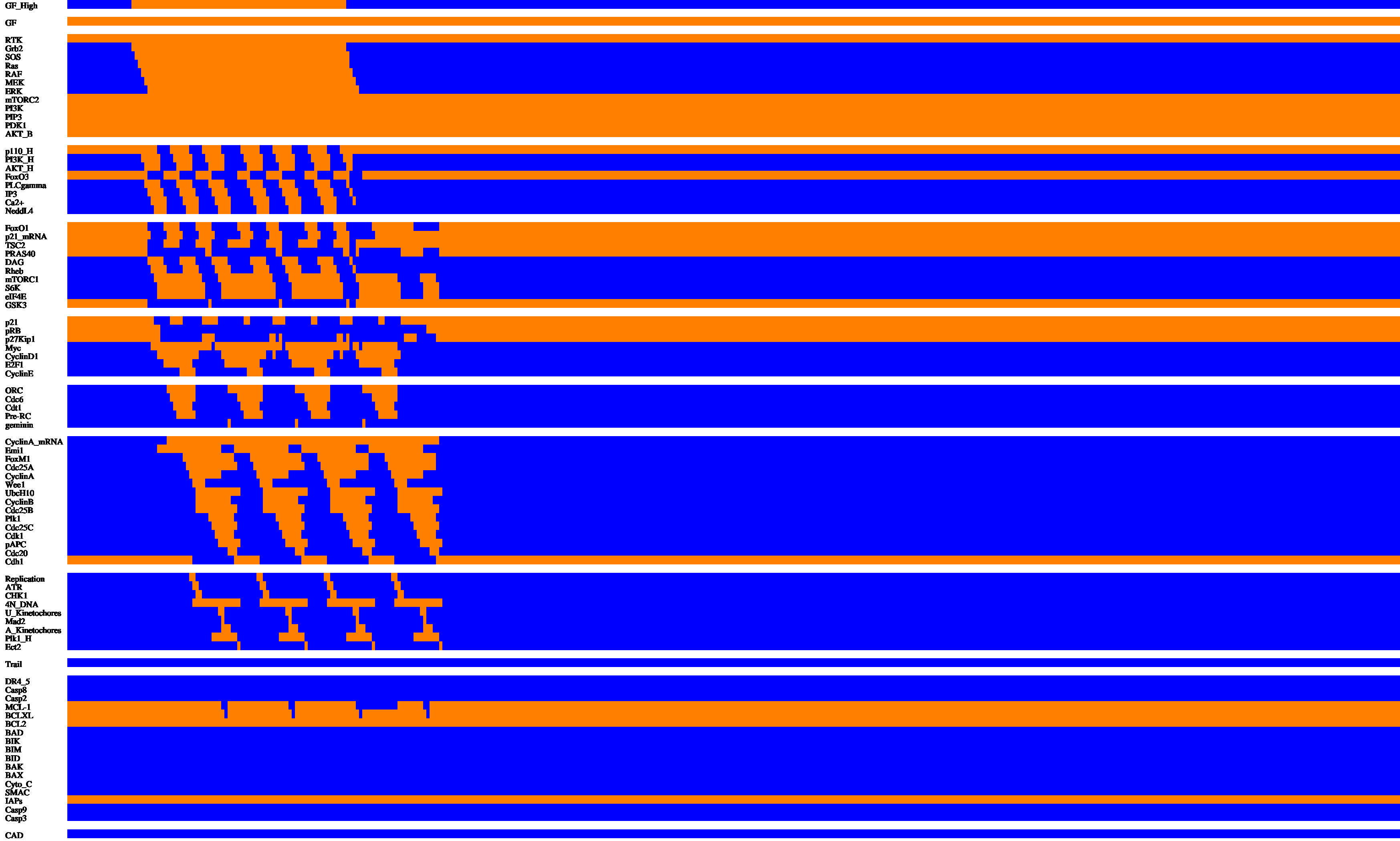

Supplement: S1 File — Full dynamics of the model for simulations shown in a truncated form on Figs 6, 8, S3, S8, S9 and S10; additional simulations mentioned in Tables 1 and 2 but not included on the figures. (ZIP) [file pcbi.1006402.s019.zip › S1_File/SFig_9B - 2_GF_High_Pulse_in_CC_ending_after_pre-commitment.pdf]

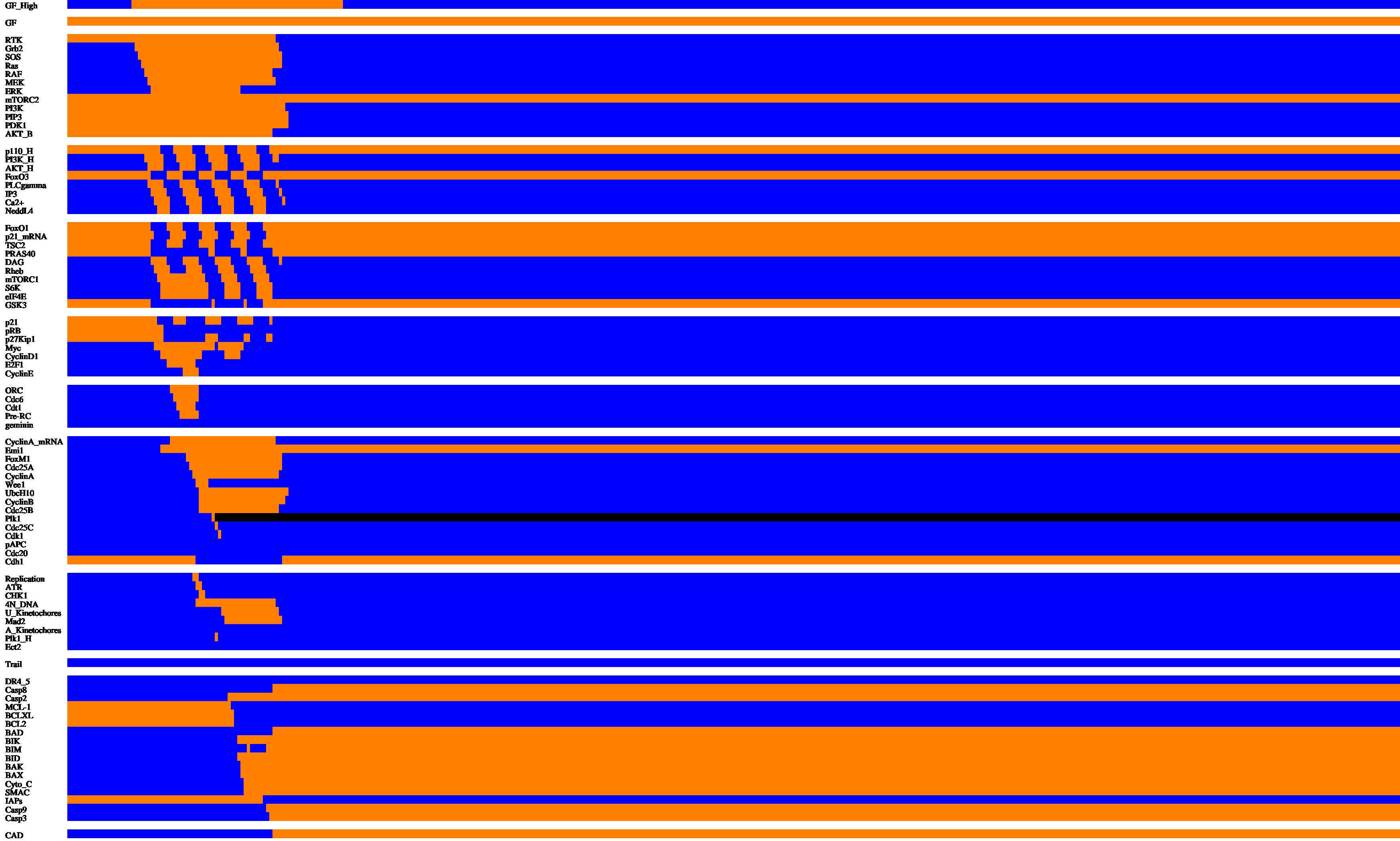

Supplement: S1 File — Full dynamics of the model for simulations shown in a truncated form on Figs 6, 8, S3, S8, S9 and S10; additional simulations mentioned in Tables 1 and 2 but not included on the figures. (ZIP) [file pcbi.1006402.s019.zip › S1_File/Fig_6B - Plk1-KO_in_ProMetaphase.pdf]

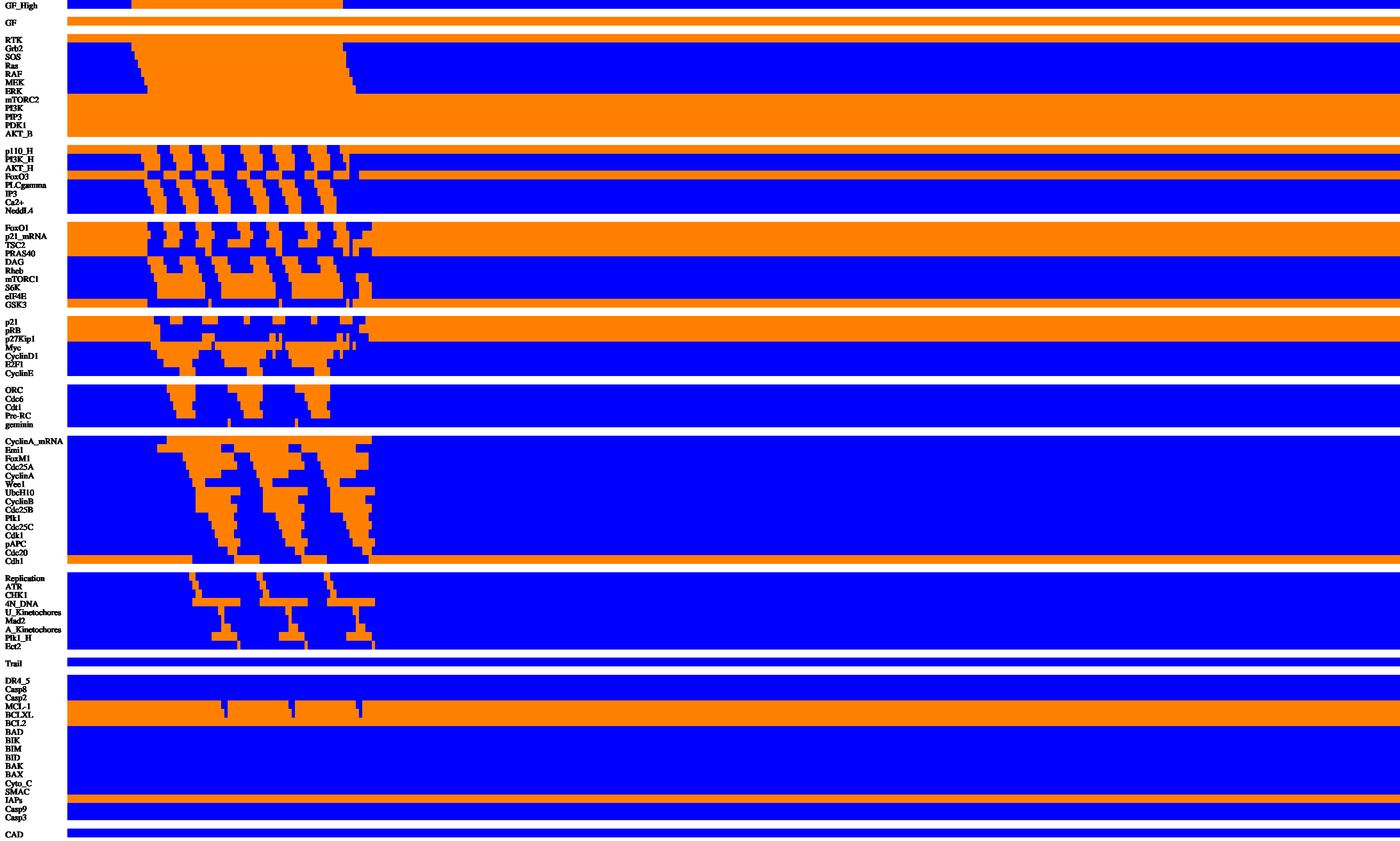

Supplement: S1 File — Full dynamics of the model for simulations shown in a truncated form on Figs 6, 8, S3, S8, S9 and S10; additional simulations mentioned in Tables 1 and 2 but not included on the figures. (ZIP) [file pcbi.1006402.s019.zip › S1_File/SFig_9B - 1_GF_High_Pulse_in_CC_ending_before_pre-commitment.pdf]

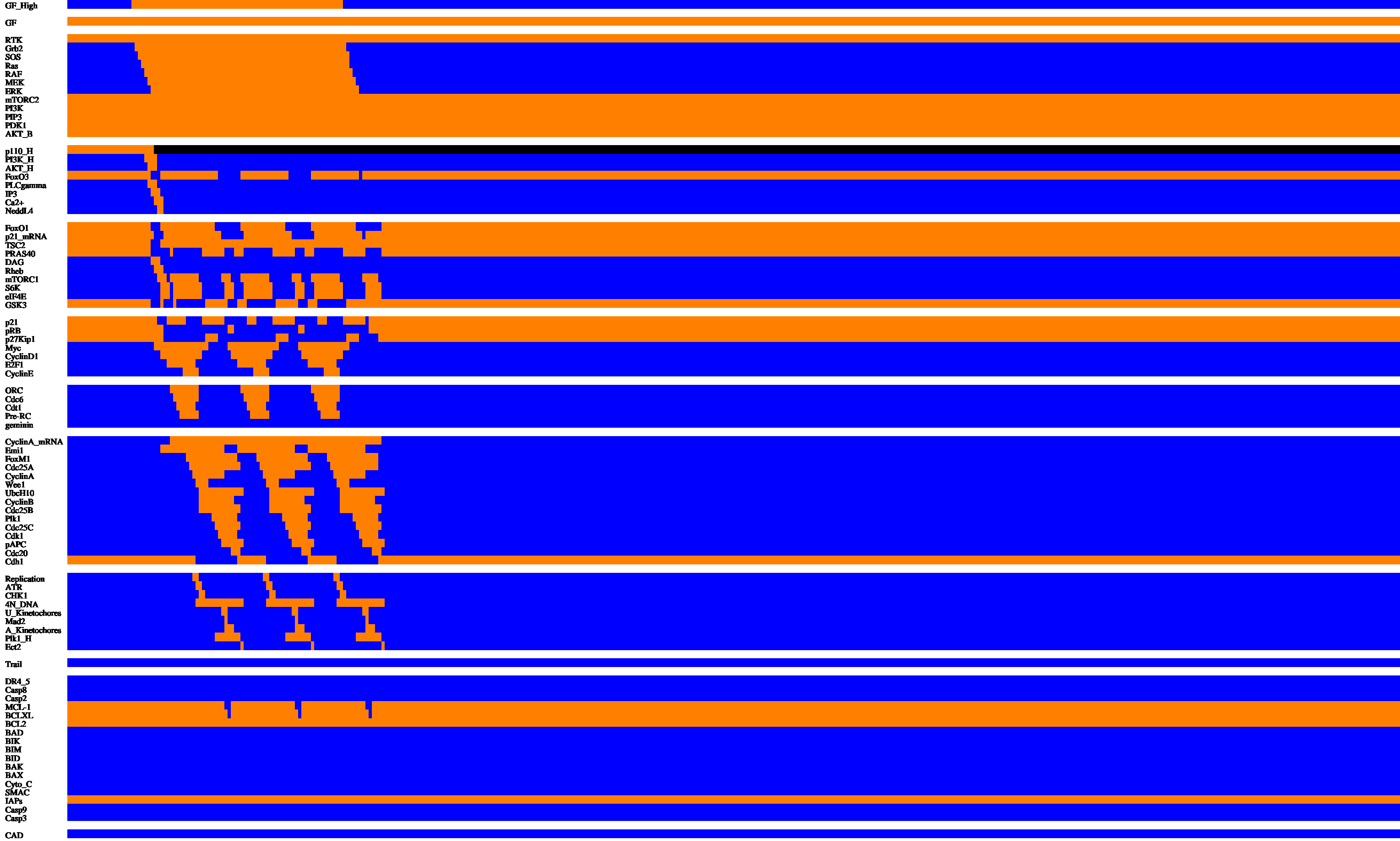

Supplement: S1 File — Full dynamics of the model for simulations shown in a truncated form on Figs 6, 8, S3, S8, S9 and S10; additional simulations mentioned in Tables 1 and 2 but not included on the figures. (ZIP) [file pcbi.1006402.s019.zip › S1_File/SFig_8A - 2_p110_H-KO_after_CyclinD_activation.pdf]

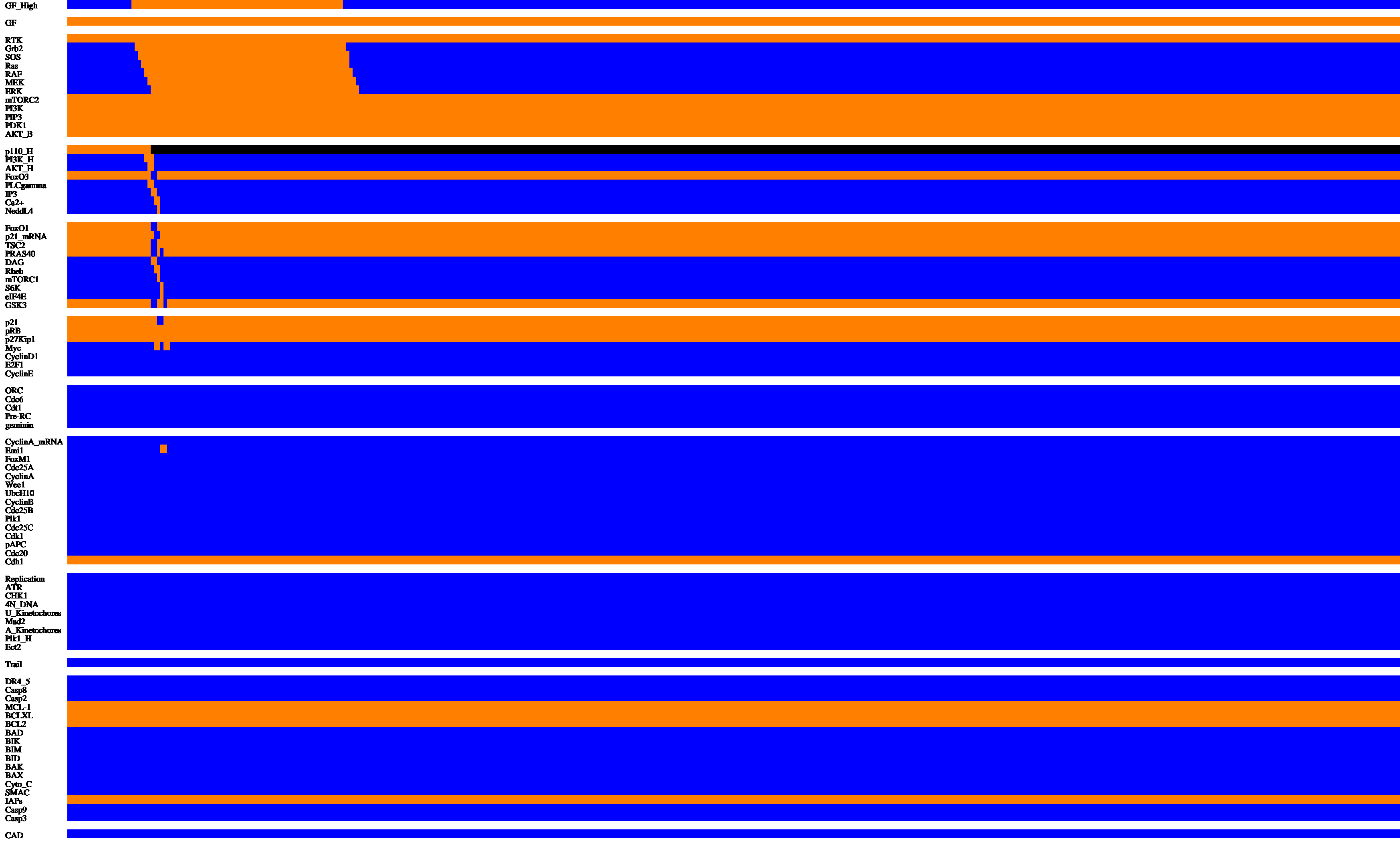

Supplement: S1 File — Full dynamics of the model for simulations shown in a truncated form on Figs 6, 8, S3, S8, S9 and S10; additional simulations mentioned in Tables 1 and 2 but not included on the figures. (ZIP) [file pcbi.1006402.s019.zip › S1_File/SFig_8A - 1_p110_H-KO_before_CyclinD_activation.pdf]

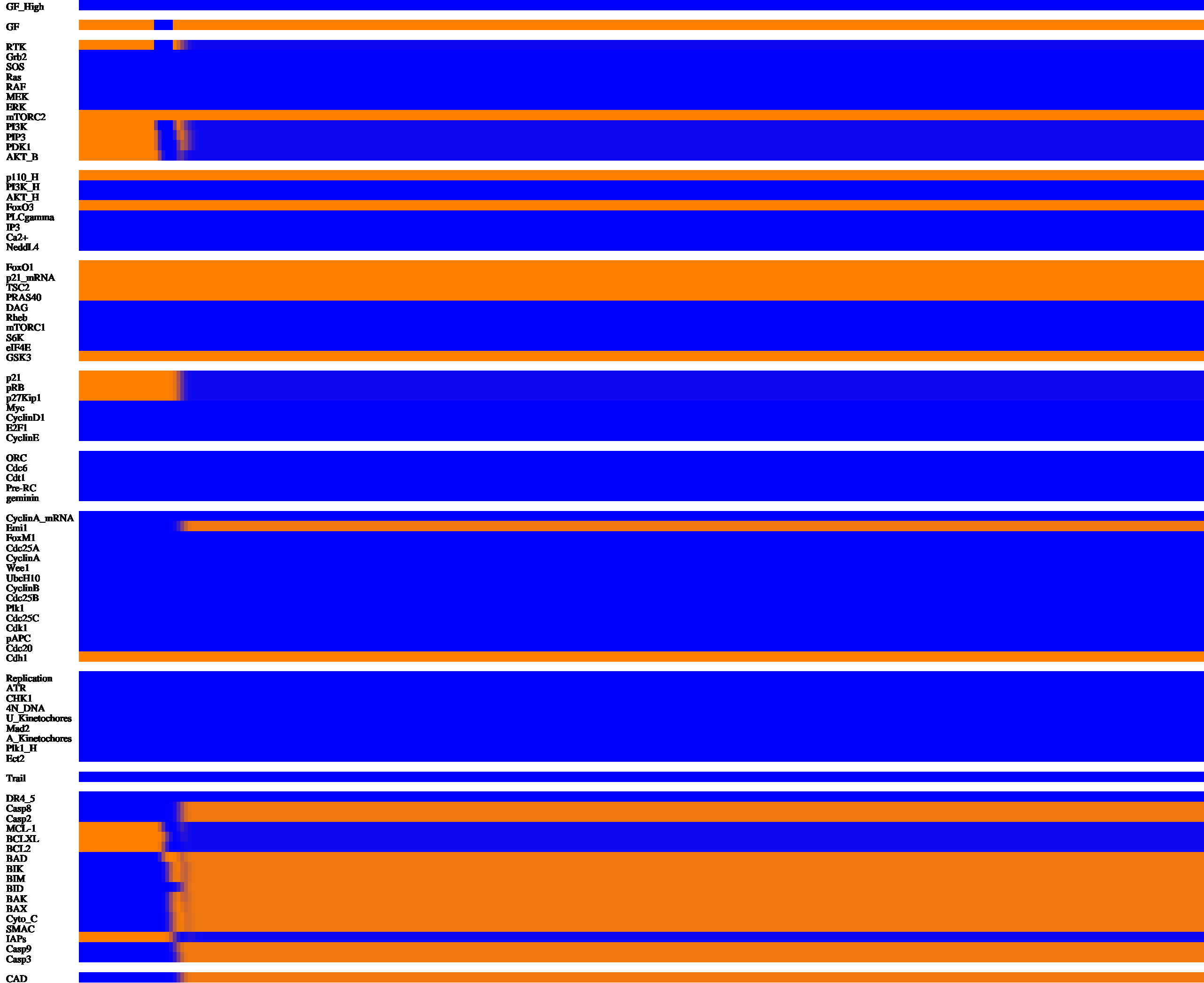

Supplement: S1 File — Full dynamics of the model for simulations shown in a truncated form on Figs 6, 8, S3, S8, S9 and S10; additional simulations mentioned in Tables 1 and 2 but not included on the figures. (ZIP) [file pcbi.1006402.s019.zip › S1_File/SFig_3C - GF-loss__Apoptosis_Pulse-5_timesteps__Asynchronous_quiescent_cell.pdf]
