# Supplementary material for: Boolean model of growth signaling, cell cycle and apoptosis predicts the molecular mechanism of aberrant cell cycle progression driven by hyperactive PI3K
Source: PLoS Comput Biol. 2019 Mar 15;15(3):e1006402. doi: 10.1371/journal.pcbi.1006402 (PMC6436762; doi:10.1371/journal.pcbi.1006402)

Supplementary Figure 5

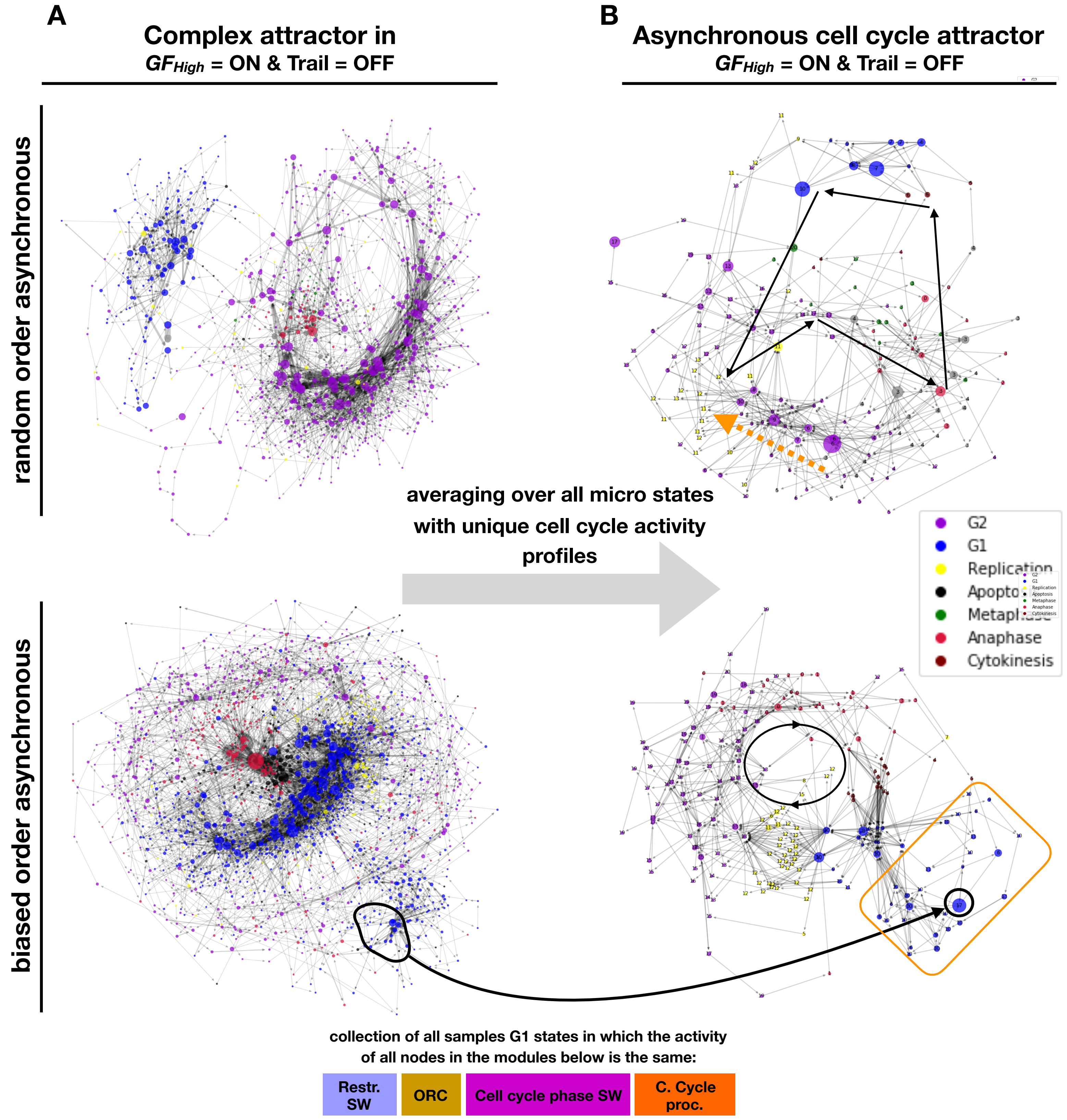

Supplement: S5 Fig — (A) State transition graph of the random order (top) vs. biased random order (bottom) asynchronous models, sampled for 10 independent runs of 1000 time-steps starting from each of the 21 synchronous cell cycle attractor states (cut short if the model reached apoptosis). The largest strongly connected component of each resulting state transition graph representing the cell cycle pseudo-attractor was visualized using the Kamada-Kawai algorithm (NetworkX [67], Python). (B) Projection of each state transition graph onto the sub-space defined by the expression of core cell cycle modules (bottom). Nodes: collection of all states that have identical core cell cycle node activity but differ in the activity of nodes in other modules such as Growth Signaling, illustrated by linked black circles from (A) to (B); Node color: cell cycle phase best approximated by each sampled state; node size: state visitation count; node label: most similar synchronous cell cycle state; black loop (top) & black cycle (bottom): areas of the projected state transition graph with a cyclic pattern of transitions that match the cell cycle; orange arrow (top): direct G2 → S transition (endo-reduplication); orange box (bottom): G0-like pause in the G1 phase of the cell cycle, forming a distinct module apart from the G1 states of cells that pre-commit in their previous cycle. (PDF) [file pcbi.1006402.s005.pdf]
