# Supplementary material for: Boolean model of growth signaling, cell cycle and apoptosis predicts the molecular mechanism of aberrant cell cycle progression driven by hyperactive PI3K
Source: PLoS Comput Biol. 2019 Mar 15;15(3):e1006402. doi: 10.1371/journal.pcbi.1006402 (PMC6436762; doi:10.1371/journal.pcbi.1006402)
Supplement: S11 Fig — Regulatory network surrounding Plk1 expression, enzyme activity and the accumulation of a Plk1H pool driven by FoxO3 or FoxO1. Red nodes: two Boolean nodes representing Plk1 activity and accumulation; Blue nodes: inputs of the two Plk1 nodes. Black arrows: regulation and maintenance of Plk1 expression, activity and persistence; green arrows: feedback on FoxO factors from Plk1, and its downstream target Cyclin B/Cdk1. (PDF) [file pcbi.1006402.s011.pdf]

Supplementary Figure 11

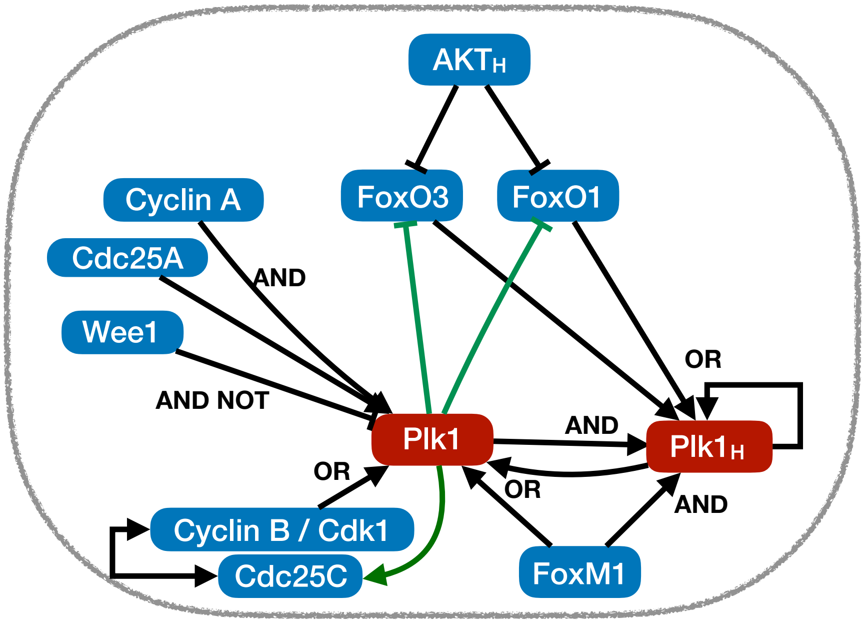

Plk1 = not Cdh1 and (FoxM1 or $Plk1_H$) and [(CyclinB and Cdk1) or (CyclinA and Cdc25A and not Wee1)]

$Plk1_H$ = Plk1 and FoxM1 and (FoxO3 or FoxO1 or $Plk1_H$)
