# Supplementary material for: Boolean model of growth signaling, cell cycle and apoptosis predicts the molecular mechanism of aberrant cell cycle progression driven by hyperactive PI3K
Source: PLoS Comput Biol. 2019 Mar 15;15(3):e1006402. doi: 10.1371/journal.pcbi.1006402 (PMC6436762; doi:10.1371/journal.pcbi.1006402)
Supplement: S1 Text — (A) Dynamics of AKT1 during the cell cycle; (B) High p110 expression in G0 is required for cell cycle entry; (C) Context-dependent timing of R-point passage; (D) Pre-commitment in p110-deficient cells; (E) Assumptions for constructing the regulatory logic of Plk1 and Plk1H. (PDF) [file pcbi.1006402.s014.pdf]

Supplementary Text 1

(A) Dynamics of AKT during the cell cycle

Our synchronous model predicts that *PI3K* oscillations are phase-locked to the cell cycle such that two peaks of *PI3K* / *AKT* signaling occurs in each cycle (**Fig 4**). While we could not find experimental data on the full length of *PI3K* oscillations, *p110* levels recover to ~75% of their initial expression within 4 hours of growth stimulation [1]. This timeframe is consistent with more than one full cycle per division. The asynchronous model shows that indeed there need to be *at least* 2 peaks of *PI3K* / *AKT* per cycle, but there may be more (**Fig 5A**). In these instances the cell pauses between two cycles. The two oscillators remain independent for most of the cycle, except at the metaphase / anaphase transition (**Fig S5**). This leads to strong heterogeneity in *pAKT* activity in G1, S and G2 cycling cells, as observed by [1]. In contrast, in cells entering the cells cycle simulation with both types of update display a high *AKT1* pulse immediately following growth stimulation, and a second peak starting before DNA replication (ending in S-phase; white boxes & arrows on **Fig 4**). Indeed, time-courses of active *AKT1*, measured along the fraction of cells in S-phase, confirm that this modeling result is in line with experimental data (**Table 2**).

(B) High *p110* expression in G0 is required for cell cycle entry

In order to test whether our model can replicate Yuan et al.'s observation that G0 cells with low *p110* levels cannot enter the cell cycle in response to growth factor stimulation [1], we ran *in silico* experiments in which we inhibited the *p110_H* node at different time-points following high growth factor stimulation. As **Fig S8** indicates, our model replicates the lack of cell cycle entry in *p110*-low cells. In contrast, a sufficient initial push of *AKT1* activation in *p110*-high cells can inhibit *FoxO*-mediated $p27^{Kip1}$ expression and aid *Cyclin D* accumulation long enough to trigger *E2F1* activation. Once the *Restriction Switch* is flipped, high *p110* is no longer required for cell cycle completion (**Fig S8A**).

(C) Our model reproduces the context-dependent timing of R-point passage and predicts pre-commitment in *p110*-deficient cells

Our previously published cell cycle model had an intriguing feature, in that it accurately reproduced restriction point passage both in cells entering the cell cycle from quiescence (with the expected R-point in late G1), and in continuously cycling cells [2]. Rapidly dividing mammalian cells such as MCF-10A epithelial cells or HS68 skin cells have recently been

shown to occasionally pre-commit to the next cycle before finishing their current one. While not all cells in the population do this, a subset reliably execute a full cell cycle after mitogen withdrawal such that their last exposure to mitogens occurs sometime during the previous G2 phase. This behavior is experimentally documented in several cell lines (**Table 3**), but previous cell cycle models lacked the molecular mechanism to reproduce it [3-5]. Our previous model indicated that the *Restriction Switch* is responsible for both types of commitment [6]; an effect preserved in our current model (**Fig S9B**). This switch is reset by DNA synthesis, but a large fraction of the inhibitory influences that reset it disappear in G2. The key remaining inhibitor keeping it form fully committing to the next division is the *E2F1* inhibitor *Cyclin A*. As a result, growth factor driven *Myc* expression is required up to the G2/M boundary where *Cyclin A* is degraded. At this point, however, the cell can lock into a committed state, no longer needing growth signaling upstream to complete one more division. Our current model requires active *Ras* → $AKT1_H$ ⊣ *GSK3β* signaling up to this point to aid *Myc* expression until *E2F1* is induced [7]. In contrast, the point of no return for quiescent cells entering the cell cycle is in late G1 (**Fig S9A**).

(D) Our model predicts pre-commitment in *p110*-deficient cells

Surprisingly, our model predicts that (saturating) growth stimulation allows cells to keep cycling in the continued absence of high *p110*. This occurs due to pre-committing to the next cell cycle during mitosis, in spite of the presence of active *GSK3β* (**Fig S10**). This is intriguing, as high *AKT1* is needed in the wild-type model to keep *GSK3β* off, due to its role in promoting *Myc* degradation [8,9]. If high *AKT1* is lost too early, *Myc* turns off before it can re-activate *E2F1*. In contrast *GSK3β* in the *p110*-inhibited model is on throughout G2, while *mTORC1* and *Myc* are both off. In spite of this, our model predicts that cells can still commit to another cell cycle during M-phase. Our model owes this behavior to recent experimental evidence on *AKT1*-independent mTORC1 activation in mitotic cells [10]. This requires the kinase activity of *Cyclin B/Cdk1*, but also that of *GSK3β*. Once *mTORC1* is active, the effect of *GSK3β* on *Myc* is balanced out by the translational elongation factor *eIF4E* [11]. Once active, *Myc* flips the *Restriction Switch* by inducing *Cyclin D* and eventually, *E2F1* (after *pRB* is inhibited by *Cyclin D*; **Fig S10**). *E2F1* re-activation marks the last time-point at which high *ERK* activity is required for pre-commitment to drive *Myc* transcription and stabilize the *E2F1* → *Myc* feedback. In summary, our model predicts that pre-commitment in *p110*-inhibited cells is driven by mitotic *mTORC1* aiding *Myc* re-activation (**Fig S10**). Testing this could elucidate the precise effects of *GSK3β* at the G2/M restriction point, by revealing the relative strengths of *eIF4E*-mediated *Myc* translation and *GSK3*-mediated degradation.

Even though pre-commitment to another cycle does not require ongoing high *p110*, our model does predict that the *fraction* of cells expected to pre-commit in the absence of high

p110 will decrease. This is due to the fact that in these cells the *Restriction Switch* is almost completely reset in G2. In addition, the benefits of this pre-commitment are short-lived. The moment cells finish a cycle without pre-committing — which they do stochastically [13], they cannot reenter the cell cycle. Indeed, our model predicts a strong inhibition of proliferation with efficient *p110* knockdown for growth environments that cannot always force pre-commitment (i.e., less than 100% high growth stimulation, modeled by a stochastically toggling GF_{High} node; *Methods & Model — Modeling non-saturating growth factor stimulation and partial knockdown / overexpression within a Boolean framework*; **Fig S8B-C**).

(E) Assumptions for constructing the regulatory logic of *Plk1* and $Plk1_H$

As shown on **Fig. S11**, key assumptions for constructing the regulatory logic governing *Plk1* were that:

i) maintaining an active pool of *Plk1* requires *Cdk*-mediated activation [14,15], the absence of APC/C^{Cdh1} [16], and either transcription by *FoxM1* [17,18] or sufficient prior *Plk1* accumulation to activate $Plk1_H$,

ii) creating an $Plk1_H$ pool requires active *Plk1*, *FoxM1*, and a brief burst of activity from a *FoxO* factor mitosis [19, 20], and

iii) once created, the $Plk1_H$ pool can be maintained by *FoxM1* and *Plk1* activity. This last requirement is important, as high *Plk1* activity shuts down *FoxO3* [21] and *FoxO1* [22].

References

1. Lemmon MA, Schlessinger J. Cell signaling by receptor tyrosine kinases. Cell. 2010;141: 1117–1134. doi:10.1016/j.cell.2010.06.011
2. Avram S, Mernea M, Mihailescu DF, Seiman CD, Seiman DD, Putz MV. Mitotic checkpoint proteins Mad1 and Mad2 - structural and functional relationship with implication in genetic diseases. Curr Comput Aided Drug Des. 2014;10: 168–181.
3. Toettcher JE, Loewer A, Ostheimer GJ, Yaffe MB, Tidor B, Lahav G. Distinct mechanisms act in concert to mediate cell cycle arrest. Proceedings of the National Academy of Sciences. 2009;106: 785–790.
4. Gérard C, Goldbeter A, Tyson JJ. Temporal Self-Organization of the Cyclin/Cdk Network Driving the Mammalian Cell Cycle. Proc Natl Acad Sci USA. National Academy of Sciences; 2009;106: 21643–21648.
5. Singhania R, Sramkoski RM, Jacobberger JW, Tyson JJ. A Hybrid Model of Mammalian Cell Cycle Regulation. Beard DA, editor. PLoS Computational Biology. 2011;7: e1001077.
6. Deritei D, Aird WC, Ercsey-Ravasz M, Regan ER. Principles of dynamical modularity in biological regulatory networks. Sci Rep. Nature Publishing Group; 2016;6: 21957. doi:10.1038/srep21957
7. Gupta S, Ramjaun AR, Haiko P, Wang Y, Warne PH, Nicke B, et al. Binding of ras to phosphoinositide 3-kinase p110alpha is required for ras-driven tumorigenesis in mice. Cell. 2007;129: 957–968. doi:10.1016/j.cell.2007.03.051

8. Sears R, Nuckolls F, Haura E, Taya Y, Tamai K, Nevins JR. Multiple Ras-dependent phosphorylation pathways regulate Myc protein stability. Genes & Development. 2000;14: 2501–2514. doi:10.1101/gad.836800

9. Welcker M, Orian A, Jin J, Grim JE, Grim JA, Harper JW, et al. The Fbw7 tumor suppressor regulates glycogen synthase kinase 3 phosphorylation-dependent c-Myc protein degradation. Proc Natl Acad Sci U S A. 2004;101: 9085–9090. doi:10.1073/pnas.0402770101

10. Ramirez-Valle F, Badura ML, Braunstein S, Narasimhan M, Schneider RJ. Mitotic Raptor Promotes mTORC1 Activity, G2/M Cell Cycle Progression, and Internal Ribosome Entry Site-Mediated mRNA Translation. Molecular and Cellular Biology. 2010;30: 3151–3164. doi:10.1128/MCB.00322-09

11. Saxton RA, Sabatini DM. mTOR Signaling in Growth, Metabolism, and Disease. Cell. 2017;169: 361–371. doi:10.1016/j.cell.2017.03.035

13. Spencer SL, Cappell SD, Tsai F-C, Overton KW, Wang CL, Meyer T. The proliferation-quiescence decision is controlled by a bifurcation in CDK2 activity at mitotic exit. Cell. 2013;155: 369–383. doi:10.1016/j.cell.2013.08.062

14. Thomas Y, Cirillo L, Panbianco C, Martino L, Tavernier N, Schwager F, et al. Cdk1 Phosphorylates SPAT-1/Bora to Promote Plk1 Activation in C. elegans and Human Cells. Cell Rep. 2016;15: 510–518. doi:10.1016/j.celrep.2016.03.049

15. Gheghiani L, Loew D, Lombard B, Mansfeld J, Gavet O. PLK1 Activation in Late G2 Sets Up Commitment to Mitosis. Cell Rep. 2017;19: 2060–2073. doi:10.1016/j.celrep.2017.05.031

16. Lindon C, Pines J. Ordered proteolysis in anaphase inactivates Plk1 to contribute to proper mitotic exit in human cells. The Journal of Cell Biology. 2004;164: 233–241. doi:10.1083/jcb.200309035

17. Laoukili J, Kooistra MRH, Brás A, Kauw J, Kerkhoven RM, Morrison A, et al. FoxM1 is required for execution of the mitotic programme and chromosome stability. Nature Cell Biology. 2005;7: 126–136. doi:10.1038/ncb1217

18. Fu Z, Malureanu L, Huang J, Wang W, Li H, van Deursen JM, et al. Plk1-dependent phosphorylation of FoxM1 regulates a transcriptional programme required for mitotic progression. Nature Cell Biology. 2008;10: 1076–1082. doi:10.1038/ncb1767

19. Alvarez B, Martínez-A C, Burgering BM, Carrera AC. Forkhead transcription factors contribute to execution of the mitotic programme in mammals. Nature. 2001;413: 744–747. doi:10.1038/35099574

20. Yuan Z, Becker EBE, Merlo P, Yamada T, DiBacco S, Konishi Y, et al. Activation of FOXO1 by Cdk1 in cycling cells and postmitotic neurons. Science. 2008;319: 1665–1668. doi:10.1126/science.1152337

21. Bucur O, Stancu AL, Muraru MS, Melet A, Petrescu SM, Khosravi-Far R. PLK1 is a binding partner and a negative regulator of FOXO3 tumor suppressor. Discoveries (Craiova). 2014;2: e16. doi:10.15190/d.2014.8

22. Yuan C, Wang L, Zhou L, Fu Z. The function of FOXO1 in the late phases of the cell cycle is suppressed by PLK1-mediated phosphorylation. Cell Cycle. 2014;13: 807–819. doi:10.4161/cc.27727
