# Supplementary material for: Boolean model of growth signaling, cell cycle and apoptosis predicts the molecular mechanism of aberrant cell cycle progression driven by hyperactive PI3K
Source: PLoS Comput Biol. 2019 Mar 15;15(3):e1006402. doi: 10.1371/journal.pcbi.1006402 (PMC6436762; doi:10.1371/journal.pcbi.1006402)
Supplement: S1 Table — Explanation and literature support for each individual link and regulatory logic gate in the model. (A) Growth signaling; (B) Restriction switch; (C) Phase switch; (D) Origin of replication licensing; (E) Cell cycle processes; (F) Apoptotic switch. (PDF) [file pcbi.1006402.s015.pdf]

**Table S1. Description and experimental support of the model's Boolean regulatory logic.**

**S1.A.** Boolean regulatory logic of Growth Signaling nodes.

| Target Node | Link | Input | Description & References |
|---|---|---|---|
| **GF_High** | ← | **GF_High** | The GFHigh node in our model represents an extracellular environment with saturating levels of growth factors; this input node is self-sustaining in the absence of in silico perturbation. |
| **GF** | ← | **GF_High** | The GF node represents an extracellular environment with low levels of growth factors capable of sustaining survival signaling. Thus, this node is ON in high growth factor as well. |
| **GF** | ← | **GF** | The GF input node is self-sustaining in the absence of in silico perturbation. |
| **GF** | **GF** or **GF_High** | | |
| **Growth Factor Signaling - Upstream PI3K cycle** | | | |
| **RTK** | ⊢ | **CAD** | Caspase-activated DNAase (CAD) inhibition of receptor tyrosine kinases ensures that apoptotic cells no longer maintain even basal levels of growth signaling. |
| **RTK** | ← | **GF** | The ON state of the RTK node in our model represents basal growth receptor activation by low / medium growth factor availability, encoded by the GF node (required to keep a normal cell alive). |
| **RTK** | ← | **GF_High** | Similarly, high growth factor availability also keeps RTK on. |
| **RTK** | not **CAD** and (**GF_High** or **GF**) | | |
| | The ON state of the RTK node in our model represents basal growth receptor activation (required to keep a normal cell alive). Thus it requires the absence of CAD and at least low growth levels of growth factors in the extracellular environment [1]. | | |
| **Grb2** | ← | **RTK** | The SH2 domain of Grb2 binds to a phosphotyrosine residue in the activated RTK, where it functions as an adaptor protein [2]. |
| **Grb2** | ← | **GF_High** | The ON state of Grb2 node in our model encode the change from basal (or absent) Grb2 activity at weakly stimulated RTKs to the level of recruitment seen in high growth factor environments, capable of mediating Ras activation. |
| **Grb2** | **RTK** and **GF_High** | | |
| | Grb2 is ON when the RTK is activated by high levels of extracellular growth factors (capable of driving proliferation). | | |
| **SOS** | ← | **Grb2** | RTK-bound Grb2 recruits SOS, a guanine nucleotide – exchange protein (GEF) that converts inactive Ras to its active GTP-bound form [2]. |
| **Ras** | ← | **Grb2** | RTK-bound Grb2 is required to recruits SOS, the GEF responsible for converting inactive Ras to its GTP-bound active form [2]. |
| **Ras** | ← | **SOS** | SOS is a GEF that is recruited to activate Ras near ligand-bound, active RTKs [2]. |

| Target Node | Link | Input | Description & References |
|---|---|---|---|
| **Ras** | **Grb2** and **SOS** | | |
| | Ras activation requires the GEF activity of SOS and the RTK-linked (active) adaptor protein Grb2 [2]. | | |
| **RAF** | ⊢ | **Casp3** | Raf-1 is cleaved and inhibited by Caspase 3 [3]. |
| **RAF** | ← | **Ras** | Active Ras phosphorylates Raf, enhancing its kinase activity [4]. |
| **RAF** | not **Casp3** and **Ras** | | |
| | Raf is active in response to Ras activity in the absence of Caspase 3. As active Raf-1 is continuously dephosphorylated and bound by 14-3-3 which translocates it to the cytoplasm from the plasma membrane (not modeled explicitly), ongoing Ras activity is necessary to keep it ON [4]. | | |
| **MEK** | ← | **RAF** | Raf phosphorylates and activates the MEK kinase [4]. |
| **ERK** | ← | **MEK** | MEK phosphorylates and activates the ERK kinase [4]. |
| **mTORC2** | ← | **PIP3** | PtdIns(3,4,5)P3 (PIP3), interacts with the mTORC2 component Sin1 to release its inhibition on the mTOR kinase domain. Thus, PIP3 is necessary for mTORC2 activation [5]. |
| **mTORC2** | ⊢ | **S6K** | Rictor, a component of the mTORC2 complex, undergoes S6K1-mediated phosphorylation at T1135, dampening mTORC2-dependent phosphorylation of Akt [6] |
| **mTORC2** | **PIP3** or not **S6K** | | |
| | Our model assumes that mTORC2 is active in quiescent cells with basal levels of PI3K activity leading to basal PIP3 generation. Alternatively, the absence of high growth factor-stimulated mTORC1 and S6K1 can also increase mTORC2 activity. | | |
| **PI3K** | ← | **Ras** | Ras binds the catalytic subunit of PI3K and Ras knockdown / over expression decreases /increases the PI3K-dependent generation of PIP3 [7,8]. |
| **PI3K** | ← | **RTK** | Active RTKs recruit PI3K to the signaling complex they nucleate, where PI3K catalyzes the production of PtdIns(3,4,5)P3 (PIP3) [1]. |
| **PI3K** | **Ras** or **RTK** | | |
| | In our model, basal PI3K activity can be maintained by active RTKs or active Ras, while high PI3K activity requires both (see below). | | |
| **PIP3** | ← | **PI3K** | Active PI3K recruited to the membrane catalyzes the production of membrane-bound PtdIns(3,4,5)P3 (PIP3) from PtdIns(4,5)P2 (PIP2) [1]. |
| **PIP3** | ← | **PI3K_H** | Active PI3K recruited to the membrane catalyzes the production of membrane-bound PtdIns(3,4,5)P3 (PIP3) from PtdIns(4,5)P2 (PIP2) [1]. |
| **PIP3** | **PI3K_H** or **PI3K** | | |
| | In our model, PIP3 is ON as a result of basal or high PI3K activity. | | |

| Target Node | Link | Input | Description & References |
|---|---|---|---|
| **PDK1** | ← | **PI3K** | The PDK1 kinase is recruited to the plasma membrane by PIP3 at the sites of active PI3K activity [9]. |
| **PDK1** | ← | **PIP3** | The PDK1 kinase is recruited to the plasma membrane by PIP3 at the sites of active PI3K activity [9]. |
| **PDK1** | **PI3K** and **PIP3** | | |
| | PDK1 enzyme activation requires active (at least basal) PI3K and PIP3 [9]. | | |
| **AKT_B** | ⊢ | **Casp3** | AKT1 is cleaved and inhibited by Caspase 3 [3]. |
| **AKT_B** | ← | **PIP3** | PIP3 recruits AKT to the plasma membrane and PIP3 binding changes the conformation of AKT such that it becomes accessible for T308 phosphorylation by PDK1 [9]. |
| **AKT_B** | ← | **PDK1** | Membrane-recruited PDK1 phosphorylates AKT at T308, a critical step in its activation [9]. |
| **AKT_B** | ← | **mTORC2** | Maximal activation of AKT requires phosphorylation of S473 by mTORC2 [9]. |
| **AKT_B** | not **Casp3** and **PIP3** and (**PDK1** or **mTORC2**) | | |
| | Basal AKT1 activity in our model requires the absence of Caspase 3, the availability of at least basal levels of PIP3, and phosphorylation by PDK1 or mTORC2. In contrast, full mitogen-stimulated AKT1 activation requires phosphorylation by both (see AKT_H) [9]. | | |
| **Growth Factor Signaling - PI3K cycle** | | | |
| **p110_H** | ← | **FoxO3** | FoxO3 is a direct inducer p110α (PIK3CA), the catalytic subunit of PI3K [10]. |
| **p110_H** | ⊢ | **NeddL4** | p110α (PIK3CA) is polyubiquitinated by the E3 ligase NEDD4L, leading to its proteasomal degradation. Both free p110 and the regulatory subunit-bound protein is subject to ubiquitination by NEDD4L [11]. |
| **p110_H** | ← | **p110_H** | Our model assumes that maintaining high p110 levels is easier than driving the re-accumulation of the protein following its rapid destruction. |
| **p110_H** | (**FoxO3** and not **NeddL4**) or [**p110_H** and (**FoxO3** or not **NeddL4**)] | | |
| | In order to capture the cyclic dynamics of p110 protein expression, we make the assumption that high p110 protein levels can be induced by FoxO3 in the absence of the growth factor-activated NeddL4 ubiquitin ligase. Once present, high p110 can be maintained by FoxO3 transcription, or the absence of activated NeddL4. | | |
| **PI3K_H** | ← | **p110_H** | High levels of PI3K activity in response to strong growth factor stimulation only occur in cells that express high levels of p110 protein [12]. |
| **PI3K_H** | ← | **PI3K** | In our model, high PI3K activation is contingent on the ON-state of the basal PI3K node. |
| **PI3K_H** | ← | **RTK** | High levels of PI3K activation only occur near at growth factor-bound RTKs, which recruit and activate PI3K at the plasma membrane [9]. |

| Target Node | Link | Input | Description & References |
|---|---|---|---|
| **PI3K_H** | ← | **Ras** | Ras binds the catalytic subunit of PI3K and Ras knockdown / over expression decreases /increases the PI3K-dependent generation of PIP3 [7,8]. |
| **PI3K_H** | **p110_H** and **PI3K** and **RTK** and **Ras** | | |
| | Full, peak-level activation of PI3K requires high levels of p110 protein, basal PI3K activation, active RTKs, and active Ras. As the ON-state of Ras in our model represents strong Ras activation in the presence of proliferation-inducing (high) growth factors, PI3K_H activation can only occur in these conditions. | | |
| **AKT_H** | ← | **AKT_B** | In our model, high AKT activation is contingent on the ON-state of basal AKT (AKT_B). |
| **AKT_H** | ← | **p110_H** | Ongoing high p110 availability and PI3K_H activity are required to induce maximal activation of AKT_H [9]. |
| **AKT_H** | ← | **PI3K_H** | Ongoing high p110 availability and PI3K_H activity are required to induce maximal activation of AKT_H [9]. |
| **AKT_H** | ← | **PIP3** | PIP3 recruits AKT to the plasma membrane and PIP3 binding changes the conformation of AKT such that it becomes accessible for T308 phosphorylation by PDK1 [9]. |
| **AKT_H** | ← | **PDK1** | Membrane-recruited PDK1 phosphorylates AKT at T308, a critical step in its activation [9]. |
| **AKT_H** | ← | **mTORC2** | Maximal activation of AKT requires phosphorylation of S473 by mTORC2 [9]. |
| **AKT_H** | ← | **Ras** | Ras binding to the catalytic subunit of PI3K is required for its full potency in PIP3 generation [7,8]. Active Ras is thus required for inducing peak AKT_H activity. |
| **AKT_H** | **AKT_B** and **p110_H** and **PI3K_H** and **PIP3** and **PDK1** and **mTORC2** and **Ras** | | |
| | In contact to basal AKT, high AKT activity in our model requires basal AKT (AKT_B), the ongoing presence of high p110 protein levels along with active PI3K_H, and PIP3. In addition this maximal AKT activation requires phosphorylation by both PDK1 and mTORC2, as well as active Ras [9]. | | |
| **FoxO3** | ⊢ | **AKT_H** | AKT mediates the translation of FoxO3 our of the nucleus through direct phosphorylation of three conserved residues. These events create a recognition site for 14-3-3 family proteins, which export and sequester FoxO3 in the cytosol [9]. |
| **FoxO3** | ⊢ | **AKT_B** | AKT mediates the translation of the FoxO3 transcription factor our of the nucleus (see above). |
| **FoxO3** | ⊢ | **ERK** | ERK downregulates FOXO3 transactional activity by phosphorylating it at three Serines, inducing its MDM2-mediated ubiquitination and degradation [13]. |
| **FoxO3** | ⊢ | **Plk1** | Plk1 binds FoxO3, induces its translocation to the cytosol, phosphorylates it and suppresses its activity through most of the the cell cycle, but most significantly during G2 and M [14]. |
| **FoxO3** | ⊢ | **Plk1_H** | Plk1 binds FoxO3, induces its translocation to the cytosol (see above). |

| Target Node | Link | Input | Description & References |
|---|---|---|---|
| **FoxO3** | not (**AKT_B** or **AKT_H** or **ERK**) or { not [**AKT_H** and (**Plk1 or Plk1_H or AKT_B or ERK**) ] and not (**Plk1 and Plk1_H and ERK**) } | | |
| | In order to account for all the influences on FoxO3 activity, we used the following logic. In the absence of basal or high AKT as well as ERK, FoxO3 remains active. In addition, FoxO3 can overcame peak (AKT_H) activation only if no other inhibitor is present and AKT_B is OFF (indicating that AKT levels are falling). Finally, the joint activity of ERK and Plk1 can also block FoxO3. | | |
| **PLCgamma** | ← | **RTK** | The SH2 domains of PLCγ binds to active RTKs at tyrosine autophosphorylation sites, leading to tyrosine phosphorylation of PLCγ and stimulation its enzymatic activity [15,16]. |
| **PLCgamma** | ← | **Grb2** | RTK tyrosine autophosphorylation induces PLCγ binding to the Grb2 adaptor protein and likely aids the translocation of PLCγ to the plasma membrane [17]. |
| **PLCgamma** | ← | **p110_H** | Membrane targeting of PLCγ to the sight of growth receptor stimulation requires PI3K activity and PIP3 generation near growth receptors [18]. Thus, peak PLCγ activity in our model requires high p110 protein expression [19]. |
| **PLCgamma** | ← | **PI3K_H** | In addition to high p110 protein levels, high PI3K activation is also required to fully activate PLCγ [18,19]. |
| **PLCgamma** | ← | **PIP3** | Membrane targeting of PLCγ to the sight of growth receptor stimulation is mediated by PIP3 binding of PLCγ [18,19]. |
| **PLCgamma** | **RTK** and **Grb2** and **p110_H** and **PI3K_H** and **PIP3** | | |
| | Peak activation of PLCγ requires active an RTK receptor node bound by active Grb2, as well as high PI3K activity (including high p110 availability and the presence of PIP3). | | |
| **IP3** | ← | **PLCgamma** | Membrane-bound, active PLCγ is responsible for converting phosphatidylinositol(4,5)P2 (PIP2) to the second messenger inositol(1,4,5)P3 (IP3) responsible for triggering a sudden $Ca^{2+}$ influx from the endoplasmic reticulum, along with DAG (diacylglycerol, another second messenger) [20]. |
| **Ca2+** | ← | **IP3** | IP3 travels from the cell membrane to the endoplasmic reticulum where it opens IP3-sensitive $Ca^{2+}$ channels, releasing a sudden $Ca^{2+}$ efflux from the ER into the cytosol [21]. |
| **NeddL4** | ← | **Ca2+** | In order to transition to its active form, the E3 ubiquitin ligase NeddL4 binds $Ca^{2+}$ and inositol 1,4,5-trisphosphate (IP3) [22]. |
| **NeddL4** | ← | **IP3** | In order to transition to its active form, the E3 ubiquitin ligase NeddL4 binds $Ca^{2+}$ and inositol 1,4,5-trisphosphate (IP3) [22]. |
| **NeddL4** | **Ca2+** and **IP3** | | |
| | Activation of NeddL4 requires both Ca2+ and IP3 binding [22]. | | |
| **Growth Factor Signaling - Downstream PI3K cycle** | | | |
| **FoxO1** | ⊢ | **AKT_H** | AKT mediates the translation of FoxO1 our of the nucleus through direct phosphorylation of three conserved residues. These events create a recognition site for 14-3-3 family proteins, which export and sequester FoxO1 in the cytosol [9]. |

| Target Node | Link | Input | Description & References |
|---|---|---|---|
| **FoxO1** | ⊢ | **Plk1** | PLK1 interacts with and phosphorylates FOXO1, mainly at the G 2/M phase of the cell cycle. PLK1-mediated phosphorylation leads to the impairment of FOXO1's transcriptional activity in an Akt-independent manner. By immunofluorescence staining and subcellular fractionation, we demonstrate that PLK1-induced FOXO1 phosphorylation causes its nuclear exclusion. [23] |
| **FoxO1** | not **Plk1** and not **AKT_H** | | |
| | FoxO1 is transcriptionally active in the absence of peak AKT activation and Plk1 activity. | | |
| **p21_mRNA** | ← | **FoxO1** | p21$^{Cip1}$ is a direct transcriptional target of FoxO1 [24]. |
| **p21_mRNA** | ← | **FoxO3** | p21$^{Cip1}$ is a direct transcriptional target of FoxO3 [24]. |
| **p21_mRNA** | ⊢ | **Myc** | Myc is a direct transcriptional repressor of the p21$^{Cip1}$ promoter (it is recruited by the DNA-binding Miz-1) [25,26]. |
| **p21_mRNA** | (**FoxO1** and **FoxO3**) or [not **Myc** and (**FoxO1** or **FoxO3**)] | | |
| | Our model requires both FoxOs to induce p21$^{Cip1}$ if Myc is active and one of the two if Myc is OFF. This is based on data showing that both FoxO3 and FoxO1 bind and induce the p21$^{Cip1}$ promoter and that loss of Myc repression alone is not sufficient to induce p21$^{Cip1}$ [24]. | | |
| **TSC2** | ⊢ | **AKT_H** | TSC2 is phosphorylated by AKT1, inhibiting it by dissociating TSC2 from lysosomal membranes [27], where it stimulates GTP hydrolysis of the small GTPase Rheb, this inactivating it [28]. |
| **TSC2** | ⊢ | **AKT_B** | TSC2 is phosphorylated by AKT1, inhibiting it by dissociating TSC2 from lysosomal membranes [27], where it stimulates GTP hydrolysis of the small GTPase Rheb, this inactivating it [28]. |
| **TSC2** | ⊢ | **ERK** | ERK phosphorylates TSC2 directly, causing dissociation of the complex and inhibition of its activity [29]. In addition, the ERK target p90RSK can also inactivate TSC2 [30]. |
| **TSC2** | not **AKT_H** or not (**AKT_B** or **ERK**) | | |
| | Blocking TSC2 requires ongoing mitogen stimulation through AKT and/or ERK. In our model, TSC2 inhibition requires high (peak) AKT activity, supported by either ERK or basal AKT (assuring that complete loss of AKT activity is not impending) [28]. | | |
| **PRAS40** | ⊢ | **AKT_H** | PRAS40 is an inhibitory component of the mTORC1 complex. It is phosphorylated by AKT, triggering its dissociation from mTORC1 and loss of mTORC1 inhibition [31]. |
| **PRAS40** | ⊢ | **AKT_B** | PRAS40 is phosphorylated by AKT, triggering its dissociation from mTORC1 [31]. |
| **PRAS40** | ⊢ | **mTORC1** | PRAS40 is a substrate of the mTORC1 kinase; its phosphorylation aids its dissociation from mTORC1 and its sequestration by 14-3-3 proteins [32]. |
| **PRAS40** | not **AKT_H** and (not **mTORC1** or not **AKT_B**) | | |
| | PRAS40 is inhibited by peak AKT activity aided by either basal AKT (meaning AKT_H is on its way down), or ongoing mTORC1 activation. Both Akt and mTORC1 phosphorylate PRAS40, leading to its dissociation from mTORC1 [33]. | | |

| Target Node | Link | Input | Description & References |
|---|---|---|---|
| **DAG** | ← | **PLCgamma** | Membrane-bound, active PLCγ is responsible for converting phosphatidylinositol(4,5)P2 (PIP2) to the second messenger diacylglycerol (DAG), along with IP3 [20]. |
| **Rheb** | ⊢ | **TSC2** | TSC2, a key component of the heterotrimeric TSC complex, is a GTPase activating protein (GAP) that induces ATP hydrolysis and deactivation of the small GTPase Rheb [34]. |
| **Rheb** | ← | **DAG** | The second messenger DAG activates both classical and novel PKCs. One of its targets, PKCη, is responsible for the translocation and accumulation of mTORC1 to perinuclear lysosomes, where the majority of Rheb is anchored. Thus, DAG brings Rheb in proximity with its target, mTORC1 [35]. |
| **Rheb** | not **TSC2** and **DAG** | | |
| | PKC (and DAG)-dependent activation of mTORC1 recruits mTORC1 to the site of Rheb activity (to prenuclear lysosomes), while AKT and ERK-mediated TSC2 inhibition guarantees that Rheb remains potent [34-36]. | | |
| **mTORC1** | ⊢ | **Casp3** | Raptor, a key component of the mTORC1 complex, is cleaved and inhibited by Caspase 3 [37]. |
| **mTORC1** | ← | **Rheb** | The Rheb small GTPase binds mTORC1 directly and activates the complex [38]. |
| **mTORC1** | ⊢ | **PRAS40** | PRAS40 is an inhibitory component of the mTORC1 complex, removed by phosphorylation by AKT or mTORC1 itself [28]. |
| **mTORC1** | ← | **E2F1** | E2F1 induces mTORC1 activity by inducing mTORC1 translocation to late endosomes. This effect does not require AKT and is not blocked by high levels of TSC2 [39]. |
| **mTORC1** | ← | **Cdk1** | During mitosis, mTORC1 is activated by the G2/M-specific phosphorylation of Raptor, a component of mTORC1, by CyclinB / Cdk1 complexes, aided by GSK3 [40]. |
| **mTORC1** | ← | **CyclinB** | mTORC1 is activated by the G2/M-specific phosphorylation of Raptor, a component of mTORC1, by CyclinB / Cdk1 complexes, aided by GSK3 [40]. |
| **mTORC1** | ← | **GSK3** | mTORC1 is activated by the G2/M-specific phosphorylation of Raptor, a component of mTORC1, by CyclinB / Cdk1 complexes, aided by GSK3 [40]. |
| **mTORC1** | not **Casp3** and [ (**Rheb** and not **PRAS40**) or **E2F1** or (**CyclinB** and **Cdk1** and **GSK3**) ] | | |
| | mTORC1 is activated by mitogenic signals via Rheb in the absence of PRAS40. Independently of this, E2F1 can promote mTORC1 activity. Finally, the mitotic Cyclin B and its Cdk1 kinase can also activate mTORC1, aided by GSK3. | | |
| **S6K** | ⊢ | **Casp3** | S6K is cleaved and inhibited by Caspase 3 [41]. |
| **S6K** | ← | **mTORC1** | mTORC1 phosphorylates and activates 40S ribosomal S6 kinases (S6Ks) [42]. |
| **S6K** | not **Casp3** and m**TORC1** | | |
| | S6K is activated by mTORC1 in the absence of Caspase 3. | | |

| Target Node | Link | Input | Description & References |
|---|---|---|---|
| **eIF4E** | ⊢ | **Casp3** | eIF4E is cleaved and inhibited by Caspase 3 [43]. |
| **eIF4E** | ← | **mTORC1** | mTORC1 phosphorylates 4EBP, triggering its dissociation from eIF4F, and thus promoting translation initiation [28]. |
| **eIF4E** | not **Casp3** and **mTORC1** | | |
| | eIF4E is activated by mTORC1-mediated repression in the absence of Caspase 3. | | |
| **GSK3** | ⊢ | **AKT_H** | AKT blocks GSK3 kinase activity via an inhibitory phosphorylation on the amino terminus, which blocks the substrate accessibility of GSK3 [9]. |
| **GSK3** | ⊢ | **ERK** | ERK binds and phosphorylates GSK3β at Thr-43, which primes it for subsequent phosphorylation by the ERK target p90RSK at Ser-9, which inactivates GSK3β [44]. |
| **GSK3** | ⊢ | **S6K** | GSK3 is a direct phosphorylation target of S6K1, resulting in its inhibition [45]. |
| **GSK3** | not **AKT_H** and not (**S6K** and **ERK**) | | |
| | GSK3 activity can be completely blocked by peak AKT activation (AKT_H), or by the joint action of S6K and ERK. | | |

**S1.B.** Boolean regulatory logic of Restriction Switch nodes.

| Target Node | Link | Input | Description & References |
|---|---|---|---|
| **p21** | ← | **p21_mRNA** | p21 protein activity requires the presence of p21 transcription and mRNA. |
| **p21** | ⊢ | **Casp3** | Caspase 3 cleaves and deactivates p21Cip1 [46]. |
| **p21** | ⊢ | **CyclinE** | p21 and Cyclin E / Cdk2 form a positive (double-negative) feedback loop in which Cyclin E / Cdk2 activates the SCF/Skp2 complex responsible for the degradation of Cyclin E / Cdk2-bound, phosphorylated p21 [47]. p21, in turn, not only blocks Cyclin E / Cdk2 activity, but it also inhibits Cyclin D1. Thus, p21 interferes with the mitogen signal that turns on Cyclin E in the first place. In quiescent cells with high basal p21 levels, this positive feedback renders cell cycle entry stochastic [48]. |
| **p21** | **p21_mRNA** and not **Casp3** and not **CyclinE** | | |
| | In this model, the p21 node corresponds to nuclear p21 in cells with relatively high basal p21 activity. p21 activity and or localization can be lowered by loss of FoxO mediated transcription (see p21_mRNA node) and via feedback from Cyclin E / Cdk2 [48]. | | |
| **pRB** | ⊢ | **Casp3** | Caspase 3 cleaves RB, generating fragments that do not associate with E2F, rendering RB inactive [49]. |
| **pRB** | ⊢ | **CyclinD1** | Cyclin D1 / Cdk4,6 complexes bind and phosphorylate RB, inhibiting its activity [50,51]. |
| **pRB** | ⊢ | **CyclinA** | Cyclin A / Cdk2 complexes phosphorylate and deactivate RB [52]. |

| Target Node | Link | Input | Description & References |
|---|---|---|---|
| **pRB** | ← | **p27Kip1** | Active p27Kip1 can counteract the inhibitory effects of active CyclinE/Cdk2 complexes [53]. |
| **pRB** | ⊢ | **CyclinE** | Cyclin E / Cdk2 complexes bind and phosphorylate RB, inhibiting its activity [51,54]. |
| **pRB** | not **Casp3** and not **CyclinD1** and not **CyclinA** and [**p27Kip1** or not **CyclinE**] | | |
| | pRB is active in the absence of Caspase 3, CyclinD1 and CyclinA. In addition, pRB maintains its activity when active p27Kip1 counteracts the effects of CyclinE [53]. | | |
| **p27Kip1** | ⊢ | **Casp3** | Caspase 3 cleaves  p27Kip1 [55]; the cleaved fragments can no longer associate with Cdk2 / Cyclin complexes [56]. |
| **p27Kip1** | ⊢ | **CyclinD1** | Active Cyclin D1 / Cdk4,6 complexes competitively bind to p27Kip1 and progressively inhibit its ability to keep Cyclin E / Cdk2 inactive [53]. |
| **p27Kip1** | ⊢ | **Cdk1** | Cyclin B / Cdk1 complexes phosphorylate p27Kip1 [57] and although they do not promote its degradation, phosphorylated p27Kip1 is exported from the nucleus and looses its ability to inhibit Cdk activity [58]. |
| **p27Kip1** | ⊢ | **CyclinB** | Cyclin B / Cdk1 complexes phosphorylate p27Kip1 [57] and although they do not promote its degradation, phosphorylated p27Kip1 is exported from the nucleus and looses its ability to inhibit Cdk activity [58]. |
| **p27Kip1** | ⊢ | **CyclinE** | Active Cyclin E / Cdk2 phosphorylate p27Kip1 [59], which marks it for degradation by the SCFSKP2 complex at the onset of S-phase [60]. (Cyclin- E/Cdk2 complexes can remain active in the presence of p27Kip1 when Cyclin A is also active). |
| **p27Kip1** | ⊢ | **CyclinA** | Cyclin A / Cdk2 complexes sequester and inactivate p27Kip1, phosphorylate it, and promote its degradation [57]. |
| **p27Kip1** | ← | **FoxO1** | FoxO factors are direct inducers of p27Kip1 expression. [61]. |
| **p27Kip1** | ← | **FoxO3** | FoxO factors are direct inducers of p27Kip1 expression. [61]. |
| **p27Kip1** | not **Casp3** and not **CyclinD1** and not (**Cdk1** and **CyclinB**) and { [not **CyclinE** and (**FoxO3** and **FoxO1**) ] or [not **CyclinA** and (**FoxO3** or **FoxO1**) ] or (not **CyclinE** and not **CyclinA**) } | | |
| | Active p27Kip1 is cleaved by Caspase 3 and inhibited (sequestered) by Cyclin D1/Cdk4,6 [53] or Cyclin B / Cdk1 [58]. In addition, maintenance of p27Kip1 requires one or both FoxO factors when sequestered by Cyclin E / Cdk2 (one FoxO factor) or Cyclin A / Cdk2 (both FoxO factors), but it cannot keep pace with the simultaneous activity of Cyclin E / Cdk2 and Cyclin A / Cdk2 [62]. | | |
| **Myc** | ← | **ERK** | Ser-62 phosphorylation by ERK increases its half life, leading to Myc accumulation [63,64]. |
| **Myc** | ⊢ | **GSK3** | Thr-58 phosphorylation by GSK-3 promotes Myc degradation [63,65] |
| **Myc** | ← | **eIF4E** | Increased translational initiation in the presence of activated eIF4E leads to an increase in Myc protein levels [66]. |

| Target Node | Link | Input | Description & References |
|---|---|---|---|
| **Myc** | ← | **E2F1** | E2F1 binds and activates the c-Myc promoter [67,68] [34, 33, 58, 59]. |
| **Myc** | ⊢ | **pRB** | E2F1's ability to induce Myc is blocked by active (hypo-phosphorylated pRB) [69]. |
| **Myc** | [**ERK** and (**eIF4E** or not **GSK3**)] or [**E2F1** and not **pRB** and (**eIF4E** or **ERK** or not **GSK3**) ] | | |
| | Myc activity is turned on by stabilization of the protein via ERK phosphorylation, aided by either an increase in translation initiated by eIF4E or loss of degradation-promoting phosphorylation by GSK3 [63]. Alternatively, increased transcription by E2F1 can also promote Myc accumulation in the absence of active pRB [70], provided that the protein is stabilized by ERK, eIF4E, or the absence of GSK3. | | |
| **CyclinD1** | ⊢ | **CHK1** | During replication, checkpoint kinases such as CHK1 (active during normal DNA synthesis) suppress Cyclin D1 [71], which has a very short half- life (~ 24 min) [72]. |
| **CyclinD1** | ⊢ | **p21** | p21Cip1 is a Cyclin Dependent kinase inhibitor which binds to and blocks the activity of Cdk2, Cdk3, Cdk4 and Cdk6 kinases [73] and thus inhibits CyclinD1/Cdk4,6 [74]. |
| **CyclinD1** | ← | **E2F1** | The Cyclin D1 promoter is bound by E2F factors including E2F1; E2F1 over-expression can increase Cyclin D1 (though its effects are context-dependent, as E2F1 over-expression can also lead to apoptosis) [75]. Dominant negative E2F1 over-expression results in a 2-3 fold decrease in Cyclin D1 expression and Cyclin D1/Cdk4,6 activity [76]. |
| **CyclinD1** | ⊢ | **pRB** | E2F1's ability to induce CyclinD1 is blocked by active (hypo-phosphorylated pRB) [77]. |
| **CyclinD1** | ← | **Myc** | Extracellular growth signals activate the MAPK pathway, leading to transcriptional activation of Cyclin D1 by Myc [78,79]. Myc overexpression leads to rapid Cyclin D1 induction and subsequent cell cycle entry [80], while its absence halves Cyclin D1 levels [81]. In addition, Myc induces Cdk4, aiding the assembly of active Cyclin D1 / Cdk4,6 complexes [81,82]. |
| **CyclinD1** | ⊢ | **GSK3** | GSK-3β phosphorylates Cyclin D1 on Thr-286, promoting its ubiquitination and degradation [83]. |
| **CyclinD1** | ← | **CyclinD1** | In order to take into account both production and stability of Cyclin D1, we assumed that the presence of active CyclinD/Cdk2,4 complexes renders transcriptional maintenance of their levels easier. |
| **CyclinD1** | not **CHK1** and { [ not **p21** and ( {not **GSK3** and [**Myc** or **E2F1**]} or {**Myc** and **CyclinD1**} or {**Myc** and **E2F1**} or {**E2F1** and **CyclinD1**} ) ] or [ not **pRB** and **E2F1** and ( {**Myc** and **CyclinD1**} or {**Myc** and not **GSK3**} or {**CyclinD1** and not **GSK3**} ) ] } | | |

| Target Node | Link | Input | Description & References |
|---|---|---|---|
| | Ongoing DNA synthesis keeps the CHK1 kinase active, which inhibits CyclinD1. The precise regulatory logic of Cyclin D1 as a function of transcriptional control by Myc and E2F, combined with the regulation of its protein stability / activity by GSK3/ basal p21 is not known. Here, we assume that in the absence of p21 (once p21 levels drop due to growth factor signals and/or Cdk2 activation), Cyclin D1 can be activated by either Myc or E2F1 in the absence of GSK3. In the presence of GSK3, we assume that Cyclin D1 can be induced by the combined action of both Myc and E2F1 [84], but sustained in an ON state by either. In the presence of basal (normal quiescent) levels of p21, we assume that Cyclin D1 transcription requires E2F1 unencumbered by pRB, as well as any two of the following: Myc, already active Cyclin D1, and no GSK3. | | |
| **E2F1** | ⊢ | **CAD** | This link from Caspase-activated DNAase (CAD) to E2F1 ensures that apoptotic cells settle into an E2F1-negative attractor regardless of their initial state. The rationale for this is that E2F1 cannot maintain its activity if DNA is fragmented. |
| **E2F1** | ⊢ | **CyclinA** | The phosphorylation of the E2F1-binding DP-1 protein by Cyclin A, which binds directly to E2F1 (as well as E2F-2,3) down-regulates E2F1 transcriptional activity in S phase [85,86]. |
| **E2F1** | ⊢ | **pRB** | RB binds to E2F/DP1 complexes and switches their DNA binding activity from an activating to a repressive form [77,87]. |
| **E2F1** | ← | **E2F1** | E2F1 binds to its own promoter and up regulates transcription (as long as Cyclin D1/E activity blocks RB-E2F1 binding) [88]. |
| **E2F1** | ← | **Myc** | Myc is required for growth-factor mediated induction of E2F1 [56, 47]. It binds to and remodels the E2F1 promoter, facilitating E2F1 transcription [89]. In addition, Myc augments protein expression of E2F1 [90]. Single-cell experiments show that Myc is a critical modulator of the amplitude of E2F activation [91]. |
| **E2F1** | not (**CAD** or **CyclinA** or **pRB**) and (**E2F1** or **Myc**) | | |
| | In the absence of both CyclinA and pRB, E2F1 transcription can be induced by Myc or maintained by active E2F1. CAD deactivates E2F1 as it destroys the cell's DNA. | | |
| **CyclinE** | ← | **E2F1** | E2F1 is a potent transcriptional activator of Cyclin E [92]. |
| **CyclinE** | ← | **Cdc6** | Chromatin association and full activation of Cyclin E / Cdk2 requires Cdc6 [93]. |
| **CyclinE** | ← | **Pre-RC** | At the G1/S transition, Cyclin E is loaded onto chromatin by pre-RC complexes (Cdc6 and Cdt1 binding), where it is required for MCM2 loading, origin firing and the start of DNA synthesis [94]. In addition, activation of its partner Cdk2 by Cdc6 is contingent on this localization [93]. |
| **CyclinE** | ⊢ | **pRB** | CyclinE transcription by E2F1 is inhibited by hypo-phosphorylated (active) pRB protein [95]. |
| **CyclinE** | ⊢ | **p27Kip1** | p27Kip1 binds to Cyclin E / Cdk2 complexes and prevents their activation [53]. |
| **CyclinE** | ⊢ | **CHK1** | Chk1 activation during normal S-phase progression keeps Cdk2 activity in a physiological range by binding to both Cdk2 and Cdc25A, aiding the loss of Cyclin E / Cdk1 activity [96]. |

| Target Node | Link | Input | Description & References |
|---|---|---|---|
| **CyclinE** | ⊢ | **Casp3** | Caspase 3 cleaves and deactivates Cyclin E, which is then rapidly degraded [97]. |
| **CyclinE** | **E2F1** and **Cdc6** and **Pre-RC** and not (**pRB** or **p27Kip1** or **CHK1** or **Casp3**) | | |
| | In our model, the ON state of Cyclin E represents active Cyclin E / Cdk2 complexes. Thus, its full activation requires transcription via E2F1 not blocked by active pRB, binding to Cdc6 and pre-RC complexes, and the absence of its inhibitors p27Kip1, CHK1 and Caspase 3. | | |

**S1.C.** Boolean regulatory logic of Phase Switch nodes.

| Target Node | Link | Input | Description & References |
|---|---|---|---|
| **CyclinA_mRNA** | ⊢ | **CAD** | This link from Caspase-activated DNAase (CAD) to Cyclin A mRNA ensures that apoptotic cells settle into a G0-like attractor regardless of their initial state. The rationale for this is that no mRNA synthesis can be maintained if DNA is fragmented (we only use these links from CAD if needed). |
| **CyclinA_mRNA** | ← | **E2F1** | Cyclin A is transcriptionally activated by E2F factors [95]. |
| **CyclinA_mRNA** | ⊢ | **pRB** | Active RB blocks E2F1's ability to transcribe Cyclin A [98]. |
| **CyclinA_mRNA** | ← | **FoxM1** | Depletion of FoxM1 results in reduced Cyclin A2 expression (it is not clear whether FoxM1 is a direct transcriptional inducer of Cyclin A) [99-102]. |
| **CyclinA_mRNA** | not **CAD** and [ (**E2F1** and not **pRB**) or **FoxM1** ] | | |
| | In non-apoptotic cells Cyclin A is transcribed by E2F1 in the absence of active RB or by FoxM1. | | |
| **Emi1** | ← | **E2F1** | Emi1 is a direct transactional target of E2F1 [103,104]. |
| **Emi1** | ⊢ | **pRB** | Active retinoblastoma protein can block Emi1 transcription mediated by E2F1 [104]. |
| **Emi1** | ⊢ | **p21** | p21 activation during DNA damage lead to a substantial decrease of Emi1 levels, not observed in p21-null cells [105]. |
| **Emi1** | ⊢ | **Plk1** | Plk1 phosphorylates Emi1 at mitotic spindle poles, stimulating its βTrCP binding and ubiquitination [106]. |
| **Emi1** | ⊢ | **CyclinB** | Cyclin B / Cdk1 enhances the ability of Plk1 to mediate Emi1 destruction [106]. |
| **Emi1** | ⊢ | **Cdk1** | Cyclin B / Cdk1 enhances the ability of Plk1 to mediate Emi1 destruction [106]. |
| **Emi1** | ⊢ | **U_Kinetochores** | As Plk1-mediated phosphorylation of Emi1 occurs at mitotic spindle poles, our model requires ongoing mitosis for this interaction [106]. |
| **Emi1** | ⊢ | **A_Kinetochores** | As Plk1-mediated phosphorylation of Emi1 occurs at mitotic spindle poles, our model requires ongoing mitosis for this interaction [106]. |

| Target Node | Link | Input | Description & References |
|---|---|---|---|
| **Emi1** | (**E2F1** or not **pRB** or not **p21**) and not [**Plk1** and **CyclinB** and **Cdk1** and (**U_Kinetochores** or **A_Kinetochores**)] | | |
| | Our model allows the sustained presence of Emi1 protein when it is either actively transcribed by E2F1 or lacks joint inhibition by pRB and p21. Degradation of Emi1 is mediated by Plk1 and CyclinB/Cdk1 complexes. In addition, it requires at least temporary co-localization of Emi1 with Plk1 at mitotic spindle poles [106]. | | |
| **FoxM1** | ← | **Myc** | FoxM1 is a direct transcriptional target of c-Myc [107]. |
| **FoxM1** | ← | **CyclinE** | Cyclin E / Cdk2 complexes bind and phosphorylate FoxM1, potently inducing its transcriptional activity, which starts during S-phase [108]. |
| **FoxM1** | ← | **CyclinA** | In addition to Cyclin E / Cdk2, Cyclin A / Cdk2 complexes can also keep FoxM1 transcriptionally active by phosphorylating its autoinhibitory N-terminal region [109]. |
| **FoxM1** | ← | **Cdc25A** | Active Cdc25A enhances the transcriptional activity of FoxM1, likely via keeping a strong pool of active Cdk2 [110]. |
| **FoxM1** | ← | **Cdc25B** | Cdc25B overexpression can increase FoxM1-dependent transcription, likely via keeping aiding Cdk1 activity [111]. |
| **FoxM1** | ← | **CyclinB** | FoxM1 binds Plk1, and phosphorylation of two key residues in this domain by Cyclin B / Cdk1 primes it for Plk1 binding [112]. |
| **FoxM1** | ← | **Cdk1** | FoxM1 binds Plk1, and phosphorylation of two key residues in this domain by Cyclin B / Cdk1 primes it for Plk1 binding [112]. |
| **FoxM1** | ← | **Plk1** | Plk1 binds and phosphorylates FoxM1, which activates the FoxM1-mediated transcription in early mitosis [112]. |
| **FoxM1** | (**Myc** and **CyclinE**) or (**CyclinA** and **Cdc25A** and **Cdc25B**) or (**Plk1** and **CyclinB** and **Cdk1**) | | |
| | In our model, FoxM1 activity requires increased expression by Myc and activating phosphorylation by Cyclin E. Alternatively, FoxM1 activity can be sustained by potent Cdk2 / Cdk1 activity in G2 (supported by Cdc25A or Cdc25B), or a serial phosphorylation by Cyclin B / Cdk1 and Plk1 during mitosis. | | |
| **Cdc25A** | ← | **FoxM1** | FoxM1 is a direct transcriptional inducer of Cdc25A [110]. |
| **Cdc25A** | ← | **E2F1** | E2F1 is a direct transcriptional inducer of Cdc25A [113]. |
| **Cdc25A** | ⊢ | **pRB** | RB blocks E2F1's ability to drive Cdc25A transcription [113,114]. |
| **Cdc25A** | ⊢ | **Cdh1** | APC/C$^{Cdh1}$ degrades Cdc25A at mitotic exit [115]. |
| **Cdc25A** | ⊢ | **GSK3** | GSK3β phosphorylates Cdc25A, promoting its proteolysis [116]. |
| **Cdc25A** | ⊢ | **CHK1** | CHK1 phosphorylates Cdc25A, promoting its proteolysis and inhibiting its interaction with Cyclin B / Cdk1 [117]. |
| **Cdc25A** | ← | **CyclinE** | Cdc25A protein levels are stabilized during S-phase by CyclinE/ Cdk2 dependent phosphorylation [108]. |

| Target Node | Link | Input | Description & References |
|---|---|---|---|
| **Cdc25A** | ← | **CyclinA** | Cdc25A protein levels are stabilized during S and G2 by Cdk2-dependent phosphorylation. Cdk2 first partners with Cyclin E [118], then continues to stabilize Cdc25A past the point of Cyclin E expression by partnering with Cyclin A [119]. |
| **Cdc25A** | ← | **Cdk1** | During mitosis, Cdc25A is stabilizes by Cyclin B / Cdk1 phosphorylation, which protects it from the proteasome [120]. |
| **Cdc25A** | ← | **CyclinB** | During mitosis, Cdc25A is stabilizes by Cyclin B / Cdk1 phosphorylation, which protects it from the proteasome [120]. |
| **Cdc25A** | { (**FoxM1** and **E2F1** and not **pRB**) or [ not **Cdh1** and {**FoxM1** or (**E2F1** and not **pRB**) } ] } and [not (**GSK3** or **CHK1**) or **CyclinE** or **CyclinA** or (**CyclinB** and **Cdk1**) ] | | |
| | As the precise combinatorial regulation of Cdc25A throughput the cell cycle is unknown, our model assumes that accumulation of the Cdc25A protein requires transcriptional activation by both E2F1 in the absence of pRB and FoxM1 to override destruction by $APC/C^{Cdh1}$. Alternatively, one of the two transcription factors can drive Cdc25A accumulation in the absence of $APC/C^{Cdh1}$. In addition, stabilization of Cdc25A either requires the absence of GSK3 and CHK1 (both of which promote its degradation), or stabilization by Cdk activity. | | |
| **CyclinA** | ← | **CyclinA_mRNA** | Our model, sustained availability of Cyclin A requires transcription. |
| **CyclinA** | ⊢ | **pAPC** | Cyclin A is degraded by the $APC/C^{Cdc20}$ in prometaphase (as soon as the APC/C components are phosphorylated by Cdk1) [121,122], before the full activation of the complex at SAC passage [123]. In our model, this stage of mitotic $APC/C^{Cdc20}$ activation is represented by Cdk1-phosphorylated APC/C (pAPC). |
| **CyclinA** | ← | **Cdc25A** | Cdc25A promotes active Cyclin A / Cdk2 complex formation by removing inhibitory phosphorylation from Cdk2 [124,125]. |
| **CyclinA** | ⊢ | **Cdh1** | Cyclin A is degraded by $APC/C^{Cdh1}$ in the presence of the UbcH10 protein [121,126]. |
| **CyclinA** | ← | **Emi1** | Emi1 binding to Cdh1 is required to stabilize Cyclin A levels at the G1/S transition, allowing Cyclin A / Cdk2 to block Cdh1 [127-129]. |
| **CyclinA** | ⊢ | **UbcH10** | Cyclin A degradation by $APC/C^{Cdh1}$ requires UbcH10 [126]. |
| **CyclinA** | ← | **CyclinA** | We assume that once activated, Cyclin A / Cdk2,1 complexes can sustain their activity under favorable conditions until Cyclin A is degraded. |
| **CyclinA** | **CyclinA_mRNA** and not **pAPC** and { [**Cdc25A** and (not **Cdh1** or **Emi1**) ] or [**CyclinA** and ( {not **Cdh1** and [**Emi1** or not **UbcH10**] } or {**Emi1** and not **UbcH10**} ) ] } | | |

| Target Node | Link | Input | Description & References |
|---|---|---|---|
| | Cyclin A activity requires transcription (Cyclin_A mRNA) and the absence of degradation by phosphorylated (mitotic) pAPC. In addition, turning ON inactive Cyclin A requires activation of Cdk2 by Cdc25A [124] and the absence / Emi1-mediated inhibition of APC/C$^{Cdh1}$. Once active, Cyclin A maintains its activity in the absence of overpowering influences driving its degradation. Namely, Cyclin A relies on either Emi1 or the absence of UbcH10 for its ability to keep inactive APC/C$^{Cdh1}$ in check. To overpower active APC/C$^{Cdh1}$, Cyclin A requires both Emi1 and no UbcH10. The precise combinatorial regulation of Cyclin A is not known; the above logic is consistent with Cyclin A activity pattern during cell cycle progression. | | |
| **Wee1** | ⊢ | **Casp3** | Caspase 3 cleaves and deactivates Wee1 [130]. |
| **Wee1** | ← | **CHK1** | During DNA replication Wee1 is activated by the checkpoint kinase Chk1 [131]. |
| **Wee1** | ← | **Replication** | To model the sensitivity of Wee1 activation to ongoing DNA synthesis even in the absence of damage, our model turns on Wee1 immediately upon the start of DNA replication and maintains it until both Replication and the checkpoint kinase Chk1 is OFF [131]. In addition, Wee1 activity has been implicated in maintaining normal replication fork procession, linking its activity directly to ongoing replication [132]. |
| **Wee1** | ⊢ | **Cdk1** | The somatic Wee1 protein is an order of magnitude more sensitive to Cdk1 activity than Cdc25C. Thus, both Cyclin B / Cdk1 and Cyclin A / Cdk1 induce Wee1 phospho- rylation and deactivation [133,134]. |
| **Wee1** | ⊢ | **CyclinB** | Cyclin B / Cdk1 is a strong inducer of Wee1 phosphorylation and deactivation [133,134]. |
| **Wee1** | ⊢ | **CyclinA** | Cyclin A / Cdk1 can also induce Wee1 phosphorylation and deactivation [133,134]. |
| **Wee1** | ⊢ | **Plk1** | Plk1 phosphorylation at S53 promotes Wee1 degradation [133]. This event is primed by Cdk1 phosphorylation of Wee1 at S123 [133]. As the main partner of Cdk1 in mitosis is Cyclin B, we assume that assistance from Plk1 to block Wee1 is more relevant when paired with Cyclin A / Cdk1 complexes. |
| **Wee1** | not **Casp3** and (**Replication** or **CHK1**) and not (**Cdk1** and **CyclinB**) and [**CHK1** or not (**Cdk1** and **CyclinA** and **Plk1**) ] | | |
| | Wee1 is active in non-apoptotic cells undergoing DNA synthesis or subject to the single-strand damage checkpoint signaled by active CHK1. That said, Wee1 is blocked by Cyclin B / Cdk1 phosphorylation or Cyclin A / Cdk1 / Plk1 in the absence of CHK1. | | |
| **UbcH10** | ⊢ | **Cdh1** | UbcH10 is degraded by APC/C$^{Cdh1}$ [84]. |
| **UbcH10** | ← | **UbcH10** | Active UbcH10 cannot be autoubiquitinated in the presence of APC/C$^{Cdh1}$ substrates and thus remains active [84]. |
| **UbcH10** | ← | **Cdc20** | The presence of APC/C$^{Cdh1}$ substrates, including Cdc20, inhibit the autoubiquitination of UbcH10 but not its function, thus preserving APC activity [84]. |

| Target Node | Link | Input | Description & References |
|---|---|---|---|
| **UbcH10** | ← | **CyclinA** | The presence of APC/C$^{Cdh1}$ substrates, including Cyclin A, inhibit the autoubiquitination of UbcH10 but not its function, thus preserving APC activity [84]. |
| **UbcH10** | ← | **CyclinB** | The presence of APC/C$^{Cdh1}$ substrates, including CyclinB, inhibit the autoubiquitination of UbcH10 but not its function, thus preserving APC activity [84]. |
| **UbcH10** | not **Cdh1** or [**UbcH10** and (**Cdc20** or **CyclinA** or **CyclinB**) ] | | |
| | The ubiquitin-conjugating enzyme (E2) UbcH10 is active in the absence of Cdh1. Alternatively, active UbcH10 is maintained in the presence of Cdh1 when some of its targets are present: Cdc20 OR CyclinA OR CyclinB [126]. | | |
| **CyclinB** | ← | **FoxM1** | FoxM1 is a direct transcriptional regulator of Cyclin B1 [102,135]. |
| **CyclinB** | ← | **FoxO3** | FoxO3 is a direct transcriptional regulator of Cyclin B; its activation in G2 helps increase/maintain Cyclin B levels [136]. |
| **CyclinB** | ← | **CyclinB** | Here we assume that FoxO3 alone can only maintain, but not independently induce Cyclin B1 expression. |
| **CyclinB** | ⊢ | **Cdh1** | Cyclin B is degraded by APC/C$^{Cdh1}$ [137]. |
| **CyclinB** | ⊢ | **pAPC** | Cyclin B is degraded by APC/C$^{Cdc20}$ [137]. |
| **CyclinB** | ⊢ | **Cdc20** | Cyclin B is degraded by APC/C$^{Cdc20}$ [137]. |
| **CyclinB** | [**FoxM1** or (**FoxO3** and **CyclinB**) ] and not [**Cdh1** or (**pAPC** and **Cdc20**) ] | | |
| | Cyclin B node is ON when the concentration of Cyclin B proteins is high (does not represent the activity of CyclinB/Cdk1 complexes). This occurs when Cyclin B is transcribed by FoxM1, maintained by FoxO3 transcription, and not undergoing APC-mediated degradation by APC/C$^{Cdc20}$ or APC/C$^{Cdh1}$. | | |
| **Cdc25B** | ← | **FoxM1** | FoxM1 is an essential inducer or Cdc25B [138]. |
| **Cdc25B** | ← | **4N_DNA** | Cdc25B is localized at centrosomes, where it is activated by Aurora A kinase [139]. As Aurora A itself is only recruited to duplicated, centrosomes before their separation [140], Cdc25B activation requires duplicated centrosomes. As our model does not directly account for centrosome dynamics, we account for this by requiring the completion of S-phase (4N_DNA). |
| **Cdc25B** | **FoxM1** and **4N_DNA** | | |
| | Cdc25B activation requires transcription by FoxM1, centrosomal localization, and activation by Aurora A kinase on replicated centrosomes. | | |
| **Plk1** | ⊢ | **Cdh1** | The majority of Plk1 is degraded in anaphase by the APC/C$^{Cdh1}$ complex [141] |
| **Plk1** | ← | **FoxM1** | Plk1 is a direct transcriptional target of FoxM1 [112]. |

| Target Node | Link | Input | Description & References |
|---|---|---|---|
| **Plk1** | ← | **Plk1_H** | Our model tracks the accumulauton of high-enough levels of Plk1 to survive $APC/C^{Cdh1}$ mediated destruction into telophase via the Plk1_H node. Its ON state represents strong prior Plk1 activation. Thus, it sustains the Plk1 node in the absence of FoxM1-mediated transcription until Plk1_H itself is lost as Plk1 levels fall. |
| **Plk1** | ← | **CyclinB** | Plk1 is activated by Cyclin B / Cdk1 phosphorylation [142-144]. |
| **Plk1** | ← | **Cdk1** | Plk1 is activated by Cyclin B / Cdk1 phosphorylation [142-144]. |
| **Plk1** | ← | **CyclinA** | Plk1 activation at the G2/M boundary, before Cdk1/Cuclin B complexes are activated, requires active Cyclin A / Cdk [145]. |
| **Plk1** | ⊢ | **Wee1** | Cyclin A-mediated induction of Plk1 is blocked by Wee1 kinase, which specifically inhibits Cdk2 activity [145]. |
| **Plk1** | ← | **Cdc25A** | As we do not include a separate Cdk2 node in our model, strong Cyclin A / Cdk2 activity requires ongoing dephosphorylation of Cdk2 by Cdc25A [125]. |
| **Plk1** | not **Cdh1** and (**FoxM1** or **Plk1_H**) and [ (**CyclinB** and **Cdk1**) or (**CyclinA** and not **Wee1** and **Cdc25A**) ] | | |
| | Plk1 activity requires the absence of $APC/C^{Cdh1}$, transcription by FoxM1, or high Plk1 levels transcribed earlier by both FoxM1 and FoxO3 (see Plk1_H below) [136]. In addition, Plk1 activation requires phosphorylation by either CyclinB/Cdk1 during mitosis or Cyclin A / Cdk2 (aided by lack of Wee1 and Cdc25A) at the G2/M boundary [145]. | | |
| **Cdc25C** | ← | **Plk1** | Plk1-mediated phosphorylation of Cdc25C before mitotic entry is required as the initiating step of the Cdc25C / Cdk1 feedback loop [145]. In addition, Plk1 induces nuclear transport of CDC25B, where it contributes to the initiation of Cdk1 activity [146]. During mitosis, Plk1 helps maintain strong Cdc25C activation by phosphorylating it on the same site as Cyclin B / Cdk1 [147], as indicated by the profound decrease of Cdc25C activity in Plk1-inhibited mitotic cells [143,148]. |
| **Cdc25C** | ← | **Cdc25B** | CDC25B starts the cascade leading to mitotic entry by activating a small centrosomal pool of Cyclin B / Cdk1, leading to their nuclear translocation where they trigger the activation of Cdc25C and eventually the larger nuclear Cyclin B / Cdk1 pool [149-151]. |
| **Cdc25C** | ⊢ | **CHK1** | Chk1 phosphorylates Cdc25C, leading to its nuclear exclusion, loss of access to its main target, Cdk1 [152]. In addition, CHK1 blocks the ability of Cdc25B to activate Cdc25C at the centrosomes by phosphorylating it and blocking it's Cdk1 activity [153,154]. |
| **Cdc25C** | ← | **Cdk1** | Cyclin B / Cdk1 are potent Cdc25C activators, creating positive feedback that causes switch-like mitotic entry [155]. |
| **Cdc25C** | ← | **CyclinB** | Cyclin B / Cdk1 are potent activators of Cdc25C [155]. |

| Target Node | Link | Input | Description & References |
|---|---|---|---|
| **Cdc25C** | ← | **4N_DNA** | The nature and localization of the signals responsible for the onset and maintenance of Cdc25C activity require replicated DNA (4N_DNA) [146,151]. Namely, Cdc25C is initially activated by a small pool of Cyclin B / Cdk1 (below the ON-threshold of Cdk1 in our model) which starts our at the replicated centrosome (see above). Moreover, the pool of mitotic Cdc25C co-localized with active Chk1/Cyclin B is found on condensed chromosomes, again requiring the presence of 4N_DNA [152]. |
| **Cdc25C** | **4N_DNA** and **Plk1** and [ (**Cdc25B** and not **CHK1**) or (CyclinB and **Cdk1**) ] | | |
| | In our model, Cdc25C is active in cells with replicated DNA (see 4N_DNA → Cdc25C link). Its activation is initiated by a small, initially cytoplasmic pool of Cyclin B / Cdk1 activated by Cdc25B (not directly represented in our model) and further increased by Cdc25B itself, which translocates to the nucleus with the aid of Plk1. During mitosis, Plk1 potentiates the ability of Cyclin B / Cdk1 to maintain Cdc25C activity. | | |
| **Cdk1** | ← | **CyclinB** | Full kinase activation of Cdk1 in our model requires it to complex with Cyclin B [156]. |
| **Cdk1** | ← | **Cdc25C** | Cdk1 is subject to inhibitory phosphorylation by Wee1 or Myt1, while its dephosphorylation is carried out by activated Cdc25C [155,156]. |
| **Cdk1** | ← | **Cdk1** | We assume that the presence of fully activated, nuclear Cdk1 is able to overcome the effect of active Wee1 (as long as CHK1 is off), given that Wee1 is very sensitive to Cdk1-mediated inhibitory phosphorylation [134]. |
| **Cdk1** | ⊢ | **Wee1** | Wee1 is a nuclear protein that ensures the completion of DNA replication prior to mitosis by blocking Cdk1 activation [157]. |
| **Cdk1** | ⊢ | **CHK1** | In the absence of CHK1 kinase, a small cytosolic (centrosomal) pool of Cyclin B / Cdk1 can be activated by Cdc25B, the nuclear translocation of which can trigger a positive feedback loop that activates the full Cdk1 pool (assuming nuclear Wee1 is also inactive). Thus, CHK1 can maintain the OFF state of inactive Cdk1 [154]. |
| **Cdk1** | **CyclinB** and **Cdc25C** and [ not **CHK1** or (not **Wee1** and **Cdk1**) ] | | |
| | Full Cdk1 kinase activation requires its binding partner Cyclin B and the Cdc25C phosphatase, which maintains Cdk1 in an active dephosphorylated state. Cdk1 is inhibited by the checkpoint kinase CHK1, unless it is already full active and Wee1 kinase is inhibited. | | |
| **pAPC** | ← | **CyclinB** | CyclinB/Cdk1 activation promotes APC/C$^{Cdc20}$ activity via APC/C subunit phosphorylation [158]. |
| **pAPC** | ← | **Cdk1** | CyclinB/Cdk1 activation promotes APC/C$^{Cdc20}$ activity via APC/C subunit phosphorylation [158]. |
| **pAPC** | ← | **Plk1** | In addition to Cyclin B / Cdk1 phosphorylation, full activation of the APC/C$^{Cdc20}$ complex also requires the kinase activity of Plk1 [159]. |

| Target Node | Link | Input | Description & References |
|---|---|---|---|
| **pAPC** | ← | **pAPC** | Activated $APC/C^{Cdc20}$ initiates the Metaphase ! Anaphase transition by degrading Cyclin B and securing [87, 68]. Once active, $APC/C^{Cdc20}$ no longer requires sustained CyclinB/Cdk1 phosphorylation. |
| **pAPC** | ← | **Cdc20** | Once catalytically active, $APC/C^{Cdc20}$ no longer requires ongoing CyclinB/Cdk1 or Plk1 phosphorylation. |
| **pAPC** | (**CyclinB** and **Cdk1** and **Plk1**) or (**CyclinB** and **Cdk1** and **pAPC**) or (**pAPC** and **Cdc20**) | | |
| | In line with evidence that Plk1 can aid full activation of APC/C, but Cdk1 appears to be the more potent inducer, our model requires both Cyclin B / Cdk1 and Plk1 to activate APC/C from an OFF state, but only Cdk1 activity to maintain it. In addition, ongoing phosphorylation of the functional $APC/C^{Cdc20}$ complex is no longer required. | | |
| **Cdc20** | ⊢ | **Cdh1** | $APC/C^{Cdh1}$ complexes degrade Cdc20, leading to a complete switch from $APC/C^{Cdc20}$ to $APC/C^{Cdh1}$ during mitotic exit [160,161]. |
| **Cdc20** | ⊢ | **Cdk1** | Cdk1-phosphorylated Cdc20 interacts with Mad2 rather than APC/C, resulting in a block on $APC/C^{Cdc20}$ activation until completion of spindle assembly [162]. |
| **Cdc20** | ⊢ | **CyclinB** | Cyclin B partners with Cdk1 to keep Cdc20 phosphorylated, increasing its interaction with Mad2 rather than APC/C [162]. |
| **Cdc20** | ⊢ | **CyclinA** | Cyclin A / Cdk2 complexes phosphorylate Cdc20 and inactivate the $APC/C^{Cdc20}$ complex during S and G2 [163]. |
| **Cdc20** | ⊢ | **Emi1** | Emi1 binds Cdc20 and inhibits the ubiquitin ligase activity of $APC/C^{Cdc20}$ [128]. |
| **Cdc20** | ⊢ | **Mad2** | The physical alignment of chromosomes along the mitotic spindle is monitored by Mad checkpoint proteins [164]. In cells with a normal spindle assembly checkpoint (SAC), even a single unattached, Mad2-bound kinetochore can sequester Cdc20 and thus inhibit $APC/C^{Cdc20}$ [165]. |
| **Cdc20** | ← | **pAPC** | Cdc20 becomes active in early mitosis by binding to APC/C, an event that requires Cyclin B / Cdk1 phosphorylation of several APC/C subunits [160,166]. |
| **Cdc20** | **pAPC** and not **Emi1** and not **Cdh1** and { not **Mad2** or [ not **CyclinA** and not (**CyclinB** and **Cdk1**) ] } | | |
| | In our model, $APC/C^{Cdc20}$ complex formation is represented by the joint activity of Cdc20 and phosphorylated APC/C (pAPC). Cdc20 is thus ON in the presence of pAPC when both Emi1 and Cdh1 are absent ($APC/C^{Cdh1}$ is represented by the Cdh1 node, see below). In addition, Cdc20 activity requires either the absence of Mad2 at unattached kinetochores, or the absence of Cdc20 phosphorylation by Cyclin B / Cdk1 or by Cyclin A / Cdk2 complexes to potentiate the interaction between Mad2 and Cdc20. and pAPC is ON (present and phosphorylated) [162]. | | |
| **Cdh1** | ⊢ | **CyclinB** | Cyclin B / Cdk1 complexes phosphorylate Cdh1, blooming its ability to bind APC/C and inhibiting $APC/C^{Cdh1}$ activity [167]. |
| **Cdh1** | ⊢ | **Cdk1** | Cyclin B / Cdk1 complexes phosphorylate Cdh1, blooming its ability to bind APC/C and inhibiting $APC/C^{Cdh1}$ activity [167]. |

| Target Node | Link | Input | Description & References |
|---|---|---|---|
| **Cdh1** | ⊢ | **CyclinA** | Active Cyclin A / Cdk1,2 complexes phosphorylate Cdh1 during S, G2 and early mitosis, impairing its interaction with APC/C until late stages of mitosis when Cdk1/2 activity falls [161]. |
| **Cdh1** | ⊢ | **Cdc25A** | As we do not include a separate Cdk2 node in our model, strong Cyclin A / Cdk2 activity capable of overriding Cdh1 activity even in the presence of Emi1 requires ongoing dephosphorylation of Cdk2 by Cdc25A [125]. |
| **Cdh1** | ⊢ | **Emi1** | Emi1 blocks APC/C$^{Cdh1}$ binding to its substrates [129], as well as its ability to add ubiquitin chains to them [168]. |
| **Cdh1** | not (**CyclinB** and **Cdk1**) and not [ **CyclinA** and (**Emi1** or **Cdc25A**) ] | | |
| | APC/C$^{Cdh1}$ activity requires the absence of Cyclin Dependent kinase phosphorylation by Cyclin B / Cdk1, or Cyclin A / Cdk2 aided by further inhibition of Cdh1 by Emi1, or ongoing Cdk2 activation by Cdc25A in the absence of Emi1. | | |

**S1.D.** Boolean regulatory logic of Origin of Replication Licensing Switch nodes.

| Target Node | Link | Input | Description & References |
|---|---|---|---|
| **ORC** | ⊢ | **E2F1** | Expression of the ORC1 gene is regulated by E2F1 [169]. |
| **ORC** | ← | **Pre-RC** | Licensed but not yet fired replication complexes (Pre-RCs containing ORC, Cdc6, Cdt1 and inactive MCMs) remain stable at sites of replication origin until fired by the activation of the MCM helicase [170]. |
| **ORC** | ← | **Cdc6** | Availability of stable (unphosphorylated) Cdc6 in the Pre-RC is necessary for the maintenance of licensed origins [170]. |
| **ORC** | ← | **Cdt1** | Active (unphosphorylated and not geminin-bound) Cdt1 bound to the Pre-RC is necessary for the maintenance of licensed origins [170]. |
| **ORC** | **E2F1** or (**Pre-RC** and **Cdt1** and **Cdc6**) | | |
| | ORC proteins can bind at origins of replication when transcribed by E2F1 or as part of a fully assembled and licensed Pre-RC complex (including active Cdc6 and Cdt1). | | |
| **Cdc6** | ⊢ | **Casp3** | Caspase 3 cleaves and deactivates Cdc6 [171]. |
| **Cdc6** | ⊢ | **CyclinA** | Phosphorylation of CDC6 by Cyclin A / Cdk2 during DNA replication leads to its re-localization to the cytoplasm [172]. |
| **Cdc6** | ⊢ | **4N_DNA** | In our model, full deactivation of Cdc6 represents the firing of all ORCs as DNA replication is completed. Thus Cyclin A's inhibitory action takes full effect once the cell reaches 4N DNA content [172]. |
| **Cdc6** | ← | **E2F1** | Transcription of Cdc6 is directly induced by E2F1 [173] |
| **Cdc6** | ← | **ORC** | ORC recruits Cdc6 to origins of replication [170]. |

<table>
<tr><th>Target Node</th><th>Link</th><th>Input</th><th>Description & References</th></tr>
<tr><td>Cdc6</td><td>⊢</td><td>Plk1</td><td>Plk1 binds, phosphorylated and strongly recruits Cdc6 to the spindle pole during metaphase, then to the central spindle in anaphase, leading to its exclusion from chromosomes until telophase, when the majority of Plk1 is degraded by APC/C$^{Cdh1}$ [174].</td></tr>
<tr><td>Cdc6</td><td>←</td><td>Pre-RC</td><td>Licensed but not yet fired replication complexes (Pre-RCs containing ORC, Cdc6, Cdt1 and inactive MCMs) remain stable and Cdc6-bound until fired by the activation of the MCM helicase [170].</td></tr>
<tr><td>Cdc6</td><td>←</td><td>Cdc6</td><td>Stable (unphosphorylated) Cdc6 in the Pre-RC is necessary for the maintenance of licensed origins [170].</td></tr>
<tr><td>Cdc6</td><td>←</td><td>Cdt1</td><td>Active (unphosphorylated and not geminin-bound) Cdt1 bound to the Pre-RC is necessary for the maintenance of licensed origins [170].</td></tr>
<tr><td>Cdc6</td><td colspan="3">not Casp3 and not (4N_DNA and CyclinA) and [ (E2F1 and ORC and not Plk1) or (Pre-RC and ORC and Cdc6 and Cdt1) ]</td></tr>
<tr><td></td><td colspan="3">In our model the Cdc6 node represents nuclear, chromatin-bound Cdc6. Thus, the node is only active during the assembly of pre-replication complexes, or their ongoing presence during DNA replication. Cdc6 is ON in the absence of Caspase 3 or CyclinA / Cdk2 phosphorylation of Cdc6 in all origins required for the completion of DNA replication (thus, its inhibition by Cyclin A also requires 4N_DNA). In addition, active Cdc6 requires either transcription by E2F1 and recruitment by origin-bound ORC proteins in the absence of mitotic Plk1 or maintenance of Pre-RCs by the presence of all of its components.</td></tr>
<tr><td>Cdt1</td><td>←</td><td>ORC</td><td>ORC-bound origin of replication sites are the point of pre-replication complex assembly, where Cdt1 is recruited by ORC-bound Cdc6 [170].</td></tr>
<tr><td>Cdt1</td><td>←</td><td>Cdc6</td><td>ORC-bound Cdc6 recruits Cdt1 to pre-RC complexes [170].</td></tr>
<tr><td>Cdt1</td><td>⊢</td><td>CyclinE</td><td>Sustained Cdk2 activity during S-phase, responsible for the firing of all origins required to complete DNA synthesis (modeled as simultaneous Cyclin E, Cyclin A and Cdc25A activity), also leads to the phosphorylation and proteasomal degradation of Cdt1 [170].</td></tr>
<tr><td>Cdt1</td><td>⊢</td><td>CyclinA</td><td>Sustained Cdk2 activity leads to phosphorylation and degradation of Cdt1 (see above) [170].</td></tr>
<tr><td>Cdt1</td><td>⊢</td><td>Cdc25A</td><td>Sustained Cdk2 activity leads to phosphorylation and degradation of Cdt1 (see above) [170].</td></tr>
<tr><td>Cdt1</td><td>←</td><td>Pre-RC</td><td>Licensed but not yet fired replication complexes (Pre-RC) remain stable until fired during DNA replication [170].</td></tr>
<tr><td>Cdt1</td><td>←</td><td>Myc</td><td>Cdt1 is a direct transcriptional target of the Myc-Max complex [175], ensuring its availability for Pre-RC formation and maintenance.</td></tr>
<tr><td>Cdt1</td><td>←</td><td>E2F1</td><td>Cdt1 is a direct transcriptional target of E2F1 [176], ensuring its availability for Pre-RC formation and maintenance.</td></tr>
</table>

| Target Node | Link | Input | Description & References |
|---|---|---|---|
| **Cdt1** | ⊢ | **pRB** | E2F1-mediated transcription of Cdt1 is blocked by hypo-phosphorylated (active) pRB [176]. |
| **Cdt1** | ⊢ | **geminin** | Geminin binds to Cdt1 at pre-replication complexes, where it blocks Cdt1 binding to DNA, sequestering it away from Pre-RCs [177]. |
| **Cdt1** | not **geminin** and **ORC** and **Cdc6** and not (**CyclinE** and **CyclinA** and **Cdc25A**) and { [**Pre-RC** and (**E2F1** or **Myc**)] or [**E2F1** and (**Myc** or not **pRB**) ] } | | |
| | Replication-origin bound Cdt1 requires the absence of geminin, the presence of origin-bound ORC and Cdc6, and the absence of sustained Cdk2 activity responsible for the firing of all origins during DNA synthesis (modeled as simultaneous Cyclin E, Cyclin A and Cdc25A activity). Bound into a licensed pre-replication complex (Pre-RC), Cdt1 remains stable as long as it is transcribed by E2F1 or Myc (this guarantees that Pre-RC complexes cannot persist indefinitely in the absence of *de novo* transcription). Alternatively, it can be turned on by E2F1, aided by Myc or the absence of RB, and FoxO3 in cells with 4N_DNA. | | |
| **Pre-RC** | ← | **ORC** | Pre-RC complexes assemble when ORC, Cdc6 and Cdt1 are all bound to sites of replication origin along the DNA [170]. |
| **Pre-RC** | ← | **Cdc6** | Pre-RC complexes assemble when ORC, Cdc6 and Cdt1 are all bound to sites of replication origin along the DNA [170]. |
| **Pre-RC** | ← | **Cdt1** | Pre-RC complexes assemble when ORC, Cdc6 and Cdt1 are all bound to sites of replication origin along the DNA, leading to the recruitment of the MCM helicase [170]. |
| **Pre-RC** | ⊢ | **Replication** | Pre-RCs fire and fall apart during DNA replication [170]. |
| **Pre-RC** | ⊢ | **4N_DNA** | In our model Pre-RC turns OFF when Replication is completed, marked by the time-point when both Replication and 4N_DNA are ON. |
| **Pre-RC** | **ORC** and **Cdc6** and **Cdt1** and not (**Replication** and **4N_DNA**) | | |
| | Pre-RC complexes assemble when ORC, Cdc6, and Cdt1 are all bound to sites of replication origin along the DNA. The node denoting their licensing turns OFF at the moment of transition from ongoing Replication to 4N_DNA (it is blocked in the one time-point when both of these nodes are ON). | | |
| **geminin** | ← | **E2F1** | Geminin is a direct transcriptional target of E2F1 [176]. |
| **geminin** | ⊢ | **Cdh1** | Geminin is a target of APC/C$^{Cdh1}$ ubiquitin ligase [178]. |
| **geminin** | ⊢ | **pAPC** | Geminin is a target of APC/C$^{Cdc20}$ at the metaphase/anaphase transition [179]. |
| **geminin** | ⊢ | **Cdc20** | Geminin is a target of APC/C$^{Cdc20}$ at the metaphase/anaphase transition [179]. |
| **geminin** | **E2F1** and not **Cdh1** and not (**pAPC** and **Cdc20**) | | |
| | Geminin is present when transcribed by E2F1 and not targeted for degradation by APC/CCh1 or APC/C$^{Cdc20}$. | | |

**S1.E.** Boolean regulatory logic of Cell Cycle Process nodes.

| Target Node | Link | Input | Description & References |
|---|---|---|---|
| **Replication** | ⊢ | **CAD** | Caspase-activated DNAase (CAD) destroys DNA, preventing ongoing replication. |
| **Replication** | ← | **Pre-RC** | Ongoing DNA replication requires licensed replication origins, which fire throughout DNA synthesis [180]. |
| **Replication** | ← | **E2F1** | In addition to E2F1 target genes directly included in our model, E2F1 transcribes an array of critical S-phase genes responsible for carrying out DNA synthesis (e.g, POLA1, POLA2, MCM3, MCM5, MCM6, PCNA, TOP2A, RFC2, TK1) [181,182]. |
| **Replication** | ← | **CyclinE** | DNA replication is initiated by fully active CyclinE/Cdk2 [183]. |
| **Replication** | ← | **Cdc25A** | Active Cdc25A is required for onset as well as progression through S-phase [184,185]. |
| **Replication** | ← | **CyclinA** | Cyclin A / Cdk1 complexes regulate the origin firing program in mammalian cells and are required for the completion of DNA replication [185,186]. |
| **Replication** | ← | **Replication** | Once ongoing, DNA synthesis continues in the presence of active Cyclin A / Cdk2, only ending when DNA content is doubled. |
| **Replication** | ⊢ | **4N_DNA** | Complete duplication of a cell's DNA, represented in our model by 4N DNA = ON, marks the end of active Replication. |
| **Replication** | not **CAD** and **Pre-RC** and { (**E2F1** and **CyclinE** and **Cdc25A**) or (**Replication** and **CyclinA** and **Cdc25A** and (**E2F1** or not **4N_DNA**) ]} | | |
| | The Replication node represents ongoing DNA synthesis. This requires a non-apoptotic cell, licensed pre-replication complexes (Pre-RC). The start of DNA synthesis requires E2F1-meditated transcription of the genes that help execute it, as well as the firing of the first round of replication origins by Cyclin E / Cdk2. Once ongoing, replication is sustained by Cyclin A / Cdk2 and Cdc25A, aided by E2F1 and terminated by completion of a full round of synthesis (4N_DNA). | | |
| **ATR** | ← | **Replication** | ATR accumulates at replication forks during unperturbed DNA synthesis [187]. |
| **CHK1** | ← | **ATR** | ATR kinase activates CHK1 at replication forks, which not only blocks premature mitosis but also regulates the rate of origin firing by keeping Cdc25 protein levels from increasing above their physiological range [187] |
| **4N_DNA** | ⊢ | **CAD** | Caspase-activated DNAase (CAD) destroys DNA, preventing maintenance of a double DNA content. |
| **4N_DNA** | ← | **Replication** | DNA content is doubled by the process of Replication. |
| **4N_DNA** | ← | **Pre-RC** | Replication can only complete DNA synthesis and produce double DNA content if the availability of licensed replication origins is not blocked [180]. |
| **4N_DNA** | ← | **CyclinA** | Cyclin A / Cdk1 complexes regulate the origin firing program in mammalian cells and are required for the completion of DNA replication [183,186]. |

| Target Node | Link | Input | Description & References |
|---|---|---|---|
| **4N_DNA** | ← | **4N_DNA** | Once achieved, a cell's 4N DNA content is sustained up to the point of cytokinesis. |
| **4N_DNA** | ⊢ | **Ect2** | At the start of cytokinesis, the Ect2 RhoGEF is recruited to the central spindle [188]. Ect2 aids the accumulation of GTP-bound RhoA [189] and the formation of the contractile ring. In our model, Ect2 recruitment to the central spindle marks the start of cytokinesis and the subsequent resetting of daughter cell DNA content to 2N. |
| **4N_DNA** | not **CAD** and { [**Replication** and ({**Pre-RC** and **CyclinA**} or **4N_DNA**) ] or (**4N_DNA** and not **Ect2**)} | | |
| | 4N DNA content in our model is reached via the completion of Replication (via the firing of the last round of replication origins by Cyclin A / Cdk complexes) and maintained in non-apoptotic cells the absence of a contractile ring (marked by Ect2), driving cytokinesis. | | |
| **U_Kinetochores** | ← | **4N_DNA** | Metaphase requires replicated sister chromatids (4N_DNA), held together by their kinetochores, face in opposing directions and can be attached to opposite poles of the mitotic spindle. |
| **U_Kinetochores** | ⊢ | **Cdh1** | Premature activation of $APC/C^{Cdh1}$ destroys the incomplete spindle by triggering premature, aberrant anaphase. This occurs due to premature degradation of APC/C targets including Securin (responsible for keeping sister chromatids attached [190]), Cyclin B, CDC20, and Aurora kinase A (AURKA) [191]. |
| **U_Kinetochores** | ⊢ | **A_Kinetochores** | In our model, the transition from unattached (U_Kinetochores) to all attached kinetochores (A_Kinetochores) marks the completion of the mitotic spindle and Spindle Assembly Checkpoint (SAC) passage. |
| **U_Kinetochores** | ← | **CyclinB** | The start of mitotic spindle assembly is initiated by active CyclinB/Cdk1 [192]. |
| **U_Kinetochores** | ← | **Cdk1** | The start of mitotic spindle assembly is initiated by active CyclinB/Cdk1 [192]. |
| **U_Kinetochores** | ← | **U_Kinetochores** | Once metaphase starts, the mitotic spindle remains incomplete as long as some of the kinetochores remain unattached. |
| **U_Kinetochores** | **4N_DNA** and not **Cdh1** and not **A_Kinetochores** and [(**CyclinB** and **Cdk1**) or **U_Kinetochores**] | | |
| | The U_Kinetochores node in our model is on from the moment the nuclear envelope is dissolved in prometaphase and the mitotic spindle starts to form, until all kinetochores are properly attached. In addition to the presence of unattached kinetochores, U_Kinetochores = ON requires attached sister chromatics, the absence of $APC/C^{Cdh1}$ activity. It is turned on my CyclinB/Cdk1 and remains on until the spindle is complete (or it is destroyed by $APC/C^{Cdh1}$). | | |
| **Mad2** | ← | **U_Kinetochores** | The Mad2 SAC protein is active and potent in the presence of even a single unattached kinetochore [165]. |
| **Mad2** | ⊢ | **A_Kinetochores** | Mad2 is inhibited by SAC passage, marked by the completion of the spindle and proper attachment of all kinetochore [165]. |

| Target Node | Link | Input | Description & References |
|---|---|---|---|
| **Mad2** | **U_Kinetochores** and not **A_Kinetochores** | | |
| | Our model represents the SAC via the Mad2 kinetochore-binding protein. Mad2 is active as long as the cell has at least one unattached kinetochore and it is responsible for keeping Cdc20 sequestered from APC/C. By keeping APC at bay until the spindle is complete, Mad2 is required for the proper timing of anaphase [165]. | | |
| **A_Kinetochores** | ← | **4N_DNA** | Completion of the mitotic spindle requires replicated and attached sister chromatids (4N_DNA). |
| **A_Kinetochores** | ⊢ | **Cdh1** | APC/C$^{Cdh1}$ destroys the spindle by triggering anaphase via the degradation of APC/C targets, including Securin [191]. |
| **A_Kinetochores** | ⊢ | **pAPC** | During normal mitosis, the completed spindle is pulled apart in response to APC/C$^{Cdc20}$-mediated degradation of Securin, which normally blocks separate from severing the cohesin rings keeping sister chromatids attached [190]. |
| **A_Kinetochores** | ⊢ | **Cdc20** | During normal mitosis, the completed spindle is pulled apart in response to APC/C$^{Cdc20}$-mediated degradation of Securin, which normally blocks separate from severing the cohesin rings keeping sister chromatids attached [190]. |
| **A_Kinetochores** | ← | **A_Kinetochores** | Once assembled, separation of the mitotic spindle requires APC/C activity to promote the destruction of sister chromatid cohesion [190]. |
| **A_Kinetochores** | ← | **U_Kinetochores** | The mitotic spindle is assembled gradually, as the number of unattached kinetochores gradually decreased by the formation of microtubule attachments. |
| **A_Kinetochores** | ← | **Plk1** | Plk1 activity at unattached kinetochores is required for promoting their attachment [193]. In its absence, kinetochores remain unattached and cells eventually undergo mitotic catastrophe and apoptosis [194]. |
| **A_Kinetochores** | ← | **CyclinB** | Ongoing Cyclin B / Cdk1 at unattached kinetochores is necessary to keep Plk1 active and allow the completion of mitosis [195]. |
| **A_Kinetochores** | ← | **Cdk1** | Ongoing Cyclin B / Cdk1 at unattached kinetochores is necessary to keep Plk1 active and allow the completion of mitosis [195]. |
| **A_Kinetochores** | **4N_DNA** and not **Cdh1** and not (**pAPC** and **Cdc20**) and [**A_Kinetochores** or (**U_Kinetochores** and **Plk1** and **CyclinB** and **Cdk1**) ] | | |
| | The completed spindle, represented by the A_Kinetochores node, requires replicated and attached sister chromatids (4N_DNA) and the absence of APC/C activity. It turns on when the process of spindle assembly (U_Kinetochores) is completed by active Plk1 localized to unattached kinetochores in the presence of ongoing Cyclin B / Cdk1 activity and it remains on until anaphase (APC/C activation). | | |
| **Plk1_H** | ← | **Plk1** | Active mitotic Plk1 is a prerequisite for the accumulation of the larger active Plk1 pool denoted by Plk1_H. |
| **Plk1_H** | ← | **FoxM1** | Plk1 is a direct transcriptional target of FoxM1; loss of FoxM1 severely reduces Plk1 protein levels [101,112]. |

| Target Node | Link | Input | Description & References |
|---|---|---|---|
| **Plk1_H** | ← | **FoxO3** | Plk1 is a direct transcriptional target of FoxO3, but Plk1 appears to be sufficiently induced in the absence of FoxO preteens to aid its G2/M and mitotic functions. In contrast, accumulation if a large enough Plk1 pool to briefly outlast APC/$C^{Cdh1}$ activation (modeled by the Plk1_H node), requires FoxO activity in G2 [136]. |
| **Plk1_H** | ← | **FoxO1** | In addition to FoxO3, FoxO1 also binds the Plk1 promoter, potentially aiding its accumulation during G2 [196]. |
| **Plk1_H** | ← | **Plk1_H** | Once accumulated, we assume that the Plk1_H pool of active Plk1 remains stable in the absence of FoxO-mediated transcription. This is supported by negative feedback regulation of FoxO proteins by Plk1 [14], indicating that ongoing high FoxO activity is likely not required for the maintenance of Plk1_H. |
| **Plk1_H** | **Plk1** and **FoxM1** and (**Plk1_H** or **FoxO3** or **FoxO1**) | | |
| | The ON state of Plk1_H encodes the short-lived memory of a sufficiently large active Plk1 pool to temporarily survive Plk1 destruction by APC/CCdh1 [141], recruit Ect2 to the central spindle, and thus aid the completion of cytokinesis [188]. Thus, Plk1_H requires ongoing Plk1 activation and transcription by FoxM1, and either induction by FoxO3 or FoxO1, or prior accumulation. | | |
| **Ect2** | ← | **4N_DNA** | Formation of a spindle midzone, where Ect2 accumulates in preparation of cytokinesis requires recently separated sister chromatids (4N DNA content). |
| **Ect2** | ← | **Plk1_H** | Plk1 activity in telophase (Plk1_H) is required for the recruitment of Ect2 to the central spindle [141]. |
| **Ect2** | ← | **Cdh1** | APC/$C^{Cdh1}$ -mediated destruction of Aurora kinase is required for the assembly of a robust spindle midzone at anaphase and for the normal timing of cytokinesis [197]. |
| **Ect2** | ⊢ | **U_Kinetochores** | Formation of a spindle midzone requires the separation of sister chromatids; thus it cannot occur before anaphase. |
| **Ect2** | ⊢ | **A_Kinetochores** | Formation of a spindle midzone requires the separation of sister chromatids; thus it cannot occur before anaphase. |
| **Ect2** | **4N_DNA** and **Plk1_H** and **Cdh1** and not **U_Kinetochores** and not **A_Kinetochores** | | |
| | Ect2 activaiton at the spindle midzone represents the step of cytokinesis in our model. Thus, Ect2 requires 4N_DNA, high Plk1 activity, as well as Cdh1 for the assembly of a normal spindle midzone. Finally, Ect2 cannot be recruited to the mid zone before anaphase is completed. | | |

**S1.F.** Boolean regulatory logic of Apoptotic Switch nodes.

| Target Node | Link | Input | Description & References |
|---|---|---|---|
| **Trail** | ← | **Trail** | The Trail node represents environmental availability of the Trail protein outside the cell; this input node remains on/off if set ON/OFF in the absence of in silico perturbation. |
| **DR4_5** | ← | **Trail** | The DR4 and DR5 death receptors, represented by the DR4_5 node, are activated by extracellular Trail [198]. |
| **Casp8** | ← | **DR4_5** | Trail-bound (active) DR4 and DR5 receptors trigger the assembly of the pro-apoptotic death-inducing signaling complex (DISC), which binds a cluster of pro-Caspase 8 proteins and initiates their cleavage into active Caspase 8 [199]. |
| **Casp8** | ← | **Casp3** | Caspase 3 indirectly activates Caspase 8 by cleaving Caspase 6 [200], which, in turn, cleaves Caspase 8 [201]. |
| **Casp8** | **DR4_5** or **Casp3** | | |
| | Pro-Caspase 8 may be cleaved independently by DISC (DR4_5) or Caspase 3. | | |
| **Casp2** | ← | **Casp3** | Caspase 2 is a target of Caspase 3, as its inhibition severely limits Caspase 2 cleavage during apoptosis [202,203]. |
| **Casp2** | ← | **U_Kinetochores** | Although the precise molecular mechanism by which Caspase 2 is activated during prolonged or stalled mitosis is unclear, its activation platform, the PIDDosome, has been localized to unattached kinetochores [204]. Even though a checkpoint protein keeps the PIDDosome unresponsive to DNA damage signals, the loss of protective Cyclin B / Cdk1 phosphorylation only leads to Caspase 2 activation in the presence of a partially assembled mitotic spindle, and requires active SAC. |
| **Casp2** | ← | **Mad2** | A functional spindle assembly checkpoint is required for mitotic cell death upon prolonged mitotic arrest [205] or spindle damage [206]. |
| **Casp2** | ⊢ | **Cdk1** | Cyclin B1/Cdk1 phosphorylate caspase-2 at Ser 340, preventing its activation [207]. |
| **Casp2** | ⊢ | **CyclinB** | Cyclin B1/Cdk1 phosphorylate caspase-2 at Ser 340, preventing its activation [207]. |
| **Casp2** | **Casp3** or [**U_Kinetochores** and **Mad2** and not (**CyclinB** and **Cdk1**) ] | | |
| | Pro-caspase 2 is cleaved and activated by Caspase 3, or by failed cytokinesis marked by the presence of unattached kinetochores, an active SAC, and the absence of active Cyclin B / Cdk1 complexes to phosphorylate and inhibit Caspase 2. | | |
| **MCL-1** | ⊢ | **Casp3** | Caspase 3 cleaves and deactivated MCL-1 [208]. |
| **MCL-1** | ⊢ | **Casp2** | Caspase 2 activation destabilizes the MCL-1 protein [209]. |
| **MCL-1** | ⊢ | **U_Kinetochores** | During prolonged mitotic arrest (U_kinetochores), MCL-1 levels drop steadily due to phosphorylation by JNK, p38 and/or CKII and its subsequent degradation by the E3 ubiquitin ligase SCF(FBW7) [210]. |

| Target Node | Link | Input | Description & References |
|---|---|---|---|
| **MCL-1** | ⊢ | **Cdk1** | Phosphorylation by CyclinB / Cdk1 in cells arrested in mitosis initiates MCL-1 degradation [211]. |
| **MCL-1** | ⊢ | **CyclinB** | In cells arrested in mitosis, phosphorylation by CyclinB / Cdk1 on T92 initiates MCL-1 degradation [211]. |
| **MCL-1** | ⊢ | **E2F1** | E2F1 is a direct transcriptional repressor of MCL-1 [212]. |
| **MCL-1** | ← | **ERK** | ERK phosphorylates Mcl-1, promoting its interaction with Pin1, which stabilizes it [213,214]. |
| **MCL-1** | ⊢ | **GSK3** | MCL-1 is phosphorylated by GSK-3, leading to ubiquitinylation and degradation of MCL-1 [215]. |
| **MCL-1** | ← | **AKT_B** | In order to account for the loss of MCL-1 in the complete absence of growth factors vs. its presence in low growth factor environments, we required basal AKT to modulate the strength of GSK3 inhibition [215]. |
| **MCL-1** | not **Casp3** and not **Casp2** and {not **GSK3** or [**AKT_B** and (**ERK** or not **E2F1**) ] } and [not (**Cdk1** and **CyclinB** and **U_Kinetochores**) ] | | |
| | Caspase 3 or 2-mediated destruction of MCL-1 must be absent for MCL-1 to be ON. Avoiding degradation via GSK3 requires the GSK3-weakenjng presence of basal AKT activity (AKT_B) [215] and either ERK-mediated stabilization, or the absence of its repressor E2F1. Finally, during mitotic arrest (U_Kinetochores), MCL-1 is deactivated by CyclinB / Cdk1 phosphorylation, which shields it from the PPA2-mediated dephosphorylation of its degradation-targeting sites [210]. | | |
| **BCLXL** | ⊢ | **Casp3** | BCL-xL is cleaved and deactivated by Caspase 3 [216]. |
| **BCLXL** | ⊢ | **BAD** | Bad can bind BCL-xL and displace it from BAX, thus deactivating it [217]. |
| **BCLXL** | ← | **BCL2** | BCL2 competes with BCL-xL for BAD binding. Although BCL-xL is a stronger binding partner, we assume that BAD alone cannot fully block BCL-xL in the presence of BCL2 [217]. |
| **BCLXL** | ← | **MCL-1** | MCL-1 competes with BCL-xL for BAK binding; the presence of MCL-1 can keep part of the BCL-xL pool active [218]. |
| **BCLXL** | ⊢ | **U_Kinetochores** | Prolonged mitosis is required for the accumulation of BCL-xL phosphorylation, weakening its interaction with Bax [219]. |
| **BCLXL** | ← | **Plk1** | In addition to other effects of prolonged mitotic arrest on BCL-2 proteins, Plk1 inhibition synergistically enhances the inhibitory phosphorylation of BCL-2 and BCL-xL, as well as downregulation of MCL-1 [220]. |
| **BCLXL** | ⊢ | **Cdk1** | During normal mitosis, Cyclin B / Cdk1 only transiently phosphorylates part of the BCLXL pool. Prolonged mitosis, however, results in high levels of BCL-xL (and Bcl-2) phosphorylation, priming the system for Caspase 2-mediated apoptosis [221,222]. |
| **BCLXL** | ⊢ | **CyclinB** | Cyclin B / Cdk1 phosphorylates BCL-xL (and Bcl-2) during mitosis [221,222]. |

| Target Node | Link | Input | Description & References |
|---|---|---|---|
| **BCLXL** | not **Casp3** and (**BCL2** or not **BAD**) and { not **U_Kinetochores** or [ **Plk1** and ( not {**CyclinB** and **Cdk1**} or {**BCL2** and **MCL-1**} ) ] or [ **BCL2** and **MCL-1** and not (**CyclinB** and **Cdk1**) ] } | | |
| | While the precise combinatorial logic governing BCLXL activity is not clear from literature. BCLXL activity requires the absence of Caspase 3. In addition, BAD can block BCLXL when BCL2 is also OFF. Finally, mitotic BCLXL can be inhibited by Cdk1 activity if either BCL2, MCL-1, or Plk1 are OFF. In the absence of Plk1, loss of either BCL2 or MCL-1 can result in BCLXL inhibition (even without Cdk1 phosphorylation), as we assume its targets are no longer competitively bound by its family members. | | |
| **BCL2** | ⊢ | **Casp3** | BCL2 is cleaved and deactivated by Caspase 3 [223]. |
| **BCL2** | ⊢ | **BAD** | BCL2 competes with BCL-xL for BAD binding. BAD displaces BCL2 from its  Although BCL-xL is a stronger binding partner, we assume that BAD alone cannot fully block BCL-xL in the presence of BCL2 [217]. |
| **BCL2** | ⊢ | **BIM** | BIM binds BCL2 and they mutually inhibit each other's ability to activate further targets [224]. |
| **BCL2** | ⊢ | **BIK** | BIK also binds BCL2 and they mutually inhibit each other's activity [225]. |
| **BCL2** | ← | **BCLXL** | BCL2 competes with BCL-xL for binding most of their apoptotic partners, including BIK, BIM, BID, BAX and BAK. |
| **BCL2** | ← | **MCL-1** | MCL-1 competes with BCL2 for binding most of their apoptotic partners, including BIK, BIM, BID, BAX and BAK. |
| **BCL2** | ⊢ | **U_Kinetochores** | Prolonged mitosis is required for the accumulation of BCL-2 phosphorylation [221,222,226]. |
| **BCL2** | ← | **Plk1** | In addition to other effects of prolonged mitotic arrest on BCL-2 proteins, Plk1 inhibition synergistically enhances the inhibitory phosphorylation of BCL-2 and BCL-xL, as well as downregulation of MCL-1 [220]. |
| **BCL2** | ⊢ | **Cdk1** | Prolonged mitosis results in high levels of BCL-xL and Bcl-2 phosphorylation, priming the system for Caspase 2-mediated apoptosis [221,222,226]. |
| **BCL2** | ⊢ | **CyclinB** | Cyclin B / Cdk1 phosphorylates Bcl-2 (and BCL-xL) during mitosis [221,222]. |
| **BCL2** | not (**Casp3** or **BAD** or **BIM** or **BIK**) and  {not **U_Kinetochores** or (**MCL-1** and **BCLXL**) or [**Plk1** and (**BCLXL** or **MCL-1** or not {**Cdk1** and **CyclinB**} ) ] } | | |
| | While the precise combinatorial logic governing BCL2 activity is not clear from literature, we modeled BCL2 as ON in the absence of Caspase 3, BAD, BIM or BIK. This choice makes BCL2 the most sensitive of the three family members to activation of its three inhibitors. In addition, mitotic BCL2 is blocked by Cdk1 if both BCL-xL and MCL-1 are OFF. In the absence of Plk1, loss of either BCL2 or MCL-1 can result in BCL-2 inhibition (even without Cdk1 phosphorylation), as we assume its targets are no longer competitively bound by its family members. | | |
| **BAD** | ← | **Casp3** | Caspase 3 cleaves BAD, generating a more potently apoptotic fragment [227]. |

| Target Node | Link | Input | Description & References |
|---|---|---|---|
| **BAD** | ← | **Casp8** | Caspase 8 is also able to cleave BAD, generating a more potently apoptotic fragment [227]. In addition, TRAIL-mediated apoptosis results in BAD cleavage by a Caspase upstream of MOMP, creating a potent apoptotic inducer before full Caspase 3 activation [228]. |
| **BAD** | ⊢ | **AKT_B** | Akt phosphorylates BAD at Ser-136, inducing its sequestration away from the mitochondrial membrane where its BCL-2 family targets are located (e.g., BCL-2, BCL-xL) [229] |
| **BAD** | ⊢ | **AKT_H** | Akt phosphorylates BAD at Ser-136, inducing its sequestration away from the mitochondrial membrane where its BCL-2 family targets are located (e.g., BCL-2, BCL-xL) [229] |
| **BAD** | ⊢ | **ERK** | ERK phosphorylates BAD at Ser-112, inducing its sequestration away from the mitochondrial membrane where its BCL-2 family targets are located (e.g., BCL-2, BCL-xL)[230]. |
| **BAD** | ⊢ | **S6K** | S6K1 phosphorylates BAD at Ser-155, directly blocking its binding to BCL-xL [231]. |
| **BAD** | **Casp3** or not (**AKT_H** or **AKT_B** or **ERK** or **S6K**) or {**Casp8** and [ not (**AKT_B** and **ERK** and **S6K**) and not (**AKT_H** and {**AKT_B** or **ERK**} ) ] } | | |
| | BAD in our model is ON when cleaved by Caspase 3, or in the complete absence of survival signals (AKT, ERK or S6K). Alternatively, BAD can be cleaved and activated by Caspase 8 in the absence of strong survival signaling. We modeled this inhibitory survival signal as either the combined activity of ERK, S6K and (at least) basal AKT, or high AKT in the joint presence of ERK and basal AKT (indicating that AKT_H will not drop by the next time-step). | | |
| **BIK** | ⊢ | **BCL2** | BCL2 binds BIK; they mutually inhibit each other [225]. |
| **BIK** | ⊢ | **BCLXL** | BCLXL also binds BIK; they mutually inhibit each other [232]. |
| **BIK** | ⊢ | **MCL-1** | MCL-1 also binds BIK; they mutually inhibit each other [233]. |
| **BIK** | not (**MCL-1** or **BCLXL** or **BCL2**) | | |
| | BIK is free to activate its target, BAX, only when it is not sequestered by any of the three BCL-2 family proteins in our model [233]. | | |
| **BIM** | ← | **FoxO3** | FoxO3 is a transcriptional activator of BIM [234]. |
| **BIM** | ← | **GSK3** | GSK3 kinase is likely required for the AP1-dependent expression of BIM [235]. |
| **BIM** | ⊢ | **ERK** | The MEK/ERK pathway represses BIM protein levels, likely via transcriptional repression [236]. |
| **BIM** | ⊢ | **BCL2** | BLC2 binds BIM and inhibits its apoptotic activity [224]. |
| **BIM** | ⊢ | **BCLXL** | BLC-XL binds BIM and inhibits its apoptotic activity [224]. |
| **BIM** | ⊢ | **MCL-1** | MCL-1 binds BIM and inhibits its apoptotic activity [237]. |
| **BIM** | **FoxO3** and **GSK3** and not (**ERK** or **MCL-1** or **BCLXL** or **BCL2**) | | |

| Target Node | Link | Input | Description & References |
|---|---|---|---|
| | BIM's pro-apoptotic activity requires expression driven by FoxO3 and aided by GSK3 and the absence of all three inhibitory BCL2 family proteins. | | |
| **BID** | ← | **Casp8** | In response to TRAIL (or FAS ligand), the initiator caspase 8 cleaves BID to its active truncated form [238-240]. |
| **BID** | ← | **Casp2** | Caspase 2 cleaves BID to its active truncated form [241] |
| **BID** | ⊢ | **BCL2** | All three anti-apoptotic BCL2 proteins (BCL2, BCL-xL and MCL-1) sequesters BID into stable complexes, preventing them from activating BAX or BAK [242]. |
| **BID** | ⊢ | **BCLXL** | All three anti-apoptotic BCL2 proteins (BCL2, BCL-xL and MCL-1) sequesters BID into stable complexes, preventing them from activating BAX or BAK [242]. |
| **BID** | ⊢ | **MCL-1** | All three anti-apoptotic BCL2 proteins (BCL2, BCL-xL and MCL-1) sequesters BID into stable complexes, preventing them from activating BAX or BAK [242]. |
| **BID** | **Casp8** or { **Casp2** and not (**BCL2** or **BCLXL** or **MCL-1**) } | | |
| | BID is truncated in response to Caspase 8 activation. In addition, Caspase 2 can also promote BID activation once all three pro-apoptotic BCL2 family proteins are blocked. | | |
| **BAK** | ← | **BID** | Activated (truncated) BID binds to mitochondrial BAK, resulting in its activation and oligomerization in the mitochondrial membrane, followed by cytochrome c release [243]. |
| **BAK** | ← | **BIM** | BAK is preferentially activated by BID compared to BIM, but BIM can also promote BAK oligomerization [244] |
| **BAK** | ← | **BIK** | BIK can aid the activation of both BAK and BAX by triggering BAK oligomerization on the ER membrane and promoting a Ca2+ efflux required for the fragmentation of hyper fused mitochondrial tubules, aiding BAK and BAX activation [245]. |
| **BAK** | ⊢ | **BCLXL** | BCLXL binds BAK and prevent its oligomerization in the mitochondrial membrane [246-248]. |
| **BAK** | ⊢ | **MCL-1** | MCL-1 binds BAK and prevent its oligomerization in the mitochondrial membrane [247,248]. |
| **BAK** | ⊢ | **BCL2** | BCL2 can also bind BAK to prevent its oligomerization, but it does so less potently than the other two BCL-2 family members [247-249]. |
| **BAK** | {**BID** and [**BIM** or **BIK** or not (**BCL2** and **BCLXL** and **MCL-1**) ] } or { (**BIM** or **BIK**) and not (**BCLXL** or **MCL-1**) } | | |
| | Given that BAK is preferentially activated by BID compared to BIM [244] and that it is less responsive to sequestration by BCL2 than the other two anti-apoptotic BCL2 family proteins [247,248], BAK in our model turns on when stimulated by BID if one or more BCL2 family proteins are absent, or if BIM or BIK are also present. In contrast, BIM or BIK only activate BAK if BCLXL and MCL-1 are absent (BCL-2 alone cannot block them). | | |
| **BAX** | ← | **BIM** | Activated BIM binds to mitochondrial BAX, resulting in its allosteric activation and oligomerization in the mitochondrial membrane, leading to cytochrome c release [244]. |

| Target Node | Link | Input | Description & References |
|---|---|---|---|
| **BAX** | ← | **BID** | BAX is preferentially activated by BIM compared to BID, but BID can also promote BAK oligomerization [244]. |
| **BAX** | ← | **BIK** | BIK can aid the activation of both BAK and BAX by triggering BAK oligomerization on the ER membrane and promoting a Ca2+ efflux required for the fragmentation of hyper fused mitochondrial tubules, aiding BAK and BAX activation [245]. |
| **BAX** | ⊢ | **BCL2** | BCL2 binds BAX and prevent its oligomerization in the mitochondrial membrane [247,248]. |
| **BAX** | ⊢ | **BCLXL** | BCL-xL binds BAK and prevent its oligomerization in the mitochondrial membrane [247,248]. |
| **BAX** | ⊢ | **MCL-1** | MCL-1 can also bind BAK to prevent its oligomerization, but it does so less potently than the other two BCL-2 family members [247,248,250]. |
| **BAX** | {**BIM** and [ (**BID** or **BIK**) or not (**BCL2** and **BCLXL** and **MCL-1**) ] } or { (**BID** or **BIK**) and not (**BCL2** or **BCLXL**) } | | |
| | In contrast to BAK, BAX is preferentially activated by BIM compared to BID [244] and it is less responsive to sequestration by MCL-1 than the other two anti-apoptotic BCL2 family proteins [247,248]. BAX in our model turns on when stimulated by BIM if one or more BCL2 family proteins are absent, or if BID or BIK are also present. In contrast, BID or BIK only activate BAK if BCL2 and BCLXL are absent (MCL-1 alone cannot block them). | | |
| **Cyto_C** | ← | **BAX** | BAX oligomerization at the mitochondrial membrane triggers MOMP, which results in the release of cytochrome C from mitochondria [251,252]. |
| **Cyto_C** | ← | **BAK** | BAK oligomerization at the mitochondrial membrane triggers MOMP, which results in the release of cytochrome C from mitochondria [253]. |
| **Cyto_C** | **BAX** or **BAK** | | |
| | Cytochrome C release from mitochondria requires the oligomerization of either BAK or BAX [253]. | | |
| **SMAC** | ← | **BAX** | BAX oligomerization at the mitochondrial membrane triggers MOMP, which results in the release of cytochrome C from mitochondria [253,254]. |
| **SMAC** | ← | **BAK** | BAK oligomerization at the mitochondrial membrane triggers MOMP, which results in the release of cytochrome C from mitochondria [253,254]. |
| **SMAC** | **BAX** or **BAK** | | |
| | SMAC/Diablo release from mitochondria requires the oligomerization of either BAK or BAX [253,254]. | | |
| **IAPs** | ← | **AKT_H** | cIAP-2 and XIAP are both transcriptionally up-regulated in response to strong PI3K/AKT1 activation [255] |
| **IAPs** | ⊢ | **SMAC** | SMAC / Diablo binds tightly to IAP proteins and blocks their ability to inhibit Caspase 3 [256]. |

| Target Node | Link | Input | Description & References |
|---|---|---|---|
| **IAPs** | not **SMAC** or **AKT_H** | | |
| | Inhibitor of Apoptosis Proteins (IAPs) are active in the absence of SMAC/Diablo inhibition, or following AKT_H mediated up regulation (this protection from SMAC requires peak or oncogenic AKT activity). | | |
| **Casp9** | ← | **Casp3** | Procaspase 9 is a direct cleavage target of Caspase 3 [203]. |
| **Casp9** | ⊢ | **IAPs** | XIAP, cIAP1 and cIAP2 inhibitor the the cytochrome c-induced activation of procaspase-9 [257]. |
| **Casp9** | ← | **Cyto_C** | Cytochrome c binds to APAF-1 proteins, promoting their assembly into the apoptosome, a platform for procaspase 9 binding and cleavage into its active form [258]. |
| **Casp9** | **Casp3** or (not **IAPs** and **Cyto_C**) | | |
| | Procaspase 9 is cleaved into active Caspase 9 by Caspase 3, or by the apoptosome (which relies on Cytochrome C for its assembly) in the absence of IAP proteins. | | |
| **Casp3** | ⊢ | **IAPs** | IAPs bind tightly to the active site of Caspase 3, keeping its activity in check [257,259]. |
| **Casp3** | ← | **Casp9** | Active Caspase 9 cleaves Caspase 3 [260]. |
| **Casp3** | ← | **Casp8** | Caspase 8 can cleave Caspase 3 [261], but full Caspase 3 activation also requires MOMP (potentially due to a need for IAP inhibition) [262]. |
| **Casp3** | ← | **Casp3** | Once activated, Caspase 3 helps sustain its own activation by cleaving procaspase 8 and 6. Caspase 6, in turn, generates additional active caspase 8 and 9. Together they all sustains a continuing active pool of Caspase 3. |
| **Casp3** | (**Casp9** and **Casp8**) or [ **Casp3** and (**Casp9** or **Casp8**) ] or [ not **IAPs** and (**Casp9** or **Casp8** or **Casp3**) ] | | |
| | Activation of Caspase 3 requires proteolytic cleavage of procaspase-3 by initiator caspases such as Caspase 9 or Caspase 8. In our model, cooperation of two of the three caspases (Casp9, Casp8, Casp3) is required in the presence of IAPS, which inhibit the proteolytic activity of Caspase 3 by bind tightly to its active site. In the absence of IAPS, either of the three caspases can cleave and activate Caspase 3. | | |
| **CAD** | ← | **Casp3** | Caspase 3 relives CAD inhibition by cleaving its inhibitor ICAD [263]. |
| **CAD** | ← | **Casp9** | In addition of Caspase 3, CAD inhibition can also be relieved by ICAD cleavage by Caspase 7, which is a direct target of Caspase 9 [263]. |
| **CAD** | **Casp3** and **Casp9** | | |
| | Caspase-activated DNase (CAD) is activated when its inhibition is released via the cleavage of ICAD (inhibitor of caspase-activated DNase). While Capsase 3 and 7 (a direct target of Caspase 9) can inhibit ICAD [263], in our model they are both required, as CAD = ON is represents terminal, irreversible apoptotic commitment, which is fully locked in when both Caspase 3 and 9 are on. | | |

## References


1. Lemmon MA, Schlessinger J. Cell signaling by receptor tyrosine kinases. Cell. 2010;141: 1117–1134. doi:10.1016/j.cell.2010.06.011
2. Uzman A. Molecular Cell Biology (4th edition) Harvey Lodish, Arnold Berk, S. Lawrence Zipursky, Paul Matsudaira, David Baltimore and James Darnell; Freeman & Co., New York, NY, 2000, 1084 pp., ISBN 0-7167-3136-3. Biochemistry and Molecular Biology Education. 2001;29: 126–128. doi:10.1016/S1470-8175(01)00023-6
3. Widmann C, Gibson S, Johnson GL. Caspase-dependent cleavage of signaling proteins during apoptosis. A turn-off mechanism for anti-apoptotic signals. J Biol Chem. 1998;273: 7141–7147.
4. Chang F, Steelman LS, Lee JT, Shelton JG, Navolanic PM, Blalock WL, et al. Signal transduction mediated by the Ras/Raf/MEK/ERK pathway from cytokine receptors to transcription factors: potential targeting for therapeutic intervention. Leukemia. 2003;17: 1263–1293. doi:10.1038/sj.leu.2402945
5. Liu P, Gan W, Chin YR, Ogura K, Guo J, Zhang J, et al. PtdIns(3,4,5)P3-Dependent Activation of the mTORC2 Kinase Complex. Cancer Discov. 2015;5: 1194–1209. doi:10.1158/2159-8290.CD-15-0460
6. Dibble CC, Asara JM, Manning BD. Characterization of Rictor phosphorylation sites reveals direct regulation of mTOR complex 2 by S6K1. Mol Cell Biol. 2009;29: 5657–5670. doi:10.1128/MCB.00735-09
7. Rodriguez-Viciana P, Warne PH, Dhand R, Vanhaesebroeck B, Gout I, Fry MJ, et al. Phosphatidylinositol-3-OH kinase as a direct target of Ras. Nature. 1994;370: 527–532. doi:10.1038/370527a0
8. Gupta S, Ramjaun AR, Haiko P, Wang Y, Warne PH, Nicke B, et al. Binding of ras to phosphoinositide 3-kinase p110alpha is required for ras-driven tumorigenesis in mice. Cell. 2007;129: 957–968. doi:10.1016/j.cell.2007.03.051
9. Manning BD, Toker A. AKT/PKB Signaling: Navigating the Network. Cell. 2017;169: 381–405. doi:10.1016/j.cell.2017.04.001
10. Hui RC-Y, Gomes AR, Constantinidou D, Costa JR, Karadedou CT, Fernández de Mattos S, et al. The forkhead transcription factor FOXO3a increases phosphoinositide-3 kinase/Akt activity in drug-resistant leukemic cells through induction of PIK3CA expression. Mol Cell Biol. 2008;28: 5886–5898. doi:10.1128/MCB.01265-07
11. Wang Z, Dang T, Liu T, Chen S, Li L, Huang S, et al. NEDD4L Protein Catalyzes Ubiquitination of PIK3CA Protein and Regulates PI3K-AKT Signaling. Journal of Biological Chemistry. 2016;291: 17467–17477. doi:10.1074/jbc.M116.726083
12. Yuan TL, Wulf G, Burga L, Cantley LC. Cell-to-Cell Variability in PI3K Protein Level Regulates PI3K-AKT Pathway Activity in Cell Populations. Curr Biol. 2011;21: 173–183. doi:10.1016/j.cub.2010.12.047
13. Yang J-Y, Zong CS, Xia W, Yamaguchi H, Ding Q, Xie X, et al. ERK promotes tumorigenesis by inhibiting FOXO3a via MDM2-mediated degradation. Nat Cell Biol. 2008;10: 138–148. doi:10.1038/ncb1676
14. Bucur O, Stancu AL, Muraru MS, Melet A, Petrescu SM, Khosravi-Far R. PLK1 is a binding partner and a negative regulator of FOXO3 tumor suppressor. Discoveries (Craiova). 2014;2: e16. doi:10.15190/d.2014.8
15. Cockcroft S, Thomas GM. Inositol-lipid-specific phospholipase C isoenzymes and their differential regulation by receptors. Biochem J. 1992;288 ( Pt 1): 1–14.
16. Kim HK, Kim JW, Zilberstein A, Margolis B, Kim JG, Schlessinger J, et al. PDGF stimulation of inositol phospholipid hydrolysis requires PLC-gamma 1 phosphorylation on tyrosine residues 783 and 1254. Cell. 1991;65: 435–441.

17. Zhang W, Trible RP, Zhu M, Liu SK, McGlade CJ, Samelson LE. Association of Grb2, Gads, and phospholipase C-gamma 1 with phosphorylated LAT tyrosine residues. Effect of LAT tyrosine mutations on T cell angigen receptor-mediated signaling. J Biol Chem. 2000;275: 23355–23361. doi:10.1074/jbc.M000404200
18. Falasca M, Logan SK, Lehto VP, Baccante G, Lemmon MA, Schlessinger J. Activation of phospholipase C gamma by PI 3-kinase-induced PH domain-mediated membrane targeting. EMBO J. 1998;17: 414–422. doi:10.1093/emboj/17.2.414
19. Rameh LE, Rhee SG, Spokes K, Kazlauskas A, Cantley LC, Cantley LG. Phosphoinositide 3-kinase regulates phospholipase Cgamma-mediated calcium signaling. J Biol Chem. 1998;273: 23750–23757.
20. Gresset A, Sondek J, Harden TK. The phospholipase C isozymes and their regulation. Subcell Biochem. 2012;58: 61–94. doi:10.1007/978-94-007-3012-0_3
21. Michell RH, Kirk CJ, Jones LM, Downes CP, Creba JA. The stimulation of inositol lipid metabolism that accompanies calcium mobilization in stimulated cells: defined characteristics and unanswered questions. Philos Trans R Soc Lond, B, Biol Sci. 1981;296: 123–138.
22. Escobedo A, Gomes T, Aragón E, Martín-Malpartida P, Ruiz L, Macias MJ. Structural basis of the activation and degradation mechanisms of the E3 ubiquitin ligase Nedd4L. Structure. 2014;22: 1446–1457. doi:10.1016/j.str.2014.08.016
23. Yuan C, Wang L, Zhou L, Fu Z. The function of FOXO1 in the late phases of the cell cycle is suppressed by PLK1-mediated phosphorylation. Cell Cycle. 2014;13: 807–819. doi:10.4161/cc.27727
24. Seoane J, Le H-V, Shen L, Anderson SA, Massagué J. Integration of Smad and forkhead pathways in the control of neuroepithelial and glioblastoma cell proliferation. Cell. 2004;117: 211–223.
25. Staller P, Peukert K, Kiermaier A, Seoane J, Lukas J, Karsunky H, et al. Repression of p15INK4b expression by Myc through association with Miz-1. Nature Cell Biology. 2001;3: 392–399. doi:10.1038/35070076
26. Seoane J, Le H-V, Massagué J. Myc suppression of the p21(Cip1) Cdk inhibitor influences the outcome of the p53 response to DNA damage. Nature. 2002;419: 729–734. doi:10.1038/nature01119
27. Inoki K, Li Y, Zhu T, Wu J, Guan K-L. TSC2 is phosphorylated and inhibited by Akt and suppresses mTOR signalling. Nature Cell Biology. 2002;4: 648–657. doi:10.1038/ncb839
28. Saxton RA, Sabatini DM. mTOR Signaling in Growth, Metabolism, and Disease. Cell. 2017;169: 361–371. doi:10.1016/j.cell.2017.03.035
29. Phosphorylation and Functional Inactivation of TSC2 by Erk: Implications for Tuberous Sclerosisand Cancer Pathogenesis. Cell. 2005;121: 179–193. doi:10.1016/j.cell.2005.02.031
30. Roux PP, Ballif BA, Anjum R, Gygi SP, Blenis J. Tumor-promoting phorbol esters and activated Ras inactivate the tuberous sclerosis tumor suppressor complex via p90 ribosomal S6 kinase. Proc Natl Acad Sci U S A. 2004;101: 13489–13494. doi:10.1073/pnas.0405659101
31. Haar EV, Lee S-I, Bandhakavi S, Griffin TJ, Kim D-H. Insulin signalling to mTOR mediated by the Akt/PKB substrate PRAS40. Nature Cell Biology. 2007;9: 316–323. doi:10.1038/ncb1547
32. Fonseca BD, Smith EM, Lee VH-Y, MacKintosh C, Proud CG. PRAS40 is a target for mammalian target of rapamycin complex 1 and is required for signaling downstream of this complex. J Biol Chem. 2007;282: 24514–24524. doi:10.1074/jbc.M704406200
33. Wiza C, Nascimento EBM, Ouwens DM. Role of PRAS40 in Akt and mTOR signaling in health and disease. Am J Physiol-Endoc M. 2012;302: E1453–60. doi:10.1152/ajpendo.00660.2011
34. Inoki K, Li Y, Xu T, Guan K-L. Rheb GTPase is a direct target of TSC2 GAP activity and regulates mTOR signaling. Genes & Development. 2003;17: 1829–1834. doi:10.1101/gad.1110003

35. Liu M, Clarke CJ, Salama MF, Choi YJ, Obeid LM, Hannun YA. Co-ordinated activation of classical and novel PKC isoforms is required for PMA-induced mTORC1 activation. PLoS ONE. 2017;12: e0184818. doi:10.1371/journal.pone.0184818
36. Demetriades C, Plescher M, Teleman AA. Lysosomal recruitment of TSC2 is a universal response to cellular stress. Nat Commun. 2016;7: 10662. doi:10.1038/ncomms10662
37. Martin R, Desponds C, Eren RO, Quadroni M, Thome M, Fasel N. Caspase-mediated cleavage of raptor participates in the inactivation of mTORC1 during cell death. Cell Death Discov. 2016;2: 16024. doi:10.1038/cddiscovery.2016.24
38. Long X, Lin Y, Ortiz-Vega S, Yonezawa K, Avruch J. Rheb Binds and Regulates the mTOR Kinase. Current Biology. 2005;15: 702–713. doi:10.1016/j.cub.2005.02.053
39. Real S, Meo-Evoli N, Espada L, Tauler A. E2F1 regulates cellular growth by mTORC1 signaling. PLoS ONE. 2011;6: e16163. doi:10.1371/journal.pone.0016163
40. Ramirez-Valle F, Badura ML, Braunstein S, Narasimhan M, Schneider RJ. Mitotic Raptor Promotes mTORC1 Activity, G2/M Cell Cycle Progression, and Internal Ribosome Entry Site-Mediated mRNA Translation. Molecular and Cellular Biology. 2010;30: 3151–3164. doi:10.1128/MCB.00322-09
41. Proteolytic cleavage of p70 ribosomal S6 kinase by caspase-3 during DNA damage-induced apoptosis. Biochemistry. 2009;48: 1474–1480. doi:10.1021/bi801840s
42. Ma XM, Blenis J. Molecular mechanisms of mTOR-mediated translational control. Nat Rev Mol Cell Bio. 2009;10: 307–318. doi:10.1038/nrm2672
43. Bushell M, McKendrick L, Jänicke RU, Clemens MJ, Morley SJ. Caspase-3 is necessary and sufficient for cleavage of protein synthesis eukaryotic initiation factor 4G during apoptosis. FEBS Letters. 1999;451: 332–336.
44. Ding Q, Xia W, Liu J-C, Yang J-Y, Lee D-F, Xia J, et al. Erk associates with and primes GSK-3beta for its inactivation resulting in upregulation of beta-catenin. Mol Cell. 2005;19: 159–170. doi:10.1016/j.molcel.2005.06.009
45. Zhang HH, Lipovsky AI, Dibble CC, Sahin M, Manning BD. S6K1 regulates GSK3 under conditions of mTOR-dependent feedback inhibition of Akt. Mol Cell. 2006;24: 185–197. doi:10.1016/j.molcel.2006.09.019
46. Gervais JL, Seth P, Zhang H. Cleavage of CDK inhibitor p21(Cip1/Waf1) by caspases is an early event during DNA damage-induced apoptosis. J Biol Chem. 1998;273: 19207–19212.
47. Lu Z, Hunter T. Ubiquitylation and proteasomal degradation of the p21(Cip1), p27(Kip1) and p57(Kip2) CDK inhibitors. Cell Cycle. 2010;9: 2342–2352. doi:10.4161/cc.9.12.11988
48. Overton KW, Spencer SL, Noderer WL, Meyer T, Wang CL. Basal p21 controls population heterogeneity in cycling and quiescent cell cycle states. Proceedings of the National Academy of Sciences. National Acad Sciences; 2014;111: 201409797–E4393. doi:10.1073/pnas.1409797111
49. Fattman CL, Delach SM, Dou QP, Johnson DE. Sequential two-step cleavage of the retinoblastoma protein by caspase-3/-7 during etoposide-induced apoptosis. Oncogene. 2001;20: 2918–2926. doi:10.1038/sj.onc.1204414
50. Kato J, Matsushime H, Hiebert SW, Ewen ME, Sherr CJ. Direct binding of cyclin D to the retinoblastoma gene product (pRb) and pRb phosphorylation by the cyclin D-dependent kinase CDK4. Genes & Development. 1993;7: 331–342.
51. Novak B, Tyson JJ. A model for restriction point control of the mammalian cell cycle. J Theor Biol. 2004;230: 563–579. doi:10.1016/j.jtbi.2004.04.039
52. Zarkowska T, Mittnacht S. Differential phosphorylation of the retinoblastoma protein by G1/S cyclin-dependent kinases. J Biol Chem. 1997;272: 12738–12746.
53. Coqueret O. New roles for p21 and p27 cell-cycle inhibitors: a function for each cell compartment? Trends Cell Biol. 2003;13: 65–70.
54. Hinds PW, Mittnacht S, Dulic V, Arnold A, Reed SI, Weinberg RA. Regulation of retinoblastoma protein functions by ectopic expression of human cyclins. Cell. 1992;70: 993–1006.

55. Eymin B, Sordet O, Droin N, Munsch B, Haugg M, Van de Craen M, et al. Caspase-induced proteolysis of the cyclin-dependent kinase inhibitor p27Kip1 mediates its anti-apoptotic activity. Oncogene. 1999;18: 4839–4847. doi:10.1038/sj.onc.1202860
56. Levkau B, Koyama H, Raines EW, Clurman BE, Herren B, Orth K, et al. Cleavage of p21Cip1/Waf1 and p27Kip1 mediates apoptosis in endothelial cells through activation of Cdk2: role of a caspase cascade. Mol Cell. 1998;1: 553–563.
57. Montagnoli A, Fiore F, Eytan E, Carrano AC, Draetta GF, Hershko A, et al. Ubiquitination of p27 is regulated by Cdk-dependent phosphorylation and trimeric complex formation. Genes & Development. 1999;13: 1181–1189.
58. Ishida N, Hara T, Kamura T, Yoshida M, Nakayama K, Nakayama KI. Phosphorylation of p27Kip1 on serine 10 is required for its binding to CRM1 and nuclear export. J Biol Chem. 2002;277: 14355–14358. doi:10.1074/jbc.C100762200
59. Müller D, Bouchard C, Rudolph B, Steiner P, Stuckmann I, Saffrich R, et al. Cdk2-dependent phosphorylation of p27 facilitates its Myc-induced release from cyclin E/cdk2 complexes. Oncogene. 1997;15: 2561–2576. doi:10.1038/sj.onc.1201440
60. Sheaff RJ, Groudine M, Gordon M, Roberts JM, Clurman BE. Cyclin E-CDK2 is a regulator of p27Kip1. Genes & Development. 1997;11: 1464–1478.
61. Medema RH, Kops GJ, Bos JL, Burgering BM. AFX-like Forkhead transcription factors mediate cell-cycle regulation by Ras and PKB through p27kip1. Nature. 2000;404: 782–787. doi:10.1038/35008115
62. Faure A, Naldi A, Chaouiya C, Thieffry D. Dynamical analysis of a generic Boolean model for the control of the mammalian cell cycle. Bioinformatics 2006;22: e124–31. doi:10.1093/bioinformatics/btl210
63. Sears R, Nuckolls F, Haura E, Taya Y, Tamai K, Nevins JR. Multiple Ras-dependent phosphorylation pathways regulate Myc protein stability. Genes & Development. 2000;14: 2501–2514. doi:10.1101/gad.836800
64. Lutterbach B, Hann SR. Hierarchical phosphorylation at N-terminal transformation-sensitive sites in c-Myc protein is regulated by mitogens and in mitosis. Molecular and Cellular Biology. 1994;14: 5510–5522.
65. Welcker M, Orian A, Jin J, Grim JE, Grim JA, Harper JW, et al. The Fbw7 tumor suppressor regulates glycogen synthase kinase 3 phosphorylation-dependent c-Myc protein degradation. Proc Natl Acad Sci U S A. 2004;101: 9085–9090. doi:10.1073/pnas.0402770101
66. Lin C-J, Malina A, Pelletier J. c-Myc and eIF4F constitute a feedforward loop that regulates cell growth: implications for anticancer therapy. Cancer Research. 2009;69: 7491–7494. doi:10.1158/0008-5472.CAN-09-0813
67. Oswald F, Lovec H, Möröy T, Lipp M. E2F-dependent regulation of human MYC: trans-activation by cyclins D1 and A overrides tumour suppressor protein functions. Oncogene. 1994;9: 2029–2036. Available: http://europepmc.org/abstract/med/8208548
68. Thalmeier K, Synovzik H, Mertz R, Winnacker EL, Lipp M. Nuclear factor E2F mediates basic transcription and trans-activation by E1a of the human MYC promoter. Genes & Development. 1989;3: 527–536.
69. Batsché E, Lipp M, Cremisi C. Transcriptional repression and activation in the same cell type of the human c-MYC promoter by the retinoblastoma gene protein: antagonisation of both effects by SV40 T antigen. Oncogene. 1994;9: 2235–2243.
70. Roussel MF, Davis JN, Cleveland JL, Ghysdael J, Hiebert SW. Dual control of myc expression through a single DNA binding site targeted by ets family proteins and E2F-1. Oncogene. 1994;9: 405–415.
71. Stacey DW. Three Observations That Have Changed Our Understanding of Cyclin D1 and p27 in Cell Cycle Control. Genes Cancer. 2010;1: 1189–1199. doi:10.1177/1947601911403475

72. Diehl JA, Zindy F, Sherr CJ. Inhibition of cyclin D1 phosphorylation on threonine-286 prevents its rapid degradation via the ubiquitin-proteasome pathway. Genes & Development. 1997;11: 957–972.
73. Harper JW, Elledge SJ, Keyomarsi K, Dynlacht B, Tsai LH, Zhang P, et al. Inhibition of cyclin-dependent kinases by p21. Molecular Biology of the Cell. 1995;6: 387–400.
74. Xiong Y, Hannon GJ, Zhang H, Casso D, Kobayashi R, Beach D. p21 is a universal inhibitor of cyclin kinases. Nature. 1993;366: 701–704. doi:10.1038/366701a0
75. Guo Z-Y, Hao X-H, Tan F-F, Pei X, Shang L-M, Jiang X-L, et al. The elements of human cyclin D1 promoter and regulation involved. Clin Epigenetics. 2011;2: 63–76. doi:10.1007/s13148-010-0018-y
76. Fan J, Bertino JR. Functional roles of E2F in cell cycle regulation. Oncogene. 1997;14: 1191–1200. doi:10.1038/sj.onc.1200940
77. Chellappan SP, Hiebert S, Mudryj M, Horowitz JM, Nevins JR. The E2F transcription factor is a cellular target for the RB protein. Cell. 1991;65: 1053–1061. doi:10.1016/0092-8674(91)90557-F
78. Hitomi M, Stacey DW. Cyclin D1 production in cycling cells depends on ras in a cell-cycle-specific manner. Curr Biol. 1999;9: 1075–1084. doi:10.1016/S0960-9822(99)80476-X
79. Aktas H, Cai H, Cooper GM. Ras links growth factor signaling to the cell cycle machinery via regulation of cyclin D1 and the Cdk inhibitor p27KIP1. Molecular and Cellular Biology. 1997;17: 3850–3857.
80. Daksis JI, Lu RY, Facchini LM, Marhin WW, Penn LJ. Myc induces cyclin D1 expression in the absence of de novo protein synthesis and links mitogen-stimulated signal transduction to the cell cycle. Oncogene. 1994;9: 3635–3645.
81. Mateyak M, Obaya A, Sedivy J. c-Myc regulates cyclin D-Cdk4 and-Cdk6 activity but affects cell cycle progression at multiple independent points. Mol Cell Biol. 1999;19: 4672–4683.
82. Matsumura I, Tanaka H, Kanakura Y. E2F1 and c-Myc in cell growth and death. Cell Cycle. 2003;2: 333–338.
83. Diehl JA, Cheng M, Roussel MF, Sherr CJ. Glycogen synthase kinase-3beta regulates cyclin D1 proteolysis and subcellular localization. Genes & Development. 1998;12: 3499–3511. doi:10.1101/gad.12.22.3499
84. Leung JY, Ehmann GL, Giangrande PH, Nevins JR. A role for Myc in facilitating transcription activation by E2F1. Oncogene. 2008;27: 4172–4179. doi:10.1038/onc.2008.55
85. Krek W, Ewen ME, Shirodkar S, Arany Z, Kaelin WG, Livingston DM. Negative regulation of the growth-promoting transcription factor E2F-1 by a stably bound cyclin A-dependent protein kinase. Cell. 1994;78: 161–172.
86. Xu M, Sheppard KA, Peng CY, Yee AS, Piwnica-Worms H. Cyclin A/CDK2 binds directly to E2F-1 and inhibits the DNA-binding activity of E2F-1/DP-1 by phosphorylation. Mol Cell Biol. 1994;14: 8420–8431.
87. Weintraub SJ, Prater CA, Dean DC. Retinoblastoma protein switches the E2F site from positive to negative element. Nature. 1992;358: 259–261. doi:10.1038/358259a0
88. Johnson DG, Ohtani K, Nevins JR. Autoregulatory control of E2F1 expression in response to positive and negative regulators of cell cycle progression. Genes & Development. 1994;8: 1514–1525.
89. Leone G, DeGregori J, Sears R, Jakoi L, Nevins JR. Myc and Ras collaborate in inducing accumulation of active cyclin E/Cdk2 and E2F. Nature. 1997;387: 422–426. doi:10.1038/387422a0
90. Tanaka H, Matsumura I, Ezoe S, Satoh Y, Sakamaki T, Albanese C, et al. E2F1 and c-Myc potentiate apoptosis through inhibition of NF-kappaB activity that facilitates MnSOD-mediated ROS elimination. Mol Cell. 2002;9: 1017–1029.
91. Dong P, Maddali MV, Srimani JK, Thélot F, Nevins JR, Mathey-Prevot B, et al. Division of labour between Myc and G1 cyclins in cell cycle commitment and pace control. Nat Commun. 2014;5: 4750. doi:10.1038/ncomms5750

92. Ohtani K, DeGregori J, Nevins JR. Regulation of the cyclin E gene by transcription factor E2F1. Proc Natl Acad Sci U S A. 1995;92: 12146–12150. doi:10.1073/pnas.92.26.12146

93. Lunn CL, Chrivia JC, Baldassare JJ. Activation of Cdk2/Cyclin E complexes is dependent on the origin of replication licensing factor Cdc6 in mammalian cells. Cell Cycle. 2010;9: 4533–4541. doi:10.4161/cc.9.22.13789

94. Geng Y, Lee Y-M, Welcker M, Swanger J, Zagozdzon A, Winer JD, et al. Kinase-independent function of cyclin E. Mol Cell. 2007;25: 127–139. doi:10.1016/j.molcel.2006.11.029

95. Helin K. Regulation of cell proliferation by the E2F transcription factors. Curr Opin Genet Dev. 1998;8: 28–35.

96. Sørensen CS, Syljuåsen RG, Falck J, Schroeder T, Rönnstrand L, Khanna KK, et al. Chk1 regulates the S phase checkpoint by coupling the physiological turnover and ionizing radiation-induced accelerated proteolysis of Cdc25A. Cancer Cell. 2003;3: 247–258.

97. Mazumder S, Gong B, Chen Q, Drazba JA, Buchsbaum JC, Almasan A. Proteolytic cleavage of cyclin E leads to inactivation of associated kinase activity and amplification of apoptosis in hematopoietic cells. Molecular and Cellular Biology. 2002;22: 2398–2409. doi:10.1128/MCB.22.7.2398-2409.2002

98. Knudsen KE, Fribourg AF, Strobeck MW, Blanchard JM, Knudsen ES. Cyclin A is a functional target of retinoblastoma tumor suppressor protein-mediated cell cycle arrest. J Biol Chem. 1999;274: 27632–27641.

99. Kim I-M, Ackerson T, Ramakrishna S, Tretiakova M, Wang I-C, Kalin TV, et al. The Forkhead Box m1 transcription factor stimulates the proliferation of tumor cells during development of lung cancer. Cancer Res. 2006;66: 2153–2161. doi:10.1158/0008-5472.CAN-05-3003

100. Kalin TV, Wang I-C, Ackerson TJ, Major ML, Detrisac CJ, Kalinichenko VV, et al. Increased levels of the FoxM1 transcription factor accelerate development and progression of prostate carcinomas in both TRAMP and LADY transgenic mice. Cancer Res. 2006;66: 1712–1720. doi:10.1158/0008-5472.CAN-05-3138

101. Laoukili J, Kooistra MRH, Brás A, Kauw J, Kerkhoven RM, Morrison A, et al. FoxM1 is required for execution of the mitotic programme and chromosome stability. Nature Cell Biology. 2005;7: 126–136. doi:10.1038/ncb1217

102. Alvarez-Fernández M, Halim VA, Krenning L, Aprelia M, Mohammed S, Heck AJ, et al. Recovery from a DNA-damage-induced G2 arrest requires Cdk-dependent activation of FoxM1. EMBO Rep. 2010;11: 452–458. doi:10.1038/embor.2010.46

103. Tategu M, Nakagawa H, Sasaki K, Yamauchi R, Sekimachi S, Suita Y, et al. Transcriptional regulation of human polo-like kinases and early mitotic inhibitor. J Genet Genomics. 2008;35: 215–224. doi:10.1016/S1673-8527(08)60030-2

104. Hsu JY, Reimann JDR, Sørensen CS, Lukas J, Jackson PK. E2F-dependent accumulation of hEmi1 regulates S phase entry by inhibiting APC(Cdh1). Nature Cell Biology. 2002;4: 358–366. doi:10.1038/ncb785

105. Lee J, Kim JA, Barbier V, Fotedar A, Fotedar R. DNA damage triggers p21WAF1-dependent Emi1 down-regulation that maintains G2 arrest. Mol Biol Cell. 2009;20: 1891–1902. doi:10.1091/mbc.e08-08-0818

106. Hansen DV, Loktev AV, Ban KH, Jackson PK. Plk1 regulates activation of the anaphase promoting complex by phosphorylating and triggering SCFbetaTrCP-dependent destruction of the APC Inhibitor Emi1. Molecular Biology of the Cell. 2004;15: 5623–5634. doi:10.1091/mbc.e04-07-0598

107. Pan H, Zhu Y, Wei W, Shao S, Rui X. Transcription factor FoxM1 is the downstream target of c-Myc and contributes to the development of prostate cancer. World J Surg Oncol. 2018;16: 59. doi:10.1186/s12957-018-1352-3

108. Lüscher-Firzlaff JM, Lilischkis R, Lüscher B. Regulation of the transcription factor FOXM1c by Cyclin E/CDK2. FEBS Letters. 2006;580: 1716–1722. doi:10.1016/j.febslet.2006.02.021

109. Laoukili J, Alvarez M, Meijer LAT, Stahl M, Mohammed S, Kleij L, et al. Activation of FoxM1 during G2 requires cyclin A/Cdk-dependent relief of autorepression by the FoxM1 N-terminal domain. Mol Cell Biol. 2008;28: 3076–3087. doi:10.1128/MCB.01710-07

110. Sullivan C, Liu Y, Shen J, Curtis A, Newman C, Hock JM, et al. Novel interactions between FOXM1 and CDC25A regulate the cell cycle. PLoS ONE. 2012;7: e51277. doi:10.1371/journal.pone.0051277

111. Major ML, Lepe R, Costa RH. Forkhead box M1B transcriptional activity requires binding of Cdk-cyclin complexes for phosphorylation-dependent recruitment of p300/CBP coactivators. Molecular and Cellular Biology. 2004;24: 2649–2661. doi:10.1128/MCB.24.7.2649-2661.2004

112. Fu Z, Malureanu L, Huang J, Wang W, Li H, van Deursen JM, et al. Plk1-dependent phosphorylation of FoxM1 regulates a transcriptional programme required for mitotic progression. Nature Cell Biology. 2008;10: 1076–1082. doi:10.1038/ncb1767

113. Vigo E, Müller H, Prosperini E, Hateboer G, Cartwright P, Moroni MC, et al. CDC25A phosphatase is a target of E2F and is required for efficient E2F-induced S phase. Molecular and Cellular Biology. 1999;19: 6379–6395.

114. Wu L, Goodwin EC, Naeger LK, Vigo E, Galaktionov K, Helin K, et al. E2F-Rb complexes assemble and inhibit cdc25A transcription in cervical carcinoma cells following repression of human papillomavirus oncogene expression. Molecular and Cellular Biology. 2000;20: 7059–7067.

115. Donzelli M, Squatrito M, Ganoth D, Hershko A, Pagano M, Draetta GF. Dual mode of degradation of Cdc25 A phosphatase. EMBO J. 2002;21: 4875–4884. doi:10.1093/emboj/cdf491

116. Kang T, Wei Y, Honaker Y, Yamaguchi H, Appella E, Hung M-C, et al. GSK-3 beta targets Cdc25A for ubiquitin-mediated proteolysis, and GSK-3 beta inactivation correlates with Cdc25A overproduction in human cancers. Cancer Cell. 2008;13: 36–47. doi:10.1016/j.ccr.2007.12.002

117. Chen M-S, Ryan CE, Piwnica-Worms H. Chk1 kinase negatively regulates mitotic function of Cdc25A phosphatase through 14-3-3 binding. Molecular and Cellular Biology. 2003;23: 7488–7497. doi:10.1128/MCB.23.21.7488-7497.2003

118. Hoffmann I, Draetta G, Karsenti E. Activation of the phosphatase activity of human cdc25A by a cdk2-cyclin E dependent phosphorylation at the G1/S transition. EMBO J. 1994;13: 4302–4310.

119. Mazzolini L, Broban A, Froment C, Burlet-Schiltz O, Besson A, Manenti S, et al. Phosphorylation of CDC25A on SER283 in late S/G2 by CDK/cyclin complexes accelerates mitotic entry. Cell Cycle. 2016;15: 2742–2752. doi:10.1080/15384101.2016.1220455

120. Mailand N, Podtelejnikov AV, Groth A, Mann M, Bartek J, Lukas J. Regulation of G(2)/M events by Cdc25A through phosphorylation-dependent modulation of its stability. EMBO J. 2002;21: 5911–5920. doi:10.1093/emboj/cdf567

121. Geley S, Kramer E, Gieffers C, Gannon J, Peters JM, Hunt T. Anaphase-promoting complex/cyclosome-dependent proteolysis of human cyclin A starts at the beginning of mitosis and is not subject to the spindle assembly checkpoint. The Journal of Cell Biology. 2001;153: 137–148.

122. Elzen den N, Pines J. Cyclin A is destroyed in prometaphase and can delay chromosome alignment and anaphase. The Journal of Cell Biology. 2001;153: 121–136.

123. Di Fiore B, Pines J. How cyclin A destruction escapes the spindle assembly checkpoint. J Cell Biol. 2010;190: 501–509. doi:10.1083/jcb.201001083

124. Blomberg I, Hoffmann I. Ectopic expression of Cdc25A accelerates the G(1)/S transition and leads to premature activation of cyclin E- and cyclin A-dependent kinases. Mol Cell Biol. 1999;19: 6183–6194.

125. Timofeev O, Cizmecioglu O, Hu E, Orlik T, Hoffmann I. Human Cdc25A phosphatase has a non-redundant function in G2 phase by activating Cyclin A-dependent kinases. FEBS Letters. 2009;583: 841–847. doi:10.1016/j.febslet.2009.01.044

126. Rape M, Kirschner MW. Autonomous regulation of the anaphase-promoting complex couples mitosis to S-phase entry. Nature. 2004;432: 588–595. doi:10.1038/nature03023

127. Machida YJ, Dutta A. The APC/C inhibitor, Emi1, is essential for prevention of rereplication. Genes & Development. 2007;21: 184–194. doi:10.1101/gad.1495007

128. Reimann JD, Freed E, Hsu JY, Kramer ER, Peters JM, Jackson PK. Emi1 is a mitotic regulator that interacts with Cdc20 and inhibits the anaphase promoting complex. Cell. 2001;105: 645–655.

129. Reimann JD, Gardner BE, Margottin-Goguet F, Jackson PK. Emi1 regulates the anaphase-promoting complex by a different mechanism than Mad2 proteins. Genes & Development. 2001;15: 3278–3285. doi:10.1101/gad.945701

130. Zhou BB, Li H, Yuan J, Kirschner MW. Caspase-dependent activation of cyclin-dependent kinases during Fas-induced apoptosis in Jurkat cells. Proc Natl Acad Sci U S A. 1998;95: 6785–6790.

131. Lee J, Kumagai A, Dunphy WG. Positive regulation of Wee1 by Chk1 and 14-3-3 proteins. Molecular Biology of the Cell. 2001;12: 551–563. doi:10.1091/mbc.12.3.551

132. Domínguez-Kelly R, Martín Y, Koundrioukoff S, Tanenbaum ME, Smits VAJ, Medema RH, et al. Wee1 controls genomic stability during replication by regulating the Mus81-Eme1 endonuclease. J Cell Biol. 2011;194: 567–579. doi:10.1083/jcb.201101047

133. Watanabe N, Arai H, Iwasaki J-I, Shiina M, Ogata K, Hunter T, et al. Cyclin-dependent kinase (CDK) phosphorylation destabilizes somatic Wee1 via multiple pathways. Proc Natl Acad Sci U S A. 2005;102: 11663–11668. doi:10.1073/pnas.0500410102

134. Deibler RW, Kirschner MW. Quantitative reconstitution of mitotic CDK1 activation in somatic cell extracts. Mol Cell. 2010;37: 753–767. doi:10.1016/j.molcel.2010.02.023

135. Leung TW, Lin SS, Tsang AC, Tong CS, Ching JC, Leung WY, et al. Over-expression of FoxM1 stimulates cyclin B1 expression. FEBS Letters. 2001;507: 59–66.

136. Alvarez B, Martínez-A C, Burgering BM, Carrera AC. Forkhead transcription factors contribute to execution of the mitotic programme in mammals. Nature. 2001;413: 744–747. doi:10.1038/35099574

137. Harper JW, Burton JL, Solomon MJ. The anaphase-promoting complex: it's not just for mitosis any more. Genes & Development. 2002;16: 2179–2206. doi:10.1101/gad.1013102

138. Wang I-C, Chen Y-J, Hughes D, Petrovic V, Major ML, Park HJ, et al. Forkhead box M1 regulates the transcriptional network of genes essential for mitotic progression and genes encoding the SCF (Skp2-Cks1) ubiquitin ligase. Molecular and Cellular Biology. 2005;25: 10875–10894. doi:10.1128/MCB.25.24.10875-10894.2005

139. Dutertre S, Cazales M, Quaranta M, Froment C, Trabut V, Dozier C, et al. Phosphorylation of CDC25B by Aurora-A at the centrosome contributes to the G2-M transition. J Cell Sci. 2004;117: 2523–2531. doi:10.1242/jcs.01108

140. Dutertre S, Descamps S, Prigent C. On the role of aurora-A in centrosome function. Oncogene. 2002;21: 6175–6183. doi:10.1038/sj.onc.1205775

141. Lindon C, Pines J. Ordered proteolysis in anaphase inactivates Plk1 to contribute to proper mitotic exit in human cells. The Journal of Cell Biology. 2004;164: 233–241. doi:10.1083/jcb.200309035

142. Kotani S, Tugendreich S, Fujii M, Jorgensen PM, Watanabe N, Hoog C, et al. PKA and MPF-activated polo-like kinase regulate anaphase-promoting complex activity and mitosis progression. Mol Cell. 1998;1: 371–380.

143. Qian YW, Erikson E, Li C, Maller JL. Activated polo-like kinase Plx1 is required at multiple points during mitosis in Xenopus laevis. Molecular and Cellular Biology. 1998;18: 4262–4271.

144. Thomas Y, Cirillo L, Panbianco C, Martino L, Tavernier N, Schwager F, et al. Cdk1 Phosphorylates SPAT-1/Bora to Promote Plk1 Activation in C. elegans and Human Cells. Cell Rep. 2016;15: 510–518. doi:10.1016/j.celrep.2016.03.049

145. Gheghiani L, Loew D, Lombard B, Mansfeld J, Gavet O. PLK1 Activation in Late G2 Sets Up Commitment to Mitosis. Cell Rep. 2017;19: 2060–2073. doi:10.1016/j.celrep.2017.05.031

146. Lobjois V, Jullien D, Bouché J-P, Ducommun B. The polo-like kinase 1 regulates CDC25B-dependent mitosis entry. Biochim Biophys Acta. 2009;1793: 462–468. doi:10.1016/j.bbamcr.2008.12.015
147. Ouyang B, Li W, Pan H, Meadows J, Hoffmann I, Dai W. The physical association and phosphorylation of Cdc25C protein phosphatase by Prk. Oncogene. 1999;18: 6029–6036. doi:10.1038/sj.onc.1202983
148. Cogswell JP, Brown CE, Bisi JE, Neill SD. Dominant-negative polo-like kinase 1 induces mitotic catastrophe independent of cdc25C function. Cell Growth Differ. 2000;11: 615–623.
149. De Souza CP, Ellem KA, Gabrielli BG. Centrosomal and cytoplasmic Cdc2/cyclin B1 activation precedes nuclear mitotic events. Experimental Cell Research. 2000;257: 11–21. doi:10.1006/excr.2000.4872
150. Jackman M, Lindon C, Nigg EA, Pines J. Active cyclin B1-Cdk1 first appears on centrosomes in prophase. Nature Cell Biology. 2003;5: 143–148. doi:10.1038/ncb918
151. Lindqvist A, Källström H, Lundgren A, Barsoum E, Rosenthal CK. Cdc25B cooperates with Cdc25A to induce mitosis but has a unique role in activating cyclin B1-Cdk1 at the centrosome. The Journal of Cell Biology. 2005;171: 35–45. doi:10.1083/jcb.200503066
152. Lopez-Girona A, Furnari B, Mondesert O, Russell P. Nuclear localization of Cdc25 is regulated by DNA damage and a 14-3-3 protein. Nature. 1999;397: 172–175. doi:10.1038/16488
153. Schmitt E, Boutros R, Froment C, Monsarrat B, Ducommun B, Dozier C. CHK1 phosphorylates CDC25B during the cell cycle in the absence of DNA damage. J Cell Sci. 2006;119: 4269–4275. doi:10.1242/jcs.03200
154. Krämer A, Mailand N, Lukas C, Syljuåsen RG, Wilkinson CJ, Nigg EA, et al. Centrosome-associated Chk1 prevents premature activation of cyclin-B-Cdk1 kinase. Nature Cell Biology. 2004;6: 884–891. doi:10.1038/ncb1165
155. Hoffmann I, Clarke PR, Marcote MJ, Karsenti E, Draetta G. Phosphorylation and activation of human cdc25-C by cdc2--cyclin B and its involvement in the self-amplification of MPF at mitosis. EMBO J. 1993;12: 53–63.
156. Jackman MR, Pines JN. Cyclins and the G2/M transition. Cancer Surv. 1997;29: 47–73.
157. Heald R, McLoughlin M, McKeon F. Human wee1 maintains mitotic timing by protecting the nucleus from cytoplasmically activated Cdc2 kinase. Cell. 1993;74: 463–474.
158. Shteinberg M, Protopopov Y, Listovsky T, Brandeis M, Hershko A. Phosphorylation of the cyclosome is required for its stimulation by Fizzy/cdc20. Biochemical and Biophysical Research Communications. 1999;260: 193–198. doi:10.1006/bbrc.1999.0884
159. Golan A, Yudkovsky Y, Hershko A. The cyclin-ubiquitin ligase activity of cyclosome/APC is jointly activated by protein kinases Cdk1-cyclin B and Plk. J Biol Chem. 2002;277: 15552–15557. doi:10.1074/jbc.M111476200
160. Manchado E, Eguren M, Malumbres M. The anaphase-promoting complex/cyclosome (APC/C): cell-cycle-dependent and -independent functions. Biochem Soc Trans. 2010;38: 65–71. doi:10.1042/BST0380065
161. Peters J-M. The anaphase promoting complex/cyclosome: a machine designed to destroy. Nat Rev Mol Cell Biol. 2006;7: 644–656. doi:10.1038/nrm1988
162. D'Angiolella V, Mari C, Nocera D, Rametti L, Grieco D. The spindle checkpoint requires cyclin-dependent kinase activity. Genes & Development. 2003;17: 2520–2525. doi:10.1101/gad.267603
163. Hein JB, Nilsson J. Interphase APC/C-Cdc20 inhibition by cyclin A2-Cdk2 ensures efficient mitotic entry. Nat Commun. 2016;7: 10975. doi:10.1038/ncomms10975
164. Avram S, Mernea M, Mihailescu DF, Seiman CD, Seiman DD, Putz MV. Mitotic checkpoint proteins Mad1 and Mad2 - structural and functional relationship with implication in genetic diseases. Curr Comput Aided Drug Des. 2014;10: 168–181.
165. Nezi L, Musacchio A. Sister chromatid tension and the spindle assembly checkpoint. Current opinion in cell biology. 2009;21: 785–795. doi:10.1016/j.ceb.2009.09.007

166. Thornton BR, Toczyski DP. Precise destruction: an emerging picture of the APC. Genes & Development. 2006;20: 3069–3078. doi:10.1101/gad.1478306

167. Kramer ER, Scheuringer N, Podtelejnikov AV, Mann M, Peters JM. Mitotic regulation of the APC activator proteins CDC20 and CDH1. Molecular Biology of the Cell. 2000;11: 1555–1569. doi:10.1091/mbc.11.5.1555

168. Wang W, Kirschner MW. Emi1 preferentially inhibits ubiquitin chain elongation by the anaphase-promoting complex. Nat Cell Biol. 2013;15: 797–806. doi:10.1038/ncb2755

169. Ohtani K, DeGregori J, Leone G, Herendeen DR, Kelly TJ, Nevins JR. Expression of the HsOrc1 gene, a human ORC1 homolog, is regulated by cell proliferation via the E2F transcription factor. Molecular and Cellular Biology. 1996;16: 6977–6984.

170. DePamphilis ML, Blow JJ, Ghosh S, Saha T, Noguchi K, Vassilev A. Regulating the licensing of DNA replication origins in metazoa. Curr Opin Cell Biol. 2006;18: 231–239. doi:10.1016/j.ceb.2006.04.001

171. Pelizon C, d'Adda di Fagagna F, Farrace L, Laskey RA. Human replication protein Cdc6 is selectively cleaved by caspase 3 during apoptosis. EMBO reports. 2002;3: 780–784. doi:10.1093/embo/reports/kvf161

172. Petersen BO, Lukas J, Sørensen CS, Bartek J, Helin K. Phosphorylation of mammalian CDC6 by cyclin A/CDK2 regulates its subcellular localization. EMBO J. 1999;18: 396–410. doi:10.1093/emboj/18.2.396

173. Yan Z, DeGregori J, Shohet R, Leone G, Stillman B, Nevins JR, et al. Cdc6 is regulated by E2F and is essential for DNA replication in mammalian cells. Proc Natl Acad Sci U S A. 1998;95: 3603–3608.

174. Yim H, Erikson RL. Cell division cycle 6, a mitotic substrate of polo-like kinase 1, regulates chromosomal segregation mediated by cyclin-dependent kinase 1 and separase. Proc Natl Acad Sci U S A. 2010;107: 19742–19747. doi:10.1073/pnas.1013557107

175. Valovka T, Schönfeld M, Raffeiner P, Breuker K, Dunzendorfer-Matt T, Hartl M, et al. Transcriptional control of DNA replication licensing by Myc. Sci Rep. 2013;3: 3444. doi:10.1038/srep03444

176. Yoshida K, Inoue I. Regulation of Geminin and Cdt1 expression by E2F transcription factors. Oncogene. 2004;23: 3802–3812. doi:10.1038/sj.onc.1207488

177. Yanagi K-I, Mizuno T, You Z, Hanaoka F. Mouse geminin inhibits not only Cdt1-MCM6 interactions but also a novel intrinsic Cdt1 DNA binding activity. J Biol Chem. 2002;277: 40871–40880. doi:10.1074/jbc.M206202200

178. García-Higuera I, Manchado E, Dubus P, Cañamero M, Méndez J, Moreno S, et al. Genomic stability and tumour suppression by the APC/C cofactor Cdh1. Nat Cell Biol. 2008;10: 802–811. doi:10.1038/ncb1742

179. Clijsters L, Ogink J, Wolthuis R. The spindle checkpoint, APC/C(Cdc20), and APC/C(Cdh1) play distinct roles in connecting mitosis to S phase. J Cell Biol. 2013;201: 1013–1026. doi:10.1083/jcb.201211019

180. Fragkos M, Ganier O, Coulombe P, Méchali M. DNA replication origin activation in space and time. Nat Rev Mol Cell Bio. 2015;16: 360–374. doi:10.1038/nrm4002

181. Ren B, Cam H, Takahashi Y, Volkert T, Terragni J, Young RA, et al. E2F integrates cell cycle progression with DNA repair, replication, and G(2)/M checkpoints. Genes & Development. 2002;16: 245–256. doi:10.1101/gad.949802

182. Cam H, Dynlacht BD. Emerging roles for E2F: beyond the G1/S transition and DNA replication. Cancer Cell. 2003;3: 311–316.

183. Coverley D, Laman H, Laskey RA. Distinct roles for cyclins E and A during DNA replication complex assembly and activation. Nature Cell Biology. 2002;4: 523–528. doi:10.1038/ncb813

184. Mailand N, Falck J, Lukas C, Syljuâsen RG, Welcker M, Bartek J, et al. Rapid destruction of human Cdc25A in response to DNA damage. Science. 2000;288: 1425–1429.

185. Donzelli M, Draetta GF. Regulating mammalian checkpoints through Cdc25 inactivation. EMBO reports. 2003;4: 671–677. doi:10.1038/sj.embor.embor887

186. Katsuno Y, Suzuki A, Sugimura K, Okumura K, Zineldeen DH, Shimada M, et al. Cyclin A-Cdk1 regulates the origin firing program in mammalian cells. Proc Natl Acad Sci U S A. 2009;106: 3184–3189. doi:10.1073/pnas.0809350106

187. Petermann E, Maya-Mendoza A, Zachos G, Gillespie DAF, Jackson DA, Caldecott KW. Chk1 requirement for high global rates of replication fork progression during normal vertebrate S phase. Molecular and Cellular Biology. 2006;26: 3319–3326. doi:10.1128/MCB.26.8.3319-3326.2006

188. Petronczki M, Glotzer M, Kraut N, Peters J-M. Polo-like kinase 1 triggers the initiation of cytokinesis in human cells by promoting recruitment of the RhoGEF Ect2 to the central spindle. Dev Cell. 2007;12: 713–725. doi:10.1016/j.devcel.2007.03.013

189. Burkard ME, Randall CL, Larochelle S, Zhang C, Shokat KM, Fisher RP, et al. Chemical genetics reveals the requirement for Polo-like kinase 1 activity in positioning RhoA and triggering cytokinesis in human cells. Proc Natl Acad Sci U S A. 2007;104: 4383–4388. doi:10.1073/pnas.0701140104

190. Uhlmann F, Lottspeich F, Nasmyth K. Sister-chromatid separation at anaphase onset is promoted by cleavage of the cohesin subunit Scc1. Nature. 1999;400: 37–42. doi:10.1038/21831

191. Listovsky T, Sale JE. Sequestration of CDH1 by MAD2L2 prevents premature APC/C activation prior to anaphase onset. J Cell Biol. 2013;203: 87–100. doi:10.1083/jcb.201302060

192. Sánchez I, Dynlacht BD. New insights into cyclins, CDKs, and cell cycle control. Semin Cell Dev Biol. 2005;16: 311–321. doi:10.1016/j.semcdb.2005.02.007

193. Petronczki M, Lénárt P, Peters J-M. Polo on the Rise—from Mitotic Entry to Cytokinesis with Plk1. Dev Cell. 2008;14: 646–659. doi:10.1016/j.devcel.2008.04.014

194. Schmit TL, Zhong W, Setaluri V, Spiegelman VS, Ahmad N. Targeted depletion of Polo-like kinase (Plk) 1 through lentiviral shRNA or a small-molecule inhibitor causes mitotic catastrophe and induction of apoptosis in human melanoma cells. J Invest Dermatol. 2009;129: 2843–2853. doi:10.1038/jid.2009.172

195. Vecchione A, Baldassarre G, Ishii H, Nicoloso MS, Belletti B, Petrocca F, et al. Fez1/Lzts1 absence impairs Cdk1/Cdc25C interaction during mitosis and predisposes mice to cancer development. Cancer Cell. 2007;11: 275–289. doi:10.1016/j.ccr.2007.01.014

196. Yuan Z, Becker EBE, Merlo P, Yamada T, DiBacco S, Konishi Y, et al. Activation of FOXO1 by Cdk1 in cycling cells and postmitotic neurons. Science. 2008;319: 1665–1668. doi:10.1126/science.1152337

197. Floyd S, Pines J, Lindon C. APC/C Cdh1 targets aurora kinase to control reorganization of the mitotic spindle at anaphase. Curr Biol. 2008;18: 1649–1658. doi:10.1016/j.cub.2008.09.058

198. Guicciardi ME, Gores GJ. Life and death by death receptors. The FASEB Journal. Federation of American Societies for Experimental Biology; 2009;23: 1625–1637. doi:10.1096/fj.08-111005

199. Pennarun B, Meijer A, de Vries EGE, Kleibeuker JH, Kruyt F, de Jong S. Playing the DISC: turning on TRAIL death receptor-mediated apoptosis in cancer. Biochim Biophys Acta. 2010;1805: 123–140. doi:10.1016/j.bbcan.2009.11.004

200. Slee EA, Harte MT, Kluck RM, Wolf BB, Casiano CA, Newmeyer DD, et al. Ordering the cytochrome c-initiated caspase cascade: hierarchical activation of caspases-2, -3, -6, -7, -8, and -10 in a caspase-9-dependent manner. The Journal of Cell Biology. 1999;144: 281–292.

201. Cowling V, Downward J. Caspase-6 is the direct activator of caspase-8 in the cytochrome c-induced apoptosis pathway: absolute requirement for removal of caspase-6 prodomain. Cell Death and Differentiation. 2002;9: 1046–1056. doi:10.1038/sj.cdd.4401065

202. Li H, Bergeron L, Cryns V, Pasternack MS, Zhu H, Shi L, et al. Activation of caspase-2 in apoptosis. J Biol Chem. 1997;272: 21010–21017.

203. Slee EA, Adrain C, Martin SJ. Executioner caspase-3, -6, and -7 perform distinct, non-redundant roles during the demolition phase of apoptosis. J Biol Chem. 2001;276: 7320–7326. doi:10.1074/jbc.M008363200

204. Shah RB, Thompson R, Sidi S. A mitosis-sensing caspase activation platform? New insights into the PIDDosome. Molecular & Cellular Oncology. 2016;3: e1059921. doi: 10.1080/23723556.2015.1059921

205. Vogel C, Kienitz A, Müller R, Bastians H. The mitotic spindle checkpoint is a critical determinant for topoisomerase-based chemotherapy. J Biol Chem. 2005;280: 4025–4028. doi:10.1074/jbc.C400545200

206. Masuda A, Maeno K, Nakagawa T, Saito H, Takahashi T. Association between mitotic spindle checkpoint impairment and susceptibility to the induction of apoptosis by anti-microtubule agents in human lung cancers. Am J Pathol. 2003;163: 1109–1116. doi:10.1016/S0002-9440(10)63470-0

207. Andersen JL, Johnson CE, Freel CD, Parrish AB, Day JL, Buchakjian MR, et al. Restraint of apoptosis during mitosis through interdomain phosphorylation of caspase-2. The EMBO Journal. 2009;28: 3216–3227. doi:10.1038/emboj.2009.253

208. Sung E-S, Park K-J, Choi H-J, Kim C-H, Kim Y-S. The proteasome inhibitor MG132 potentiates TRAIL receptor agonist-induced apoptosis by stabilizing tBid and Bik in human head and neck squamous cell carcinoma cells. Experimental Cell Research. 2012;318: 1564–1576. doi:10.1016/j.yexcr.2012.04.003

209. Lin C-F, Tsai C-C, Huang W-C, Wang Y-C, Tseng P-C, Tsai T-T, et al. Glycogen Synthase Kinase-3β and Caspase-2 Mediate Ceramide- and Etoposide-Induced Apoptosis by Regulating the Lysosomal-Mitochondrial Axis. PLoS ONE. 2016;11: e0145460. doi:10.1371/journal.pone.0145460

210. Wertz IE, Kusam S, Lam C, Okamoto T, Sandoval W, Anderson DJ, et al. Sensitivity to antitubulin chemotherapeutics is regulated by MCL1 and FBW7. Nature. 2011;471: 110–114. doi:10.1038/nature09779

211. Harley ME, Allan LA, Sanderson HS, Clarke PR. Phosphorylation of Mcl-1 by CDK1-cyclin B1 initiates its Cdc20-dependent destruction during mitotic arrest. The EMBO Journal. 2010;29: 2407–2420. doi:10.1038/emboj.2010.112

212. Croxton R, Ma Y, Song L, Haura EB, Cress WD. Direct repression of the Mcl-1 promoter by E2F1. Oncogene. 2002;21: 1359–1369. doi:10.1038/sj.onc.1205157

213. Ding Q, Huo L, Yang J-Y, Xia W, Wei Y, Liao Y, et al. Down-regulation of myeloid cell leukemia-1 through inhibiting Erk/Pin 1 pathway by sorafenib facilitates chemosensitization in breast cancer. Cancer Research. 2008;68: 6109–6117. doi:10.1158/0008-5472.CAN-08-0579

214. Townsend KJ, Trusty JL, Traupman MA, Eastman A, Craig RW. Expression of the antiapoptotic MCL1 gene product is regulated by a mitogen activated protein kinase-mediated pathway triggered through microtubule disruption and protein kinase C. Oncogene. 1998;17: 1223–1234. doi:10.1038/sj.onc.1202035

215. Maurer U, Charvet C, Wagman AS, Dejardin E, Green DR. Glycogen synthase kinase-3 regulates mitochondrial outer membrane permeabilization and apoptosis by destabilization of MCL-1. Mol Cell. 2006;21: 749–760. doi:10.1016/j.molcel.2006.02.009

216. Clem RJ, Cheng EH, Karp CL, Kirsch DG, Ueno K, Takahashi A, et al. Modulation of cell death by Bcl-XL through caspase interaction. Proc Natl Acad Sci U S A. 1998;95: 554–559.

217. Yang E, Zha J, Jockel J, Boise LH, Thompson CB, Korsmeyer SJ. Bad, a heterodimeric partner for Bcl-XL and Bcl-2, displaces Bax and promotes cell death. Cell. 1995;80: 285–291.

218. Gélinas C, White E. BH3-only proteins in control: specificity regulates MCL-1 and BAK-mediated apoptosis. Genes & Development. 2005;19: 1263–1268. doi:10.1101/gad.1326205

219. Bah N, Maillet L, Ryan J, Dubreil S, Gautier F, Letai A, et al. Bcl-xL controls a switch between cell death modes during mitotic arrest. Cell Death Dis. 2014;5: e1291–e1291. doi:10.1038/cddis.2014.251

220. Weiß LM, Hugle M, Romero S, Fulda S. Synergistic induction of apoptosis by a polo-like kinase 1 inhibitor and microtubule-interfering drugs in Ewing sarcoma cells. Int J Cancer. 2016;138: 497–506. doi:10.1002/ijc.29725
221. Terrano DT, Upreti M, Chambers TC. Cyclin-dependent kinase 1-mediated Bcl-xL/Bcl-2 phosphorylation acts as a functional link coupling mitotic arrest and apoptosis. Mol Cell Biol. 2010;30: 640–656. doi:10.1128/MCB.00882-09
222. Zhou L, Cai X, Han X, Xu N, Chang DC. CDK1 switches mitotic arrest to apoptosis by phosphorylating Bcl-2/Bax family proteins during treatment with microtubule interfering agents. Cell Biol Int. 2014;38: 737–746. doi:10.1002/cbin.10259
223. Kirsch DG, Doseff A, Chau BN, Lim DS, de Souza-Pinto NC, Hansford R, et al. Caspase-3-dependent cleavage of Bcl-2 promotes release of cytochrome c. J Biol Chem. 1999;274: 21155–21161.
224. O'Connor L, Strasser A, O'Reilly LA, Hausmann G, Adams JM, Cory S, et al. Bim: a novel member of the Bcl-2 family that promotes apoptosis. EMBO J. 1998;17: 384–395. doi:10.1093/emboj/17.2.384
225. Elangovan B, Chinnadurai G. Functional dissection of the pro-apoptotic protein Bik. Heterodimerization with anti-apoptosis proteins is insufficient for induction of cell death. J Biol Chem. 1997;272: 24494–24498.
226. Eichhorn JM, Sakurikar N, Alford SE, Chu R, Chambers TC. Critical role of anti-apoptotic Bcl-2 protein phosphorylation in mitotic death. Cell Death Dis. 2013;4: e834–e834. doi:10.1038/cddis.2013.360
227. Condorelli F, Salomoni P, Cotteret S, Cesi V, Srinivasula SM, Alnemri ES, et al. Caspase cleavage enhances the apoptosis-inducing effects of BAD. Molecular and Cellular Biology. 2001;21: 3025–3036. doi:10.1128/MCB.21.9.3025-3036.2001
228. Taghiyev AF, Guseva NV, Harada H, Knudson CM, Rokhlin OW, Cohen MB. Overexpression of BAD potentiates sensitivity to tumor necrosis factor-related apoptosis-inducing ligand treatment in the prostatic carcinoma cell line LNCaP. Mol Cancer Res. 2003;1: 500–507.
229. Datta SR, Dudek H, Tao X, Masters S, Fu H, Gotoh Y, et al. Akt phosphorylation of BAD couples survival signals to the cell-intrinsic death machinery. Cell. 1997;91: 231–241.
230. Fang X, Yu S, Eder A, Mao M, Bast RC, Boyd D, et al. Regulation of BAD phosphorylation at serine 112 by the Ras-mitogen-activated protein kinase pathway. Oncogene. 1999;18: 6635–6640. doi:10.1038/sj.onc.1203076
231. Tan Y, Demeter MR, Ruan H, Comb MJ. BAD Ser-155 phosphorylation regulates BAD/Bcl-XL interaction and cell survival. J Biol Chem. 2000;275: 25865–25869. doi:10.1074/jbc.M004199200
232. Boyd JM, Gallo GJ, Elangovan B, Houghton AB, Malstrom S, Avery BJ, et al. Bik, a novel death-inducing protein shares a distinct sequence motif with Bcl-2 family proteins and interacts with viral and cellular survival-promoting proteins. Oncogene. 1995;11: 1921–1928.
233. Gillissen B, Essmann F, Hemmati PG, Richter A, Richter A, Oztop I, et al. Mcl-1 determines the Bax dependency of Nbk/Bik-induced apoptosis. J Cell Biol. 2007;179: 701–715. doi:10.1083/jcb.200703040
234. Dijkers PF, Medema RH, Lammers JW, Koenderman L, Coffer PJ. Expression of the pro-apoptotic Bcl-2 family member Bim is regulated by the forkhead transcription factor FKHR-L1. Curr Biol. 2000;10: 1201–1204.
235. Hongisto V, Smeds N, Brecht S, Herdegen T, Courtney MJ, Coffey ET. Lithium blocks the c-Jun stress response and protects neurons via its action on glycogen synthase kinase 3. Molecular and Cellular Biology. 2003;23: 6027–6036. doi:10.1128/MCB.23.17.6027-6036.2003
236. Hughes R, Gilley J, Kristiansen M, Ham J. The MEK-ERK pathway negatively regulates bim expression through the 3' UTR in sympathetic neurons. BMC Neurosci. 2011;12: 69. doi:10.1186/1471-2202-12-69

237. Gomez-Bougie P, Bataille R, Amiot M. The imbalance between Bim and Mcl-1 expression controls the survival of human myeloma cells. Eur J Immunol. 2004;34: 3156–3164. doi:10.1002/eji.200424981
238. Yamada H, Tada-Oikawa S, Uchida A, Kawanishi S. TRAIL causes cleavage of bid by caspase-8 and loss of mitochondrial membrane potential resulting in apoptosis in BJAB cells. Biochemical and Biophysical Research Communications. 1999;265: 130–133. doi:10.1006/bbrc.1999.1641
239. Huang K, Zhang J, O'Neill KL, Gurumurthy CB, Quadros RM, Tu Y, et al. Cleavage by Caspase 8 and Mitochondrial Membrane Association Activate the BH3-only Protein Bid during TRAIL-induced Apoptosis. Journal of Biological Chemistry. 2016;291: 11843–11851. doi:10.1074/jbc.M115.711051
240. Li H, Zhu H, Xu CJ, Yuan J. Cleavage of BID by caspase 8 mediates the mitochondrial damage in the Fas pathway of apoptosis. Cell. 1998;94: 491–501.
241. Upton J-P, Austgen K, Nishino M, Coakley KM, Hagen A, Han D, et al. Caspase-2 cleavage of BID is a critical apoptotic signal downstream of endoplasmic reticulum stress. Mol Cell Biol. 2008;28: 3943–3951. doi:10.1128/MCB.00013-08
242. Kim H, Rafiuddin-Shah M, Tu H-C, Jeffers JR, Zambetti GP, Hsieh JJ-D, et al. Hierarchical regulation of mitochondrion-dependent apoptosis by BCL-2 subfamilies. Nature Cell Biology. 2006;8: 1348–1358. doi:10.1038/ncb1499
243. Wei MC, Lindsten T, Mootha VK, Weiler S, Gross A, Ashiya M, et al. tBID, a membrane-targeted death ligand, oligomerizes BAK to release cytochrome c. Genes & Development. 2000;14: 2060–2071.
244. Sarosiek KA, Chi X, Bachman JA, Sims JJ, Montero J, Patel L, et al. BID preferentially activates BAK while BIM preferentially activates BAX, affecting chemotherapy response. Mol Cell. 2013;51: 751–765. doi:10.1016/j.molcel.2013.08.048
245. Mathai JP, Germain M, Shore GC. BH3-only BIK regulates BAX,BAK-dependent release of Ca2+ from endoplasmic reticulum stores and mitochondrial apoptosis during stress-induced cell death. J Biol Chem. 2005;280: 23829–23836. doi:10.1074/jbc.M500800200
246. Lee EF, Grabow S, Chappaz S, Dewson G, Hockings C, Kluck RM, et al. Physiological restraint of Bak by Bcl-xL is essential for cell survival. Genes & Development. 2016;30: 1240–1250. doi:10.1101/gad.279414.116
247. Zhai D, Jin C, Huang Z, Satterthwait AC, Reed JC. Differential regulation of Bax and Bak by anti-apoptotic Bcl-2 family proteins Bcl-B and Mcl-1. J Biol Chem. 2008;283: 9580–9586. doi:10.1074/jbc.M708426200
248. Willis SN, Chen L, Dewson G, Wei A, Naik E, Fletcher JI, et al. Proapoptotic Bak is sequestered by Mcl-1 and Bcl-xL, but not Bcl-2, until displaced by BH3-only proteins. Genes & Development. 2005;19: 1294–1305. doi:10.1101/gad.1304105
249. Dai H, Meng XW, Lee S-H, Schneider PA, Kaufmann SH. Context-dependent Bcl-2/Bak interactions regulate lymphoid cell apoptosis. Journal of Biological Chemistry. 2009;284: 18311–18322. doi:10.1074/jbc.M109.004770
250. Germain M, Milburn J, Duronio V. MCL-1 inhibits BAX in the absence of MCL-1/BAX Interaction. J Biol Chem. 2008;283: 6384–6392. doi:10.1074/jbc.M707762200
251. Oltvai Z, MILLIMAN C, KORSMEYER S. Bcl-2 Heterodimerizes in-Vivo with a Conserved Homolog, Bax, That Accelerates Programmed Cell-Death. 1993;74: 609–619.
252. Manon S, Chaudhuri B, Guérin M. Release of cytochrome c and decrease of cytochrome c oxidase in Bax-expressing yeast cells, and prevention of these effects by coexpression of Bcl-xL. FEBS Letters. 1997;415: 29–32.
253. Wei MC, Zong WX, Cheng EH, Lindsten T, Panoutsakopoulou V, Ross AJ, et al. Proapoptotic BAX and BAK: a requisite gateway to mitochondrial dysfunction and death. Science. 2001;292: 727–730. doi:10.1126/science.1059108

254. Zhou L, Chang DC. Dynamics and structure of the Bax-Bak complex responsible for releasing mitochondrial proteins during apoptosis. J Cell Sci. 2008;121: 2186–2196. doi:10.1242/jcs.024703

255. Hu P, Han Z, Couvillon AD, Exton JH. Critical role of endogenous Akt/IAPs and MEK1/ERK pathways in counteracting endoplasmic reticulum stress-induced cell death. J Biol Chem. 2004;279: 49420–49429. doi:10.1074/jbc.M407700200

256. Du C, Fang M, Li Y, Li L, Wang X. Smac, a mitochondrial protein that promotes cytochrome c-dependent caspase activation by eliminating IAP inhibition. Cell. 2000;102: 33–42.

257. Deveraux Q, Roy N, Stennicke H, Van Arsdale T, Zhou Q, Srinivasula S, et al. IAPs block apoptotic events induced by caspase-8 and cytochrome c by direct inhibition of distinct caspases. The EMBO Journal. 1998;17: 2215–2223. doi:10.1093/emboj/17.8.2215

258. Srinivasula SM, Ahmad M, Fernandes-Alnemri T, Alnemri ES. Autoactivation of procaspase-9 by Apaf-1-mediated oligomerization. Mol Cell. 1998;1: 949–957.

259. Riedl SJ, Renatus M, Schwarzenbacher R, Zhou Q, Sun C, Fesik SW, et al. Structural basis for the inhibition of caspase-3 by XIAP. 2001;104: 791–800.

260. Li P, Nijhawan D, Budihardjo I, Srinivasula S, Ahmad M, Alnemri E, et al. Cytochrome c and dATP-dependent formation of Apaf-1/caspase-9 complex initiates an apoptotic protease cascade. 1997;91: 479–489.

261. Stennicke HR, Jürgensmeier JM, Shin H, Deveraux Q, Wolf BB, Yang X, et al. Pro-caspase-3 is a major physiologic target of caspase-8. J Biol Chem. 1998;273: 27084–27090.

262. Spencer SL, Gaudet S, Albeck JG, Burke JM, Sorger PK. Non-genetic origins of cell-to-cell variability in TRAIL-induced apoptosis. Nature. 2009;459: 428–432. doi:10.1038/nature08012

263. Wolf BB, Schuler M, Echeverri F, Green DR. Caspase-3 is the primary activator of apoptotic DNA fragmentation via DNA fragmentation factor-45/inhibitor of caspase-activated DNase inactivation. J Biol Chem. 1999;274: 30651–30656.
