# Supplementary material for: Boolean model of growth signaling, cell cycle and apoptosis predicts the molecular mechanism of aberrant cell cycle progression driven by hyperactive PI3K
Source: PLoS Comput Biol. 2019 Mar 15;15(3):e1006402. doi: 10.1371/journal.pcbi.1006402 (PMC6436762; doi:10.1371/journal.pcbi.1006402)
Supplement: S2 Table — (A) Expression profile of synchronous model attractor states, numbered to match Fig 3; orange/blue: ON/OFF. (B) explanation of the molecular signatures allowing us to match them to cellular phenotypes. (PDF) [file pcbi.1006402.s016.pdf]

S2 Table. Attractors of the synchronous Boolean model

S2.A		Apoptotic Attractors						Quiescent Attractors		Cell Cycle Attractor																				
	Attr. #	1	2	3	4	5	6	7	8	9																				
Modules	Node									1	2	3	4	5	6	7	8	9	10	11	12	13	14	15	16	17	18	19	20	21
	GF_High	0	0	1	0	0	1	0	0	1	1	1	1	1	1	1	1	1	1	1	1	1	1	1	1	1	1	1	1	1
	GF	0	1	1	0	1	1	1	1	1	1	1	1	1	1	1	1	1	1	1	1	1	1	1	1	1	1	1	1	1
GF signaling - upstream PI3K cycle	RTK	0	0	0	0	0	0	1	1	1	1	1	1	1	1	1	1	1	1	1	1	1	1	1	1	1	1	1	1	1
	Grb2	0	0	0	0	0	0	0	0	1	1	1	1	1	1	1	1	1	1	1	1	1	1	1	1	1	1	1	1	1
	SOS	0	0	0	0	0	0	0	0	1	1	1	1	1	1	1	1	1	1	1	1	1	1	1	1	1	1	1	1	1
	Ras	0	0	0	0	0	0	0	0	1	1	1	1	1	1	1	1	1	1	1	1	1	1	1	1	1	1	1	1	1
	RAF	0	0	0	0	0	0	0	0	1	1	1	1	1	1	1	1	1	1	1	1	1	1	1	1	1	1	1	1	1
	MEK	0	0	0	0	0	0	0	0	1	1	1	1	1	1	1	1	1	1	1	1	1	1	1	1	1	1	1	1	1
	ERK	0	0	0	0	0	0	0	0	1	1	1	1	1	1	1	1	1	1	1	1	1	1	1	1	1	1	1	1	1
	mTORC2	1	1	1	1	1	1	1	1	1	1	1	1	1	1	1	1	1	1	1	1	1	1	1	1	1	1	1	1	1
	PI3K	0	0	0	0	0	0	1	1	1	1	1	1	1	1	1	1	1	1	1	1	1	1	1	1	1	1	1	1	1
	PIP3	0	0	0	0	0	0	1	1	1	1	1	1	1	1	1	1	1	1	1	1	1	1	1	1	1	1	1	1	1
	PDK1	0	0	0	0	0	0	1	1	1	1	1	1	1	1	1	1	1	1	1	1	1	1	1	1	1	1	1	1	1
	AKT_B	0	0	0	0	0	0	1	1	1	1	1	1	1	1	1	1	1	1	1	1	1	1	1	1	1	1	1	1	1
GF signaling - PI3K cycle	p110_H	1	1	1	1	1	1	1	1	1	1	1	1	1	1	0	0	0	0	1	1	1	1	1	1	0	0	0	0	0
	PI3K_H	0	0	0	0	0	0	0	0	0	1	1	1	1	1	1	0	0	0	0	1	1	1	1	1	1	0	0	0	0
	AKT_H	0	0	0	0	0	0	0	0	0	0	1	1	1	1	1	0	0	0	0	0	1	1	1	1	1	0	0	0	0
	FoxO3	1	1	1	1	1	1	1	1	1	1	1	0	0	0	0	0	1	1	1	1	1	0	0	0	0	0	0	0	1
	PLCgamma	0	0	0	0	0	0	0	0	0	0	1	1	1	1	1	0	0	0	0	0	1	1	1	1	1	0	0	0	0
	IP3	0	0	0	0	0	0	0	0	0	0	0	1	1	1	1	1	0	0	0	0	0	1	1	1	1	1	0	0	0
	Ca2+	0	0	0	0	0	0	0	0	0	0	0	0	1	1	1	1	1	0	0	0	0	0	1	1	1	1	1	0	0
	NeddL4	0	0	0	0	0	0	0	0	0	0	0	0	0	1	1	1	1	0	0	0	0	0	0	1	1	1	1	0	0
GF signaling - downstream PI3K cycle	FoxO1	1	1	1	1	1	1	1	1	1	1	1	0	0	0	0	0	1	1	1	1	0	0	0	0	0	0	0	0	1
	p21_mRNA	1	1	1	1	1	1	1	1	1	1	1	1	0	0	0	0	0	1	1	1	1	0	0	0	0	0	0	0	0
	TSC2	1	1	1	1	1	1	1	1	1	1	1	0	0	0	0	0	1	1	1	1	1	0	0	0	0	0	1	1	1
	PRAS40	1	1	1	1	1	1	1	1	0	0	0	0	0	0	0	0	0	0	0	1	1	0	0	0	0	0	0	0	0
	DAG	0	0	0	0	0	0	0	0	0	0	0	1	1	1	1	1	0	0	0	0	0	1	1	1	1	1	0	0	0
	Rheb	0	0	0	0	0	0	0	0	0	0	0	0	1	1	1	1	1	0	0	0	0	0	1	1	1	1	1	0	0
	mTORC1	0	0	0	0	0	0	0	0	1	1	1	1	1	1	1	1	1	1	0	0	0	0	0	1	1	1	1	1	1
	S6K	0	0	0	0	0	0	0	0	1	1	1	1	1	1	1	1	1	1	1	0	0	0	0	0	1	1	1	1	1
	eIF4E	0	0	0	0	0	0	0	0	1	1	1	1	1	1	1	1	1	1	1	0	0	0	0	0	1	1	1	1	1
	GSK3	1	1	1	1	1	1	1	1	0	0	0	0	0	0	0	0	0	0	0	0	1	0	0	0	0	0	0	0	0
Restriction Switch	p21	0	0	0	0	0	0	1	1	0	1	1	0	0	0	0	0	0	0	1	1	1	1	0	0	0	0	0	0	0
	pRB	0	0	0	0	0	0	1	1	0	0	0	0	0	0	0	0	0	0	0	0	0	0	0	0	0	0	0	0	0
	p27Kip1	0	0	0	0	0	0	1	1	0	0	0	0	0	0	0	0	0	1	1	0	1	0	0	0	0	0	0	0	0
	Myc	0	0	0	0	0	0	0	0	1	1	1	1	1	1	1	1	1	1	1	1	1	0	1	1	1	1	1	1	1
	CyclinD1	0	0	0	0	0	0	0	0	1	1	1	1	1	1	1	1	0	0	1	0	0	0	0	1	1	1	1	1	1
	E2F1	0	0	0	0	0	0	0	0	1	1	1	1	1	1	0	0	0	0	0	0	0	0	0	0	1	1	1	1	1
	CyclinE	0	0	0	0	0	0	0	0	0	0	1	1	1	1	1	0	0	0	0	0	0	0	0	0	0	0	0	0	0
Origin licensing	ORC	0	0	0	0	0	0	0	0	1	1	1	1	1	1	1	0	0	0	0	0	0	0	0	0	0	1	1	1	1
	Cdc6	0	0	0	0	0	0	0	0	1	1	1	1	1	1	1	0	0	0	0	0	0	0	0	0	0	0	0	0	1
	Cdt1	0	0	0	0	0	0	0	0	1	1	1	1	1	1	0	0	0	0	0	0	0	0	0	0	0	0	0	0	0
	Pre-RC	0	0	0	0	0	0	0	0	0	1	1	1	1	1	1	0	0	0	0	0	0	0	0	0	0	0	0	0	0
	geminin	0	0	0	0	0	0	0	0	0	0	0	0	0	0	0	0	0	0	0	0	0	0	0	0	0	1	0	0	0
Cell Cycle Phase Switch	CyclinA_mRNA	0	0	0	0	0	0	0	0	1	1	1	1	1	1	1	1	1	1	1	1	1	1	1	1	1	1	1	1	1
	Emi1	1	1	1	1	1	1	0	0	1	1	1	1	1	1	1	1	1	1	1	1	1	1	1	0	0	0	0	1	1
	FoxM1	0	0	0	0	0	0	0	0	0	0	0	1	1	1	1	1	1	1	1	1	1	1	1	1	1	1	1	0	0
	Cdc25A	0	0	0	0	0	0	0	0	0	0	0	0	1	1	1	1	1	1	1	1	1	1	1	1	1	1	1	1	0
	CyclinA	0	0	0	0	0	0	0	0	0	0	0	0	0	1	1	1	1	1	1	1	1	1	1	0	0	0	0	0	0
	Wee1	0	0	0	0	0	0	0	0	0	0	0	0	0	0	1	1	1	1	0	0	0	0	0	0	0	0	0	0	0
	UbcH10	0	0	0	0	0	0	0	0	0	0	0	0	0	0	0	1	1	1	1	1	1	1	1	1	1	1	1	1	1
	CyclinB	0	0	0	0	0	0	0	0	0	0	0	0	0	0	0	1	1	1	1	1	1	1	1	1	1	1	0	0	0
	Cdc25B	0	0	0	0	0	0	0	0	0	0	0	0	0	0	0	1	1	1	1	1	1	1	1	1	1	1	1	1	0
	Plk1	0	0	0	0	0	0	0	0	0	0	0	0	0	0	0	0	0	0	0	1	1	1	1	1	1	1	1	0	0
	Cdc25C	0	0	0	0	0	0	0	0	0	0	0	0	0	0	0	0	0	0	0	0	1	1	1	1	1	1	1	1	0
	Cdk1	0	0	0	0	0	0	0	0	0	0	0	0	0	0	0	0	0	0	0	0	0	1	1	1	1	1	1	0	0
	pAPC	0	0	0	0	0	0	0	0	0	0	0	0	0	0	0	0	0	0	0	0	0	0	1	1	1	1	1	1	1
	Cdc20	0	0	0	0	0	0	0	0	0	0	0	0	0	0	0	0	0	0	0	0	0	0	0	0	0	1	1	1	0
	Cdh1	1	1	1	1	1	1	1	1	1	1	1	1	1	1	0	0	0	0	0	0	0	0	0	0	0	0	0	1	1
Cell cycle processes	Replication	0	0	0	0	0	0	0	0	0	0	0	0	0	1	1	0	0	0	0	0	0	0	0	0	0	0	0	0	0
	ATR	0	0	0	0	0	0	0	0	0	0	0	0	0	0	1	1	0	0	0	0	0	0	0	0	0	0	0	0	0
	CHK1	0	0	0	0	0	0	0	0	0	0	0	0	0	0	0	1	1	0	0	0	0	0	0	0	0	0	0	0	0
	4N_DNA	0	0	0	0	0	0	0	1	0	0	0	0	0	0	1	1	1	1	1	1	1	1	1	1	1	1	1	1	1
	U_Kinetochores	0	0	0	0	0	0	0	0	0	0	0	0	0	0	0	0	0	0	0	0	0	0	1	1	0	0	0	0	0
	Mad2	0	0	0	0	0	0	0	0	0	0	0	0	0	0	0	0	0	0	0	0	0	0	0	1	0	0	0	0	0
	A_Kinetochores	0	0	0	0	0	0	0	0	0	0	0	0	0	0	0	0	0	0	0	0	0	0	0	1	1	1	0	0	0
	Plk1_H	0	0	0	0	0	0	0	0	0	0	0	0	0	0	0	0	0	0	0	0	1	1	1	1	1	1	1	1	0
	Ect2	0	0	0	0	0	0	0	0	0	0	0	0	0	0	0	0	0	0	0	0	0	0	0	0	0	0	0	0	1
	Trail	1	1	1	0	0	0	0	0	0	0	0	0	0	0	0	0	0	0	0	0	0	0	0	0	0	0	0	0	0
Apoptotic Switch	DR4_5	1	1	1	0	0	0	0	0	0	0	0	0	0	0	0	0	0	0	0	0	0	0	0	0	0	0	0	0	0
	Casp8	1	1	1	1	1	1	0	0	0	0	0	0	0	0	0	0	0	0	0	0	0	0	0	0	0	0	0	0	0
	Casp2	1	1	1	1	1	1	0	0	0	0	0	0	0	0	0	0	0	0	0	0	0	0	0	0	0	0	0	0	0
	MCL-1	0	0	0	0	0	0	1	1	1	1	1	1	1	1	1	1	1	1	1	1	1	1	1	0	0	1	1	1	1
	BCLXL	0	0	0	0	0	0	1	1	1	1	1	1	1	1	1	1	1	1	1	1	1	1	1	1	0	1	1	1	1
	BCL2	0	0	0	0	0	0	1	1	1	1	1	1	1	1	1	1	1	1	1	1	1	1	1	1	1	1	1	1	1
	BAD	1	1	1	1	1	1	0	0	0	0	0	0	0	0	0	0	0	0	0	0	0	0	0	0	0	0	0	0	0
	BIK	1	1	1	1	1	1	0	0	0	0	0	0	0	0	0	0	0	0	0	0	0	0	0	0	0	0	0	0	0
	BIM	1	1	1	1	1	1	0	0	0	0	0	0	0	0	0	0	0	0	0	0	0	0	0	0	0	0	0	0	0
	BID	1	1	1	1	1	1	0	0	0	0	0	0	0	0	0	0	0	0	0	0	0	0	0	0	0	0	0	0	0
	BAK	1	1	1	1	1	1	0	0	0	0	0	0	0	0	0	0	0	0	0	0	0	0	0	0	0	0	0	0	0
	BAX	1	1	1	1	1	1	0	0	0	0	0	0	0	0	0	0	0	0	0	0	0	0	0	0	0	0	0	0	0
	Cyto_C	1	1	1	1	1	1	0	0	0	0	0	0	0	0	0	0	0	0	0	0	0	0	0	0	0	0	0	0	0
	SMAC	1	1	1	1	1	1	0	0	0	0	0	0	0	0	0	0	0	0	0	0	0	0	0	0	0	0	0	0	0
	IAPs	0	0	0	0	0	0	1	1	1	1	1	1	1	1	1	1	1	1	1	1	1	1	1	1	1	1	1	1	1
	Casp9	1	1	1	1	1	1	0	0	0	0	0	0	0	0	0	0	0	0	0	0	0	0	0	0	0	0	0	0	0
	Casp3	1	1	1	1	1	1	0	0	0	0	0	0	0	0	0	0	0	0	0	0	0	0	0	0	0	0	0	0	0
	CAD	1	1	1	1	1	1	0	0	0	0	0	0	0	0	0	0	0	0	0	0	0	0	0	0	0	0	0	0	0

S2.B

Attractor states	Phenotype	Molecular signature
**1 - 6**	**Apoptosis**	All pro-apoptotic nodes in the apoptotic switch are ON, including Caspase 3 and CAD (Caspase Activated DNAase).
**7 - 8**	**Quiescence**	Restriction Switch stable in its uncommitted state, no cycling behavior in the Phase Switch. Only anti-anti-apoptotic genes are on within the apoptotic module.
**Limit cycle attractor 9 - state along cycle**		
**9 -> 1**	**G1**	Restriction Switch flips completely into a committed state, Origin loading and licensing occurs, and Cyclin E then Cyclin A levels rise (only anti-anti-apoptotic genes are on).
**9 -> 2**		
**9 -> 3**		
**9 -> 4**		
**9 -> 5**		
**9 -> 6**	**S**	**Replication node is ON.**
**9 -> 7**		
**9 -> 8**	**G2**	Replication is OFF and the cell has 4N DNA content. Once DNA damage checkpoint signaling stops (CKH1 turns OFF), there is a Plk1 —> Cdc25C —> Cdk1/Cyclin B activation cascade that initiates pro-metaphase.
**9 -> 9**		
**9 -> 10**		
**9 -> 11**		
**9 -> 12**		
**9 -> 13**		
**9 -> 14**	**Prometaphase**	Cdk1/Cyclin B complexes are active.
**9 -> 15**	**Metaphase**	Mitotic spindle forms, some kinetochores remain unattached (U_Kinetochores node is ON), SAC not yet satisfied.
**9 -> 16**		
**9 -> 17**	**Metaphase (SAC)**	Mitotic spindle is complete, SAC passage begins: kinetochores are still (all) attached (A_Kinetochores node is ON), but $pAPC^{Cdc20}$ activation begins.
**9 -> 18**		
**9 -> 19**	**Anaphase**	The spindle is pulled apart (A_Kinetochores node is OFF), $pAPC^{Cdc20}$ is ON but $pAPC^{Cdh1}$ is still OFF.
**9 -> 20**	**Telophase**	$pAPC^{Cdh1}$ is reactivated, cell still has 4N DNA content.
**9 -> 21**	**Cytokinesis**	The Ect2 node is ON, representing the accumulation of this RhoGEF to the central spindle, where it aids the enrichment of GTP-bound RhoA and thus the formation of the contractile ring.
