# Supplementary material for: Boolean model of growth signaling, cell cycle and apoptosis predicts the molecular mechanism of aberrant cell cycle progression driven by hyperactive PI3K
Source: PLoS Comput Biol. 2019 Mar 15;15(3):e1006402. doi: 10.1371/journal.pcbi.1006402 (PMC6436762; doi:10.1371/journal.pcbi.1006402)

S3 Table. Biased update order and rationale

Node	Nonrandom order applies	Process impacted	Details	
Update at the **start** of each time-step (first in list updated first):				
**Pre_RC**	**ON**	G1→S→G2 Top image in table.	Updated early to avoid being turned off in the update round where *Cdt1* is inhibited by the joint action of CyclinA and E, so that it remains available to turn on 4N_DNA. In cells, the loss of licensed replication origins is complete only when DNA synthesis is finalized, meaning the loss of Pre-RC before 4N_DNA is non-physiological.	
Replication	**OFF**	G1→S→G2 Top image in table.	When OFF, updated early but *after* Pre-RC, so that a) it is present at the start of the update round that may lead to loss of *Cdt1 of CyclinA* is activated before *Cdt1.* This round then ends with 4N_DNA turning on. Moreover, *Replication* represents a process that should be considered ON from the moment the conditions for its activation are met.	
U_Kinetochores	**OFF**	M → Cyto Bottom image.	By turning *U_Kinetochores ON* early and turning *A_Kinetochores ON* late, we shorten the metaphase window and help cells avoid tipping the balance of pro- and anti-apoptotic signals.	
A_Kinetochores	**ON**	M → Cyto Bottom image.	By updating *U_Kinetochores* early once it's ON, we guarantee that it remains ON for the entire time-step in which Cdc20 is activated. This ensures that it remains on for an entire time-step, until explicitly turned off by APC. This helps ensure that *Emi1* does not turn ON prematurely, compromising the subsequent activation of *Cdh1* (required for cytokinesis).	
Plk1_H	**ON**	M → Cyto Bottom image.	By updating *U_Kinetochores* early once it's ON, we guarantee that it remains ON for the time-step in *FoxM1* and *Plk1* are degraded, and remains available when *Cdh1* is active to turn on *Ect2*.	
**CyclinB**	**ON**	M → Cyto Bottom image.	Once *Cyclin B* is degraded, its absence is rapidly felt by a wide array of substrates. Making sure that its transition from ON to OFF can influence its targets in a single timestep	
Cdc20	**OFF**	M → Cyto Bottom image.	By updating Cdc20 early when it's OFF, but *after* *U_Kinetochores* turn ON, we guarantee that the signal from *CyclinB / Cdk1* that turns on *U_Kinetochores* propagates to Mad2 in the same time-step (this is realistic, as *Mad2* can rapidly bind to unattached kinetochores the moment metaphase starts). This blocks premature *Cdc20* activation in the absence of Mad2, as the same *CyclinB / Cdk1* complex also phosphorylates *pAPC* later in this timestep.	
Update at the **end** of each time-step (first in list updated **last**):				
Replication	**ON**	G1→S→G2 Top image in table.	We attempt to turn OFF *Replication* only after 4N_DNA is updated to guarantee that once 4N_DNA is ON, *Replication* ends.	
**4N_DNA**	**ON**	M → Cyto Bottom image.	Turning 4N_DNA OFF *after* Ect2 in the time steps where *Ect2* goes from OFF (below) to ON guarantees that 4N_DNA correctly tracks cytokinesis.	

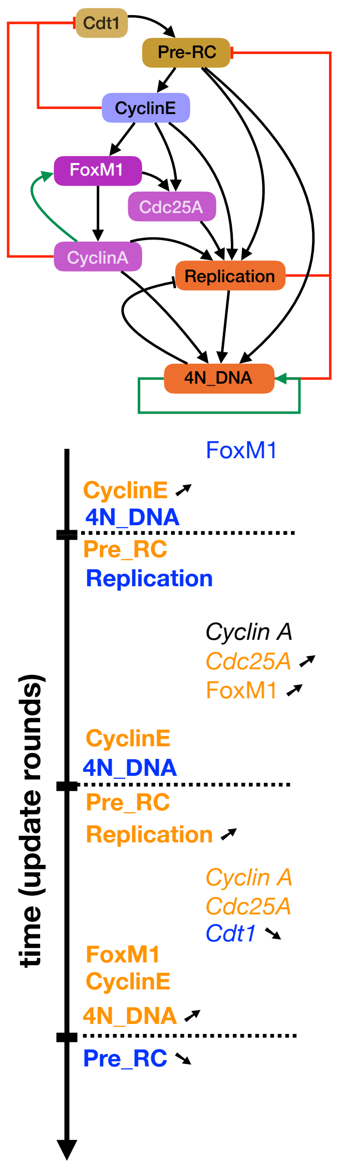

Update at the **end** of each time-step (first in list updated **last**):			
**4N_DNA**	**OFF**	G1→S→G2 Top image in table.	4N_DNA is a marker node that keeps track of the amount of DNA during division. It has a "self-activating" link to remain ON once the cell successfully replicates its DNA until the *Ect2* node representing the contractile ring and cytokinesis turns it OFF. By updating it nearly last in every time-step (but before *Replication* if S-phase is ongoing and may turn off), we guarantee that the same time-step in which *Replication* turns on but *Cdt1 is also inhibited by the same set of conditions (*firing of Pre-RC origins) does not end without 4N_DNA turning on. Otherwise Pre-RC is lost in the next timestep and the model does not register the completion of DNA synthesis.
**Ect2**	**OFF**	M → Cyto Bottom image.	*Ect2* = OFF indicates that cytokinesis has not yet been triggered. By only allowing *Ect2* to turn ON at the end of an update but before the 4N_DNA node, we guarantee that it does not flicker ON and OFF between two 4N_DNA updates and fails to trigger cytokinesis. Our model only includes the *Ect2* node to mark the assembly of a contractile ring (modeling failures of cytokinesis after this ring forms is beyond our scope), and thus we assume that even a short-lived *Ect2* = ON state results in 4N_DNA = OFF by the end of the time-step.
A_Kinetochores	**OFF**	M → Cyto Bottom image.	Turning on *A_Kinetochores* late (after *U_kinetochores* is already ON) guarantees that it is turned on as early as the conditions allow. Coupled with its early update in its ON state, this ensures that it remains on for an entire time-step, until explicitly turned off by APC. This helps ensure that *Emi1* does not turn ON prematurely, compromising the subsequent activation of *Cdh1* (required for cytokinesis).
**CyclinE**	**ON**	G1→S→G2 Top image in table.	By updating *CyclinE after FoxM1,* we make sure that the loss of *CyclinE* does not immediately affect *FoxM1.*
**FoxM1**	**ON**	G1→S→G2 Top image in table.	Aids handoff of *FoxM1* activation from *Cyclin E* to *Cyclin A* and engage the feedback loop that keeps both on in G2 (green arrow). By updating *FoxM1* at the end of a round once it's off, we guarantee that Cyclin A is on, even if it was updated before *FoxM1* in the round *FoxM1* was activated by *Cyclin E.*
Cdc20	**ON**	M → Cyto Bottom image.	$pAPC^{Cdc20}$ activity in cells is short-lived, but the degradation of its targets is necessary for the timely activation of APC^{Cdh1} . By only allowing *Cdc20* to turn back OFF after *all* it's targets are turned OFF in the same time-step (*A_kinetochores* as well as *Cyclin B*), we guarantee that *Cdh1* activity is not blocked by re-activation of *Emi1* before the *Ect2* node (which requires *Cdh1* and $Plk1_H$) turns on.
Plk1_H	**OFF**	M → Cyto Bottom image.	By only allowing $Plk1_H$ to turn ON close to the end of an update and OFF only at the start, we make sure that its own auto-regulatory loop that keep it ON for a full time-step. If during this next step (when $Plk1_H$ is updated early and remains ON) the main *Plk1* pool is degraded by APC^{Cdh1}, then it is important to guarantee that the joint presence of $Plk1_H$ and APC^{Cdh1} triggers *Ect2* activation (updated late in this time-step). In the next step, $Plk1_H$ will indeed turn off early into the update.

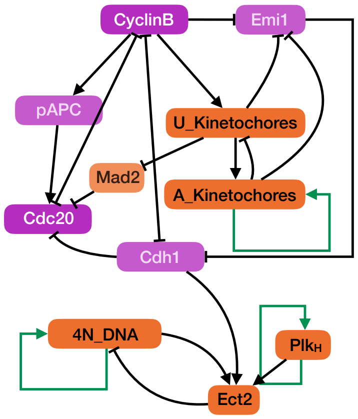

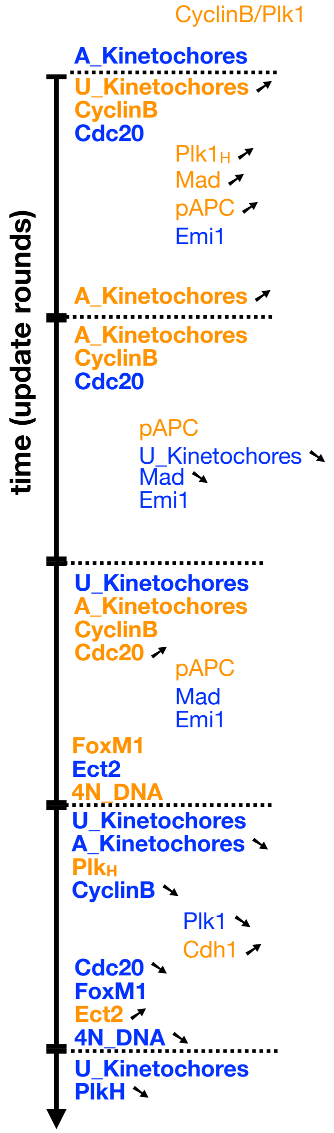

Supplement: S3 Table — Details and logic of the biased update order required to accurately reproduce cell cycle progression, including figures (last column) that summarize relevant regulatory feed-forward and feedback loops susceptible to non-biological signal propagation under fully asynchronous update, mitigated by the early/late update bias on the nodes listed in the table. Black/red/green arrows: feed-forward / negative feedback / positive feedback; node color: module membership according to Fig 3; translucent nodes: updated in random order. Time traces under each network show the order of biased update among these nodes during normal cell cycle progression; dashed horizontal line: time-step (update-round) boundary; orange/blue: ON/OFF; black up/down arrows: timestep in which nodes turn ON/OFF. (PDF) [file pcbi.1006402.s017.pdf]
