# Supplementary material for: Boolean model of growth signaling, cell cycle and apoptosis predicts the molecular mechanism of aberrant cell cycle progression driven by hyperactive PI3K
Source: PLoS Comput Biol. 2019 Mar 15;15(3):e1006402. doi: 10.1371/journal.pcbi.1006402 (PMC6436762; doi:10.1371/journal.pcbi.1006402)
Supplement: S4 Table — Rows: independent in silico knockout / over-expression experiment (downward/upward arrows), performed in stochastic non-saturating environments indicated in column 3. Figure panels (columns 5–6): changes to normal cell cycle and/or apoptosis as a function of inhibition / overexpression strength (x-axis). Each stacked bar graph shows the relative occurrence of normal cell cycle completion (mustard), G2 → G1 reset followed by genome duplication (purple), aberrant mitosis followed by genome duplication (turquoise), failed cytokinesis followed by genome duplication (blue) and apoptosis (dark red) as a function of node inhibition/overexpression, relative to the cell cycle rate in wild-type cells (black dashed line) with synchronous (left) and biased order asynchronous update (right). Sampling: 50,000 time-steps. (PDF) [file pcbi.1006402.s018.pdf]

## S4 Table. Knockout and overexertion model behaviors vs. experimental data

| Model behavior | | | | | | Experimental support |
|---|---|---|---|---|---|---|
| **KO/ OE** | **Node** | **Env. sign** | **Description** | **Synchronous result** | **Biased asynch. result** | |
| | | | | ***x*-axis:** % inhibition/over-expression<br>y-axis: occurrence in 100 time-steps<br>Normal cell cycle<br>G2 → G1 → Genome dupl.<br>Aneuploidy + Genome dupl.<br>No cytokinesis → Genome dupl.<br>Apoptosis | | |
| **Growth Signaling Module** | | | | | | |
| ↘ | ***Ras*** | *95% $GF_H$* | ***Ras*** knockdown limits proliferation under strong mitogenic stimulation. | 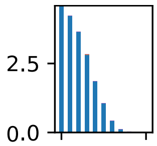 | 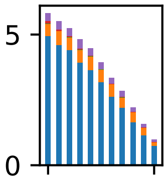 | *Ras* is a known proto-oncogene; constitutive activation of Ras is found in a variety of tumors. It activates *MAPK* and PI3K signaling, and is a potent inducer of proliferation and survival signaling [1]. |
| ↗ | | *20% Trail (no $GF_H$)* | ***Ras*** hyper activation enhances proliferation and blocks *Trail*-mediated apoptosis. | 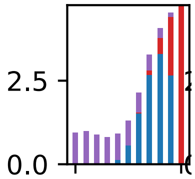 | 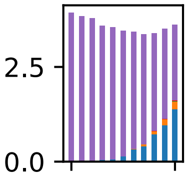 | |
| ↘ | ***ERK*** | *80% $GF_H$* | ***ERK*** knockdown limits proliferation under strong mitogenic stimulation. | 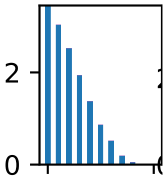 | 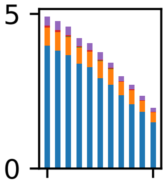 | As the often oncogenic pro-proliferative *MAPK* signaling cascade goes through ERK, ints inhibition is under investigation as a potentially potent cancer drug against a range of tumors with MAPK pathway mutations [2]. |
| ↗ | | *50% $GF_H$* | ***ERK*** hyper activation enhances proliferation under weak mitogenic stimualtion. | 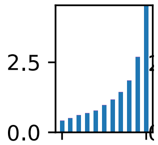 | 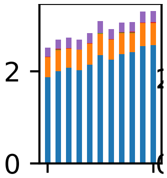 | |
| ↗ | | *50% GF (no $GF_H$)* | ***ERK*** hyper activation blocks apoptosis in the absence of survival signals. | 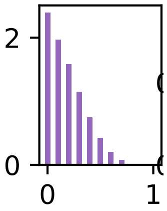 | 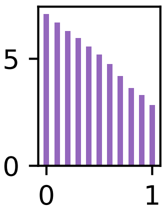 | |

| | | | | | | |
|---|---|---|---|---|---|---|
| ↘ | ***mTORC2*** | *95% $GF_H$* | ***mTORC2*** knockdown limits proliferation under strong mitogenic stimulation. | 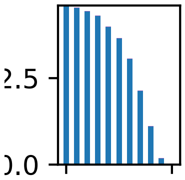 | 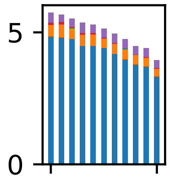 | Inhibition of the *mTORC2* subunit *Rictor* blocks proliferation and promotes apoptosis in gastric cancer [3] and glioblastoma [4]. |
| | | *50% GF (no $GF_H$)* | ***mTORC2*** knockdown enhances apoptosis in response to a loss of survival signals. | 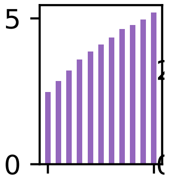 | 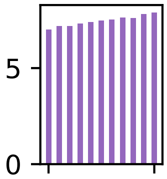 | |
| ↘ | ***PDK1*** | *80% $GF_H$* | ***PDK1*** knockdown limits proliferation under strong mitogenic stimulation. | 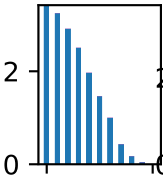 | 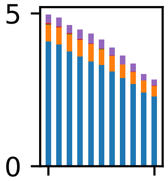 | PDK1 inhibition by RNAi in melanoma and colon cancer cells resulted in significant cell growth inhibition and apoptosis [5]. |
| | | *50% Trail* | ***PDK1*** knockdown potentiates Trail-induced apoptosis in cycling cells. | 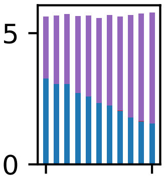 | 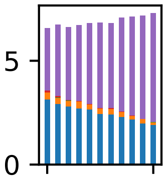 | |
| ↘ | ***PLCγ*** | *95% $GF_H$* | ***PLCγ*** knockdown enriches for cells that fail to complete cytokinesis. | 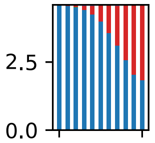 | 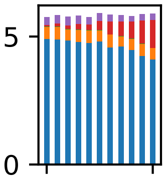 | ***prediction*** |
| | | *50% Trail* | ***PLCγ*** knockdown protects against *Trail*-mediated apoptosis in high growth factor environments. | 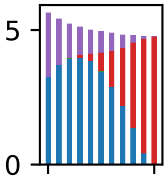 | 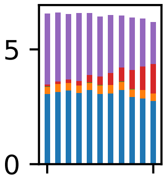 | ***prediction*** |
| ↘ | ***NeddL4*** | *95% $GF_H$* | ***NeddL4*** knockdown enriches for cells that fail to complete cytokinesis. | 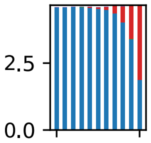 | 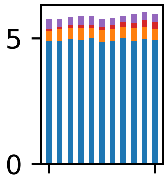 | ***prediction*** |
| | | *50% Trail* | ***NeddL4*** knockdown protects against apoptosis in high growth factor environments. | 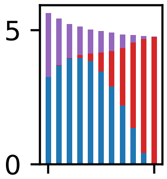 | 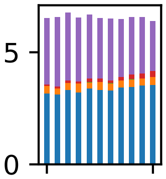 | ***prediction*** |

| | | | | | | |
|---|---|---|---|---|---|---|
| ↗ | ***FoxO1*** | *50% $GF_H$* | ***FoxO1*** over expression slows down proliferation. | 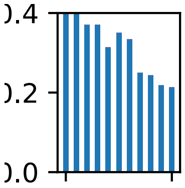 | 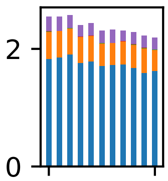 | Overexpression of the active form of FOXO family members promotes cell cycle arrest at the G1/S boundary in a variety of cell lines [6]. |
| ↗ | ***TSC2*** | *80% $GF_H$* | ***mTORC1*** over expression slows down proliferation. | 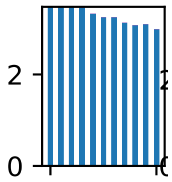 | 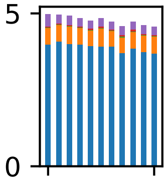 | Over-expression of TSC2 slows proliferation in oral cancer cell lines [7]. and increased the fraction of human osteosarcoma cells arrested in G1 [8]. |
| ↘ | ***Rheb*** | *65% $GF_H$* | ***Rheb*** inhibition slows proliferation under moderate mitogenic stimulation. | 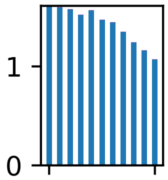 | 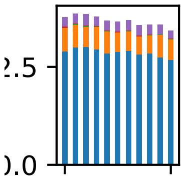 | Fruitfly Rheb promotes cell cycle progression and cell growth in vivo and in vitro [9]. |
| ↘ | ***mTORC1*** | *65% $GF_H$* | ***mTORC1*** inhibition severely limits proliferation under moderate mitogenic stimulation. | 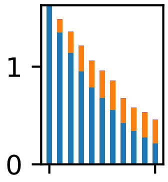 | 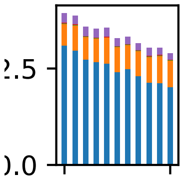 | Inhibition of *mTORC1* blocks both cell growth (in volume) and cell cycle progression [10]. |
| ↘ | ***S6K*** | *50% Trail* | ***S6K*** inhibition increases *Trail*-mediated apoptosis in cycling cells. | 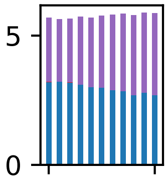 | 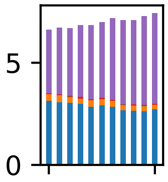 | Silencing S6K lead to a substantial increase in TNF-α as well as TRAIL-mediated apoptosis [11]. |
| ↘ | ***eIF4E*** | *65% $GF_H$* | ***eIF4E*** inhibition decreases cell proliferation at non-saturating mitogenic exposure, and leads to a small fraction of endo-reduplicating cells. | 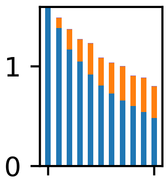 | 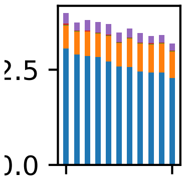 | 4E-BPs inhibit cell proliferation via inhibitory binding to *eIF4E,* and selectively blocking translation of proteins involved in cell cycle progression [10]. |
| ↘ | ***GSK3*** | *20% $GF_H$* | ***GSK3*** inhibition increases proliferation and cell cycle entry under low mitogenic stimulation | 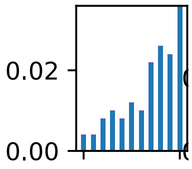 | 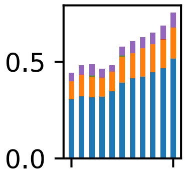 | GSK-3 inhibitors maintain proliferative capacity in mouse embryonic stem cells (ESCs) as well as mammalian cardiomyocytes [12]. |

| Restriction Switch & Origin of Replication Licensing Switch | | | | | | |
|---|---|---|---|---|---|---|
| ↘ | ***p21*** | *50% $GF_H$* | ***p21*** inhibition increases proliferation rate in the presence of moderate growth stimulation. | 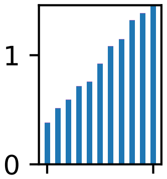 | 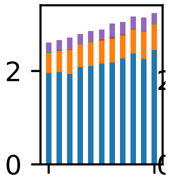 | *p21*-null cells were more likely than their wild-type counterparts to commit to another division before finding the previous one [13]. Generally, p21 is known to promote cell cycle arrest in response to several stimuli [14]. |
| ↘ | ***pRB*** | *35% $GF_H$* | ***pRB*** inhibition is a potent inducer of proliferation under weak mitogenic stimulation. | 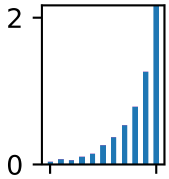 | 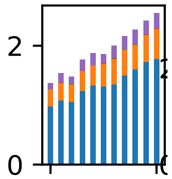 | The *RB1* gene and its protein product, retinoblastoma protein (*pRB*) is the first known tumor suppressor; its loss leads to aberrant cell cycle entry and a lack of an effective G1/S checkpoint [15]. |
| ↗ | ***p27$^{Kip1}$*** | *95% $GF_H$* | ***p27$^{Kip1}$*** up regulation slows down proliferation. | 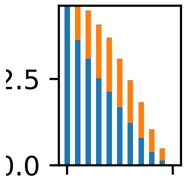 | 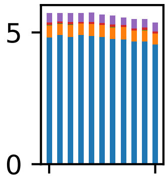 | Overexpression of p27KIP1 induced cell cycle arrest in G1 phase in a hepatoma cell line [16]. |
| ↘ | ***Myc*** | *95% $GF_H$* | ***Myc*** inhibition severely limits proliferation even under strong mitogenic stimulation. | 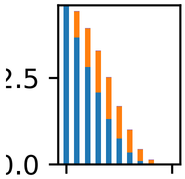 | 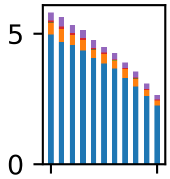 | *Myc* is the most frequently amplified human oncogene. orchestrates the transcriptional regulation of cell growth, cell-cycle progression, metabolism, and cell survival. As a result, it is a key therapeutic cancer target [17]. |
| ↗ | | *35% $GF_H$* | ***Myc*** over-expressioon promotes proliferation even under weak mitogenic stimulation. | 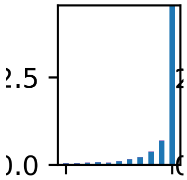 | 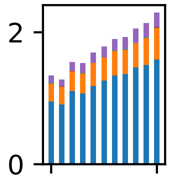 | |

| | | | | | | |
|---|---|---|---|---|---|---|
| ↘ | ***Cyclin D1*** | *95% GF$_H$* | ***Cyclin D1*** inhibition severely limits proliferation even under strong mitogenic stimulation. | 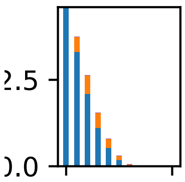 | 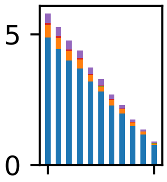 | *Cyclin D1* knockdown inhibits proliferation in a variety of cancer cells [18-19]. |
| ↗ | | *20% GF$_H$* | ***Cyclin D1*** promotes cell cycle entry and proliferation even under weak mitogenic stimulation. | 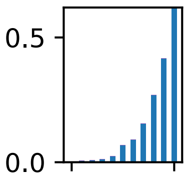 | 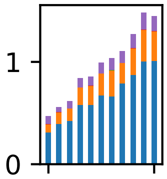 | Overexpression of *cyclin D1* is linked to tumorigenesis of many types of cancer [19]. |
| ↘ | ***E2F1*** | *80% GF$_H$* | ***E2F1*** inhibition drastically limits proliferation even in the presence of strong mitogenic stimulation. | 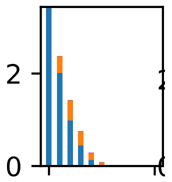 | 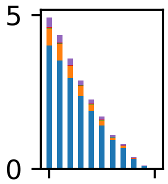 | The absence of all activating E2F proteins causes cell cycle arrest in flies or mammalian fibroblasts [20]. Reduced *E2F1* expression impaired rat glioma cell proliferation [21]. E2F-1 expression can sensitize cells to apoptosis by a number of agents [22]. |
| ↘ | | *65% Trail* | ***E2F1*** inhibition drastically limits Trail-mediated apoptosis in cyclin cells. | 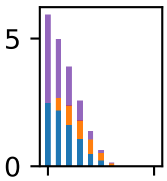 | 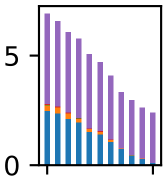 | |
| ↗ | | *20% Trail* | ***E2F1*** over expression strongly increases Trail-mediated apoptosis in *quiescent* cells. | 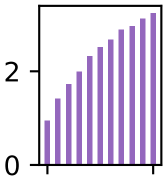 | 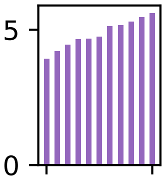 | |
| ↘ | | *20% GF (no GF$_H$)* | ***E2F1*** inhibition blocks apoptosis due to the withdrawal of survival signals. | 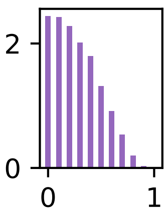 | 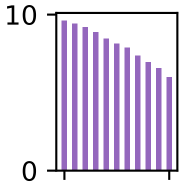 | **prediction** |
| ↘ | ***CyclinE*** | *65% GF$_H$* | ***Cyclin E*** inhibition drastically limits proliferation, and mild inhibition leads to an enrichment of endo-reduplicating (multiple S-phases with no intervening M-phase) cells. | 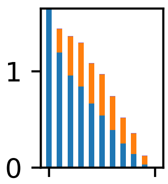 | 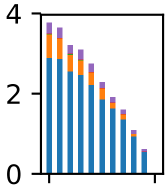 | **prediction** Circumstantial evidence: *forced Cyclin E* expression in fruit fly embryos that normally undergo endoreduplication resulted in strong inhibition of endoreduplication [23]. |

| | | | | | | |
|---|---|---|---|---|---|---|
| ↘ | ***Cdc6*** | *95% $GF_H$* | ***Cdc6*** inhibition abolishes proliferation even under near-saturating mitogenic stimulation. | 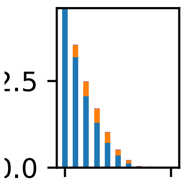  | 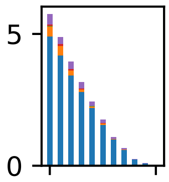  | *Cdc6* is required for initiation of DNA replication in mammalian cells [24]. |
| **Phase Switch & Cell cycle process nodes** | | | | | | |
| ↘ | ***Emi1*** | *95% $GF_H$* | ***Emi1*** inhibition limits normal proliferation and induces endo-reduplication (multiple S-phases with no intervening M-phase). *In the asynchronous model, Emi1 loss leads to aneuploidy; potentially and artifact of this update.* | 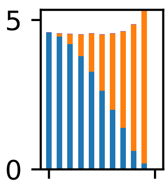  | 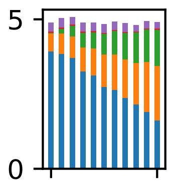  | *Emi1* knockdown with RNAi caused re-replication in HeLa cells, due to premature activation of APC/C that results in destabilization of *cyclin A* [25]. |
| ↘ | ***FoxM1*** | *95% $GF_H$* | ***FoxM1*** inhibition drastically limits normal proliferation and potently induces endo-reduplication, as well as failure to undergo cytokinesis. | 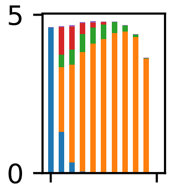  | 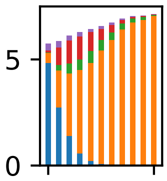  | FoxM1-deficient cells arrest in G2, but also show mitotic abnormalities such us including mitotic spindle aberrations, chromosome missegregation and overt polyploidization [26]. DNA content of fetal cells from *FoxM1* knockout mice show 6 to 50-fold polyploidy [27]. |
| ↘ | ***Cdc25A*** | *95% $GF_H$* | According to our *asynchronous* model, ***Cdc25A*** inhibition limits S-phase entry and proliferation. *The synchronous results are likely an artifact.* | 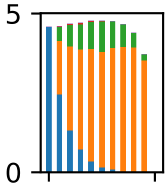  | 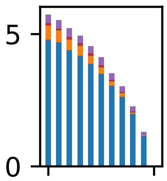  | Inhibition of Cdc25A potently blocks cell cycle progression in G1 or G2 [28]. |
| ↘ | ***CyclinA*** | *80% $GF_H$* | ***Cyclin A*** inhibition drastically limits normal proliferation and potently induces endo-reduplication (multiple S-phases with no intervening M-phase). | 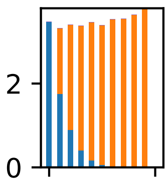  | 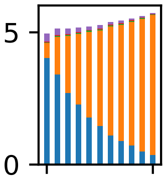  | Cyclin A is required for mitotic entry in primary human fibroblasts [29], and its depletion causes re-replication in HeLa cells [25]. |

| | | | | | | |
|---|---|---|---|---|---|---|
| ↗ | ***Cdh1*** | *80% $GF_H$* | ***Cdh1*** inhibition drastically limits the rate of proliferation. | 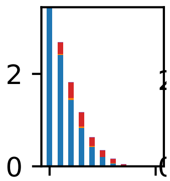 | 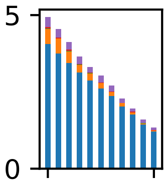 | Inhibition of *Cdh1* binding to APC/C led to significantly prolonged mitosis maintained by a functioning SAC, with no without any spindle defects [30]. |
| ↘ | ***CyclinB*** | *65% $GF_H$* | Interestingly, our two modeling frameworks do not agree about the effects of *CyclinB* depletion.<br>**Synchronous model: *CyclinB*** knockdown results in severe mitotic defects, where cells start anaphase without passing the SAC — a results supported by evidence.<br>**Asynchronous model: *CyclinB*** knockdown predominantly leads to DNA re-duplication from G2 — also supported. | 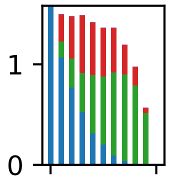 | 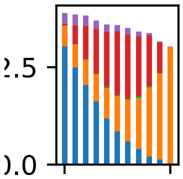 | Depletion of cyclin B1/B2 induces a strong increase of G2 cells and increase in a cycling population with double DNA content. In addition, strong mitotic chromosomal aberration such as mal-oriented chromosomes were observed at metaphase, and cells entered anaphase without reaching the SAC [31]. |
| ↘ | ***Cdc25C*** | *95% $GF_H$* | ***Cdc25C*** inhibition limits normal proliferation and (potently) induces aberrant mitotic progression, leading to aneuploidy. In addition, it enriches for cells that do not complete cytokinesis. | 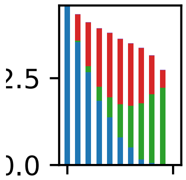 | 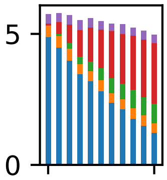 | **chromosome mis-segregation:** Excess Cdc25C M phase degradation in a mouse model that impacts Cdk1 activity lead accelerated mitotic progression, failure to arrest at SAC and improper chromosome segregation [32].<br>**failure of cytokinesis: prediction** |
| ↘ | ***Mad2*** | *80% $GF_H$* | ***Mad2*** inhibition in cells that are not exposed to microtubule damage has no effect on cell cycle progression. This result is and artifact of the fact that we do not model the “trial-and-error” nature of chromosome attachments to the spindle, and thus the mechanism that monitors this has little effect on the model. | 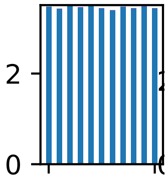 | 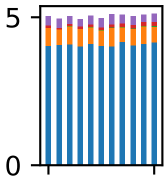 | Mad2 or BubR1 depletion leads to acceleration of mitosis in all cells whereas the depletion of Mad1, Bub1, or Bub3 leaves timing unaffected. The same is also true if the recruitment of Mad2 or BubR1 to kinetochores is blocked by RNAi [33]. |

## Apoptotic Switch

| | | | | | | |
|---|---|---|---|---|---|---|
| ↘ | ***DR4_5*** | *95% Trail ($GF_H$ ON)* | Partial knockdown of the death receptors **DR4** and **DR5** decrease the fraction of cycling and quiescent cells that undergo *Trail*-mediated apoptosis. | 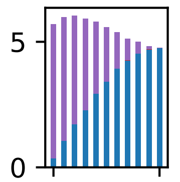 | 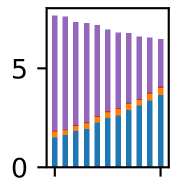 | An adenovirus shown t intrude internalization of DR4 and DR5 from the cell surface blocs Trail-induced apoptosis [34]. |
| | | *95% Trail ($GF_H$ OFF)* | | 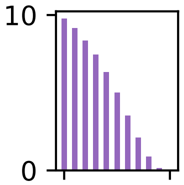 | 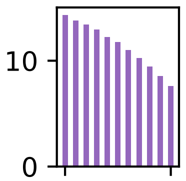 | |
| ↘ | ***Casp8*** | *95% Trail ($GF_H$ OFF)* | **Caspase 8** knockdown decreases the fraction of cycling and quiescent cells that undergo *Trail*-mediated apoptosis. | 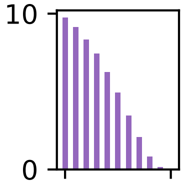 | 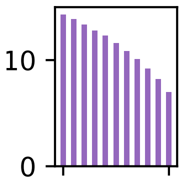 | Knock down or deletion of Caspase-8 suppressed Trail-induced apoptosis [35] |
| ↗ | ***BCL2*** | *95% Trail ($GF_H$ OFF)* | **BCL2** over-expression decreases the fraction of quiescent cells that undergo *Trail*-mediated apoptosis even under near-saturating *Trail* exposure. | 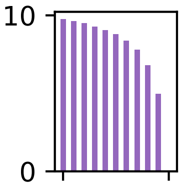 | 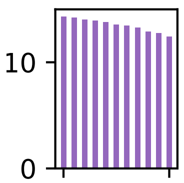 | Overexpression of Bcl-2 or Bcl-XL has been shown to inhibit TRAIL-induced apoptosis in multiple studies [36]. |
| ↗ | ***BCL-XL*** | *20% GF (no $GF_H$)* | **BCL-XL** over-expression decreases the fraction of quiescent cells that undergo growth factor withdrawal mediated apoptosis. | 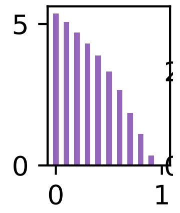 | 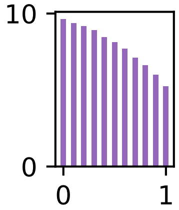 | |
| ↗ | ***Casp2*** | *65% GF (no $GF_H$)* | ***Caspase 2** hyper-activaiton* potentiates apoptosis in cells exposed to weakened survival signals. | 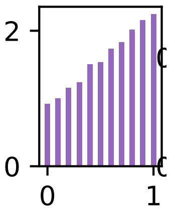 | 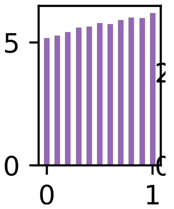 | Over-expression of the active, truncated form of Caspase2 triggers apoptosis [37]. |

| | | | | | | |
|---|---|---|---|---|---|---|
| ↘ | ***BAD*** | *20% GF (no $GF_H$)* | ***BAD*** inhibition blocks apoptosis due to the withdrawal of survival signals. | 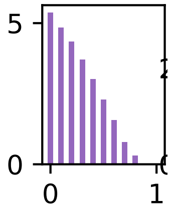 | 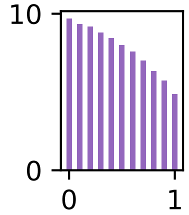 | Reduction of BAD expression by RNAi prevents apoptosis in response to the inhibition of survival pathways [38]. |
| ↘ | ***tBID*** | *95% Trail ($GF_H$ OFF)* | ***BID*** inhibition blocks *Trail*-induced apoptosis in quiescent cells. | 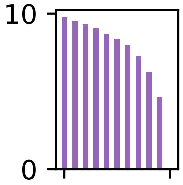 | 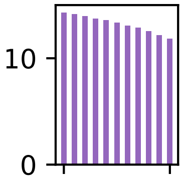 | Depletion / over-expression of Bid in human ovarian carcinoma cells decreased / increased TRAIL-mediated apoptosis [39]. |
| ↘ | ***BAK*** | *20% Trail ($GF_H$ OFF)* | ***BAK*** inhibition weakens apoptosis in response to *Trail* in quiescent and cycling cells. | 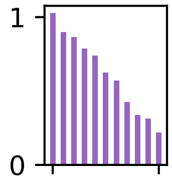 | 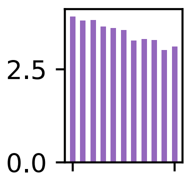 | BAK-null cells show deficient Smac/ DIABLO release from mitochondria in response to Trail, and show decreased rates of apoptosis [40]. |
| ↘ | ***BAX*** | *95% Trail ($GF_H$ OFF)* | ***BAX*** inhibition does not alter apoptosis in response to *Trail*. *This model behavior is not supported by experiments, and it is a likely artifact of the fact that in the model BAK alone can trigger MOMP.* | 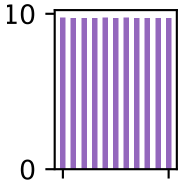 | 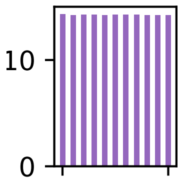 | BAX-null cells also show deficient Smac/ DIABLO release from mitochondria in response to *Trail*, and show decreased rates of apoptosis (more potent than BAK) [40]. |
| ↘ | ***IAPs*** | *95% Trail ($GF_H$ OFF)* | Inhibition of IAP proteins increases the rate of apoptosis in response to Trail (quiescent and cycling cells) as well as in response to the withdrawal of survival signaling. | 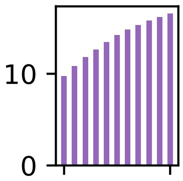 | 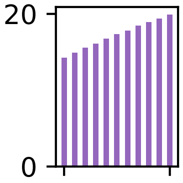 | Down-regulation of all IAPs simultaneously induces apoptosis in multiple melanoma cell lines, and sensitized them to both FASL and Trail [41]. |
| | | *50% GF ($GF_H$ OFF)* | | 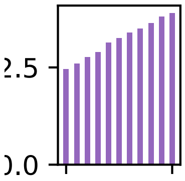 | 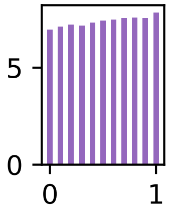 | |


## References

1. Schubbert S, Shannon K, Bollag G. Hyperactive Ras in developmental disorders and cancer. Nat Rev Cancer. Nature Publishing Group; 2007;7: 295–308. doi:10.1038/nrc2109
2. Liu F, Yang X, Geng M, Huang M. Targeting ERK, an Achilles' Heel of the MAPK pathway, in cancer therapy. Acta Pharm Sin B. 2018;8: 552–562. doi:10.1016/j.apsb.2018.01.008

3. Bian Y-H, Xu J, Zhao W-Y, Zhang Z-Z, Tu L, Cao H, et al. Targeting mTORC2 component rictor inhibits cell proliferation and promotes apoptosis in gastric cancer. Am J Transl Res. e-Century Publishing Corporation; 2017;9: 4317–4330.
4. Benavides-Serrato A, Lee J, Holmes B, Landon KA, Bashir T, Jung ME, et al. Specific blockade of Rictor-mTOR association inhibits mTORC2 activity and is cytotoxic in glioblastoma. PLoS ONE. 2017;12: e0176599. doi:10.1371/journal.pone.0176599
5. Lu Z, Cox-Hipkin MA, Windsor WT, Boyapati A. 3-phosphoinositide-dependent protein kinase-1 regulates proliferation and survival of cancer cells with an activated mitogen-activated protein kinase pathway. Molecular Cancer Research. American Association for Cancer Research; 2010;8: 421–432. doi:10.1158/1541-7786.MCR-09-0179
6. Medema RH, Kops GJ, Bos JL, Burgering BM. AFX-like Forkhead transcription factors mediate cell-cycle regulation by Ras and PKB through p27kip1. Nature. 2000;404: 782–787. doi:10.1038/35008115
7. Kawaguchi S-I, Harada K, Supriatno, Yoshida H, Sato M. Overexpression of tuberous sclerosis complex 2 exerts antitumor effect on oral cancer cell lines. Oral Oncol. 2003;39: 836–841.
8. Khare L, Astrinidis A, Senapedis W, Adams PD, Henske EP. Expression of wild type and mutant TSC2, but not TSC1, causes an increase in the G1 fraction of the cell cycle in HEK293 cells. J Med Genet. BMJ Publishing Group; 2002;39: 676–680. doi:10.1136/jmg.39.9.676
9. Patel PH, Thapar N, Guo L, Martinez M, Maris J, Gau C-L, et al. Drosophila Rheb GTPase is required for cell cycle progression and cell growth. J Cell Sci. 2003;116: 3601–3610. doi:10.1242/jcs.00661
10. Dowling RJO, Topisirovic I, Alain T, Bidinosti M, Fonseca BD, Petroulakis E, et al. mTORC1-Mediated Cell Proliferation, But Not Cell Growth, Controlled by the 4E-BPs. Science. 2010;328: 1172–1176. doi:10.1126/science.1187532
11. Sridharan S, Basu A. S6 kinase 2 promotes breast cancer cell survival via Akt. Cancer Research. American Association for Cancer Research; 2011;71: 2590–2599. doi:10.1158/0008-5472.CAN-10-3253
12. Tseng A-S, Engel FB, Keating MT. The GSK-3 inhibitor BIO promotes proliferation in mammalian cardiomyocytes. Chem Biol. 2006;13: 957–963. doi:10.1016/j.chembiol.2006.08.004
13. Spencer SL, Cappell SD, Tsai F-C, Overton KW, Wang CL, Meyer T. The proliferation-quiescence decision is controlled by a bifurcation in CDK2 activity at mitotic exit. Cell. 2013;155: 369–383. doi:10.1016/j.cell.2013.08.062
14. Karimian A, Ahmadi Y, Yousefi B. Multiple functions of p21 in cell cycle, apoptosis and transcriptional regulation after DNA damage. DNA Repair (Amst). 2016;42: 63–71. doi:10.1016/j.dnarep.2016.04.008
15. Burkhart DL, Sage J. Cellular mechanisms of tumour suppression by the retinoblastoma gene. Nat Rev Cancer. Nature Publishing Group; 2008;8: 671–682. doi:10.1038/nrc2399
16. Yang L, Si X, Wang W. Overexpression of bcl-2 protects hepatoma cell line HCC-9204 from ethanol-induced apoptosis. Chin Med J. 2002;115: 8–11.

17. McKeown MR, Bradner JE. Therapeutic strategies to inhibit MYC. Cold Spring Harb Perspect Med. Cold Spring Harbor Laboratory Press; 2014;4: a014266–a014266. doi: 10.1101/cshperspect.a014266
18. Wang J, Wang Q, Cui Y, Liu ZY, Zhao W, Wang CL, et al. Knockdown of cyclin D1 inhibits proliferation, induces apoptosis, and attenuates the invasive capacity of human glioblastoma cells. Journal of neuro-oncology. Springer US; 2012;106: 473–484. doi: 10.1007/s11060-011-0692-4
19. Shan J, Zhao W, Gu W. Suppression of cancer cell growth by promoting cyclin D1 degradation. Mol Cell. 2009;36: 469–476. doi:10.1016/j.molcel.2009.10.018
20. Chen D, Pacal M, Wenzel P, Knoepfler PS, Leone G, Bremner R. Division and apoptosis of E2f-deficient retinal progenitors. Nature. Nature Publishing Group; 2009;462: 925–929. doi:10.1038/nature08544
21. Reis Vasques Dos L, Pujiz RS, Strauss BE, Krieger JE. Knockdown of E2f1 by RNA interference impairs proliferation of rat cells in vitro. Genet Mol Biol. Sociedade Brasileira de Genética; 2010;33: 17–22. doi:10.1590/S1415-47572009005000104
22. Phillips AC, Ernst MK, Bates S, Rice NR, Vousden KH. E2F-1 potentiates cell death by blocking antiapoptotic signaling pathways. Mol Cell. 1999;4: 771–781.
23. Weiss A, Herzig A, Jacobs H, Lehner CF. Continuous Cyclin E expression inhibits progression through endoreduplication cycles in Drosophila. Curr Biol. 1998;8: 239–242.
24. Yan Z, DeGregori J, Shohet R, Leone G, Stillman B, Nevins JR, et al. Cdc6 is regulated by E2F and is essential for DNA replication in mammalian cells. Proc Natl Acad Sci U S A. National Academy of Sciences; 1998;95: 3603–3608.
25. Machida YJ, Dutta A. The APC/C inhibitor, Emi1, is essential for prevention of rereplication. Genes & Development. Cold Spring Harbor Lab; 2007;21: 184–194. doi: 10.1101/gad.1495007
26. Laoukili J, Stahl M, Medema RH. FoxM1: at the crossroads of ageing and cancer. Biochim Biophys Acta. 2007;1775: 92–102. doi:10.1016/j.bbcan.2006.08.006
27. Korver W, Schilham MW, Moerer P, van den Hoff MJ, Dam K, Lamers WH, et al. Uncoupling of S phase and mitosis in cardiomyocytes and hepatocytes lacking the winged-helix transcription factor Trident. Curr Biol. 1998;8: 1327–1330.
28. Donzelli M, Draetta GF. Regulating mammalian checkpoints through Cdc25 inactivation. EMBO reports. 2003;4: 671–677. doi:10.1038/sj.embor.embor887
29. Mitra J, Enders GH. Cyclin A/Cdk2 complexes regulate activation of Cdk1 and Cdc25 phosphatases in human cells. Oncogene. Nature Publishing Group; 2004;23: 3361–3367. doi:10.1038/sj.onc.1207446
30. Zeng X, Sigoillot F, Gaur S, Choi S, Pfaff KL, Oh D-C, et al. Pharmacologic inhibition of the anaphase-promoting complex induces a spindle checkpoint-dependent mitotic arrest in the absence of spindle damage. Cancer Cell. 2010;18: 382–395. doi:10.1016/j.ccr.2010.08.010
31. Soni DV, Sramkoski RM, Lam M, Stefan T, Jacobberger JW. Cyclin B1 is rate limiting but not essential for mitotic entry and progression in mammalian somatic cells. Cell Cycle. Taylor & Francis; 2008;7: 1285–1300. doi:10.4161/cc.7.9.5711

32. Vecchione A, Baldassarre G, Ishii H, Nicoloso MS, Belletti B, Petrocca F, et al. Fez1/Lzts1 absence impairs Cdk1/Cdc25C interaction during mitosis and predisposes mice to cancer development. Cancer Cell. 2007;11: 275–289. doi:10.1016/j.ccr.2007.01.014
33. Meraldi P, Draviam VM, Sorger PK. Timing and checkpoints in the regulation of mitotic progression. Dev Cell. 2004;7: 45–60. doi:10.1016/j.devcel.2004.06.006
34. Tollefson AE, Toth K, Doronin K, Kuppuswamy M, Doronina OA, Lichtenstein DL, et al. Inhibition of TRAIL-induced apoptosis and forced internalization of TRAIL receptor 1 by adenovirus proteins. J Virol. 2001;75: 8875–8887. doi:10.1128/JVI.75.19.8875-8887.2001
35. Henry CM, Martin SJ. Caspase-8 Acts in a Non-enzymatic Role as a Scaffold for Assembly of a Pro-inflammatory “FADDosome” Complex upon TRAIL Stimulation. Mol Cell. 2017;65: 715–729.e5. doi:10.1016/j.molcel.2017.01.022
36. Sprick MR, Walczak H. The interplay between the Bcl-2 family and death receptor-mediated apoptosis. Biochim Biophys Acta. 2004;1644: 125–132. doi:10.1016/j.bbamcr.2003.11.002
37. Wang L, Miura M, Bergeron L, Zhu H, Yuan J. Ich-1, an Ice/ced-3-related gene, encodes both positive and negative regulators of programmed cell death. Cell. 1994;78: 739–750.
38. She Q-B, Solit DB, Ye Q, O'Reilly KE, Lobo J, Rosen N. The BAD protein integrates survival signaling by EGFR/MAPK and PI3K/Akt kinase pathways in PTEN-deficient tumor cells. Cancer Cell. 2005;8: 287–297. doi:10.1016/j.ccr.2005.09.006
39. Goncharenko-Khaider N, Lane D, Matte I, Rancourt C, Piché A. The inhibition of Bid expression by Akt leads to resistance to TRAIL-induced apoptosis in ovarian cancer cells. Oncogene. Nature Publishing Group; 2010;29: 5523–5536. doi:10.1038/onc.2010.288
40. Kandasamy K, Srinivasula SM, Alnemri ES, Thompson CB, Korsmeyer SJ, Bryant JL, et al. Involvement of proapoptotic molecules Bax and Bak in tumor necrosis factor-related apoptosis-inducing ligand (TRAIL)-induced mitochondrial disruption and apoptosis: differential regulation of cytochrome c and Smac/DIABLO release. Cancer Res. 2003;63: 1712–1721.
41. Ramakrishnan V, Painuly U, Kimlinger T, Haug J, Rajkumar SV, Kumar S. Inhibitor of apoptosis proteins as therapeutic targets in multiple myeloma. Leukemia. Nature Publishing Group; 2014;28: 1519–1528. doi:10.1038/leu.2014.2
