# Supplementary material for: Boolean model of growth signaling, cell cycle and apoptosis predicts the molecular mechanism of aberrant cell cycle progression driven by hyperactive PI3K
Source: PLoS Comput Biol. 2019 Mar 15;15(3):e1006402. doi: 10.1371/journal.pcbi.1006402 (PMC6436762; doi:10.1371/journal.pcbi.1006402)
Supplement: S2 File — Jupyter Notebook in Python that uses the BooleanNet software package to simulate synchronous and asynchronous versions of our model and reproduce our key results (“SI_notebook.ipynb”; available at https://github.com/deriteidavid/cell_cycle_apoptosis_Sizek_etal_PloSCompBio_2019). (ZIP) [file pcbi.1006402.s020.zip › SI_notebook.html]

SI\_notebook


# Boolean model of growth signaling, cell cycle and apoptosis predicts the molecular mechanism of aberrant cell cycle progression driven by hyperactive PI3K¶

## Supporting Notebook¶

In [1]:

```
import boolean2
import numpy as np
import matplotlib.pyplot as plt
%matplotlib inline
import seaborn as sns
```

### Support funtions:¶

In [2]:

```
def plot_state_succession(states,state_labels=None,title=None, nodes=None, fontsizex=10):

    '''
    Creates a plot of the trajectory performed by a BooleanNet model.
    
    Inputs:
    states - container of boolean2 State objects or of dictionaries equivalent to those.
    state_labels (optional) - container of strings corresponding to every state. They will be the labels on the Y axis. Default: range(len(states))
    title (optional) - the title of the plot
    nodes (optional) - the set of nodes to be whose trajectory should be plotted. The set can be a subset or identical set to the nodes in the states. If list the order of the list will be respected on the plot.
    fontsizex (optional) - the fontsize of the X axis labels (the node names). Default: 10
    
    '''
    from matplotlib import pyplot as plt
    import matplotlib
    if nodes==None:
        nodes=states[0].keys()
    state_transition=[]
    for s in states:
        state_transition.append([s[k] for k in nodes])
    cmap = matplotlib.colors.ListedColormap(['blue', 'orange'])
    steps=len(state_transition)
    plt.figure(figsize=(len(nodes),steps))
    plt.imshow(state_transition, interpolation='none',cmap=cmap)
    ax = plt.gca()
    plt.xticks(range(len(nodes)),nodes, fontsize=fontsizex)   
    if state_labels==None:
        plt.yticks(range(steps),fontsize=12)
    else:
        plt.yticks(range(steps),state_labels,fontsize=12)
    if title!=None:
        plt.title(title)
    ax.set_yticks(np.arange(-.5, steps,1),minor=True)
    plt.grid(which='minor', color='black', linestyle='-', linewidth=2)
    plt.show()

import random
def general_async_pick( lines ):
    data = [ random.choice( lines )]
    #print data
    return data   

class TransGraph(object):
    """
    Represents a transition graph
    """
    import networkx as nx
    def __init__(self, logfile, verbose=False):
        self.graph = nx.MultiDiGraph(  )         
        self.fp = open( logfile, 'wt')
        self.verbose = verbose
        self.seen = set()
        self.store = dict()
        self.colors = dict()

    def add(self, states, times=None):
        "Adds states to the transition"
    
        # generating the fingerprints and sto
        times = times or range(len(states))
        fprints = []
        for state in states:
            if self.verbose:
                fp = state.bin()
            else:
                fp = state.fp()
            fprints.append( fp )
            self.store[fp] = state

        self.fp.write( '*** transitions from %s ***\n' % fprints[0] )

        for head, tail, tstamp in zip(fprints, fprints[1:], times ):
            pair = (head, tail)
            self.fp.write('T=%s: %s->%s\n' %  (tstamp, head, tail) ) 
            if pair not in self.seen:
                self.graph.add_edge(head, tail)
                self.seen.add(pair)
        
    def save(self, fname, colormap={}):
        "Saves the graph as gml"
        write_gml(graph=self.graph, fname=fname, colormap=colormap)
    
        self.fp.write( '*** node values ***\n' )

        # writes the mapping
        first = self.store.values()[0]
        header = [ 'state' ] + first.keys()
        self.fp.write( util.join(header) )
        
        for fprint, state in sorted( self.store.items() ):
            line = [ fprint ]  + map(int, state.values() )
            self.fp.write( util.join(line) )
            
def read_rules_text(model_name):
    """
    A simple file reading function, that reads the BooleanNet rules text files and returns them as a string
    
    Input: 
    model_name - str, containing the name of the model, assuming the convention, that the model files are name are named "model_name".txt
    
    Returns:
    rules - str, the boolean rules contained in the file
    """
    rules_file='%s.txt'%model_name
    with open(rules_file,'r') as f:
        rules=f.read()
    rules=rules.replace('#BOOLEAN RULES','') #in some cases this line is in the file, but we don't need it
    return rules

def merge_two_dicts(x, y):
    z = x.copy()   # start with x's keys and values
    z.update(y)    # modifies z with y's keys and values & returns None
    return z

def default_shuffler( lines ):
    "Default shuffler"
    temp = lines[:]
    random.shuffle( lines )
    return temp
```

## PI3K model¶

The Boolean rules for the model can be retrieved two ways: by directly defining them as a python string, or by reading them in from a file (both shown below)

In [3]:

```
model_name='PI3K'
```

In [4]:

```
rules = '''p110_H *= (p110_H and not NeddL4) or (p110_H and FoxO3) or (not NeddL4 and FoxO3)
FoxO3 *= not AKT_H
PI3K_H *= p110_H
NeddL4 *= IP3 and Ca2
IP3 *= PLCgamma
PLCgamma *= p110_H and PI3K_H
Ca2 *= IP3
AKT_H *= p110_H and PI3K_H'''
```

In [5]:

```
#alternatively:
rules=read_rules_text(model_name)
```

We need an initial state to start simulating the model. Any initial state can be chosen.

In [6]:

```
initial_state={'p110_H':0,
'FoxO3':1,
'PI3K_H':1,
'NeddL4':0,
'IP3':0,
'PLCgamma':1,
'Ca2':0,
'AKT_H':0}
```

Next we instantiate the synchronous model within the BooleanNet framework, and initialize it to the initial state selected above.

In [7]:

```
sync_model = boolean2.Model(rules, mode='sync')
sync_model.initialize(lambda node: initial_state[node])
```

Iterating the model from the initial state long enough will make it converge into the cyclic attractor, which we expect to be of length 10.

In [8]:

```
steps = 50
sync_model.iterate(steps)
```

In [9]:

```
_,cycle_length=sync_model.detect_cycles() #.detect_cycles returns the starting point and the length of a cycle
assert cycle_length == 10
cycle_states = sync_model.states[-cycle_length:]
```

We show the cyclic attractors trajectory. The indices on the Y axis will be used to refer to the individual states in the cycle later

In [10]:

```
plot_state_succession(cycle_states)
```

## Studying the PI3K model with general asynchronous update¶

We expect the model to be roboust enough, such that the cyclic behavior will re-emerge with general asyncronous update, as a complex attractor.

The model is instatiated below, together with a plot of the trajectory, to aid the validation for the asynchronous update scheme.

In [11]:

```
async_model = boolean2.Model(rules, mode='async')
async_model.initialize(lambda node: initial_state[node])
```

In [12]:

```
steps = 20
async_model.iterate(steps, shuffler=general_async_pick)
plot_state_succession(async_model.states)
```

## Creating an STG based on an extended random walk¶

To see if a complex attractor emerges we sample the STG with a random walk, expecting two things: first the number of unique states visited is much less than the number of steps taken, meaning the random walker is in a complex attractor, second, a set of states in the complex attractor should be very similar (or identical) to the synchronous attractor states, preferably respecting the synchronous order too.

In [13]:

```
nr_of_steps=10000
```

In [14]:

```
import networkx as nx
from itertools import combinations, product
from collections import Counter
start_state=cycle_states[0]
async_model.initialize(lambda node: start_state[node])
async_model.iterate(nr_of_steps,shuffler=general_async_pick)

TG=TransGraph(logfile='states.txt')
TG.add(async_model.states)
G=TG.graph
print "Number of unique states visited:",G.number_of_nodes()
print "Number of unique transitions:",G.number_of_edges()
```

```
Number of unique states visited: 59
Number of unique transitions: 173
```

Next, we visualize the resulting STG, by plotting the network, with a few visual aides:

- Node size: proportional to the number of times the random walk visited the state
- Node label: the closest synchronous state (see the label-state mapping above)
- Node color: overlap with the closest synchronous state
- Edge width: proportional to the number of times the unique transition has been made
- Layout: Kamada-Kawai algorithm

In [15]:

```
## Calculating some of the metrics discussed above
fp_state_dict=dict(zip(async_model.fp(),async_model.states))
overlap_to_sync_cycle={}
closest_sync_state={}
for n in G.nodes:
    async_state=fp_state_dict[n]
    overlaps=[]
    for j in range(len(cycle_states)):
        
        #async_state=dict(zip(model_async.nodes,top_PR_states[i]))
        sync_state=cycle_states[j]
        overlap=0
        for s in async_state.keys():
            if async_state[s] == sync_state[s]:
                overlap+=1.
        overlap/=len(async_state.keys())
        overlaps.append(overlap)
        
    overlap_to_sync_cycle[n]=max(overlaps)
    closest_sync_state[n]=overlaps.index(max(overlaps))

fp_states=async_model.fp()
state_occurances=Counter(fp_states)
edge_occurances=Counter([(fp_states[i-1], fp_states[i]) for i in range(len(fp_states))])
```

Ploting the resulting STG

In [16]:

```
# drawing nodes and edges separately so we can capture collection for colobar
plt.figure(figsize=(20,20))
pos = nx.kamada_kawai_layout(G)
ec = nx.draw_networkx_edges(G, pos, alpha=0.2,with_labels=False,width=[float(edge_occurances[i])*0.1 for i in G.edges()])
lc = lc = nx.draw_networkx_labels(G,pos,{i:closest_sync_state[i] for i in G.nodes()}, font_size=16)
nc = nx.draw_networkx_nodes(G, pos, node_color=[overlap_to_sync_cycle[i] for i in G.nodes()], 
                                with_labels=[closest_sync_state[i] for i in G.nodes()], node_size=[float(state_occurances[j]) for j in G.nodes()], cmap=plt.cm.RdPu, alpha=0.7)
plt.colorbar(nc)
plt.axis('off')
plt.show()
```

## "Carpet" pattern for visualizing the cyclic behavior in the general asynchronous trajectory¶

In [17]:

```
selected_states=async_model.states[:500]
hamming_dist_top_async_sync=np.zeros((len(selected_states),len(cycle_states)))
for i in range(len(selected_states)):
    for j in range(len(cycle_states)):
        
        #async_state=dict(zip(model_async.nodes,top_PR_states[i]))
        async_state=selected_states[i]
        sync_state=cycle_states[j]
        
        for s in async_state.keys():
            if async_state[s] == sync_state[s]:
                hamming_dist_top_async_sync[i,j]+=1.
                
hamming_dist_top_async_sync/=len(async_model.nodes)
plt.figure(figsize=(10,50))
plt.imshow(hamming_dist_top_async_sync, cmap=plt.cm.RdPu, aspect='auto')
plt.xlabel('Syncronous Attractor States',fontsize=20)
plt.ylabel('Async State Transition',fontsize=20)
clb=plt.colorbar()
clb.set_label('Normalized overlap',fontsize=20)
```

# Finding the static attractors of the modules¶

Bellow we specify a method of full exploration of the state space of the synchronous models. The modules making up the full model are small enough so this could be done. The largest one is the Apoptosis module, however given the BoolNet rules of the rest of the networks the same script is applicable. One just has to substitute the name of the desired module in the model\_name variable, and rerunning the cells belonging to this secion shall find and plot the attractors.  
The modules provided in separate files:

- Apoptotic\_Switch
- Origin\_Licensing\_Switch
- Restriction\_Switch
- Phase\_Switch

### Mapping the full STG¶

In [18]:

```
model_name='Origin_Licensing_Switch' # use here any of the module names stated above
```

In [19]:

```
rules=read_rules_text(model_name)
```

In [20]:

```
################################################################################################
#SYNC STG
################################################################################################
def get_sync_STG(model_sync):
    '''
    Given a syncronous model the function initiates all 2**N initial states, where N is the number of nodes, and performs one step, adding it to a state transition graph (STG).
    WARNING! Due to the exponential growth of the state space, with the size of the network, this can be extremely demanding for larger networks

    Inputs: model_sync - boolean2 type network model initiated as synchronous 

    Returns: g - networx graph object
    '''

    fp_state_dict={}
    tg=TransGraph(logfile='sync_states.txt')
    for init_state, some_func in boolean2.state.all_initial_states(model_sync.nodes): 

        model_sync.initialize(lambda node: init_state[node])
        model_sync.iterate(1)
        tg.add(model_sync.states)
        fp_state_dict=merge_two_dicts(fp_state_dict,dict(zip(model_sync.fp(),model_sync.states)))
    g=tg.graph
    g.remove_edges_from(g.selfloop_edges()) 
    return g,fp_state_dict
```

In [21]:

```
sync_model = boolean2.Model(rules, mode='sync')
print 'State space size:',2**len(sync_model.nodes)
```

```
State space size: 16
```

In [22]:

```
g,fp_state_dict=get_sync_STG(sync_model)
```

In [23]:

```
g.remove_edges_from(g.selfloop_edges()) 
static_attractors=[]
for i in g.nodes():
    if g.out_degree(i) == 0:
        static_attractors.append(fp_state_dict[i])
```

In [24]:

```
plot_state_succession(static_attractors, state_labels=['Attractor_%d'%i for i in range(len(static_attractors))])
```

# 3. The network of linked regulatory models reproduces environment-dependent proliferation (cell cycle), quiescence and/or apoptosis¶

In [25]:

```
model_name='full_cell_cycle'
rules=read_rules_text(model_name)
```

We initialize the model in a state that is in the basin of the cyclic attractor.

In [26]:

```
init_array=np.loadtxt('cell_cycle_init.txt',dtype=str)
initial_state=dict(zip(init_array[:,0],init_array[:,1].astype(bool)))
print initial_state
```

```
{'PLCgamma': False, 'Cyto_C': False, 'Ca2': False, 'IP3': False, 'CHK1': False, 'A_Kinetochores': False, 'CyclinB': False, 'CyclinA': False, 'CyclinE': False, 'p110_H': False, 'p27Kip1': False, 'Cdc25C': True, 'Cdc25B': True, 'Cdc25A': True, 'PI3K_H': False, 'Cdh1': True, 'CyclinD1': True, 'NeddL4': False, 'f4N_DNA': True, 'pAPC': True, 'Ras': True, 'AKT_B': True, 'GF_High': True, 'Myc': True, 'AKT_H': False, 'UbcH10': True, 'p21': False, 'Pre_RC': False, 'Rheb': False, 'Cdc6': False, 'S6K': True, 'Cdt1': False, 'RTK': True, 'p21_mRNA': False, 'BCLXL': True, 'GSK3': False, 'RAF': True, 'DR4_5': False, 'SMAC': False, 'IAPs': True, 'Cdk1': False, 'mTORC2': True, 'mTORC1': True, 'ATR': False, 'TSC2': True, 'PRAS40': False, 'Ect2': False, 'Trail': False, 'Replication': False, 'Plk1_H': True, 'PIP3': True, 'PI3K': True, 'FoxM1': False, 'Casp8': False, 'MEK': True, 'Cdc20': True, 'Mad2': False, 'Wee1': False, 'ORC': True, 'Casp2': False, 'Casp3': False, 'MCL_1': True, 'BAX': False, 'Casp9': False, 'BAD': False, 'geminin': False, 'Emi1': True, 'BAK': False, 'CAD': False, 'BIM': False, 'BIK': False, 'BID': False, 'CyclinA_mRNA': True, 'SOS': True, 'Grb2': True, 'eIF4E': True, 'ERK': True, 'U_Kinetochores': False, 'pRB': False, 'Plk1': False, 'FoxO3': False, 'FoxO1': False, 'DAG': False, 'E2F1': True, 'GF': True, 'PDK1': True, 'BCL2': True}
```

In [27]:

```
sync_model = boolean2.Model(rules, mode='sync')
sync_model.initialize(lambda node: initial_state[node])
steps = 100
sync_model.iterate(steps)
_,cycle_length=sync_model.detect_cycles() #.detect_cycles returns the starting point and the length of a cycle
assert cycle_length == 21 #sanity check
```

We need the 21 states of the cyclic attractor states for later assesment of the asyncronous dynamics

In [28]:

```
cycle_states = sync_model.states[-cycle_length:]
```

The node order given below corresponds with the order represented on the figures shown in the main article, which were generated intependently of this code.

In [29]:

```
node_order=["GF_High","GF","RTK","Grb2","SOS","Ras","RAF","MEK","ERK","mTORC2","PI3K","PIP3","PDK1","AKT_B","p110_H","PI3K_H","AKT_H","FoxO3","PLCgamma","IP3","Ca2","NeddL4","FoxO1","p21_mRNA","TSC2","PRAS40","DAG","Rheb","mTORC1","S6K","eIF4E","GSK3","p21","pRB","p27Kip1","Myc","CyclinD1","E2F1","CyclinE","ORC","Cdc6","Cdt1","Pre_RC","geminin","CyclinA_mRNA","Emi1","FoxM1","Cdc25A","CyclinA","Wee1","UbcH10","CyclinB","Cdc25B","Plk1","Cdc25C","Cdk1","pAPC","Cdc20","Cdh1","Replication","ATR","CHK1","f4N_DNA","U_Kinetochores","Mad2","A_Kinetochores","Plk1_H","Ect2","Trail","DR4_5","Casp8","Casp2","MCL_1","BCLXL","BCL2","BAD","BIK","BIM","BID","BAK","BAX","Cyto_C","SMAC","IAPs","Casp9","Casp3","CAD"]
```

In [30]:

```
plot_state_succession(cycle_states,nodes=node_order)
```

### Static attractors of the system¶

Next we validate the 6 static attractors found. We did this by initializing the model in each of them and iterating it a single step. In synchronous models if there's no change in the state of the model, it means it is an attractor. The attractor states are saved in files named in accordance with their indexing in the paper. We also use a sinle random state as control.

- attr\_1 - ...

In [31]:

```
attr_indices=[1,2,3,4,5,7] #just to follow the sync model's naming convention
static_attractors={}
for attr_index in attr_indices:
    attr_n=dict(np.loadtxt('attr_%d.txt'%attr_index,dtype=str)[1:])
    for node,value in attr_n.items():
        attr_n[node]=bool(int(value))
    static_attractors['attr_%d'%attr_index] = attr_n
    
#a randomly generated state for control: 
random_state=attr_n.copy()
for node,value in random_state.items():
    random_state[node]=bool(int(random.randint(0,1)))
static_attractors['random_state'] = random_state
```

We test if the stable attractors of the sync model are correct and if they actually remain stable. We include the control random state that shouldn't remain stable. Here we use an update scheme that updates all the nodes in order

In [32]:

```
for attr_name, attr_n in static_attractors.items():
    print attr_name
    sync_model.initialize(lambda node: attr_n[node])
    steps = 1
    sync_model.iterate(steps)
    if attr_name!='random_state':
        assert sync_model.states[0]==sync_model.states[-1]
    plot_state_succession(sync_model.states,nodes=node_order)

random_state=static_attractors['random_state']
_=static_attractors.pop('random_state')
```

```
attr_2
```

```
attr_3
```

```
attr_1
```

```
attr_7
```

```
attr_4
```

```
attr_5
```

```
random_state
```

# 4. Our model’s dynamics is robust with respect to fluctuations in timing, and reproduces dynamical features of cycling cells with synchronous as well as asynchronous update¶

## Random Order asynchronous update¶

In [33]:

```
model_name='full_cell_cycle'
rules=read_rules_text(model_name)
```

In [34]:

```
init_array=np.loadtxt('cell_cycle_init.txt',dtype=str)
initial_state=dict(zip(init_array[:,0],init_array[:,1].astype(bool)))
print initial_state
```

```
{'PLCgamma': False, 'Cyto_C': False, 'Ca2': False, 'IP3': False, 'CHK1': False, 'A_Kinetochores': False, 'CyclinB': False, 'CyclinA': False, 'CyclinE': False, 'p110_H': False, 'p27Kip1': False, 'Cdc25C': True, 'Cdc25B': True, 'Cdc25A': True, 'PI3K_H': False, 'Cdh1': True, 'CyclinD1': True, 'NeddL4': False, 'f4N_DNA': True, 'pAPC': True, 'Ras': True, 'AKT_B': True, 'GF_High': True, 'Myc': True, 'AKT_H': False, 'UbcH10': True, 'p21': False, 'Pre_RC': False, 'Rheb': False, 'Cdc6': False, 'S6K': True, 'Cdt1': False, 'RTK': True, 'p21_mRNA': False, 'BCLXL': True, 'GSK3': False, 'RAF': True, 'DR4_5': False, 'SMAC': False, 'IAPs': True, 'Cdk1': False, 'mTORC2': True, 'mTORC1': True, 'ATR': False, 'TSC2': True, 'PRAS40': False, 'Ect2': False, 'Trail': False, 'Replication': False, 'Plk1_H': True, 'PIP3': True, 'PI3K': True, 'FoxM1': False, 'Casp8': False, 'MEK': True, 'Cdc20': True, 'Mad2': False, 'Wee1': False, 'ORC': True, 'Casp2': False, 'Casp3': False, 'MCL_1': True, 'BAX': False, 'Casp9': False, 'BAD': False, 'geminin': False, 'Emi1': True, 'BAK': False, 'CAD': False, 'BIM': False, 'BIK': False, 'BID': False, 'CyclinA_mRNA': True, 'SOS': True, 'Grb2': True, 'eIF4E': True, 'ERK': True, 'U_Kinetochores': False, 'pRB': False, 'Plk1': False, 'FoxO3': False, 'FoxO1': False, 'DAG': False, 'E2F1': True, 'GF': True, 'PDK1': True, 'BCL2': True}
```

In [35]:

```
async_model = boolean2.Model(rules, mode='async')
async_model.initialize(lambda node: initial_state[node])
```

### Creating an STG based on an extended random walk¶

To see if a complex attractor emerges we sample the STG with a random walk, expecting two things: first the number of unique states visited is much less than the number of steps taken, meaning the random walker is in a complex attractor, second, a set of states in the complex attractor should be very similar (or identical) to the synchronous attractor states, preferably respecting the synchronous order too.

In [36]:

```
nr_of_steps=1000
```

In [37]:

```
import networkx as nx
from itertools import combinations, product
from collections import Counter

start_state=cycle_states[0]
async_model.initialize(lambda node: start_state[node])
async_model.iterate(nr_of_steps,shuffler=default_shuffler)

TG=TransGraph(logfile='states.txt')
TG.add(async_model.states)
G=TG.graph
print "Number of unique states visited:",G.number_of_nodes()
print "Number of unique transitions:",G.number_of_edges()
```

```
Number of unique states visited: 66
Number of unique transitions: 66
```

In [38]:

```
selected_states=async_model.states[:200]
hamming_dist_top_async_sync=np.zeros((len(selected_states),len(cycle_states)))
for i in range(len(selected_states)):
    for j in range(len(cycle_states)):
        
        #async_state=dict(zip(model_async.nodes,top_PR_states[i]))
        async_state=selected_states[i]
        sync_state=cycle_states[j]
        
        for s in async_state.keys():
            if async_state[s] == sync_state[s]:
                hamming_dist_top_async_sync[i,j]+=1.
                
hamming_dist_top_async_sync/=len(async_model.nodes)
plt.figure(figsize=(20,100))
plt.imshow(hamming_dist_top_async_sync, cmap=plt.cm.RdPu, aspect='auto')
plt.xlabel('Syncronous Attractor States',fontsize=20)
plt.ylabel('Async State Transition',fontsize=20)
clb=plt.colorbar()
clb.set_label('Normalized overlap',fontsize=20)
```

## Sampling the STG¶

Next we create a sample of the state transition graph (STG) of the cell cycle. Unfortuantely the cycle is not a complex attractor in the strict sense, and it can converge into the static attractor of apoptosis. To sample the cyclic part of the basin, we employ the following algorithm:

- We run random order asynchronous update from each \* synchronous attractor state \* (21) as an initial state
  $N$ times doing $n$ time-steps
- If the system reaches any static attractor state, we stop and continue with the next iteration
- We create a statistical aggragate of the state transition graphs obtained in the individual runs
- We remove the static attractor from the final weighted graph to see if a cycle has emerged

In [39]:

```
def evolve_from_state_with_break(model,steps,break_states,initial_state, shuffler=default_shuffler):
    '''
    The function iterates through a given BooleaNet model with a condition of stopping whenever a break state has occured. 
    
    Inputs: model - a BooleanNet model object
            steps - int, the number of desired iterations
            break_states - dict of states with names of break states as keys, and states of the model as values
            initial_state - boolnet2.State or dict, representing the state from which the model should be initialized
            shuffler - suffler function (optional), random order async shuffler by default
            
    Returns: break_state_name (str) - the string identifying the state that caused the break (if any)
             breaking_step (int) - the timestep where the break occured. If no break happened it will equal the steps parameter.
            
    '''
    model.initialize(lambda node: initial_state[node])

    stop_walk=False
    breaking_step=steps
    for i in range(steps):
        model.iterate(1,shuffler=shuffler)
        for break_state_name, break_state in break_states.items():
            if dict(model.states[-1])==break_state:
                #print 'Breaking at %s at step #%d'%(break_state_name,i)
                stop_walk=True
                breaking_step=i
                break
        if stop_walk:
            break
    return break_state_name,breaking_step
```

The configuration of the STG sampling procedure:

In [40]:

```
import networkx as nx
from itertools import combinations, product
from collections import Counter
import cPickle
import pandas as pd

df=pd.DataFrame(columns=['starting_state', 'number_of_steps','steps_taken', 'break_attractor'])

steps=1000
ensemble=10

save_results_to_disk=False
read_results_from_disk=True

save_folder='ROA_update_result_files'
state_collection_file_name="%s/state_collection_steps_%d_ens_%d.pickle"%(save_folder,steps,ensemble)
edge_collection_file_name="%s/edge_collection_steps_%d_ens_%d.pickle"%(save_folder,steps,ensemble)
large_fp_dict_file_name="%s/large_fp_dict_steps_%d_ens_%d.pickle"%(save_folder,steps,ensemble)


if read_results_from_disk:
    with open(state_collection_file_name,"rb") as pickle_in:
        state_collection = cPickle.load(pickle_in)
    with open(edge_collection_file_name,"rb") as pickle_in:
        edge_collection = cPickle.load(pickle_in)
    with open(large_fp_dict_file_name,"rb") as pickle_in:
        large_fp_dict = cPickle.load(pickle_in)
```

If running it for the first time, the a actual simulation is in the cell below:

In [41]:

```
if not read_results_from_disk:

    state_collection=Counter()
    edge_collection=Counter()
    large_fp_dict={}
    fp_dict_absolute_size=0

    for cell_cylce_steps in range(len(cycle_states)):
        initial_state=cycle_states[cell_cylce_steps]
        print 'cell_cycle_state:',cell_cylce_steps
        for ens in range(ensemble):
            break_attr,breaking_step=evolve_from_state_with_break(model=async_model,steps=steps,break_states=static_attractors,initial_state=initial_state,shuffler=default_shuffler) 

            fp_states=async_model.fp()

            state_occurances=Counter(fp_states)
            edge_occurances=Counter([(fp_states[i-1], fp_states[i]) for i in range(len(fp_states))])

            state_collection+=state_occurances
            edge_collection+=edge_occurances

            fp_state_dict=dict(zip(async_model.fp(),async_model.states))
            large_fp_dict= merge_two_dicts(fp_state_dict,large_fp_dict)

            fp_dict_absolute_size+=len(fp_state_dict)

            if breaking_step == steps:
                break_attr=" "
            df=df.append(pd.DataFrame([[fp_states[0], steps, breaking_step, break_attr]], columns=['starting_state', 'number_of_steps','steps_taken', 'break_attractor']))

        
    #This tells all how many states were visited with and without repetition
    print 'Number of states visited:',fp_dict_absolute_size
    print 'Number of unique states visited',len(large_fp_dict)
```

In [42]:

```
#we save the important dictionaries into pickles, so we don't have to re-run the simulations every time
#Memory WARNING! cPickle is an effective "lazy" way to store python objects on the disk, however saving and loading can take up significant amout of memory in case of large objects
if save_results_to_disk:
    with open(state_collection_file_name,"wb") as pickle_out:
        cPickle.dump(state_collection, pickle_out)
    with open(edge_collection_file_name,"wb") as pickle_out:
        cPickle.dump(edge_collection, pickle_out)
    with open(large_fp_dict_file_name,"wb") as pickle_out:
        cPickle.dump(large_fp_dict, pickle_out)
```

In [43]:

```
G=nx.DiGraph()
G.add_edges_from(edge_collection)

node_threshold=1
filtered_nodes=[]
for n in G.nodes():
    if state_collection[n]>node_threshold:
        filtered_nodes.append(n)
        
print 'Size of the filtered network:',len(filtered_nodes)
g=nx.subgraph(G,filtered_nodes)   

print 'Number of connected components:',nx.number_connected_components(g.to_undirected())
print 'Size of the largestcomponent:',len(sorted(nx.connected_components(g.to_undirected()), key = len, reverse=True)[0])
g=G.subgraph(sorted(nx.connected_components(g.to_undirected()), key = len, reverse=True)[0])
print g.number_of_nodes()
```

```
Size of the filtered network: 5027
Number of connected components: 459
Size of the largestcomponent: 4447
4447
```

In [44]:

```
#Taking the largest SCC

g=g.subgraph([c for c in sorted(nx.strongly_connected_components(g),key=len, reverse=True)][0]).copy()
print "Size of the largest SCC:",g.number_of_nodes()
```

```
Size of the largest SCC: 737
```

In [45]:

```
## Calculating the closest sync states for node lables
closest_sync_state={}
for n in g.nodes:
    async_state=large_fp_dict[n]
    overlaps=[]
    for j in range(len(cycle_states)):
        
        #async_state=dict(zip(model_async.nodes,top_PR_states[i]))
        sync_state=cycle_states[j]
        overlap=0
        for s in async_state.keys():
            if async_state[s] == sync_state[s]:
                overlap+=1.
        overlap/=len(async_state.keys())
        overlaps.append(overlap)
        
    overlap_to_sync_cycle[n]=max(overlaps)
    closest_sync_state[n]=overlaps.index(max(overlaps))
```

### Coloring the STG based on the Cell Cycle Phases¶

G1: 4N\_DNA = 0 & Replication = 0   
G2: 4N\_DNA = 1, Replication = 0, U\_Kinetochores = 0, A\_Kinetochores = 0, Ect2 = 0, Cdc20 = 0   
Metaphase: 4N\_DNA = 1, Replication = 0, U\_Kinetochores = 1, A\_Kinetochores = 0, Ect2 = 0 (edited)   
Anaphase: 4N\_DNA = 1, Replication = 0, A\_Kinetochores = 1, Ect2 = 0   
Cytokinesis: Ect2 = 1   
Apoptosis: CAD = 1

In [46]:

```
CC_Phases={'G1':{'f4N_DNA' : 0, 'Replication' : 0,'Ect2': 0},
'G2': {'f4N_DNA' : 1, 'Replication': 0, 'U_Kinetochores':0, 'A_Kinetochores' : 0, 'Ect2': 0, 'Cdc20': 0},
'Metaphase': {'f4N_DNA': 1, 'Replication': 0, 'U_Kinetochores' : 1, 'Ect2' : 0,'A_Kinetochores': 0},
'Anaphase': {'f4N_DNA': 1, 'Replication' :0, 'A_Kinetochores': 1, 'Ect2': 0},
'Cytokinesis': {'Ect2' : 1},
'Replication':{'Replication':1}}

CC_Phase_colors={'G1':'blue', 'G2':'darkviolet', 'Metaphase':'green', 'Anaphase':'crimson', 'Cytokinesis':'black','Replication':'yellow'}
```

In [47]:

```
phase_colors={}
for n in g.nodes():
    n_state=large_fp_dict[n]
    color='grey'
    for ccp,marker in CC_Phases.items():
        if np.prod([n_state[m]==bool(marker[m]) for m in marker.keys()])==1: #all marker nodes match
            color=CC_Phase_colors[ccp]
    phase_colors[n]=color
    
# drawing nodes and edges separately so we can capture collection for colobar
plt.figure(figsize=(20,20))
pos = nx.kamada_kawai_layout(g)
ec = nx.draw_networkx_edges(g, pos, alpha=0.2,with_labels=False,width=[float(edge_collection[i]) for i in g.edges()])
#lc = nx.draw_networkx_labels(g,pos,{i:closest_sync_state[i] for i in g.nodes()})
nc = nx.draw_networkx_nodes(g, pos,  node_color=[phase_colors[i] for i in g.nodes()], node_size=[state_collection[j]*5 for j in g.nodes()],alpha=0.7)
markers = [plt.Line2D([0,0],[0,0],color=color, marker='o', linestyle='') for color in CC_Phase_colors.values()]
plt.legend(markers, CC_Phase_colors.keys(), numpoints=1)
plt.axis('off')
plt.show()
```

## Meging states based on a equal subsets of nodes:¶

In [48]:

```
relevance_set={"p21","pRB","p27Kip1","Myc","CyclinD1","E2F1","CyclinE","CyclinA_mRNA","Emi1","FoxM1","Cdc25A","CyclinA","Wee1","UbcH10","CyclinB","Cdc25B","Plk1","Cdc25C","Cdk1","pAPC","Cdc20","Cdh1","Replication","ATR","CHK1","f4N_DNA","U_Kinetochores","Mad2","A_Kinetochores","Plk1_H","Ect2","ORC","Cdc6","Cdt1","Pre_RC","geminin"}
```

In [49]:

```
G=g.copy()
merged=False

while not merged:
    merged=True
    #cleaning a bit
    for e in G.edges():
        if e[0]==e[1]:
            G.remove_edge(e[0],e[1])
    for i in G.nodes():
        for j in G.nodes():
            if i==j:
                continue
            if np.prod([large_fp_dict[i][x]==large_fp_dict[j][x] for x in relevance_set]):
                merged=False
                
                #CONTRACTION
                G= nx.contracted_nodes(G, i, j,self_loops=False)

        if merged==False:
            break


el=np.array(list(G.edges()))
el_counted=np.unique(el,axis=0,return_counts=True)
edges_and_weights={tuple(el_counted[0][e]):el_counted[1][e] for e in range(len(el_counted[0])) if el_counted[0][e][0]!= el_counted[0][e][1]}
el=list(set(G.edges()))
G=nx.DiGraph()
G.add_edges_from(edges_and_weights)
```

In [50]:

```
# drawing nodes and edges separately so we can capture collection for colobar
plt.figure(figsize=(20,20))
pos = nx.kamada_kawai_layout(G)
ec = nx.draw_networkx_edges(G, pos, alpha=0.2,with_labels=False,width=[2 for i in G.edges()])
lc = nx.draw_networkx_labels(G,pos,{i:closest_sync_state[i] for i in G.nodes()})
nc = nx.draw_networkx_nodes(G, pos,  node_color=[phase_colors[i] for i in G.nodes()], node_size=[state_collection[j]*20 for j in G.nodes()], cmap=plt.cm.jet,alpha=0.7)
markers = [plt.Line2D([0,0],[0,0],color=color, marker='o', linestyle='') for color in CC_Phase_colors.values()]
plt.legend(markers, CC_Phase_colors.keys(), numpoints=1)
plt.axis('off')
plt.show()
```

## Sampling the STG with biased updating scheme¶

In [51]:

```
def biased_random_shuffler( lines, model, update_first, update_last):
    """
    This shuffler produces a biased random order, for timestep t+1 based on the state of the model in time t
    
    Inputs:
    lines - list, BooleanNet rules - see BooleanNet shuffler documentation
    model - BooleanNet model instance, the model being iterated
    update first - list of node-state pair tuples. The nodes in this list will be updated first if they are in the state given in the tuple. If multiple states are true, the order of the list is followed.
    update first - list of node-state pair tuples. The nodes in this list will be updated last if they are in the state given in the tuple. If multiple states are true, the *reverse* order of the list is followed.
    
    Returns: 
    temp - a shuffeled list of the update rules
    """
    
    
    temp = lines[:]
    random.shuffle( temp )

    state=model.states[-1]

    
    for n,s in update_first[::-1]:
        if int(state[n])==s:
            #finrding the line
            for line_index in range(len(lines)):
                if '%s * = '%n in temp[line_index]:
                    break
            #moving to the front
            temp.insert(0, temp.pop(line_index))
    for n,s in update_last[::-1]:
        if int(state[n])==s:
            #finrding the line
            for line_index in range(len(lines)):
                if '%s * = '%n in temp[line_index]:
                    break
            #moving to the back
            temp.insert(len(temp), temp.pop(line_index))

    return temp
```

In [52]:

```
update_first=[('Pre_RC',1),
              ('Replication',0),
              ('U_Kinetochores',0),
              ('A_Kinetochores',1),
              ('Plk1_H',1),
              ('CyclinB',1),
              ('Cdc20',0)]

update_last=[('Replication',1),
             ('f4N_DNA',0),
             ('f4N_DNA',1),
             ('Ect2',0),
             ('A_Kinetochores',0),
             ('U_Kinetochores',1),
             ('CyclinE',1),
             ('FoxM1',1),
             ('Cdc20',1),
             ('Plk1_H',0)]
```

In [53]:

```
async_model = boolean2.Model(rules, mode='async')
async_model.initialize(lambda node: initial_state[node])
biased_shuffler= lambda lines: biased_random_shuffler(lines,async_model,update_first,update_last)
```

In [54]:

```
import networkx as nx
from itertools import combinations, product
from collections import Counter

nr_of_steps=1000

start_state=cycle_states[0]
async_model.initialize(lambda node: start_state[node])
async_model.iterate(nr_of_steps,shuffler=biased_shuffler)

TG=TransGraph(logfile='states.txt')
TG.add(async_model.states)
G=TG.graph
print "Number of unique states visited:",G.number_of_nodes()
print "Number of unique transitions:",G.number_of_edges()
```

```
Number of unique states visited: 220
Number of unique transitions: 221
```

In [55]:

```
biased_shuffler?
```

In [56]:

```
selected_states=async_model.states[:100]
hamming_dist_top_async_sync=np.zeros((len(selected_states),len(cycle_states)))
for i in range(len(selected_states)):
    for j in range(len(cycle_states)):
        
        #async_state=dict(zip(model_async.nodes,top_PR_states[i]))
        async_state=selected_states[i]
        sync_state=cycle_states[j]
        
        for s in async_state.keys():
            if async_state[s] == sync_state[s]:
                hamming_dist_top_async_sync[i,j]+=1.
                
hamming_dist_top_async_sync/=len(async_model.nodes)
plt.figure(figsize=(20,100))
plt.imshow(hamming_dist_top_async_sync, cmap=plt.cm.RdPu, aspect='auto')
plt.xlabel('Syncronous Attractor States',fontsize=20)
plt.ylabel('Async State Transition',fontsize=20)
clb=plt.colorbar()
clb.set_label('Normalized overlap',fontsize=20)
```

### Using the same procedure as in case of the random order asynchrnous update we sample the STG, the only difference being the update scheme.¶

In [57]:

```
shuffler=biased_shuffler
```

In [58]:

```
import networkx as nx
from itertools import combinations, product
from collections import Counter
import cPickle
import pandas as pd

df=pd.DataFrame(columns=['starting_state', 'number_of_steps','steps_taken', 'break_attractor'])

steps=1000
ensemble=10

save_results_to_disk=False
read_results_from_disk=True

save_folder='BROA_update_result_files'
state_collection_file_name="%s/state_collection_steps_%d_ens_%d.pickle"%(save_folder,steps,ensemble)
edge_collection_file_name="%s/edge_collection_steps_%d_ens_%d.pickle"%(save_folder,steps,ensemble)
large_fp_dict_file_name="%s/large_fp_dict_steps_%d_ens_%d.pickle"%(save_folder,steps,ensemble)


if read_results_from_disk:
    with open(state_collection_file_name,"rb") as pickle_in:
        state_collection = cPickle.load(pickle_in)
    with open(edge_collection_file_name,"rb") as pickle_in:
        edge_collection = cPickle.load(pickle_in)
    with open(large_fp_dict_file_name,"rb") as pickle_in:
        large_fp_dict = cPickle.load(pickle_in)
```

In [59]:

```
if not read_results_from_disk:

    state_collection=Counter()
    edge_collection=Counter()
    large_fp_dict={}
    fp_dict_absolute_size=0

    for cell_cylce_steps in range(len(cycle_states)):
        initial_state=cycle_states[cell_cylce_steps]
        print 'cell_cycle_step:',cell_cylce_steps
        for ens in range(ensemble):
            break_attr,breaking_step=evolve_from_state_with_break(model=async_model,steps=steps,break_states=static_attractors,initial_state=initial_state,shuffler=shuffler) 

            fp_states=async_model.fp()

            state_occurances=Counter(fp_states)
            edge_occurances=Counter([(fp_states[i-1], fp_states[i]) for i in range(len(fp_states))])

            state_collection+=state_occurances
            edge_collection+=edge_occurances

            fp_state_dict=dict(zip(async_model.fp(),async_model.states))
            large_fp_dict= merge_two_dicts(fp_state_dict,large_fp_dict)

            fp_dict_absolute_size+=len(fp_state_dict)

            if breaking_step == steps:
                break_attr=" "
            df=df.append(pd.DataFrame([[fp_states[0], steps, breaking_step, break_attr]], columns=['starting_state', 'number_of_steps','steps_taken', 'break_attractor']))


    #This tells all how many states were visited with and without repetition
    print 'Number of states visited:',fp_dict_absolute_size
    print 'Number of unique states visited',len(large_fp_dict)
```

In [60]:

```
#we save the important dictionaries into pickles, so we don't have to re-run the simulations every time
#Memory WARNING! cPickle is an effective "lazy" way to store python objects on the disk, however saving and loading can take up significant amout of memory in case of large objects
if save_results_to_disk:
    with open(state_collection_file_name,"wb") as pickle_out:
        cPickle.dump(state_collection, pickle_out)
    with open(edge_collection_file_name,"wb") as pickle_out:
        cPickle.dump(edge_collection, pickle_out)
    with open(large_fp_dict_file_name,"wb") as pickle_out:
        cPickle.dump(large_fp_dict, pickle_out)
```

In [61]:

```
G=nx.DiGraph()
G.add_edges_from(edge_collection)

node_threshold=1
filtered_nodes=[]
for n in G.nodes():
    if state_collection[n]>node_threshold:
        filtered_nodes.append(n)
        
print 'Size of the filtered network:',len(filtered_nodes)
g=nx.subgraph(G,filtered_nodes)   

print 'Number of connected components:',nx.number_connected_components(g.to_undirected())
print 'Size of the largestcomponent:',len(sorted(nx.connected_components(g.to_undirected()), key = len, reverse=True)[0])
g=G.subgraph(sorted(nx.connected_components(g.to_undirected()), key = len, reverse=True)[0])
print g.number_of_nodes()
```

```
Size of the filtered network: 3547
Number of connected components: 265
Size of the largestcomponent: 3234
3234
```

In [62]:

```
#Taking the largest SCC

g=g.subgraph([c for c in sorted(nx.strongly_connected_components(g),key=len, reverse=True)][0]).copy()
print "Size of the largest SCC:",g.number_of_nodes()
```

```
Size of the largest SCC: 1498
```

In [63]:

```
## Calculating the closest sync states for node lables
closest_sync_state={}
for n in g.nodes:
    async_state=large_fp_dict[n]
    overlaps=[]
    for j in range(len(cycle_states)):
        
        #async_state=dict(zip(model_async.nodes,top_PR_states[i]))
        sync_state=cycle_states[j]
        overlap=0
        for s in async_state.keys():
            if async_state[s] == sync_state[s]:
                overlap+=1.
        overlap/=len(async_state.keys())
        overlaps.append(overlap)
        
    overlap_to_sync_cycle[n]=max(overlaps)
    closest_sync_state[n]=overlaps.index(max(overlaps))
```

In [64]:

```
phase_colors={}
for n in g.nodes():
    n_state=large_fp_dict[n]
    color='grey'
    for ccp,marker in CC_Phases.items():
        if np.prod([n_state[m]==bool(marker[m]) for m in marker.keys()])==1: #all marker nodes match
            color=CC_Phase_colors[ccp]
    phase_colors[n]=color
    
# drawing nodes and edges separately so we can capture collection for colobar
plt.figure(figsize=(20,20))
pos = nx.kamada_kawai_layout(g)
ec = nx.draw_networkx_edges(g, pos, alpha=0.2,with_labels=False,width=[float(edge_collection[i]) for i in g.edges()])
#lc = nx.draw_networkx_labels(g,pos,{i:closest_sync_state[i] for i in g.nodes()}, fontsize=8)
nc = nx.draw_networkx_nodes(g, pos,  node_color=[phase_colors[i] for i in g.nodes()], node_size=[state_collection[j]*5 for j in g.nodes()],alpha=0.7)
markers = [plt.Line2D([0,0],[0,0],color=color, marker='o', linestyle='') for color in CC_Phase_colors.values()]
plt.legend(markers, CC_Phase_colors.keys(), numpoints=1)
plt.axis('off')
plt.show()
```

In [65]:

```
relevance_set={"p21","pRB","p27Kip1","Myc","CyclinD1","E2F1","CyclinE","CyclinA_mRNA","Emi1","FoxM1","Cdc25A","CyclinA","Wee1","UbcH10","CyclinB","Cdc25B","Plk1","Cdc25C","Cdk1","pAPC","Cdc20","Cdh1","Replication","ATR","CHK1","f4N_DNA","U_Kinetochores","Mad2","A_Kinetochores","Plk1_H","Ect2","ORC","Cdc6","Cdt1","Pre_RC","geminin"}


G=g.copy()
merged=False

while not merged:
    merged=True
    #cleaning a bit
    for e in G.edges():
        if e[0]==e[1]:
            G.remove_edge(e[0],e[1])
    for i in G.nodes():
        for j in G.nodes():
            if i==j:
                continue
            if np.prod([large_fp_dict[i][x]==large_fp_dict[j][x] for x in relevance_set]):
                merged=False
                
                #CONTRACTION
                G= nx.contracted_nodes(G, i, j,self_loops=False)

        if merged==False:
            break


el=np.array(list(G.edges()))
el_counted=np.unique(el,axis=0,return_counts=True)
edges_and_weights={tuple(el_counted[0][e]):el_counted[1][e] for e in range(len(el_counted[0])) if el_counted[0][e][0]!= el_counted[0][e][1]}
el=list(set(G.edges()))
G=nx.DiGraph()
G.add_edges_from(edges_and_weights)
```

In [66]:

```
# drawing nodes and edges separately so we can capture collection for colobar
plt.figure(figsize=(20,20))
pos = nx.kamada_kawai_layout(G)
ec = nx.draw_networkx_edges(G, pos, alpha=0.2,with_labels=False,width=[2 for i in G.edges()])
lc = nx.draw_networkx_labels(G,pos,{i:closest_sync_state[i] for i in G.nodes()})
nc = nx.draw_networkx_nodes(G, pos,  node_color=[phase_colors[i] for i in G.nodes()], node_size=[state_collection[j]*20 for j in G.nodes()], cmap=plt.cm.jet,alpha=0.7)
markers = [plt.Line2D([0,0],[0,0],color=color, marker='o', linestyle='') for color in CC_Phase_colors.values()]
plt.legend(markers, CC_Phase_colors.keys(), numpoints=1)
plt.axis('off')
plt.show()
```

# In-Silico experiments¶

In [67]:

```
def plot_state_succession_KO(states,nodes,state_labels=None,title=None, fontsizex=10, color_mask=None):

    '''
    docstring
    '''
    from matplotlib import pyplot as plt
    import matplotlib
    if nodes==None:
        nodes=states[0].keys()
    state_transition=[]
    for s in states:
        state_transition.append([int(s[k]) for k in nodes])
    if color_mask.any()!=None:
        state_transition=state_transition+color_mask
    cmap = matplotlib.colors.ListedColormap(['blue', 'orange','black', 'white'])
    bounds = [0, 1, 2, 3,4]
    norm = matplotlib.colors.BoundaryNorm(bounds, cmap.N)
    steps=len(state_transition)
    plt.figure(figsize=(len(nodes),steps))
    plt.imshow(state_transition, interpolation='none',cmap=cmap,norm=norm)
    ax = plt.gca()
    plt.xticks(range(len(nodes)),nodes, fontsize=fontsizex)   
    if state_labels==None:
        plt.yticks(range(steps),fontsize=12)
    else:
        plt.yticks(range(steps),state_labels,fontsize=12)
    if title!=None:
        plt.title(title)
    ax.set_yticks(np.arange(-.5, steps,1),minor=True)
    plt.grid(which='minor', color='black', linestyle='-', linewidth=2)
    plt.show()
    return state_transition
```

### Set the parameters of the experiment¶

In [68]:

```
steps = 200
update_mode='async' #the other option is: async, which will result in random order async update
biased_async=False
```

In [69]:

```
if biased_async:
    biased_shuffler= lambda lines: biased_random_shuffler(lines,update_first,update_last, model)
    update_first=[('Pre_RC',1),
              ('Replication',0),
              ('U_Kinetochores',0),
              ('A_Kinetochores',1),
              ('Plk1_H',1),
              ('CyclinB',1),
              ('Cdc20',0)]

    update_last=[('Replication',1),
                 ('f4N_DNA',0),
                 ('f4N_DNA',1),
                 ('Ect2',0),
                 ('A_Kinetochores',0),
                 ('U_Kinetochores',1),
                 ('CyclinE',1),
                 ('FoxM1',1),
                 ('Cdc20',1),
                 ('Plk1_H',0)]
```

In [70]:

```
input_mode={'node':'GF_High','node_state':True, 'input_start':0 ,'input_stop':steps,'p_input':0.8 }

manipulated_nodes={'p110_H': True,'PI3K_H':True }
start_times= {'p110_H': 20, 'PI3K_H':20}
flip_probabilities = {'p110_H': 0.8, 'PI3K_H':0.8}
```

In [71]:

```
break_at_state=True
break_state = static_attractors['attr_3'] #apoptosis
```

### Initializing and iterating throug the experiment¶

In [72]:

```
model_name='full_cell_cycle'
rules=read_rules_text(model_name)
model = boolean2.Model(rules, mode=update_mode)
init_array=np.loadtxt('cell_cycle_init.txt',dtype=str)
initial_state=dict(zip(init_array[:,0],init_array[:,1].astype(bool)))
#print initial_state
model.initialize(lambda node: initial_state[node])

node_order=["GF_High","GF","RTK","Grb2","SOS","Ras","RAF","MEK","ERK","mTORC2","PI3K","PIP3","PDK1","AKT_B","p110_H","PI3K_H","AKT_H","FoxO3","PLCgamma","IP3","Ca2","NeddL4","FoxO1","p21_mRNA","TSC2","PRAS40","DAG","Rheb","mTORC1","S6K","eIF4E","GSK3","p21","pRB","p27Kip1","Myc","CyclinD1","E2F1","CyclinE","ORC","Cdc6","Cdt1","Pre_RC","geminin","CyclinA_mRNA","Emi1","FoxM1","Cdc25A","CyclinA","Wee1","UbcH10","CyclinB","Cdc25B","Plk1","Cdc25C","Cdk1","pAPC","Cdc20","Cdh1","Replication","ATR","CHK1","f4N_DNA","U_Kinetochores","Mad2","A_Kinetochores","Plk1_H","Ect2","Trail","DR4_5","Casp8","Casp2","MCL_1","BCLXL","BCL2","BAD","BIK","BIM","BID","BAK","BAX","Cyto_C","SMAC","IAPs","Casp9","Casp3","CAD"]

   
def evolve_with_node_manipulation(model,steps, manipulated_nodes, start_times, flip_probabilities,node_order=None,input_mode=None, break_state=None, shuffler=None):
    
    '''
    The function iterates through a given BooleaNet model with optional conditions of node manipulation, such as:
        - propbabilistic input pulse 
        - probabilistic node knockout/overexpression
        - halting the iteration when a certain state (usually a point attractor) is reached, similar to the evolve_with_break() function.
    
    Inputs: model - a BooleanNet model object
            steps - int, the number of iterations (timesteps)
            manipulated_nodes - dict of nodes with node names as keys and states as values. The True state corresponds to overexpression, the False state to knockout.
            start_times - dict of nodes with node names as keys and time steps as values. The timestep assigned to each node will determine at wich point the manipulation will begin in the evolution
            flip_probabilities - dict of nodes with node names as keys and probabilities of enforcing the assigned values at each step. 
            input_mode (optional) - the manipulation of a single input
            break_state (optional)- a state where the evolution of the model is halted
            shuffler (optional)- suffler function, random order async shuffler by default
            
            
            
    Returns: t - int, the timestep where the break occured. If no break happened it will equal the steps parameter.
             color_mask - numpy.array encoding each successful overexpression/knockout. Useful for coloring the evolution when plotting the trajectory
             
    Example for the inputs:
        input_mode={'node':'GF_High','node_state':True, 'input_start':0 ,'input_stop':steps,'p_input':0.8 }
        manipulated_nodes={'p110_H': True,'PI3K_H':True }
        start_times= {'p110_H': 20, 'PI3K_H':20}
        flip_probabilities = {'p110_H': 0.8, 'PI3K_H':0.8}
    '''
    
    
    if node_order==None:
        node_order= model.nodes
        
    color_mask=np.zeros((steps+1, len(node_order)))
    
    for t in range(steps):
        if break_state!=None and dict(model.states[-1])==break_state:
            print 'break state reached at timestep %d, stopping iteration'%t
            break
        
        if input_mode!=None and t>=input_mode['input_start'] and t<=input_mode['input_stop']:
            #flip a biassed coin
            if np.random.binomial(1,input_mode['p_input']):
                model.states[-1][input_mode['node']]=input_mode['node_state']
            else:
                model.states[-1][input_mode['node']]=not input_mode['node_state']

        for node,forced_state in manipulated_nodes.items():
            ni=node_order.index(node)
            if t>=start_times[node]:
                #flip a biassed coin
                if np.random.binomial(1,flip_probabilities[node]):
                    model.states[-1][node]=forced_state
                    color_mask[t,ni]=int(forced_state)+2
        if shuffler!=None:
            model.iterate(1, shuffler=shuffler)
        else:
            model.iterate(1)
            
    return t, color_mask[:len(model.states),:]

t_end, color_mask=evolve_with_node_manipulation(model,steps, manipulated_nodes, start_times, flip_probabilities,node_order,input_mode,break_state)
print t_end
```

```
break state reached at timestep 88, stopping iteration
88
```

In [73]:

```
x=plot_state_succession_KO(model.states[:],nodes=node_order, color_mask=color_mask)
```
